# MaRAP2‐4, a waterlogging‐responsive ERF from *Mentha,* regulates bidirectional sugar transporter *AtSWEET10* to modulate stress response in *Arabidopsis*

**DOI:** 10.1111/pbi.12762

**Published:** 2017-07-25

**Authors:** Ujjal J. Phukan, Gajendra Singh Jeena, Vineeta Tripathi, Rakesh Kumar Shukla

**Affiliations:** ^1^ Biotechnology Division CSIR‐Central Institute of Medicinal and Aromatic Plants (CSIR‐CIMAP) Lucknow India; ^2^ CSIR‐Central Drug Research Institute (CSIR‐CDRI) Lucknow India

**Keywords:** SWEET, DRE, ERF, RAP2‐4, Abiotic stress, Antioxidant

## Abstract

As waterlogging and successive events severely influence growth and development of economically important plants, we attempted to characterize the role of a waterlogging‐responsive group I (A‐6) ethylene response factor (MaRAP2‐4) from *Mentha arvensis*. Waterlogging, ethylene and methyl jasmonate rapidly induced the expression of *MaRAP2‐4*. MaRAP2‐4 interacted with multiple *cis*‐elements like dehydration response elements (DRE1/2), anoxia/jasmonic acid response element (JARE) and GCC box showing its involvement in multiple responses. MaRAP2‐4 localizes in the nucleus and acts as a transcriptional activator. Truncation and internal deletion identified a 20 amino acids potential transactivation domain (PLPSSVDAKLEAICQSLAIN) in MaRAP2‐4. MaRAP2‐4 transgenic *Arabidopsis* showed enhanced waterlogging and subsequent oxidative stress tolerance. Microarray analysis revealed that within up‐regulated genes 483, 212 and 132 promoters carry either single or multiple copies of DRE, JARE and GCC *cis*‐element/s, respectively. Within these promoters, a large section belongs to carbohydrate metabolism/transport, including many SWEET transporters. Further analysis showed MaRAP2‐4 specifically targets two positions in *AtSWEEET10* promoter carrying DRE and/or GCC box that might regulate carbohydrate availability and waterlogging tolerance. These results demonstrate that MaRAP2‐4 is a positive regulator of waterlogging tolerance, and as energy‐consuming processes such as carbohydrate biosynthesis are reduced under waterlogging‐induced hypoxia, sugar transport through SWEETs may be the primary option to make sugar available to the required tissue.

## Introduction

Waterlogging is becoming a critical problem around the world because of the rapid climatic fluctuations. During prolonged waterlogging, fertile arable lands are affected by severe hypoxia alleviating growth and yield of commercially valuable plants. Important crops such as rice, barley, maize, soya bean and wheat suffer huge loss because of waterlogging (Bates *et al*., 2008; Rathore and Warsi, 1998; Scott *et al*., 1989; Setter *et al*., 1999). Waterlogging induces gradual decline in oxygen partial pressure and rapid gas diffusion, impairing normal cellular physiology and metabolism (Nilsen and Orcutt, 1997). To survive waterlogging stress and to regulate different adaptive responses, plants modulate various transcriptional and metabolic changes (Bailey‐serres and Colmer, 2014; Bailey‐Serres and Voesenek, 2008; Kim *et al*., 2015; Phukan *et al*., 2015; Voesenek *et al*., 1996). Anaerobic ethanolic fermentation, glycolysis coupled with NAD regenerative pathways and alanine production mostly fulfil the ATP need under waterlogging (Ricoult *et al*., 2006). Waterlogging‐induced anaerobic respiration leads to over‐reduction of photosynthetic electron transport chain (pETC). It results in induced formation of superoxide radicals and singlet oxygen species. Initial ROS and NO generation help in adaptive responses such as aerenchyma and adventitious root formation, but excess of it causes severe oxidative damage under waterlogging stress (Igamberdiev and Hill, 2004; Planchet and Kaiser, 2006; Steffens *et al*., 2012). Endogenous antioxidant enzymes and nonenzymatic scavengers counteract this oxidative stress.

Carbohydrate availability, metabolism and transport not only regulate waterlogging tolerance but other abiotic stress tolerance as well (Rosa *et al*., 2009). Tolerant rice (FR13A, Kalaputia) varieties contain higher amount of nonstructural carbohydrate than susceptible (Sarala and IR42) varieties (Panda and Sarkar, 2014). Some plants conserve carbohydrate content to survive through the stress like *Mentha piperita* (which may be a problem in prolonged waterlogging stress), while some utilize this reserve to rapidly grow and escape the stress like *M. arvensis* (Phukan *et al*., 2014). It is reported that efficient starch transport in mung bean, cotton and *Cynodon* regulated waterlogging tolerance (Kuai *et al*., 2014; Li *et al*., 2015; Sairam *et al*., 2009). One class of bidirectional sugar transporter involved in various developmental and stress responses is SWEET (Sugar Will Eventually be Exported Transporter) proteins (Quirino *et al*., 2001). Many members from rice and *Arabidopsis* such as AtSWEET10/AtSWEET8/OsSWEET11 can transport sugars across the plasma membrane. SWEET proteins like OsSWEET11/14 are targeted by bacterial pathogens for sugar need and virulence (Chen *et al*., 2010). Despite wide range of biological importance, role of many SWEETs has remained elusive, which needs further study and exploration in different responses.

Plants respond differently to waterlogging stress based on their genotype, age and severity of the stress. Flood tolerance of rice FR13A and C9285 is regulated at the genetic level. FR13A possesses an ERF transcription factor (TF) locus SUB1 (submergence1) which restrict shoot elongation and carbohydrate consumption, while C9285 possesses two ERFs SNORKEL1 and SNORKEL2 which induce gibberellin‐mediated internode elongation to provide submergence tolerance (Fukao and Bailey‐Serres, 2008; Hattori *et al*., 2009). Some ERFs such as RAP2‐2, HRE1, HRE2 and RAP2‐12 are reported to be involved in hypoxia response, while only RAP2‐6L is characterized in response to waterlogging stress in *Arabidopsis* (Liu et al., 2012). In our previous study, we isolated an expressed sequence tag (EST) encoding ERF TF from subtractive hybridization of waterlogging‐tolerant *M. arvensis* against intolerant *M. piperita* (Phukan *et al*., 2014). To further assess the waterlogging response of this EST, we focused on functional characterization of full‐length ORF (MaRAP2‐4) in this report. We investigated DNA binding and transactivation property of this TF, also analysed its transactivation domain. We generated transgenic *Arabidopsis* lines to study biochemical and physiological response of them under waterlogging stress. To study transcriptional response, we did microarray analysis that identified many sugar metabolism/transport responsive genes carrying MaRAP2‐4 interacting *cis*‐elements. Further, we investigated one of the highly expressed transporter *AtSWEET10 in vivo* and *in vitro* whether it is a downstream target of MaRAP2‐4. Also we assessed the response of transgenic lines in drought and salt stress.

## Results

### MaRAP2‐4 was highly induced under waterlogging stress

**Figure 1:**
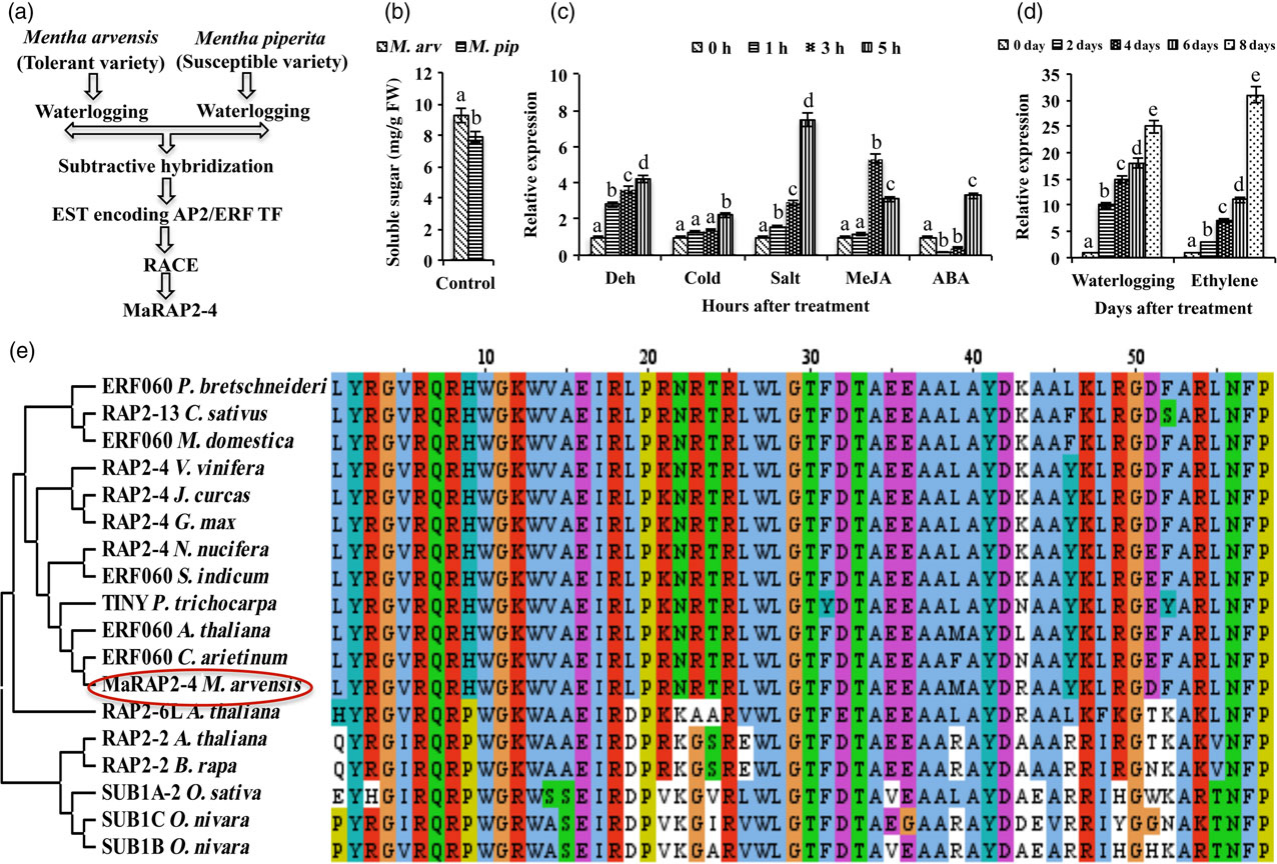
Expression and phylogenetic analysis of MaRAP2‐4. (a) Through SSH of waterlogging‐tolerant *Mentha arvensis* and susceptible *M. piperita,* an EST encoding ERF TF was obtained. Through 5′ and 3′ RACE, it was made full length and named as MaRAP2‐4. (b) Higher soluble sugars content (mg/g FW) of *M. arvensis* in comparison to *M. piperita* under control conditions. (c) Induced relative expression of MaRAP2‐4 after 1, 3 and 5 h of drought, salt, cold, ABA and MeJA treatment. (d) Induced relative expression of MaRAP2‐4 after 2, 4, 6 and 8 days of waterlogging and ethylene treatment. (e) MEGA6.06 phylogenetic tree of MaRAP2‐4 with hypoxia/waterlogging‐responsive and other ERFs. Represented values were calculated from mean of three independent experiment performed. The error bars show mean ± SD. The letters above column indicates a statistically significant difference for the data of *M. arvensis* treated (different stress and hormone treatments) samples at different time intervals. Different letters indicate a significant difference between columns (*P* < 0.05) while same letters indicate no significant difference.

Suppression subtractive hybridization (SSH) of *M. arvensis* (tolerant variety) and *M. piperita* (susceptible variety) generated an EST encoding ERF TF (GenBank accession‐JZ468949) as shown in Figure 1a (Phukan *et al*., 2014). The list of other ESTs/DEG from SSH analysis is mentioned in Data S2. We also observed that total soluble sugars content of *M. arvensis* was more in comparison to *M. piperita*, which might be one of the reasons for enhanced waterlogging tolerance of *M. arvensis* (Figure 1b). We did RACE to obtain 5′ and 3′ flanking regions of the EST that was submitted to GenBank (BankIt) NCBI as *MaRAP2‐4* (accession–KX267734). Full‐length ORF with its amino acid alignment is shown in Figure S1. It was showing induced expression in response to abiotic stresses (waterlogging, drought, cold and salt) and hormone treatments (ABA, MeJA and ethylene) in *M. arvensis* (Figure 1c, d). The notable point is that MaRAP2‐4 was showing maximum induction after waterlogging and ethylene treatments. MEGA6.06 phylogenetic analysis and Clustal Omega DBD alignment of hypoxia‐responsive ERFs (group VII), and some other ERFs revealed that MaRAP2‐4 is highly similar to RAP2‐4/ERF60 and belongs to group I (A‐6). It did not show significant homology with hypoxia‐responsive group VII ERFs (Figure 1e) and did not contain Met‐Cys motif involved in N‐end rule protein destabilization.

### MaRAP2‐4 interacted with multiple *cis‐*elements and has a 20 amino acids transactivation domain

**Figure 2:**
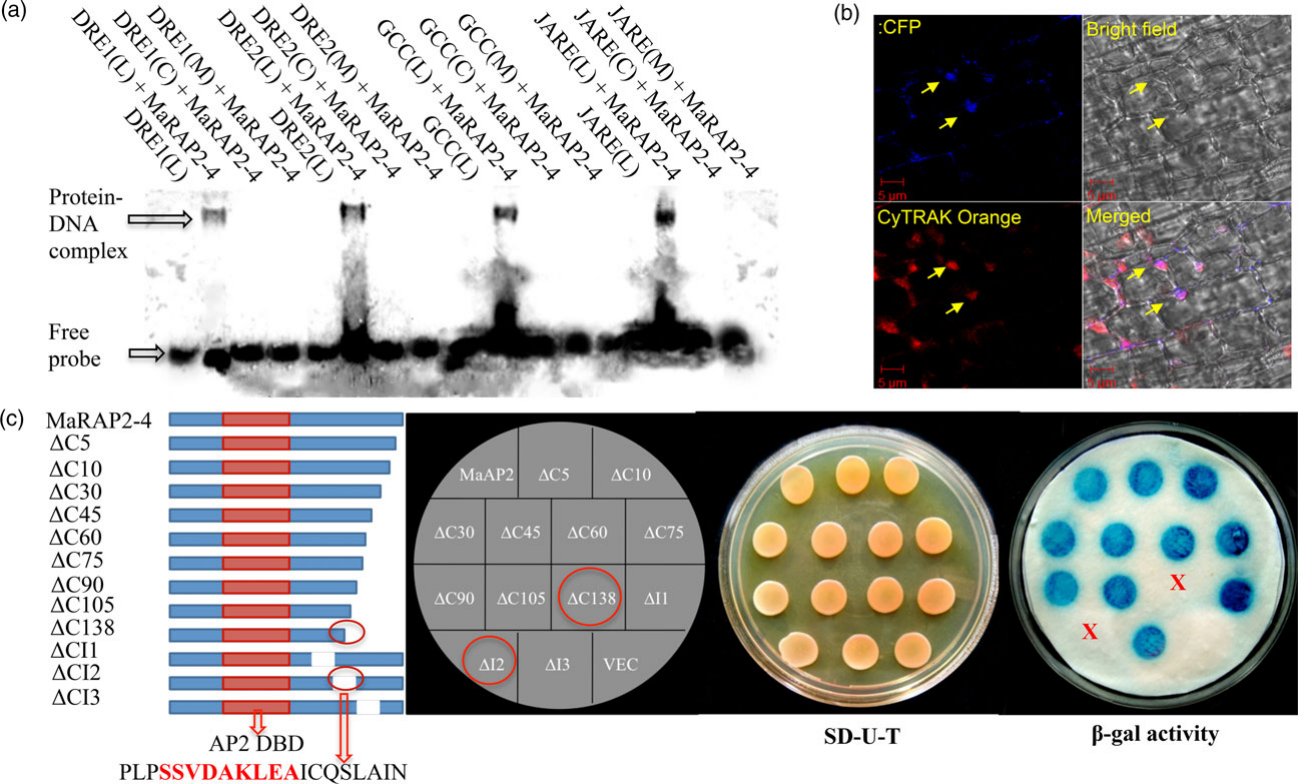
DNA binding property and transactivation domain mapping of MaRAP2‐4. (a) EMSA of MaRAP2‐4 with DRE1/2, JARE and GCC box showed positive interaction (L—DIG‐labelled, C—cold/unlabelled, M—mutated/substituted). (b) Nuclear localization of MaRAP2‐4. Left upper image shows MaRAP2‐4‐CFP fluorescence. Left lower image shows CYTRAK Orange fluorescence that stains both nucleus and cytoplasm with differential intensity. Right upper image shows bright field illumination. Left lower image shows the merged view. The arrows indicate nuclear localization of MaRAP2‐4. (c) To map the transactivation domain, nine C’ terminal truncation and three internal deletions are made. Constructs were transformed, spotted and β‐galactosidase assay was performed. No colour formation in the colonies carrying ΔC138 truncation and ΔCI2 deletion confirms the presence of 20 amino acid transactivation domain in that region. Red coloured region within the transactivation domain highlights the nine amino acid transactivation domain predicted by 9aaTAD Prediction Tool (http://www.med.muni.cz/9aaTAD/index.php).

To study the DNA binding affinity of MaRAP2‐4, we designed several probes with known ERF‐interacting or anoxia‐responsive *cis‐*elements (Figure S3). We observed specific interaction of MaRAP2‐4 with DRE1 (GCCGAC–dehydration response element 1), DRE2 (TACCGACAT), GCC box (AGCCGCC–involved in biotic response) and JARE (CATGAATT–anoxia/jasmonic acid response element) *cis‐*elements, while no interaction was observed with single nucleotide‐substituted probes (Figure 2a). This strong binding affinity with multiple *cis‐*elements could result in regulation of several downstream genes simultaneously to provide multiple responses. JARE was reported to be the most occurred *cis*‐element in the promoters of anoxia‐responsive genes in rice; a condition follows after prolonged waterlogging stress (Mohanty *et al*., 2012).

TFs bind to the respective *cis‐*elements present in the promoter of target genes by their DBD and activate transcription of them by their transactivation domain. To study this at first, we observed the subcellular localization of MaRAP2‐4. CFP‐tagged MaRAP2‐4 was specifically localized in the nucleus (Figure 2b). Then to study transactivation property, we transformed MaRAP2‐4‐GAL4‐DBD in yeast Y187 and observed that it positively transactivate the reporter gene LacZ to produce β‐galactosidase, which gave blue colour on X‐gal application (Figure S4). Further to study and identify its transactivation domain, we made nine truncations from its 3′ end and observed their transactivation property by β‐galactosidase assay. The constructs were named as shown in Figure 2c. All constructs up to ∆C105 truncation showed positive transactivation of LacZ but ∆C138 did not. So it became evident that transactivation domain lies ahead of ∆C105 region. So we made an internal deletion (∆CI2) of 20 amino acids covering that region and two more internal deletions (∆CI1/3) flanking ∆CI2 as shown in Figure 2c. We observed that ∆CI1 and ∆CI3 were able to transactivate LacZ, while ∆CI2 could not which proved that ∆CI2 (PLPSSVDAKLEAICQSLAIN) is the probable transactivation domain. We further analysed the 20 amino acid region with ‘Nine Amino Acids Transactivation Domain 9aaTAD Prediction Tool’—http://www.med.muni.cz/9aaTAD/index.php (Piskacek *et al*., 2007), which showed a potential nine amino acid transactivation domain (SSVDAKLEA) within it.

### MaRAP2‐4 *Arabidopsis* transgenic lines showed more tolerance to waterlogging stress

**Figure 3:**
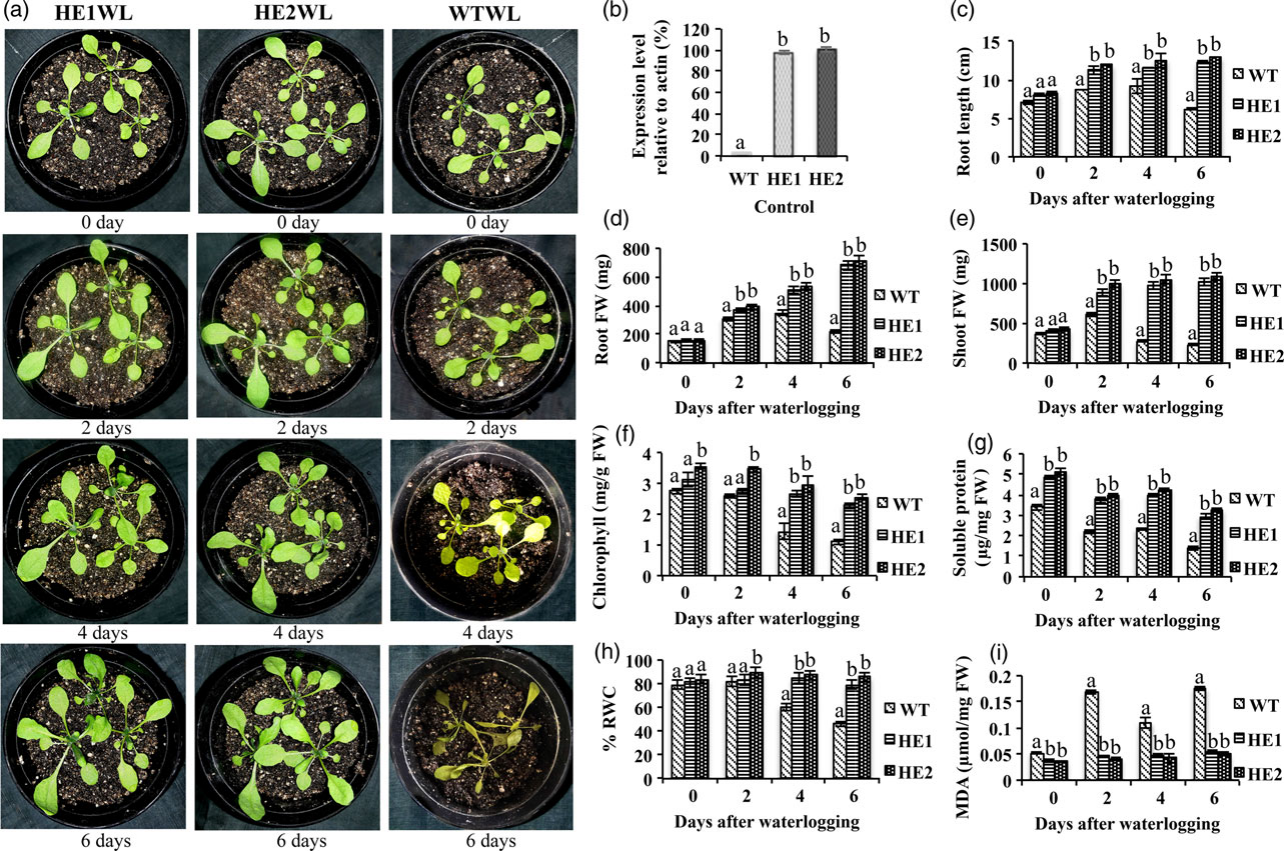
HE lines displayed better morphological and physiological properties under 2, 4 and 6 days of waterlogging stress. (a) HE1 and HE2 showed enhanced waterlogging tolerance than WT. (b) Expression of MaRAP2‐4 in HE lines relative to actin under control condition. (c) Root length (in cm) of HE1 and HE2 was more than WT. (d) Root FW (in mg) was more in HE1 lines. (e) Shoot FW (in mg) was more in HE lines. (f) Chlorophyll content (μg/mg FW) showed relatively more decrease in WT. (g) Soluble protein content (μg/mg FW) was more in HE lines. (h) % RWC declined in WT, while HE1 and HE2 maintained a constant level. (i) MDA content (μmol/mg FW) was more in WT than HE1 and HE2. Represented values were means of 15 independent measurements on a different plant. The error bars show mean ± SD. The letters above column indicates a statistically significant difference for the data of WT plants compared to those of HE lines at different time intervals of waterlogging stress. Different letters indicate a significant difference between columns (*P* < 0.05), while same letters indicate no significant difference.

As *MaRAP2‐4* was highly induced in response to waterlogging treatment, and it specifically interacted with anoxia‐responsive JARE *cis*‐element, so we wanted to know whether MaRAP2‐4 has any role in waterlogging tolerance. There are few reports of transformation and regeneration of Mentha, but the process is very tedious and time consuming. Therefore transgenic *Arabidopsis* lines HE1, HE2 and WT were given waterlogging treatment for 6 consecutive days. We observed that HE1 and HE2 were showing better response than WT after 6 days of treatment (Figure 3a). We also observed the MaRAP2‐4 expression relative to actin in transgenic lines and found that the nature of the phenotype was related to expression level (Figure 3b). WT leaves showed phenotypes of waterlogging‐induced senescence and chlorosis after 6 days of treatment in contrast to HE1 and HE2. Root length of HE1 and HE2 was more than WT, which indicates that HE lines provide better adaptation and tolerance towards waterlogging stress and help to maintain the morphology of plants to cope up with the situation (Figure 3c). Similarly a significant reduction of root and shoot FW of WT in comparison to HE1 and HE2 was observed under waterlogging treatment (Figure 3d, e). Stress affects protein metabolism and chlorophyll content (Ahsan *et al*., 2007). In correlation, we found a gradual decrease in protein and chlorophyll content in all lines, but relative decrease was more in WT (Figure 3f, g). Sufficient water content is a physiological basic need required to stabilize subcellular structures and facilitate recovery of cell from stress (Leul and Zhou, 1999). HE1 and HE2 were showing more % RWC than WT after 6 days of stress (Figure 3h). During stress, lipid peroxidation caused due to decomposition of polyunsaturated fatty acid hydroperoxides results in formation of MDA. So when we measured lipid peroxidation‐induced oxidative damage in all lines, it was observed that MDA content was significantly high in WT in comparison to HE1 and HE2 (Figure 3i). This suggests that MaRAP2‐4 leads to waterlogging tolerance by probably regulating morphological/biochemical features and MDA‐mediated oxidative damage.

### MaRAP2‐4 regulated oxidative stress response by modulating ROS scavenging

**Figure 4:**
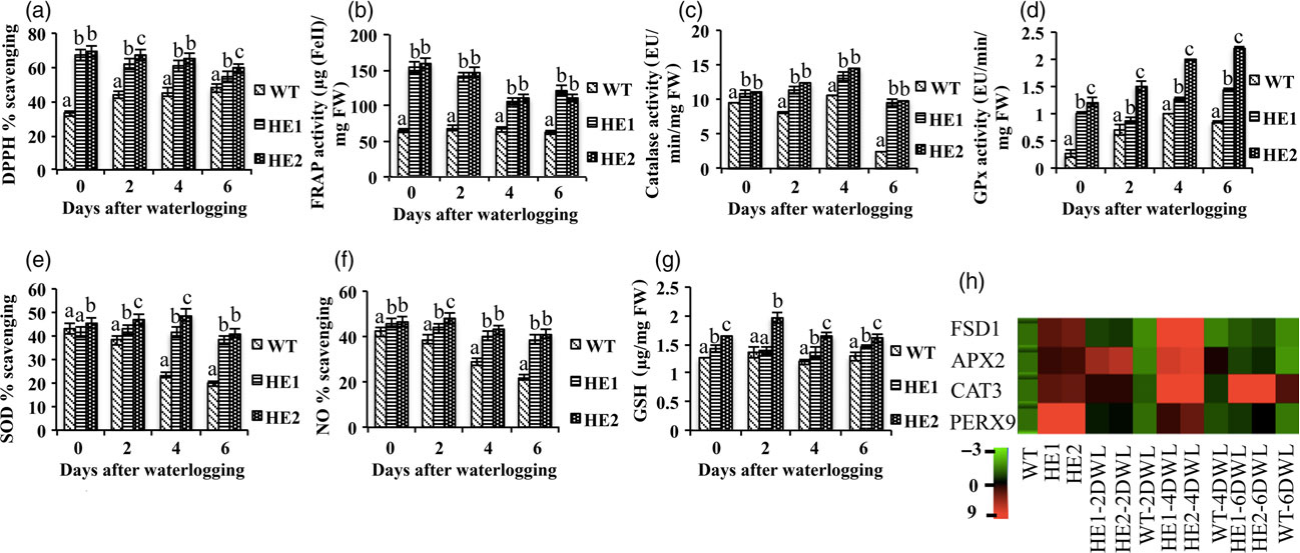
HE lines showed enhanced antioxidant property after 2, 4 and 6 days of waterlogging stress. (a) DPPH % scavenging showed higher antioxidant activity in HE1 and HE2 than WT. (b) FRAP activity (μg(FeII)/mg FW) was more in HE lines. (c) Catalase activity (μmol/min/mg FW) was more in HE lines. (d) GPx activity (μmol/min/mg FW) was more in HE1 and HE2 than WT. (e) SOD % scavenging was more in HE lines. (f) NO % scavenging showed relatively more decrease in WT. (g) GSH content (μg/mg FW) was also more in HE1 and HE2 than WT. (h) Relative expression of antioxidant‐responsive genes was more in HE1 and HE2 than WT under both control and waterlogging treatment. Represented values were means of 15 independent measurements on a different plant. The error bars show mean ± SD. The letters above column indicates a statistically significant difference for the data of WT plants compared to those of HE lines at different time intervals of waterlogging stress. Different letters indicate a significant difference between columns (*P* < 0.05), while same letters indicate no significant difference.

Waterlogging disrupts cellular homoeostasis resulting in enhanced ROS production. Antioxidant enzymes and nonenzymatic free radical scavengers make a defensive barrier against waterlogging‐induced oxidative stress. We analysed the antioxidant property of both the lines under waterlogging stress. We measured DPPH % scavenging and FRAP assay, which revealed that total antioxidant activity was more in HE1 and HE2 in comparison to WT (Figure 4a, b). Catalase activity, GPx activity and SOD % scavenging were more in HE1 and HE2 (Figure 4c, d, e). Despite the diverse beneficial physiological functions of NO, it contributes to oxidative stress by reacting with superoxide to form peroxynitrite anion, which decomposes to produce NO^−^ and OH^−^ (Patel and Patel, 2011). NO scavenging was measured and despite the decrease in both the lines, the decrease was more in WT (Figure 4f). Additionally we quantified the level of nonenzymatic radical scavenger GSH (total thiol content) and observed more accumulation in HE1 and HE2 (Figure 4g). To verify these results, we observed the relative expression of genes involved in ROS regulation under 2, 4 and 6 days of waterlogging treatment. Peroxidase9 (Perx9—removes H_2_O_2_ and oxidizes toxic reductants), catalase3 (Cat3—catalyses H_2_O_2_), ascorbate peroxidase2 (APX2—removes H_2_O_2_) and superoxide dismutase [Fe]1 (FSD1—removes superoxide anion radicals) showed higher expression in HE1 and HE2 under control and waterlogged condition in comparison to WT (Figure 4h). So these results highlight that HE1 and HE2 are able to counteract the ROS production better than WT under waterlogging stress by regulating endogenous antioxidant system and genes associated with it.

### MaRAP2‐4 influenced several metabolic pathways

**Figure 5:**
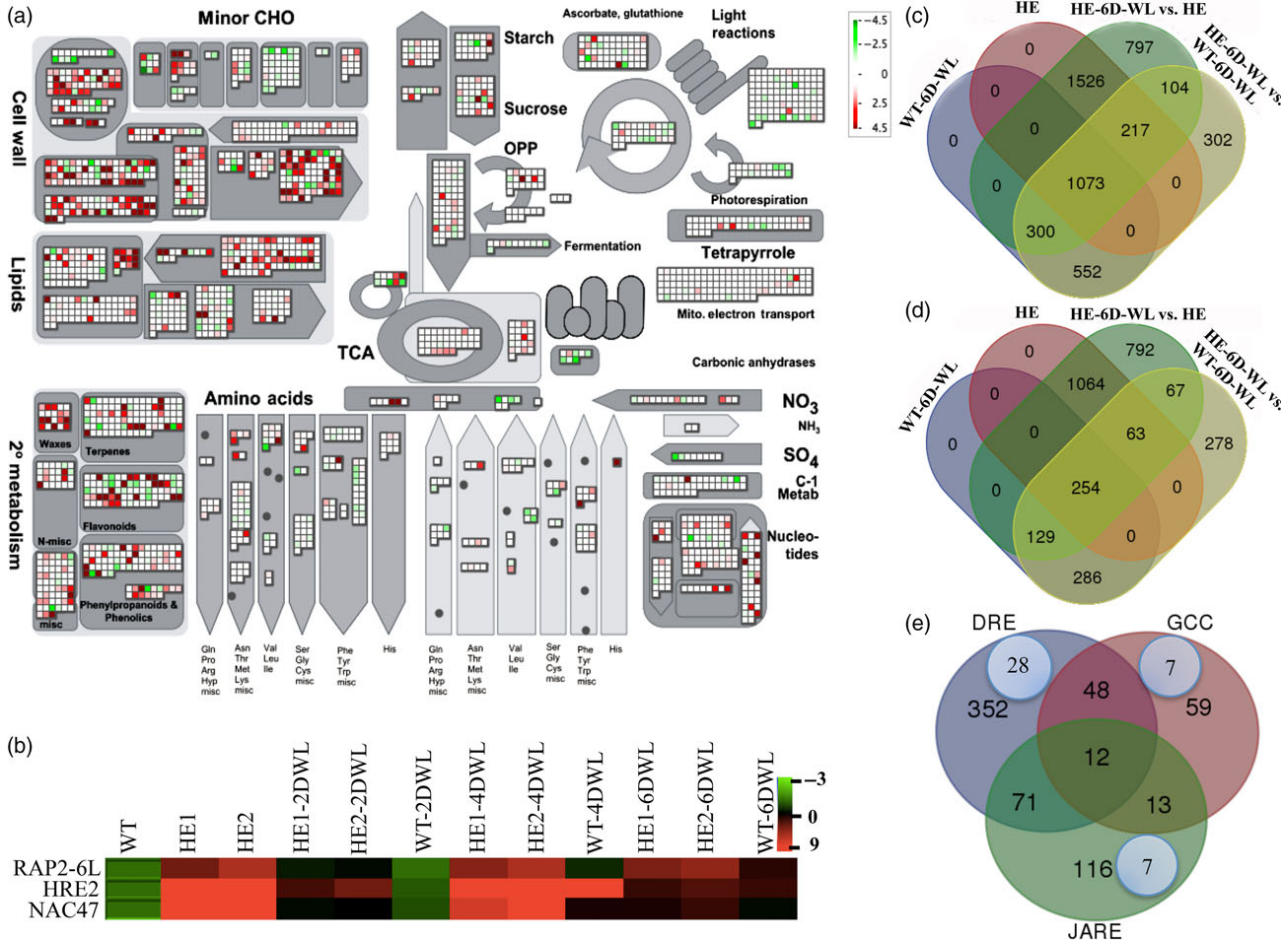
Microarray and promoter analysis in HE lines. (a) Normalized log2 expression values for up‐ and down‐regulated genes are plotted onto boxes corresponding to their putative functional annotation using MapMan 3.6. Colour intensity reflects the extent of increase in expression, with more intense colour (red—up‐regulation, green—down‐regulation) means more up‐ or down‐regulation. (b) Induced expression of hypoxia/waterlogging‐responsive genes (also induced in microarray data) was more in HE1 and HE2 than WT under control and waterlogged condition. (c) Venn diagram showing the distribution of up‐regulated genes (*P* < 0.05) in MaRAP2‐4HE and WT under control as well as waterlogged condition. (d) Venn diagram showing the distribution of down‐regulated genes (*P* < 0.05) in MaRAP2‐4HE and WT under control as well as waterlogged condition. (e) Venn diagram showing the distribution of DRE, JARE and GCC motifs in the promoters of up‐regulated genes of MaRAP2‐4HE under control condition. The blue circles within the Venn diagram highlight abundance of sugar metabolism/transport responsive genes.

Microarray analysis of both HE lines and WT was performed under untreated and after 6 days of waterlogging treatment. Common transcripts in both the lines were screened, and fold induction values were normalized for a single fold induction. Microarray analysis of MaRAP2‐4HE (test) vs WT (control) under untreated condition revealed extensive changes in expression of different transcripts, with 1768 up‐regulated (>twofold) and 382 down‐regulated (<‐twofold) having *P*‐value <0.05 (Figure 5c, d, Data S3, Figure S6a). When we compared data between MaRAP2‐4HE‐6D‐WL (test) vs MaRAP2‐4HE (control), we found 655 up‐regulated (>twofold) and 256 down‐regulated (<‐twofold) transcripts with *P* < 0.05 (Figure 5c, d, Data S4, Figure S6b). Also comparison between MaRAP2‐4HE‐6D‐WL (test) vs WT‐6D‐WL (control) showed 251 up‐regulated (>twofold) and 90 down‐regulated (<‐twofold) transcripts with *P* < 0.05 (Figure 5c, d, Data S5, Figure S6c) suggesting huge transcriptional alteration involving wide interconnected network of regulatory and metabolic processes. The up‐ and down‐regulated genes were classified into functional and pathway categories by DAVID database. Carbohydrate biosynthetic process (GO: 0016051) and carbohydrate metabolic process (GO: 0005975) are significantly affected along with stress response pathways (GO: 0006950) (Data S6). This reflects that waterlogging leads to disruption of normal cellular metabolism and modulate various pathways to deal with the adversities associated with it. To verify these results, we observed the expression of three TFs from our microarray data already reported to be involved in waterlogging/hypoxia response. Two ERFs (waterlogging‐responsive *RAP2‐6L* and hypoxia‐responsive *HRE2*) and a NAC (waterlogging‐responsive *NAC47/SHYG*) TF were showing similar expression pattern as observed in microarray data (Figure 5b).

Global transcript profiling as represented and analysed via MapMan 3.6 in untreated condition showed altered expression of wide array of transcripts. Genes involved in abiotic stress (bin 20.2, *P*‐value = 0.003) rather specifically in drought/salt stress (bin 20.2.3, *P*‐value = 3.89E‐^04^) are significantly affected probably because of interaction with DRE *cis‐*elements. Ethylene‐mediated regulation (bin 17.5.3, *P*‐value = 0.02) is also affected. MaRAP2‐4 modulates antioxidant enzyme activity so redox regulation by dismutases and catalases (bin 21.6, *P*‐value = 0.02), thioredoxin (bin 21.1, *P*‐value = 0.005) and glutaredoxins (bin 21.4, *P*‐value = 0.006) are also affected (Figures 5a, S7, Data S7).

### MaRAP2‐4 interacting *cis*‐elements were present in promoter of carbohydrate metabolism/transport responsive genes

Waterlogging affects carbohydrate metabolism, so minor CHO metabolism (bin 3.5, *P*‐value = 1.14E^−05^), signalling in sugar and nutrient physiology (bin 30.1, *P*‐value = 0.04) and TCA cycle (bin 8.1.4, *P*‐value = 0.04) are significantly influenced. Within the up‐regulated set of genes, we examined the promoters for the presence of interacting *cis‐*elements from Figure 2a (DREs, JARE and GCC). There were 483, 212 and 132 genes whose promoter carry single or multiple copies of DRE/s, JARE/s or GCC/s, respectively. Also promoters of 71 genes carry both DRE and JARE, 48 promoters carry both DRE and GCC, while 13 promoters carry both JARE and GCC. All three *cis‐*elements were present in promoters of 12 genes (Figure 5e, Data S8). As sugar metabolism was deeply affected, we screened these promoters whose downstream genes are responsive to sugar metabolism and/or transport. In this category, we found 28 promoters with DRE, seven promoters with JARE and seven promoters with GCC *cis‐*elements as shown in Table 1 (Figure 5e). We also observed that MaRAP2‐4 regulates many transcripts involved in glycolysis/gluconeogenesis and starch/sucrose metabolism (Figures S9, S10). These findings suggest that MaRAP2‐4 regulates waterlogging tolerance probably by regulating carbohydrate metabolism/transport through DRE‐, GCC‐ and JARE‐mediated cascade.

**Table 1:** Up‐regulated carbohydrate metabolism/transport‐related genes carrying MaRAP2‐4 interacting *cis*‐elements

TAIR ID	Name	*cis*‐elements	Fold induction	*P*‐value
AT1G34580	Sugar transport protein 5	3 DRE	7.00	0.0287
AT4G15210	Beta‐amylase 5	2 DRE	6.86	0.0079
AT4G35670	Pectin lyase‐like	1 DRE	4.76	0.0488
AT2G05790	O‐glycosyl hydrolases	1 DRE	4.70	0.0142
AT2G36190	Beta‐fructofuranosidase	1 DRE	4.19	0.0267
AT5G65730	Xyloglucan endotransglucosylase/hydrolase	1 DRE	4.11	0.0085
AT4G37800	Xyloglucan endotransglucosylase/hydrolase	2 DRE	4.11	0.0409
AT5G04160	Nucleotide‐sugar transporter	1 DRE	3.65	0.0192
AT2G39060	Bidirectional sugar transporter SWEET9	1 DRE	3.43	0.0195
AT1G52400	Beta‐glucosidase 18	1 DRE	3.16	0.0171
AT3G53080	D‐galactoside/L‐rhamnose binding SUEL lectin	1 DRE	3.14	0.0334
AT1G09350	Galactinol synthase 3	4 DRE	2.98	0.0214
AT5G49215	Pectin lyase‐like superfamily protein	1 DRE	2.92	0.0348
AT1G21460	Bidirectional sugar transporter SWEET1	1 DRE	2.87	0.0288
AT1G13250	Galacturonosyltransferase‐like 3	4 DRE	2.76	0.0081
AT1G45130	Beta‐galactosidase 5	1 DRE	2.75	0.0185
AT2G38060	Phosphate transporter 4;2,	3 DRE	2.71	0.0375
AT3G05400	Sugar transporter ERD6‐like 12	2 DRE	2.68	0.0214
AT5G42720	Glycosyl hydrolase family 17 protein	1 DRE	2.24	0.0454
AT4G15530	Pyruvate, phosphate dikinase 1	1 JARE	2.70	0.0321
AT1G10550	Xyloglucan endotransglucosylase/hydrolase 33	1 JARE	2.45	0.0337
AT1G16390	Organic cation/carnitine transporter 3	1 JARE	9.35	0.0286
AT1G77210	Sugar transport protein 14	1 GCC	3.34	0.0312
AT3G26140	Cellulase (glycosyl hydrolase family 5)	1 GCC	8.01	0.0008
AT3G57510	Polygalacturonase ADPG1	1 DRE, 1 JARE	3.62	0.0219
AT2G22900	Glycosyltransferase 7	4 DRE, 1 JARE	2.67	0.0036
AT5G39320	UDP‐glucose 6‐dehydrogenase 2	1 DRE, 2 GCC	2.60	0.0024
AT5G11230	Sugar phosphate/phosphate translocator	1 DRE, 1 GCC	2.39	0.0293
AT1G55740	Raffinose synthase 1	1 DRE, 1 GCC	2.85	0.0424
AT5G50790	Bidirectional sugar transporter SWEET10	3 DRE, 1 GCC	9.30	0.0010
AT1G10640	Pectin lyase‐like protein	2 DRE, 1 GCC	7.91	0.0153
AT3G61490	Polygalacturonase‐like protein	1 DRE, 1 GCC	3.85	0.0149
AT3G12700	Aspartyl protease family protein	3 DRE, 1 GCC	2.76	0.0254
AT3G52340	Sucrose‐phosphatase 2	1 JARE, GCC	2.95	0.0216
AT1G02460	Pectin lyase‐like superfamily protein	1 DRE, 1 JARE, 1 GCC	3.28	0.0000

### MaRAP2‐4 interacted with *AtSWEET10* promoter to affect carbohydrate content

**Figure 6:**
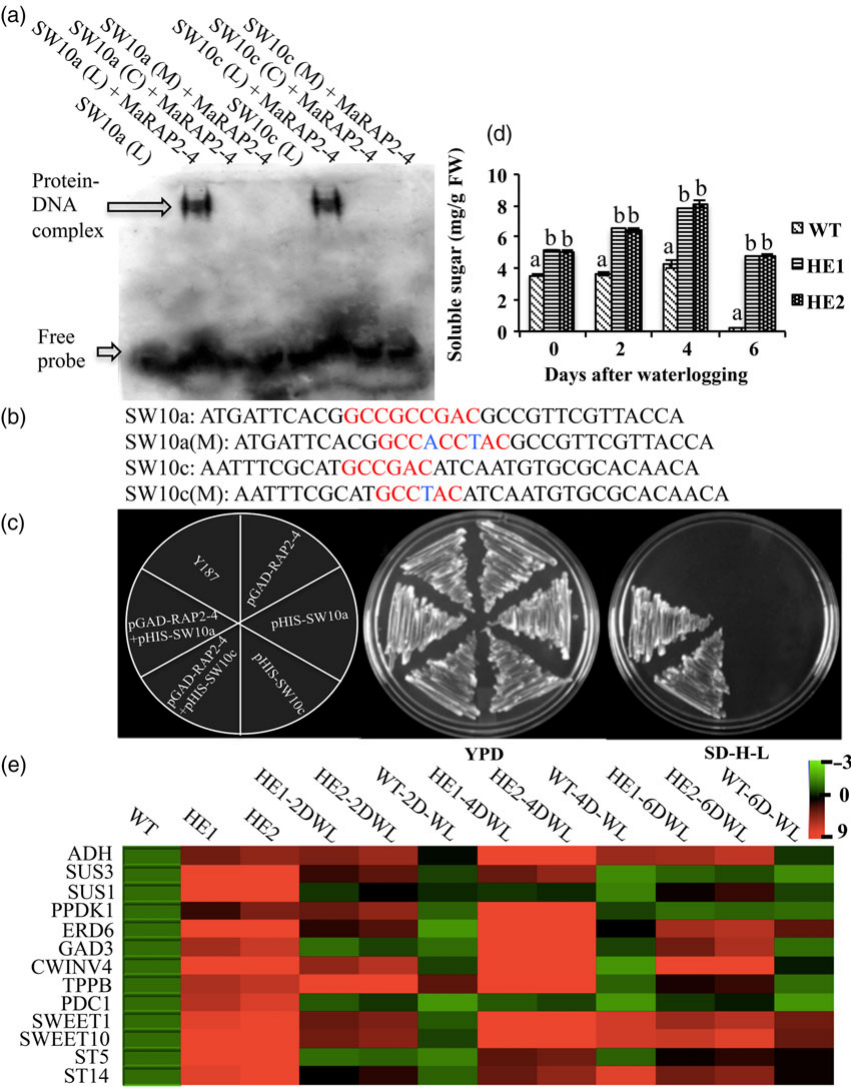
MaRAP2‐4 interacts with *AtSWEET10* to modulate carbohydrate level. (a) MaRAP2‐4 specifically interacts at two positions of *AtSWEET10* promoter in EMSA. (b) Promoter sequences used as probes. Red colour indicates *cis*‐element, and blue colour indicates substitution in *cis*‐element. (c) *In vivo* interaction of MaRAP2‐4 with both the promoter fragments carrying the *cis*‐elements in yeast Y187. (d) Soluble sugars content (mg/g FW) was more in HE1 and HE2 than WT. (e) Induced expression of sugar metabolism/transport responsive genes was observed in HE lines in comparison to WT under both control and waterlogged condition. Soluble sugars content is the mean of 15 independent measurements on a different plant. The error bars show mean ± SD. The letters above column indicates a statistically significant difference for the data of WT plants compared to those of HE lines at different time intervals of waterlogging stress. Different letters indicate a significant difference between columns (*P* < 0.05), while same letters indicate no significant difference.

In microarray data among sugar responsive genes, *AtSWEET10* was showing highest expression value of 9.3 with *P*‐value 0.001 (Table 1). Also its promoter contains three DRE and one GCC *cis‐*elements (Figure S8). To study whether MaRAP2‐4 interacts with *AtSWEET10* promoter, we designed specific probes from these promoter regions and performed EMSA (Figure 6c). MaRAP2‐4 showed specific interaction at two positions in the *AtSWEET10* promoter, first one carrying an overlap of DRE and GCC and second one carrying only DRE (Figure 6a). To further verify these interactions *in vivo*, we separately cloned the promoter fragments carrying the interacting *cis*‐elements in pHIS2.0 vector. When *MaRAP2‐4* cloned in pGADT7 was cotransformed with these promoter fragments in yeast Y187, we observed positive Y1H interaction within them (Figure 6d). Then we quantified total soluble sugars content in HE1, HE2 and WT under control and waterlogging condition. Soluble sugars content was found to be more in HE1 and HE2 lines (Figure 6b). Further we screened highly expressed sugar metabolism/transport responsive genes from microarray data and observed their expression in WT, HE1 and HE2 under control and waterlogged condition. *ADH* (involved in anaerobic respiration and required for survival/acclimation in hypoxic conditions, especially in roots), *SUS1/3* (cleaves sucrose to yield UDP‐glucose and fructose), *PPDK1* (forms phosphoenolpyruvate and regulates flux of carbon into starch and fatty acids), *GAD3* (forms GABA that plays a major role in carbon and nitrogen metabolism), *CWINV4* (forms beta‐D‐fructofuranosides), *TPPB* (Removes phosphate from trehalose 6‐phosphate to produce free trehalose involved in abiotic stress response) and *PDC1* (plays major role in ethanolic fermentation during anoxia) showed induced expression in HE1 and HE2 under control as well as waterlogged condition. Similarly sugar transport‐related genes like *SWEET1/10*,* STP5/14* and *ERD6* were showing induced expression (Figure 6e). These results indicate that MaRAP2‐4 regulates sugar metabolism/transport by interacting with *AtSWEET10* promoter and probably with other sugar responsive genes to provide abiotic stress tolerance in *Arabidopsis*.

### MaRAP2‐4 imparted drought and salt tolerance

**Figure 7:**
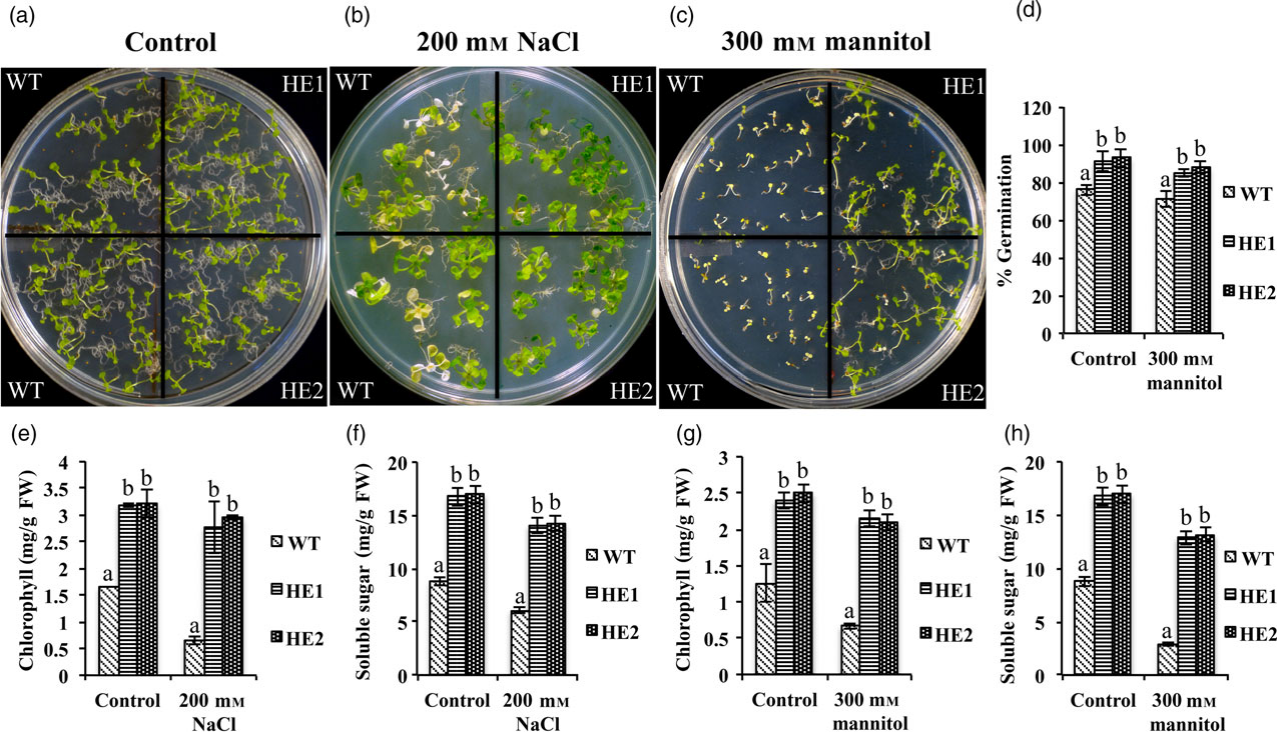
MaRAP2‐4 leads to enhanced drought and salt tolerance. (a) Growth of HE1, HE2 and WT on " MS media. (b) In 200 mm NaCl, WT seedlings showed more senescence and chlorosis than HE lines. (c) In 300 mm mannitol, WT seedlings died just after germination, while HE lines were able to grow. (d) % Germination was also more in HE1 and HE2 than WT under dehydration stress. (e) Chlorophyll content (mg/g FW) was more in HE1 and HE2 than WT under salt stress. (f) Soluble sugars content (mg/g FW) was more in HE1 and HE2 than WT under salt stress. (g) Chlorophyll content (mg/g FW) was more in HE lines under dehydration stress. (h) Soluble sugars content (mg/g FW) was more in HE lines under dehydration stress. Chlorophyll content, sugar content and % germination were calculated based on the mean of three independent experiment performed. The error bars show mean ± SD. The letters above column indicates a statistically significant difference for the data of WT plants compared to those of HE lines under control and stress conditions. Different letters indicate a significant difference between columns (*P* < 0.05), while same letters indicate no significant difference.

To study whether MaRAP2‐4 has any role in other stress tolerance, we gave drought and salt stress to HE1, HE2 and WT. Under control conditions, both HE lines and WT showed similar phenotype (Figure 7a). When WT seedlings were transferred to 200 mm NaCl media, they showed early senescence and chlorosis while HE1 and HE2 seedlings showed tolerance (Figure 7b). Chlorophyll and total soluble sugars content was also significantly low in WT (Figure 7d). To study drought response when seeds were directly germinated on 300 mm mannitol media, WT seedlings died just after germination, while HE1 and HE2 seedlings were showing tolerance (Figure 7c). Chlorophyll, total soluble sugars content and % germination were more in HE1 and HE2 (Figure 7e, f). These results indicate that MaRAP2‐4 is also a positive regulator of drought and salt stress tolerance.

## Discussion

ERFs are involved in wide range of developmental and stress responses. They regulate downstream signalling of these responses by interacting with various *cis*‐elements (Chen *et al*., 2016; Shukla *et al*., 2006). In many instances, they regulate simultaneous multiple responses. In this report, we have shown that MaRAP2‐4, an ERF from waterlogging‐tolerant *Mentha arvensis,* provides waterlogging tolerance in *Arabidopsis*. Induced expression of *MaRAP2‐4* in response to drought, salt and cold implies that it is multiple stress responsive. MaRAP2‐4 also interacted with two DRE isoforms, which explains the enhanced drought and salt stress tolerance of HE lines. Ethylene‐, MeJA‐ and ABA‐mediated induction of TF are observed in stress response, which suggests the involvement of MaRAP2‐4 in different stresses. It also interacted with GCC box (involved mainly in defence response of PR genes) suggesting the probable involvement of MaRAP2‐4 in biotic stress response. Finally *MaRAP2‐4* was highly induced in waterlogging stress highlighting its role in waterlogging response. Presence of JARE motif most abundantly in the ERF‐induced anoxia genes and specific interaction of MaRAP2‐4 with it suggest that MaRAP2‐4 is involved in waterlogging‐induced anoxia response. Although in this study DRE/GCC‐mediated waterlogging response through *AtSWEET10* is observed, we believe that JARE‐mediated waterlogging response is also operational as many genes were carrying this motif in their promoters.

Anaerobic proteins supports fermentation/glycolysis, prevents enhanced ROS production and maintains membrane integrity under waterlogging stress (Greenway and Gibbs, 2003). So enhanced antioxidant enzyme activity and induced expression of responsive genes correspond with the higher protein content. Higher level of MDA and/or NO puts harmful effect on plants, which is quite evident from the morphological features of WT such as reduced FW and root length. Growth and developmental anomalies in WT are also because of reduced movement of O_2_, CO_2_ and ethylene as well as because of reduced ATP generation (Gibbs and Greenway, 2003).

**Figure 8:**
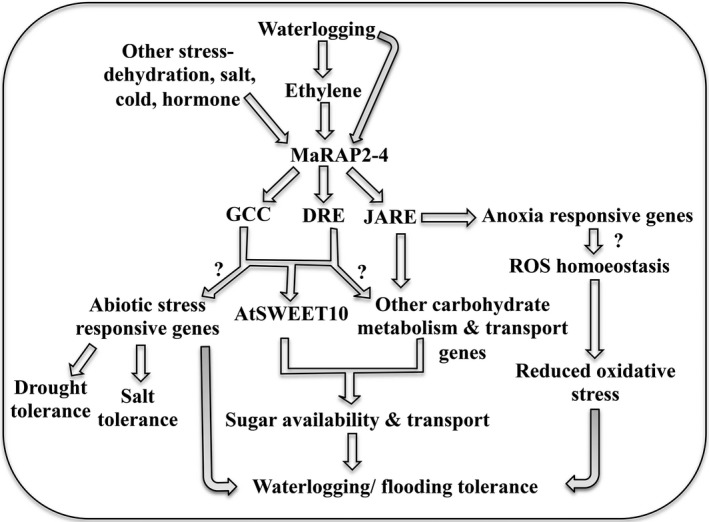
Schematic representation of the mechanism of action of MaRAP2‐4 towards stress response. Waterlogging and ethylene induces expression of *MaRAP2‐4*, which further activates *AtSWEET10*, and probably other sugar metabolism/transport‐related genes through DRE/GCC/JARE cascades. They regulate carbohydrate content that plays an important role in waterlogging tolerance. Higher sugar content generally favours stress tolerance as the energy required to establish cellular homoeostasis is provided by these carbon sources. MaRAP2‐4 also reduces stress‐induced oxidative damage through increasing activity of antioxidant enzymes and genes associated it. Salinity and drought stress also induce MaRAP2‐4 expression and enhance tolerance of plants to these stresses. The question marks indicate direct/indirect regulation of MaRAP2‐4 (not experimentally proved).

The modest ATP generation capability of anaerobic respiration depends on a ready supply of glucose and its precursors. Higher expression of sugar transporters such as AtSWEET10 and higher accumulation of total soluble sugars in HE lines under waterlogging stress are positively correlated. Membrane‐embedded SWEET proteins play an important role in various developmental processes and stress response where sugar efflux is necessary (Feng and Frommer, 2016). They maintain sugar homoeostasis by transporting sugars across cell membranes down a concentration gradient and accumulate them in the sink tissue (Sosso *et al*., 2015; Wei *et al*., 2014). Carbohydrate content is an important aspect of waterlogging rather abiotic stress tolerance (Yang *et al*., 2014). Apart from being a substrate in carbon and energy metabolism, sugars have an essential role in signal transduction. It is reported that pigeon pea genotypes (ICPL84023 and ICP301) that are waterlogging tolerant contain higher total, reducing and nonreducing sugar content than susceptible ICP7035 and Pusa207 genotypes (Kumutha *et al*. 2008). The efficiency with which sugars are transported actually determines photosynthetic productivity by alleviating product inhibition, which ultimately contributes to plant vigour through control of source/sink alliance and biomass distribution (Ayre, 2011). During submergence, some rice varieties such as C9285 rapidly utilize their carbohydrate reserve to escape the hypoxia stress (LOES‐low oxygen escape syndrome), while some other varieties such as FR13A conserve the carbohydrate reserve to endure the hypoxia stress and to regrow afterwards (LOQS‐low oxygen quiescence syndrome). An illustrative pathway showing the probable mechanism of multiple stress tolerance by MaRAP2‐4 is given in Figure 8. The depicted pathway highlights that waterlogging influences ethylene accumulation which induces expression of *MaRAP2‐4*. Then MaRAP2‐4 activates downstream *AtSWEET10* by DRE and GCC boxes, which further influence carbohydrate movement and availability. AtSWEET10 probably has a role in GA uptake when expressed in yeast and oocytes (Kanno *et al*. 2016), and GA crosstalk might help in hypoxia response of plants (Phukan *et al*., 2015). Also http://string-db.org shows that AtSWEET10 can probably interact with other sugar transporters such as AtSWEET8 and SUC2. Therefore, it is an important candidate for MaRAP2‐4 regulated sugar mediated hypoxia response. MaRAP2‐4 also might regulate other sugar metabolism/transport responsive genes as well as anoxia‐responsive genes through JARE or DRE/GCC motifs. All together, these responses contribute to waterlogging tolerance in HE lines. Another cascade involves up‐regulation of MaRAP2‐4 in response to various other abiotic stresses such as drought and salinity as well as hormonal treatments. Induced MaRAP2‐4 then provides drought and salt tolerance through interacting with DRE and GCC *cis*‐elements.

The study concludes that MaRAP2‐4, an ERF TF from *M. arvensis,* activates *AtSWEET10* in *Arabidopsis* that directly or indirectly assist in sugar accumulation in the required tissues. Sugar availability deeply affects HE lines, which might be the reason for enhanced tolerance to waterlogging, drought and salinity. Multiple binding affinities of a single ERF and its crosstalk with a sugar transporter to regulate various responses have not been studied extensively. So MaRAP2‐4 and its homologues could be potential targets for acquiring multiple stress and developmental responses simultaneously.

## Material & method

### Plant material and stress treatment

*Mentha* plants were grown according to Phukan *et al*. (2014). Stress treatments in *Mentha* were given according to Mishra *et al*. (2015). For *Arabidopsis thaliana* Col‐0, seeds were given stratification treatment for 4 days and then transferred to a climate‐controlled chamber with 16‐h light period with 120–150 μmol/m^2^s light intensity, 60% relative humidity and 23 °C temperature. For full‐length cloning of *MaRAP2‐4*, Rapid Amplification of cDNA Ends (RACE) was performed according to Mishra *et al*. (2015). List of all primers used in this study is given in Data S1. For generation of *Arabidopsis* transgenic lines, *MaRAP2‐4* was cloned in pBI121 vector under constitutive expression of CaMV‐35S promoter. The construct was transformed in GV3101 *Agrobacterium tumefaciens*. The positive colonies were used to transform *Arabidopsis* plants (primary bolt clipped, secondary bolt 5–10 cm long, few open flowers and many closed buds) by floral‐dip method (Clough and Bent 1998). The seeds were selected on 50 μg/mL Kanamycin supplemented " MS media for three generations. NPT2‐Kan^(R)^ primers were used for genomic DNA PCR confirmation of integration (Figure S5). Ten independent lines were generated, and further experiments were performed with two lines (HE1 and HE2—HE for heterologous expression). For *Arabidopsis* waterlogging treatment, plants were put inside beakers and waterlogged up to the soil‐atmosphere interface. For salt stress in HE1, HE2 and WT, seeds were grown for 1 week on " MS media and were transferred to media supplemented with 200 mm NaCl for 10 days. For dehydration treatment HE1, HE2 and WT seeds were geminated on " MS media supplemented with 300 mm mannitol and grown for 2 weeks. The percentage germination was calculated based on three independent experiment performed. For physiological, morphological and qRT‐PCR analysis, whole plant samples were collected at an interval of 2, 4 and 6 days of treatment. For morphological and physiological (biochemical and antioxidant properties) analysis, five independent replicated pots of both lines (three plants/pot) were used. Represented values were means of 15 independent measurements on a different plant. For control, plants were put inside beakers without waterlogging. For microarray analysis, WT and both lines of MaRAP2‐4HE (control and waterlogged) were used. Fold change values of both independent microarrays along with their normalized fold value and adjusted *P*‐value are given in Data S3.

### EMSA

For *in vitro* protein–DNA interaction, *MaRAP2‐4* was cloned in pGEX‐4T2 vector in fusion with GST. MaRAP2‐4‐GST fusion protein was induced with 0.3 mM IPTG and purified with GST‐Sepharose beads (Amersham) from *E. coli* BL21‐CodonPlus (DE3) strain (Figure S2). EMSA was performed by interacting MaRAP2‐4‐GST with DIG‐labelled probes using DIG Gel Shift Kit 2nd Generation (Roche) according to manufacturer's protocol.

### Yeast One‐Hybrid Assay (Y1H)

For *in vivo* Y1H assay, *MaRAP2‐4* was cloned in pGADT7 vector, and promoter fragments were cloned in pHIS‐2.0 vector. Both the fragments were cotransformed in *Sacchromyces cerevisiae* Y187 strain. The positive interaction was screened by plating them on SD‐His‐Leu media. All yeast experiments were performed according to Yeast Protocols Handbook Clontech.

### Subcellular localization

For subcellular localization, *MaRAP2‐4* was cloned in pBI121‐CFP (CFP inserted in place of GUS) under constitutive expression of CaMV‐35S promoter. The construct was transformed in *Agrobacterium tumefaciens* GV3101. The positive colonies were used to infiltrate *N. benthamiana* leaves by infiltration media (10 mm MES, pH5.7, 10 mm MgCl2, 150 μm acetosyringone), and samples were collected after 24 h. CFP fluorescence was excited using a 405‐nm UV Laser and observed using an emission bandwidth of 450–505 nm in Leica SP5x confocal microscope (Leica Microsystems). CYTRAK Orange was used that preferentially stains the nucleus (excitation 534, emission 615).

### Transactivation‐β‐galactosidase assay and transactivation domain mapping

For transactivation assay, *MaRAP2‐4* was cloned in pGBKT7 vector in fusion with GAL4‐DBD and transformed in yeast Y187. Transformed colonies were screened on SD‐Ura‐Trp media. For positive transactivation property, the colonies were screened for blue colour with β‐galactosidase colony lift assay using X‐gal. For mapping transactivation domain, nine subsequent truncations (named as ∆Cʽnʼ, n is the number of amino acids truncated) from 3′ end were made and cloned in pGBKT7. Additionally three internal deletions (named as ∆CI1/2/3) were made and cloned in pGBKT7. All these constructs were screened for β‐galactosidase activity to map the probable transactivation domain.

### Quantification of biochemical properties

Quantification of chlorophyll content was performed as described by Vernon (1960), by taking absorbance at 663 and 645 nm. Relative water content (RWC) was determined as described by Yamasaki and Dillenburg (1999) from leaves of treated and control plants. Quantification of protein was performed by Bradford method using Bradford reagent (CBB G250‐ Sigma‐Aldrich) by taking absorbance at 595 nm (Bradford, 1976). Lipid peroxidation was analysed by the thiobarbituric acid test, which determines MDA content (Hodges *et al*., 1999). Carbohydrate quantification was performed by measuring absorbance at 490 nm according to the method by Chow and Landhäusser, 2004; Qi *et al*., 2012. Detailed protocol is given in Data S9.

### Quantification of antioxidant properties

To study antioxidant properties, whole plant samples (0.4–0.8 g) were homogenized in ice‐cold extraction buffer (pH 7.5) containing 50 mm HEPES, 0.4 mm EDTA, 5 mm MgCl_2_, 10% glycerol, 1% PVP, 2 mm DTT and 1 mm PMSF (Gegenheimer, 1990). The homogenate was centrifuged (14 000 ***g***) at 4 °C for 20 min. The supernatant was assayed for antioxidant activity. Detailed protocols are given in Data S9. In brief, Catalase activity was assayed by taking OD at 610 nm after 0, 30, 60 and 90 s as described by Sinha (1972). Its activity was expressed in terms of μmol of H_2_O_2_ consumed/min/mg fresh weight (FW). Similarly for GPx (glutathione peroxidase) activity, OD was taken at 412 nm, and the enzyme activity was expressed in terms of μmol of glutathione utilized/min/mg FW (Rotruck *et al*., 1973). For DPPH (2,2‐diphenyl‐1‐picrylhydrazyl) radical scavenging activity, the reduction capability of DPPH radical was determined by the decrease in its absorbance at 517 nm (Patel and Patel, 2011). Total antioxidant activity by FRAP (ferric ion reducing antioxidant power) assay was carried out according to Benzie and Strain (1996). Absorbance was read at 593 nm, and results are expressed in μgFe(II)/mg FW and compared with that of ascorbic acid. SOD (superoxide dismutase) % scavenging was assayed according to Das *et al*. (2000) by taking absorbance at 560 nm. For NO scavenging activity, the absorbance of chromophore formed was measured at 546 nm, and percent scavenging was calculated (Patel and Patel, 2011). For Estimation of GSH (glutathione reduced), Boyne and Ellman (1972) method was applied. Absorbance was read at 412 nm within 2 min, and GSH concentration was expressed as μg/mg FW. Estimation of GSH is actually estimation of total thiol content but as glutathione is the most abundant reduced sulphur compound (with several functions in stress response); we showed it as the total glutathione content (Herschbach *et al*., 2005).

All spectrophotometric measurements were performed in Eppendorf BioPhotometer D30.

### Microarray preparation and analysis

Total RNA from whole plants (WT, HE1 and HE2 lines) was extracted by RNeasy^(R)^ Plant Mini Kit (Qiagen) under control and 6 days of waterlogging treatment following manufacturer's protocols. RNA was processed and labelled with Cy3‐CTP and hybridized with *Arabidopsis*_GXP_4x44K AMADID slide. Microarray was processed and scanned at 535 nm; images were analysed by Agilent Feature Extraction software (v10.7) to quantify signal and background intensity. The microarray images were with very low background noise and overall clean showing uniform intensity. For normalization and statistical significance, GeneSpring GX 12.6 software was used. Fold induction values from both independent lines were normalized for a single fold induction. *P*‐values for up‐ and down‐regulation were corrected for biological and experimental replicates using Student's *t*‐test. For up‐ and down‐regulation of genes, *P*‐value cut‐off was set <0.05 (Data S3). The microarray data have been processed for deposition in Gene Expression Omnibus with GEO accession number GSE64070. Microarray expression data were visualized using the MapMan 3.6 software (Thimm *et al*., 2004), and functional bins were identified as being significantly responsive to waterlogging stress. Bins with their names and *P*‐values corrected with Wilcoxon rank sum test are given in Data S7. Detailed protocol is given in Data S9.

### Expression analysis of stress inducible genes

RNA was isolated by RNeasy^(R)^ Plant Mini Kit (Qiagen), and cDNA was synthesized using High Capacity cDNA Reverse Transcription kit (Applied Biosystems). Relative expression of transcripts was performed by qRT‐PCR using the SYBR Green PCR master mix kit (Applied Biosystems) in Applied Biosystems 7900 HT Fast Real‐Time PCR System. qRT‐PCR reaction condition consisted of initial step (50 °C for 2 min, 95 °C for 10 min), 40 cycles (95 °C for 15 s, 60 °C for 1 min) and denaturing step (95 °C for 15 s, 60 °C for 15 s, 95 °C for 15 s, 37 °C for 2.5 min). *MaRAP2‐4* expression in HE lines, normalized to actin gene was calculated by 2^−(C^_T_^*MaRAP2‐4*‐C^_T_^*Actin*)^ × 100, and result was represented as expression level relative to Actin (%). Expression analysis of target genes under different treatments, and HE lines were performed in comparison to the WT or experimental control and was calculated by 2^−(C^_T_^treated/HE‐C^_T_^control/WT)^ for which actin was used as an internal control.

### Statistical analysis

The experiments in this study were repeated as stated above, and data shown are means ± SDs. One‐way analysis of variance (ANOVA) was used to assess the effect of waterlogging on morphological features and biochemical parameters, using StatPlus 6.0 software. Post hoc Tukey HSD (honestly significant difference) test was performed to measure the degree of significance using online tool http://astatsa.com/OneWay_Anova_with_TukeyHSD. Different letters indicate a significant difference between columns (*P* < 0.05), while same letters indicate no significant difference.

## Conflict of interest

The authors declare no conflict of interest.

## Supporting information


**Figure S1** Nucleotide and amino acid alignment of MaRAP2‐4.
**Figure S2** Protein induction and purification of MaRAP2‐4.
**Figure S3** Sequences of probes used for EMSA.
**Figure S4** Transactivation assay of MaRAP2‐4.
**Figure S5** Genomic DNA PCR confirmation of MaRAP2‐4 integration.
**Figure S6** 2D scatter plot of differentially regulated genes in microarray data.
**Figure S7** Mapman representation of differentially regulated genes in microarray data.
**Figure S8** AtSWEET10 promoter sequence.
**Figure S9** Up‐ and down‐regulated genes in Glycolysis/gluconeogenesis.
**Figure S10** Up‐ and down‐regulated genes in Starch and sucrose metabolism.
**Data S1** List of primers.
**Data S2** List of DEGs from SSH analysis.
**Data S3** List of up‐ and down‐regulated genes (HE vs WT).
**Data S4** List of up‐ and down‐regulated genes (HE‐6D‐WL vs HE).
**Data S5** List of up‐ and down‐regulated genes (HE‐6D‐WL vs WT‐6D‐WL).
**Data S6** List of up‐ and down‐regulated genes in functional and pathway categories in HE vs WT data under control condition.
**Data S7** List of Bins with their names and normalized p‐value used in Mapman.
**Data S8** List of up‐regulated genes with MaRAP2‐4 interacting *cis* –elements.
**Data S9** Detailed material and method.
