# Supplementary material for: MaRAP2‐4, a waterlogging‐responsive ERF from Mentha, regulates bidirectional sugar transporter AtSWEET10 to modulate stress response in Arabidopsis
Source: Plant Biotechnol J. 2017 Jul 25;16(1):221–33. doi: 10.1111/pbi.12762 (PMC5785340; doi:10.1111/pbi.12762)
Supplement: Supplementary file 1 — Figure S1 Nucleotide and amino acid alignment of MaRAP2‐4. Figure S2 Protein induction and purification of MaRAP2‐4. Figure S3 Sequences of probes used for EMSA. Figure S4 Transactivation assay of MaRAP2‐4. Figure S5 Genomic DNA PCR confirmation of MaRAP2‐4 integration. Figure S6 2D scatter plot of differentially regulated genes in microarray data. Figure S7 Mapman representation of differentially regulated genes in microarray data. Figure S8 AtSWEET10 promoter sequence. Figure S9 Up‐ and down‐regulated genes in Glycolysis/gluconeogenesis. Figure S10 Up‐ and down‐regulated genes in Starch and sucrose metabolism. Data S1 List of primers. Data S2 List of DEGs from SSH analysis. Data S3 List of up‐ and down‐regulated genes (HE vs WT). Data S4 List of up‐ and down‐regulated genes (HE‐6D‐WL vs HE). Data S5 List of up‐ and down‐regulated genes (HE‐6D‐WL vs WT‐6D‐WL). Data S6 List of up‐ and down‐regulated genes in functional and pathway categories in HE vs WT data under control condition. Data S7 List of Bins with their names and normalized p‐value used in Mapman. Data S8 List of up‐regulated genes with MaRAP2‐4 interacting cis –elements. Data S9 Detailed material and method. [file PBI-16-221-s001.pdf]

Supplementary figure 1

```
ctaactcgactcgatctgatgctcagacactgagagttattgtgcttggaatcacttacg
ctttaacgttacttatgtagaacagtcgattagctttttctcatcttaagttattatata
catcatcttacgtactcgatttcgaagtagaatggcagcagctacaaagattatgtagca
gcactttagtagtagtttaatgtgatctttatatatacagatcatttagagagagttgat
gcaagcacttgaacttttattcataacagtgcttctacaacttctccttgttcattttct
ttatcttcatcttcatctcctccatcatcgtcatctttctcatcttcttcttctcctttt
tctttatcttcaacttcttcatcttcacagcctgattttggttacccagattttgttca
acatcttctacaggtacccaa
atgtttctcaagggtattttagtaattatgagcaattatcatcagctgttcttgattct
Met F  S  Q  G  Y  F  S  N  Y  E  Q  L  S  S  A  V  L  D  S
tcatctccttcttctttcaatcttaatcatcttaccttagctcagattcaacaacttcaa
 S  S  P  S  S  F  N  L  N  H  L  T  L  A  Q  I  Q  Q  L  Q
caacagattcttcttcaacaacaacagaattcatacgtgaacatgatttcacaacatcaa
 Q  Q  I  L  L  Q  Q  Q  Q  N  S  Y  V  N  M  I  S  Q  H  Q
acaacccagaattcatacctaagtccaaaatcaattccaatgaaagcagtgggtttatca
 T  T  Q  N  S  Y  L  S  P  K  S  I  P  M  K  A  V  G  L  S
tcacagacagccaaacccacaaagctatacaggggagttagacagagacattggggaaaa
 S  Q  T  A  K  P  T  K  L  Y  R  G  V  R  Q  R  H  W  G  K
tgggttgcagagatcagactaccaagaaacaggacaaggctttggttaggtactttgac
 W  V  A  E  I  R  L  P  R  N  R  T  R  L  W  L  G  T  F  D
actgctgaggaagctgccatggcttatgacagagctgcttacaagctaagaggtgatttt
 T  A  E  E  A  A  M  A  Y  D  R  A  A  Y  K  L  R  G  D  F
gccagactcaatgtcccacatctgaagcaccaattatcaagtgctggtgatgggaaattt
 A  R  L  N  V  P  H  L  K  H  Q  L  S  S  A  G  D  G  K  F
ggagatgaacaaaataaacctcttccttcatctgttgatgcaaagcttgaagctatttgt
 G  D  E  Q  N  K  P  L  P  S  S  V  D  A  K  L  E  A  I  C
cagagtttggctattaataactctgcctctcagaaacaagggaagatttccaaaccagtt
 Q  S  L  A  I  N  N  S  A  S  Q  K  Q  G  K  I  S  K  P  V
agagtttctagtaagaagactaagaaaatcatggtcactgaagctgctgctgttgttgat
 R  V  S  S  K  K  T  K  K  I  M  V  T  E  A  A  A  V  V  D
tcaactccatcagtggtagttccagagaaggattcgtcatcatcaaaatgtgaaatgggt
 S  T  P  S  V  V  V  P  E  K  D  S  S  S  S  K  C  E  M  G
tttctgggttcatcacattcagaggtttacaaagtggagaattcgttgtcaccaccatta
 F  L  G  S  S  H  S  E  V  Y  K  V  E  N  S  L  S  P  P  L
agcagtagtgatgatgtatcagttttatcatattcttcacctgaatcttgcattgagatc
 S  S  S  D  D  V  S  V  L  S  Y  S  S  P  E  S  C  I  E  I
ccagatttcacagaacaagcatggaacaatgattgttcttcatcagagaattttatgttg
 P  D  F  T  E  Q  A  W  N  N  D  C  S  S  S  E  N  F  M  L
cagaaatatccttcttatgaggttgattgggctgctatcctttcttag
 Q  K  Y  P  S  Y  E  V  D  W  A  A  I  L  S Stop
ggtctagaatggcagctacaaaatattagggctgctatcctttcttagggatccccgagg
gctgctatcctttcttagggatcccca
```

Supplementary figure 1: Full length assembled alignment of MaRAP2-4 nucleotide and amino acid sequence with 5’ and 3’ UTRs. Blue colour indicates 58 amino acid long DBD.

Supplementary figure 2

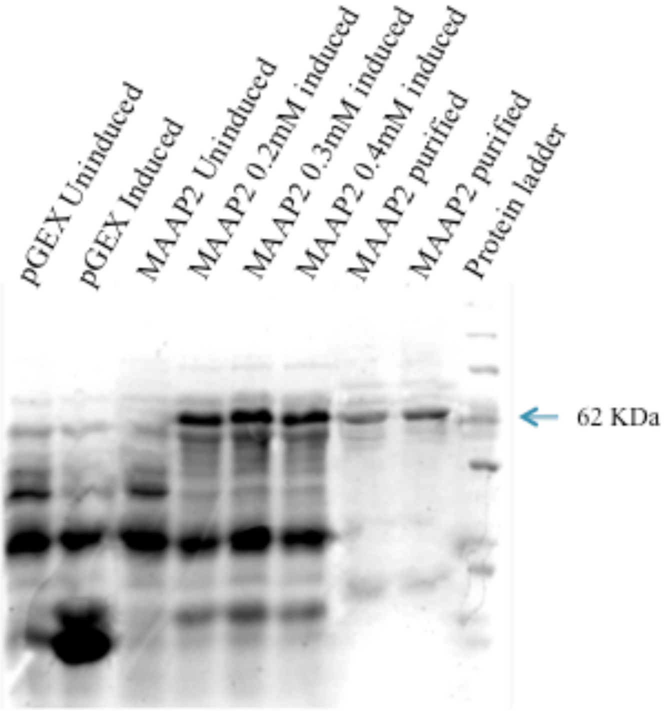

Supplementary figure 2: Induction of MaRAP2-4-GST with different concentration of IPTG and its purification with Sepharose GST beads (Amersham).

Supplementary figure 3

DRE1:AGAGCCGACAGATGCCGACTGGCCGACCT

DRE1(M):AGAGCCTACAGATGCCTACTGGCCTACCT

DRE2:AGATACCGACATAGATTACCGACATTGTACCGACATCT

DRE2(M):AGATACTGACATAGATTACTGACATTGTACTGACATCT

GCC:AGAAGCCGCCAGATAGCCGCCTGAGCCGCCCT

GCC(M):AGAAGCCACCAGATAGCCACCTGAGCCACCCT

JARE:AGACATGAATTAGATCATGAATTTGCATGAATTCT

JARE(M):AGACATGCATTAGATCATGCATTTGCATGCATTCT

Supplementary figure 3: Sequences of probes used for EMSA. Red colour indicates *cis*-element and blue colour indicates substitution in *cis*-element.

Supplementary figure 4

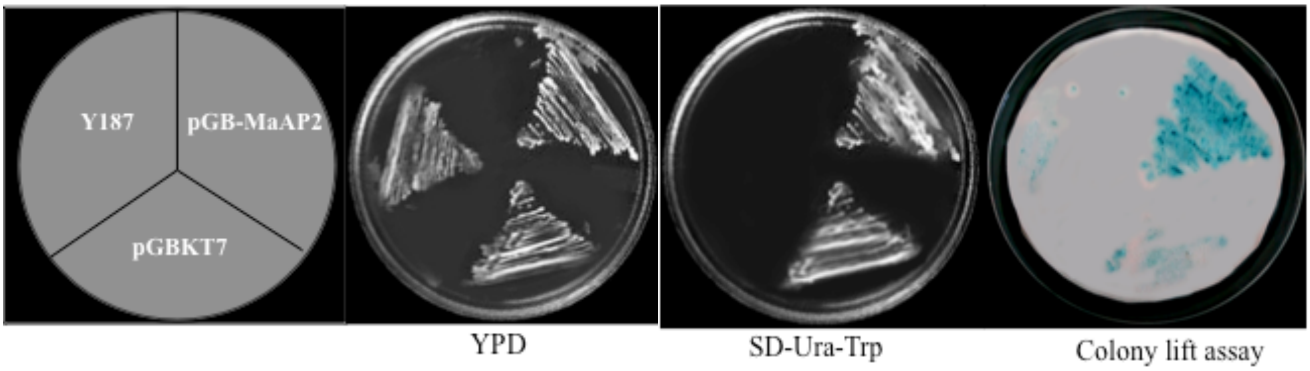

Supplementary figure 4: Transactivation/β-Galactosidase colony lift assay in which MaRAP2-4-GAL4-DBD activates target gene LacZ to produce blue colour.

Supplementary figure 5

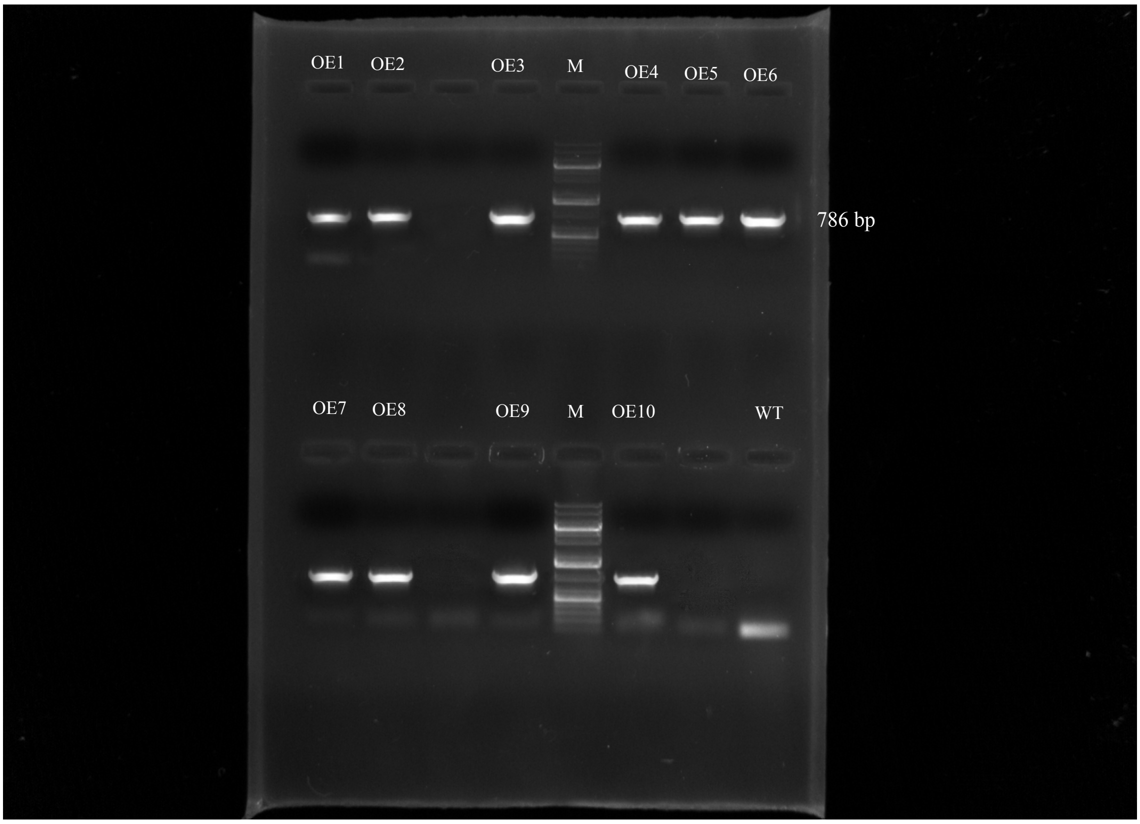

Supplementary figure 5: Genomic DNA PCR with NPT2 (Kan^R) primers for confirmation of MaRAP2-4 transformation in *Arabidopsis*

Supplementary figure 6

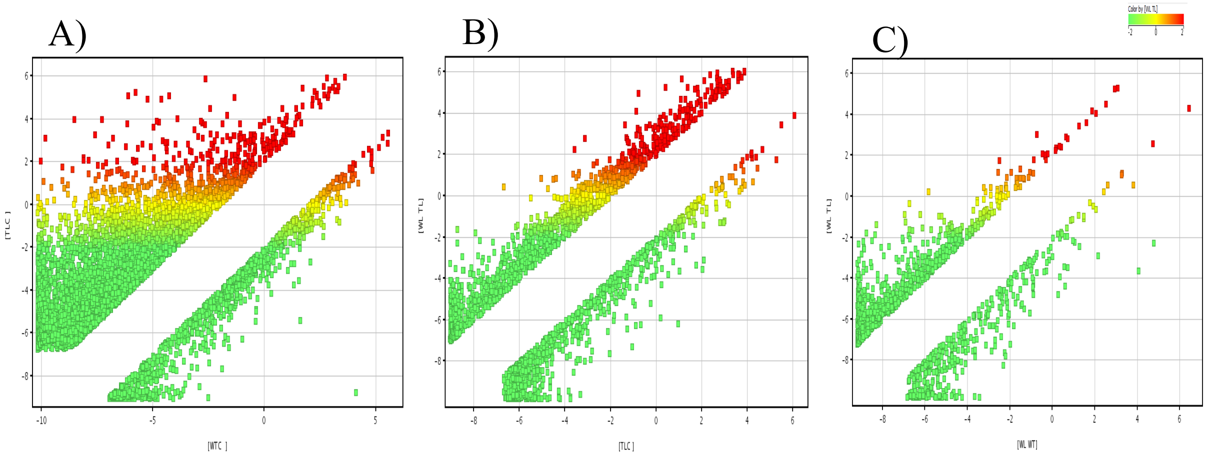

Supplementary figure 6: The Scatter Plot shows a 2-D scatter of differentially regulated probes for fold change >2 and <-2 above of the active entity. The axes of the scatter plot is based on intensity in terms of log to the base 10 in each group. Red colour represents up-regulation and green represents down-regulation. A) RAP2-4OE vs WT under control condition. B) OE-6D-WL vs RAP2-4OE. C) OE-6D-WL vs WT-6D-WL.

Supplementary figure 7

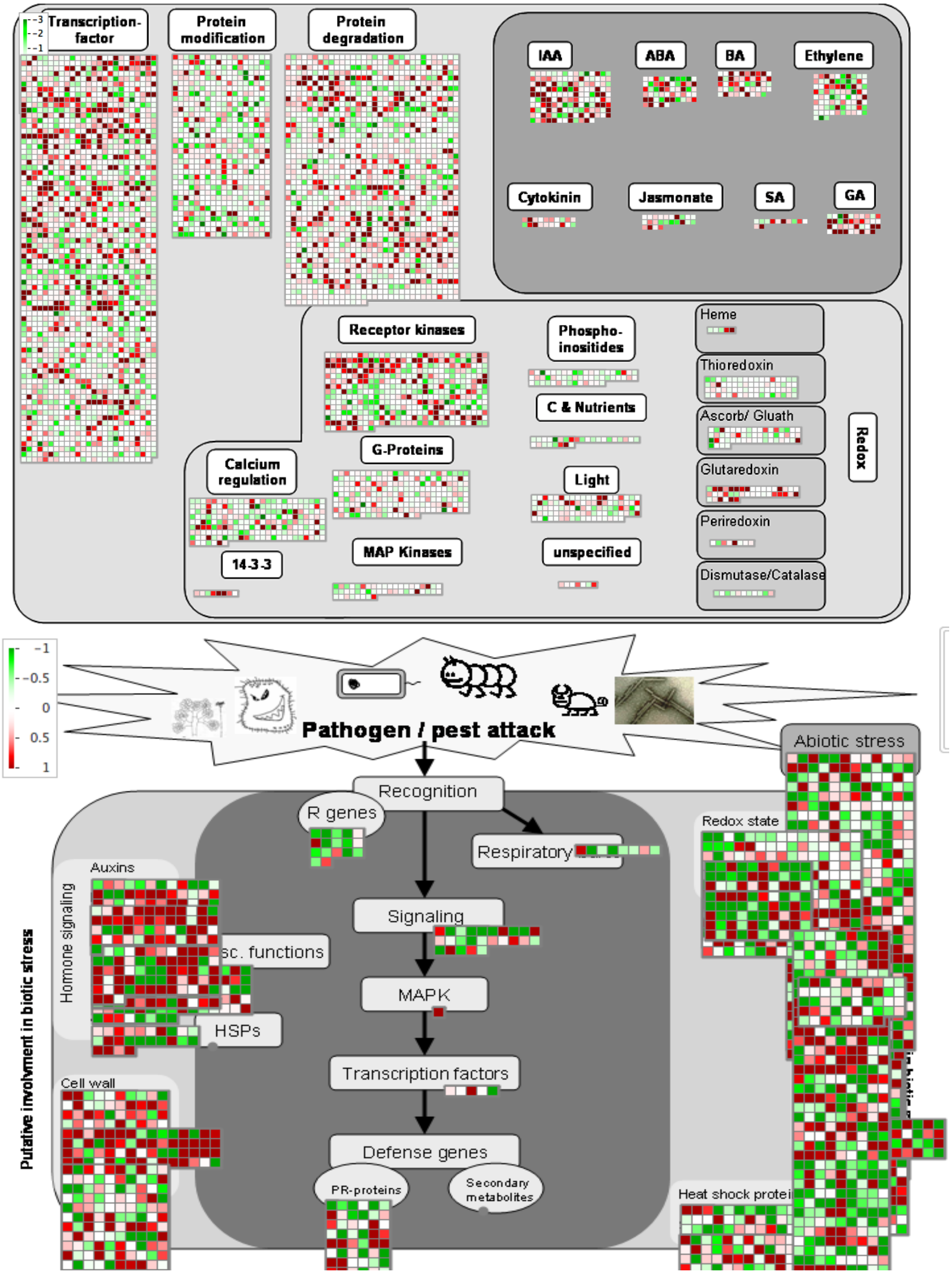

Supplementary figure 7: Normalized log2 expression values for up- and down- regulated genes of MaRAP2-4 vs WT under control condition are plotted onto boxes corresponding to their putative functional annotation using Mapman 3.6. Color intensity reflects the extent of increase in expression level, with more intense color (red- up-regulation, green- down-regulation) means more up- or down-regulation.

Supplementary figure 8

Arabidopsis thaliana Sweet 10 promoter- 9.34 fold

ATTGATGAAACCAAAGCAAAAAATTAAACGAACGAAATGAAATCATCTTGCAAATGAAGAAGAAGGGGA
ATAGTAAAAACAGAGTTTCAGATTTGTGGCAGATCTGCAATCGAGTATTCGAAATCAAAGCTTTTCAATAAT
CATTTGCGTCCTCACTATTCACCATTAATGATATTTTGTTTATTGGAAGAGAATTCCATTCAAAAACTAAACC
CTAATGATTATTGTAAAGGGAACCCAAGCAAAAAAAATCAAACGCACATTGATAAGTTTCTGAGATAGAAT
CTGATGATAAAGAGGAAGATCAGAGAAAAGAAAAAGAAACTGAGCCATCTCTCTCA**TGATTCACGG**
CCGCCGACGCCGTTCGTTACCATTGAAGGAGCTCATCTTAAGGGACACAACCAATCAACTATC
GTCACCGGAGCTAGCCCCGCTTCAATCACGTTGTAAAAAATCAATTGATCTGCAGAGAGAGAAAGAGATTT
GATGGAAACGGAGACAGATCCAGTTGTTAATGGCGA**TGATGATGGCGTCGGTGGTTACGTTG**
GTGATGATGATGATGAGGGTTGATAACGACGACGACGACAACACAAAAAAATCATACAATTTTTATTTG
TTTTCTCTAAATGATGAATCAAGGGTAATAGCGTCATTTCAAATAAGTAAAAGTACCTGATTTCGTAAGCCAT
CTTAAAAGTACCGGATTCTATAAATTTTTATGTTTTTGTGGTTTTTTAGACAAATAAGTCTCTGTAATTTCTTT
GATTATGCACTGGTTGAACATATATATTTGGCAACTTAAATATAAAAGAATCATATAAGGGTTTTGAAAATTT
TAGAGCATTTATGGAGTGGTATTTCAAAATTATCAGCTCTGCGAGAAAACAAGACAATAGCGTTACGAGTG
GATTTTTGTTCTGAAAGTATCTAATAGAACAGTGGATGACAAACTCTATATGAAATATAGAATTTTACTATAA
GTATACTATTTAACGAGAAGATATATAAATAAAATTTCGCCTTATAAGTTTGTTAAAGTTGTACATGGTTCAAT
AGATATGCAGACTAATCCAAAAGTCACCCAATACCTATAGCAAACATAAAAATAATAATATTTATAAAAGAAT
GAACCAAAAACTTATAAAAAGTTAAGAACTATAGAAATTCGTGATGAGATACATGTGAATTACCAACGAGA
GACTCGATTACATGAGTTTCTCTTAACTAGTTTAACAAAACATGGTTCATCTTATACTACAAAAAATAAGCC
AATCTAGACATGATTAAAAACAAATTAGTGTTATATTACAAAAGTTTCCCTCAAAAAAAACACATTTTGAGG
AAGCTAATCATGTCTAAACAAAGTAAATTCATTACATCACAAAATTGTGGAAAAGAGCAAGATGTAGTATAT
CTGTAATCAAATATGTTCAAACGAGCATCAAAATTCACCTAAACAACCTTCTGAACACTATGATAATATCTTC
AATCTGTTTAGTATATCTTTTACAATCGTGATTATTCTTTCTTTGGTTCCCCGCTCGCTCCATTGAATAGGACT
AATTTATATTGTTCACATTGCGGATTCACCTACTGGAAGTGTAAGAGTTAATGCAGCCAACCTCATTTTTTCC
AGAGAGATTACATAAAGACCTAAATAAAAATTGGGGAAACATATATTCTTTTCTTTCTTTTTGTTAATATGTT
GGCGGAAAGATTTCAAAAGGGGATTTCCCTAAATGATAGCTTTACATTTAGAAAACGAACTATGAGCATGA
CAATTCGGTTTAGGTAAAAGACCAACTTGCTCTCATTGCACTTGTAAAACCCTAAGCAAGAGAGTTGAAAA
CAGTAAACGTCTAAGCACCTACTTGGTTGGTGACACGTCGGAAAGGCCAAAATTTTGGTCCACTAGAATGT
ACCAGAATGGTACTTGCAAGATTTTTAAGGTTTTAGTGCTCATAAGTTGCTGAGCTGGATAGATACCCACCC
ACATTTTAGGTTTTAGGGTTTTGATTATTCCCCACAAATTTTATGAGATAAGATTTAGGTTA**ATTTCGCAT**
GCCGACATCAATGTGCGCACAACATCATTCATTCGTTTCATTTGATGAACAAAAAAAAAAATG
GTGAAATTACCCTTCTCATCAAAGATATTATTATAGAGTGAACATTATGTGAATTTTCTTGAAATGGAGAGAA
TTCTATTCCTCACGATATATCTAGATTATATATATATTTTTTTAATATATATATCTAGAATATATATTTCCTATCAAG
AGTCACTGATTTATATTGGTCACCCAAAATTGATCTTACCCAATTCATACACAATTACATTATTTTGCTAAAAC
TTTATACACGATTCATGCTAATAAAACAGTAATATATGACTCGTATTATGCTATATAGAGTGACCACTGACCAT
ACATTCTTCTTGAGGAAACTAGTATTCGAGAAAACTTAGTCATGTTCAGATATGCAGATCTAAAAGCTCATA
AAAATTAACGGTTTAACCATTTACATAAACACACAAAAAACTATGTATAAATTGAGTACATGAGTCATAAAA
TCGATAGATGGGTAAGATCATTATGCTTAGGTGAAAGAAAAAAAAGGGAACCTTCCGAGGCAATGGGGCAT
GAAGTATACAACTGTAAAGTAAGAGATAATGCTCTTTGATTCTTTTTTCCCCACTATACATCCCATTGAGTTC
AAACTCTTCTTTCTCTCTCTCTCTCTCTCTAGGTCTCTCACTCACAGACGCATTTTGCAAGTTGATACACTTT
TCTTCCTCCTATATAAATCACTACACTTCTCAATCAAAAATTGT

Supplementary figure 8: Promoter sequence of AtSWEET10 with MaRAP2-4 interacting *cis*-motifs

Supplementary figure 9

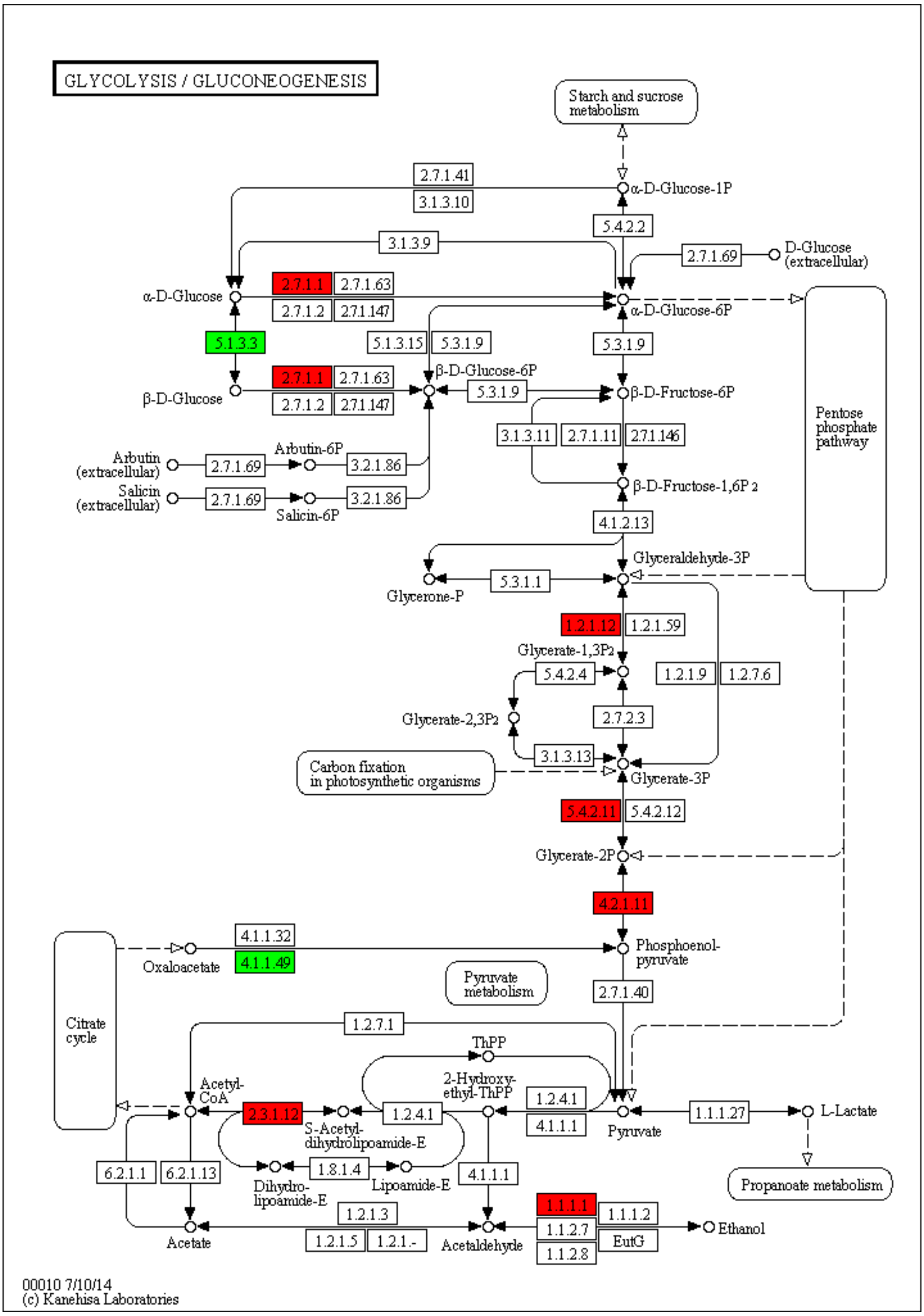

Supplementary figure 9: Up- and down-regulated genes in Glycolysis/ Gluconeogenesis. Red colour represents up-regulation and green colour represents down-regulation.

Supplementary figure 10

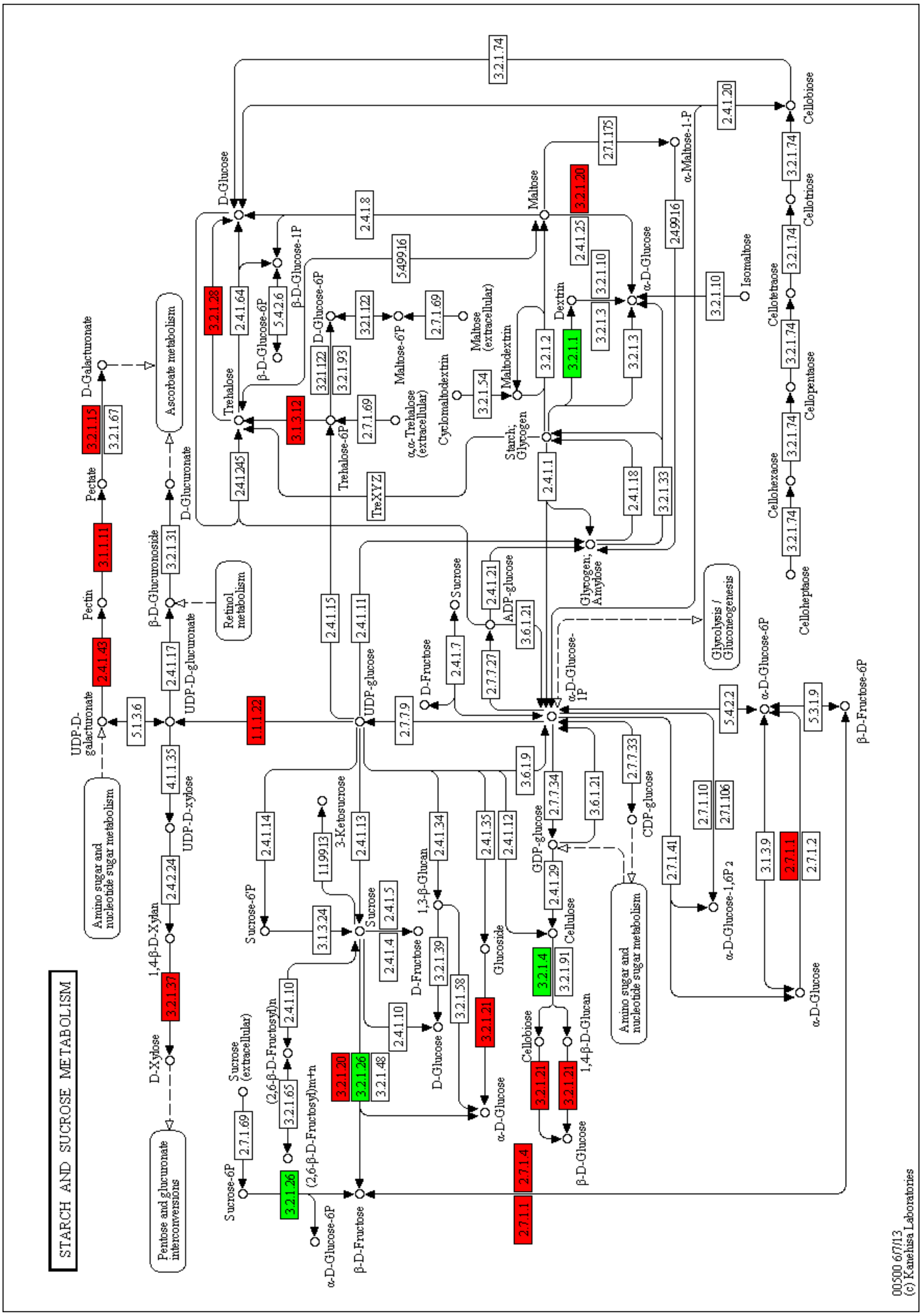

Supplementary figure 10: Up- and down-regulated genes in Starch and Sucrose metabolism. Red colour represents up-regulation and green colour represents down-regulation.

Supplementary material 1: List of primers with their sequences used in this study.

Designation	IDs	Sequence	Tm °C	Category
APRACE3'		TTGACTTGGTACCTGCCCGG	63.94	RACE
APRACE5'		TTTCCAGTGGTTATTACCGG	56.03	
RAP-RTF		TCACCTGAATCTTGCATTGAGATC	61.28	qRT-PCR *Mentha*
RAP-RTR		TAGCAGCCCAATCAACCTCAT	60.42	
MACTINF		GGATATTCAGCCCCTCGTCT	58.95	
MACTINR		GTAGAAGGTATGATGCCAAA	51.31	
RAP-pGEXF		GGGATCCTCTTCTACAGGTACCCAA	63.43	for pGEX4T2 cloning
RAP-pGEXR		TCTCGAGCTAAGAAAGGATAGCAGCCCA	69.26	
RAP-pBIF		GTCTAGATCTTCTACAGGTACCCAA	55.87	for pBI121 cloning and transgenic confirmation
RAP-pBIR		TGGATCCCTAAGAAAGGATAGCAGCCCA	70.32	
NPT2F		GAACAAGATGGATTGCACGC	60.05	
NPT2R		GAAGAACTCGTCAAGAAGGC	55.17	
RAP-pGBF		GCATATGTCTTCTACAGGTACCCAA	59.31	for pGBKT7 cloning
RAP-pGBR		TGGATCCCTAAGAAAGGATAGCAGCCCA	70.32	
RAP-pGB5D		GCCCGGATCCCTAAGCCCAATCAACCTCATA	76.03	for cloning of different truncations in pGBKT7
RAP-pGB10D		GCCCGGATCCCTACTGCAACATAAAATTCTC	71.72	
RAP-pGB30D		GCCCGGATCCCTATTGTTCTGTGAAATCTGG	74.07	
RAP-pGB45D		TGGATCCCTAATATGATAAAACTGATGC	62.18	
RAP-pGB60D		TGGATCCCTACAACGAATTCTCCACTTTG	69.02	
RAP-pGB75D		TGGATCCCTAAAAACCCATTTCACATTTTG	68.96	
RAP-pGB90D		TGGATCCCTATACCACTGATGGAGTTGAATC	68.83	
RAP-pGB105D		TGGATCCCTACATGATTTTCTTAGTCTTC	62.69	
RAP-pGB138D		TGGATCCACTATTCAAGCTTTGCATCAAC	68.29	
D1F		AGACTCAATTTCCCACCTCTTCCTTCATCTGTT	70.2	for cloning of different internal deletions in pGBKT7
D1R		AGATGAAGGAAGAGGTGGGAAATTGAGTCTGGC	73.31	
D2F		GATGAACAAAATAAAAACTCTGCCTCTCAGAAA	66.67	
D2R		CTGAGAGGCAGAGTTTTTATTTTGTTCATCTCC	67.87	
D3F		AGTTTGGCTATTAATACTAAGAAAATCATGGTC	62.07	
D3R		CATGATTTTCTTAGTATTAATAGCCAAACTCTG	61.7	
RAP-pGADF		GGGATCCTCTTCTACAGGTACCCAA	63.43	for pGAD cloning
RAP-pGADR		TCATATGCTAAGAAAGGATAGCAGCCCA	66.33	
PHISW3F		TGAATTCAATAAGCCAATCTAGACATG	60.48	For cloning promoter fragments in pHIS2.0
PHISW3R		TGAGCTCAATTCACATAATGTTCACTC	61.46	
PHISW1F		TACGCGTATTGATGAAACCAAAGCAAA	66.71	
PHISW1R		TACTAGTCGACGCCATCATCATCGCCA	71.51	
TPPB trehalose-6-phosphate phosphatase	AT1G78090	CCAAGGTAGAAAGGTACTTGAAATCC	60.38	Sugar metabolism/transport responsive genes
		TGAGTGCCTGGCCCTTGT	61.24	
cwINV4 beta-fructofuranosidase	AT2G36190	TTCTCATGTGCTCCGATGCTA	60.34	
		TGCGAGTAAACCCGTGTCTTG	61.92	
STP5 sugar transport protein 5	AT1G34580	TGACGATATTCGTCATCATGTTCTT	60.54	
		TGCTTCTCCCAAACTTGATACATAGA	61.07	
STP14 sugar transport protein 14	AT1G77210	TGTGGTGTGCGTGAACCTTT	60.99	
		CACAGCGACACCAGGAAACA	61.71	
SUS1 sucrose synthase 1	AT5G20830	TCGAACCTTGACCGTCTTGAG	61.15	
		ATGGGCGATACTTCAATGCAT	60.14	
adh like	AT1G09510	CAGTTTTGAATGCATTGGTTTAGC	60.65	
		CCCGGGCGAGTACTCTTGA	62.06	
Sucrose synthase 3	AT4G02280	AATTCCGCGACTTGGTGAAA	61.32	
		GCTGAACCGGGTCAGTCATC	61.44	
Pyruvate, phosphate dikinase 1	AT4G15530	GTCGGCAAGTTTCTACCGATTT	60.3	
		TCAAAAGGGTCGTGCTGTAAGA	60.6	
Sugar transporter ERD6-like 12/ERD6	AT3G05400	TTTCCAGTCGTTTGGCATTCT	60.44	
		CGCCGATGCAAGTGAAGA	60.49	
Glutamate decarboxylase 3/GAD3	AT2G02000	GTGGCCGAAATGCTTCGT	60.59	
		ATCCGCAGGCATCGTGTAG	60.62	
Sweet10	AT5G50790	AGCTTTTAACTATCAAATGTCGATGTG	59.81	
		GACCCAAATAAAAACATTAAACCCTATC	60.21	

Sweet1	AT1G21460	TGGGTTTGGATGTGCATTAGG	61.43	
		TGCAGATTTCTCTCCTTTGTTTCC	61.66	
Pyruvate decarboxylase 1/ PDC1	AT4G33070	GGAGAAGAAAGATTGTCTATGTTTCATAGA	60.47	
		GCGTGAGCCCCACTCAAG	61.39	
Peroxidase9/PERX9	AT5G42180	AGCCTCTCCTGCAAGATTCG	60.45	antioxidant responsive genes
		CAATAAGCCTTTGCCCCATAGA	61.05	
Catalase 3/CAT3	AT1G20620	TGGTCGCTTGGTTCTGAACA	61.21	
		GCAAGCTGCTCAGTTTCATTGA	61.39	
L-ascorbate peroxidase 2/APX2	AT3G09640	GCCACAAGGAGCGTTCA	60.98	
		CAAAAATGAGCGGGTTTGGT	60.66	
Iron/manganese SOD family protein/FSD1	AT3G56350	GGGAAGCTGTAAGTGCCAGACT	60.62	
		CAAATTGTTCAGAAATTTGCTTAAGC	60.29	
ERF transcription factor ERF071/HRE2	AT2G47520	CAAATGGGCAGCGGAGAT	60.55	TFs
		TGTGCCAAGCCAGACACGTA	62.25	
ERF transcription factor ERF113/RAP2.6L	AT5G13330	TTTCAACGCCTTCTTCATCTTCT	60.18	
		TGTAACCATAATTCTTCTCTTCTTCTTCA	59.89	
NAC domain containing protein 47/SHYG	AT3G04070	TTGGATCAGAAGTTACCGCAGTT	60.81	
		CTTCCGATGCCGCTTGTC	61.3	
Arabidopsis Actin 1	AT2G37620	CTTGGCCTCCCTCAGTACCTT	60.44	actin
		TGAACAATCGATGGACCTGACT	60.33	

Supplementary material 2: List of differentially expressed ESTs after SSH analysis of *M. arvensis* and *M. piperita* (red colour indicates the gene of study)

Sl No.	Gene bank accession	Annotation
1	JZ468944	17.9 kDa class II heat shock protein
2	JZ468946	syntaxin-43-like
3	JZ468947	Integrase-type DNA-binding superfamily protein
4	JZ468948	dehydration responsive element binding protein
5	JZ468949	ethylene-responsive transcription factor RAP2-4-like
6	JZ468950	AP2 4, putative
7	JZ468951	AP2/ERF domain-containing transcription factor
8	JZ468952	hypothetical protein VITISV 026680
9	JZ468953	uncharacterized protein LOC100839475
10	JZ468954	No significant similarity
11	JZ468955	ribulose bisphosphate carboxylase small chain 1, chloroplastic-like
12	JZ468957	Cysteine-rich RLK (RECEPTOR-like protein kinase) 29
13	JZ468958	beta-carotene isomerase D27, chloroplastic-like
14	JZ468960	predicted protein
15	JZ468961	SGT1, putative isoform 1
16	JZ468962	uncharacterized protein LOC101246360 isoform 1
17	JZ468963	acetohydroxyacid synthase
18	JZ468964	acetolactate synthase protein
19	JZ468965	hypothetical protein CL192Contig1 03
20	JZ468966	vacuolar protein 8-like
21	JZ468967	peptidyl-prolyl cis-trans isomerase FKBP16-4, chloroplastic-like
22	JZ468968	rubisco activase
23	JZ468969	fructose-1,6-bisphosphatase
24	JZ468970	No significant similarity
25	JZ468971	No significant similarity
26	JZ468972	No significant similarity
27	JZ468973	hypothetical protein VITISV 009255
28	JZ468974	hypothetical protein VITISV 009561
29	JZ468975	leukocyte receptor cluster (lrc) member, putative
30	JZ468976	hypothetical protein VITISV 010970
31	JZ468977	predicted protein
32	JZ468978	cysteine-rich receptor-like protein kinase 15-like, partial
33	JZ468979	hypothetical protein OsJ 30144
34	JZ468980	predicted protein
35	JZ468981	17.9 kDa class II heat shock protein
36	JZ468982	No significant similarity
37	JZ468983	unnamed protein product

Supplementary material 3: List of up- and down-regulated genes (HE vs WT) with their normalized fold induction and adjusted p value

Normalized Fold WT	Fold RAP2-4OE 1	Fold RAP2-4OE 2	Normalized Fold RAP2-4OE	Adjusted P value	Gene ID
0.00	12.51	12.54	12.52	0.0008	AT1G56100
0.00	12.95	11.69	12.32	0.0325	AT4G15750
0.00	12.10	11.70	11.90	0.0107	AT2G42840
0.00	11.04	12.30	11.67	0.0343	AT5G20630
0.00	11.29	10.83	11.06	0.0132	AT3G20210
0.00	10.21	11.52	10.86	0.0383	AT5G07530
0.00	10.32	11.03	10.67	0.0212	AT5G45670
0.00	11.21	10.08	10.64	0.0338	AT3G08770
0.00	10.87	10.35	10.61	0.0156	AT4G29030
0.00	10.21	10.74	10.47	0.0161	AT1G24020
0.00	10.28	10.07	10.18	0.0066	AT1G72260
0.00	10.50	9.64	10.07	0.0272	AT1G06080
0.00	10.37	9.57	9.97	0.0255	AT1G61720
0.00	10.15	9.50	9.82	0.0211	AT5G33370
0.00	9.34	10.29	9.81	0.0308	AT4G12870
0.00	9.90	9.14	9.52	0.0254	AT5G47500
0.00	9.81	9.17	9.49	0.0215	AT5G49180
0.00	10.06	8.87	9.46	0.0400	AT5G39220
0.00	9.75	9.14	9.44	0.0206	AT1G11850
0.00	9.34	9.40	9.37	0.0020	AT4G14730
0.00	9.77	8.93	9.35	0.0286	AT1G16390
0.00	9.32	9.29	9.30	0.0010	AT5G50790
0.00	9.22	9.35	9.29	0.0045	AT1G71680
0.00	9.95	8.54	9.25	0.0485	AT1G13710
0.00	9.64	8.85	9.24	0.0272	AT3G54340
0.00	9.37	8.81	9.09	0.0196	AT4G12960
0.00	8.54	9.41	8.97	0.0308	AT5G42235
0.00	9.61	8.28	8.95	0.0472	AT1G11600
0.00	8.75	9.13	8.94	0.0135	AT5G21150
0.00	9.19	8.69	8.94	0.0178	AT5G22430
0.00	9.18	8.60	8.89	0.0208	AT1G19320
0.00	8.88	8.86	8.87	0.0007	AT1G53130
0.00	8.74	8.85	8.79	0.0040	AT2G38540
0.00	9.16	8.34	8.75	0.0298	AT4G33145
0.00	9.05	8.43	8.74	0.0226	AT1G72290
0.00	8.40	9.09	8.74	0.0251	AT5G01075
0.00	8.63	8.80	8.72	0.0062	AT2G10940
0.00	8.40	9.02	8.71	0.0226	AT1G71690
0.00	8.46	8.94	8.70	0.0176	AT5G08000
0.00	8.08	9.17	8.63	0.0402	AT3G04280
0.00	8.66	8.56	8.61	0.0037	AT5G51950
0.00	8.38	8.76	8.57	0.0141	AT5G09370
0.00	7.98	9.14	8.56	0.0431	AT4G12890
0.00	8.94	8.18	8.56	0.0282	AT2G46960
0.00	9.02	7.93	8.47	0.0409	AT1G26945
0.00	8.44	8.46	8.45	0.0008	AT3G16370
0.00	8.46	8.35	8.41	0.0042	AT5G24910
0.00	8.15	8.53	8.34	0.0145	AT5G55720
0.00	8.21	8.42	8.32	0.0080	AT1G10540
0.00	8.49	8.08	8.29	0.0157	AT5G47600
0.00	8.62	7.91	8.27	0.0273	AT4G22880

0.00	8.16	8.34	8.25	0.0069	AT3G25260
0.00	8.60	7.89	8.25	0.0274	AT5G50335
0.00	8.34	8.11	8.22	0.0089	AT1G23205
0.00	7.94	8.48	8.21	0.0209	AT4G32490
0.00	8.06	8.31	8.18	0.0097	AT3G17600
0.00	7.96	8.39	8.17	0.0167	AT5G07550
0.00	8.67	7.64	8.16	0.0402	AT1G71691
0.00	7.70	8.58	8.14	0.0344	AT2G14247
0.00	8.32	7.92	8.12	0.0157	AT1G55260
0.00	7.84	8.32	8.08	0.0189	AT3G12610
0.00	7.80	8.34	8.07	0.0213	AT5G25460
0.00	8.63	7.48	8.05	0.0454	AT5G63230
0.00	7.87	8.20	8.04	0.0131	AT1G73620
0.00	8.00	8.02	8.01	0.0008	AT3G26140
0.00	7.99	7.98	7.98	0.0004	AT5G51490
0.00	7.86	8.10	7.98	0.0096	AT4G11320
0.00	7.54	8.41	7.97	0.0347	AT4G22870
0.00	7.91	8.02	7.97	0.0044	AT2G32370
0.00	8.04	7.90	7.97	0.0056	AT4G29305
0.00	8.10	7.72	7.91	0.0153	AT1G10640
0.00	7.29	8.52	7.90	0.0494	AT2G47900
0.00	7.95	7.82	7.88	0.0052	AT2G04034
0.00	8.28	7.43	7.86	0.0344	AT4G32170
0.00	8.15	7.49	7.82	0.0268	AT4G22050
0.00	8.09	7.52	7.81	0.0232	AT2G42990
0.00	7.98	7.60	7.79	0.0155	AT4G15160
0.00	7.47	8.10	7.79	0.0257	AT2G27385
0.00	7.56	8.00	7.78	0.0180	AT3G14210
0.00	8.19	7.37	7.78	0.0335	AT4G21080
0.00	7.18	8.20	7.69	0.0422	AT5G56300
0.00	7.87	7.48	7.68	0.0162	AT1G51000
0.00	7.67	7.68	7.67	0.0004	AT4G28365
0.00	7.32	7.94	7.63	0.0259	AT1G27080
0.00	7.76	7.49	7.63	0.0113	AT5G24900
0.00	7.14	8.06	7.60	0.0385	AT2G02147
0.00	8.10	7.10	7.60	0.0418	AT5G55320
0.00	7.84	7.33	7.59	0.0214	AT4G16590
0.00	7.66	7.49	7.57	0.0071	AT5G60140
0.00	7.91	7.20	7.56	0.0299	AT4G39330
0.00	7.20	7.91	7.56	0.0299	AT4G09960
0.00	7.91	7.19	7.55	0.0303	AT3G25750
0.00	7.25	7.83	7.54	0.0245	AT1G61095
0.00	7.39	7.68	7.54	0.0122	AT4G26590
0.00	7.98	7.09	7.53	0.0376	AT1G26796
0.00	8.00	7.06	7.53	0.0397	AT2G33880
0.00	7.61	7.42	7.51	0.0080	AT1G23240
0.00	7.69	7.32	7.50	0.0157	AT2G35310
0.00	7.25	7.73	7.49	0.0204	AT4G35725
0.00	7.21	7.76	7.49	0.0234	AT1G80660
0.00	7.32	7.64	7.48	0.0136	AT4G22400
0.00	7.66	7.29	7.48	0.0158	AT1G49800
0.00	6.98	7.97	7.48	0.0421	AT5G46950
0.00	7.15	7.79	7.47	0.0273	AT1G12570
0.00	7.66	7.27	7.47	0.0166	AT2G27880
0.00	7.52	7.41	7.46	0.0047	AT1G50310
0.00	7.57	7.34	7.46	0.0098	AT5G44440
0.00	7.32	7.54	7.43	0.0094	AT1G51035
0.00	6.90	7.95	7.42	0.0449	AT3G60670
0.00	7.91	6.94	7.42	0.0415	AT4G14750

0.00	7.81	7.01	7.41	0.0343	AT1G48195
0.00	7.49	7.32	7.41	0.0073	AT2G42830
0.00	7.14	7.62	7.38	0.0207	AT4G36740
0.00	6.93	7.82	7.37	0.0384	AT1G04645
0.00	7.85	6.85	7.35	0.0432	AT5G19700
0.00	7.22	7.48	7.35	0.0113	AT5G01790
0.00	7.76	6.86	7.31	0.0391	AT3G52780
0.00	7.41	7.20	7.31	0.0091	AT5G07160
0.00	7.07	7.53	7.30	0.0201	AT3G24280
0.00	7.61	6.96	7.29	0.0284	AT1G20730
0.00	7.62	6.95	7.28	0.0293	AT4G27420
0.00	7.77	6.77	7.27	0.0437	AT1G15360
0.00	7.60	6.90	7.25	0.0307	AT1G70560
0.00	7.31	7.13	7.22	0.0079	AT1G52695
0.00	7.25	7.18	7.22	0.0031	AT2G04925
0.00	6.96	7.43	7.20	0.0208	AT1G43710
0.00	7.26	7.13	7.19	0.0058	AT3G05600
0.00	7.19	7.16	7.18	0.0013	AT4G00870
0.00	6.91	7.42	7.16	0.0226	AT5G24780
0.00	6.60	7.71	7.15	0.0493	AT2G41990
0.00	7.18	7.09	7.13	0.0040	AT4G32714
0.00	6.98	7.29	7.13	0.0138	AT1G43666
0.00	6.75	7.50	7.12	0.0335	AT3G10570
0.00	7.25	6.99	7.12	0.0116	AT5G49170
0.00	7.10	7.13	7.12	0.0013	AT2G34700
0.00	6.67	7.56	7.12	0.0398	AT1G62500
0.00	7.26	6.97	7.11	0.0130	AT5G58400
0.00	7.36	6.83	7.09	0.0238	AT2G42885
0.00	7.00	7.15	7.08	0.0067	AT5G22460
0.00	6.53	7.62	7.08	0.0489	AT5G27200
0.00	7.28	6.79	7.04	0.0222	AT1G04150
0.00	6.51	7.56	7.03	0.0474	AT2G12480
0.00	7.40	6.60	7.00	0.0287	AT1G34580
0.00	6.64	7.29	6.97	0.0297	AT5G63180
0.00	6.50	7.42	6.96	0.0420	AT2G19802
0.00	7.23	6.66	6.95	0.0261	AT5G24355
0.00	6.95	6.93	6.94	0.0009	AT2G25060
0.00	7.47	6.41	6.94	0.0485	AT1G77780
0.00	6.96	6.91	6.94	0.0023	AT5G47150
0.00	6.71	7.14	6.93	0.0198	AT3G21860
0.00	6.41	7.44	6.93	0.0473	AT5G63250
0.00	7.21	6.63	6.92	0.0267	AT5G61412
0.00	7.06	6.78	6.92	0.0129	AT1G26799
0.00	6.89	6.93	6.91	0.0018	AT1G61090
0.00	6.65	7.13	6.89	0.0222	AT5G38180
0.00	7.42	6.35	6.89	0.0494	AT5G15720
0.00	7.26	6.51	6.88	0.0346	AT2G42110
0.00	6.59	7.16	6.88	0.0264	AT5G60553
0.00	7.00	6.74	6.87	0.0120	AT2G40610
0.00	6.78	6.95	6.86	0.0079	AT4G15210
0.00	7.38	6.34	6.86	0.0482	AT5G44630
0.00	6.91	6.80	6.85	0.0051	AT1G15150
0.00	6.52	7.18	6.85	0.0306	AT4G26790
0.00	6.37	7.32	6.84	0.0441	AT5G07540
0.00	7.11	6.50	6.81	0.0285	AT3G10780
0.00	7.16	6.46	6.81	0.0327	AT4G30130
0.00	6.90	6.69	6.79	0.0098	AT4G26420
0.00	6.96	6.60	6.78	0.0169	AT4G12825
0.00	6.28	7.27	6.77	0.0464	AT1G65450

0.00	6.66	6.88	6.77	0.0103	AT1G12845
0.00	6.66	6.86	6.76	0.0094	AT3G60700
0.00	6.91	6.58	6.75	0.0156	AT5G08460
0.00	6.45	7.03	6.74	0.0274	AT1G09790
0.00	6.80	6.66	6.73	0.0066	AT2G21465
0.00	6.72	6.74	6.73	0.0009	AT5G50740
0.00	7.15	6.25	6.70	0.0427	AT4G28780
0.00	6.95	6.43	6.69	0.0247	AT3G20750
0.00	6.40	6.95	6.68	0.0262	AT3G12000
0.00	6.41	6.93	6.67	0.0248	AT5G15230
0.00	6.72	6.60	6.66	0.0057	AT5G18000
0.00	6.24	7.06	6.65	0.0392	AT5G24410
0.00	6.89	6.40	6.64	0.0235	AT1G31310
0.00	6.76	6.53	6.64	0.0110	AT4G33790
0.00	6.75	6.50	6.63	0.0120	AT3G43960
0.00	6.91	6.32	6.61	0.0284	AT1G17200
0.00	6.73	6.49	6.61	0.0116	AT5G07030
0.00	6.14	7.07	6.61	0.0496	AT2G02000
0.00	6.52	6.68	6.60	0.0077	AT5G38970
0.00	6.26	6.93	6.59	0.0323	AT5G15780
0.00	6.70	6.48	6.59	0.0106	AT2G24310
0.00	6.31	6.86	6.59	0.0266	AT3G23130
0.00	6.91	6.26	6.58	0.0314	AT4G17480
0.00	7.01	6.15	6.58	0.0415	AT3G19200
0.00	6.55	6.58	6.56	0.0015	AT1G21395
0.00	6.33	6.77	6.55	0.0214	AT1G28170
0.00	6.89	6.20	6.54	0.0335	AT3G62610
0.00	6.76	6.32	6.54	0.0214	AT5G38960
0.00	6.14	6.92	6.53	0.0380	AT5G43760
0.00	6.62	6.42	6.52	0.0098	AT5G13930
0.00	6.02	7.01	6.52	0.0483	AT3G05980
0.00	6.42	6.60	6.51	0.0088	AT5G48100
0.00	6.31	6.70	6.51	0.0191	AT1G78950
0.00	6.90	6.08	6.49	0.0402	AT4G14695
0.00	6.33	6.64	6.48	0.0152	AT3G42830
0.00	6.32	6.64	6.48	0.0157	AT4G22105
0.00	6.80	6.16	6.48	0.0314	AT2G20870
0.00	6.88	6.08	6.48	0.0392	AT1G05280
0.00	6.06	6.87	6.47	0.0398	AT1G09370
0.00	6.54	6.38	6.46	0.0079	AT3G13370
0.00	6.77	6.10	6.44	0.0331	AT1G43640
0.00	6.46	6.40	6.43	0.0030	AT1G12170
0.00	6.20	6.65	6.43	0.0223	AT3G13540
0.00	6.18	6.67	6.42	0.0243	AT1G58430
0.00	6.60	6.24	6.42	0.0178	AT1G49770
0.00	5.97	6.87	6.42	0.0446	AT1G48930
0.00	6.55	6.27	6.41	0.0139	AT1G66100
0.00	6.18	6.63	6.40	0.0224	AT5G49100
0.00	6.33	6.47	6.40	0.0070	AT5G23212
0.00	6.24	6.56	6.40	0.0159	AT3G50870
0.00	6.70	6.09	6.40	0.0303	AT1G13130
0.00	6.49	6.29	6.39	0.0100	AT5G25090
0.00	6.13	6.64	6.38	0.0254	AT2G41240
0.00	6.43	6.30	6.37	0.0065	AT3G21800
0.00	6.35	6.38	6.37	0.0015	AT4G31840
0.00	6.01	6.73	6.37	0.0359	AT5G17090
0.00	6.26	6.46	6.36	0.0100	AT4G23600
0.00	6.19	6.52	6.36	0.0165	AT2G41225
0.00	5.95	6.76	6.36	0.0405	AT3G12870

0.00	6.11	6.60	6.35	0.0245	AT5G48900
0.00	5.94	6.75	6.35	0.0406	AT3G06868
0.00	5.97	6.72	6.35	0.0376	AT5G11360
0.00	6.49	6.19	6.34	0.0151	AT3G50990
0.00	6.17	6.50	6.34	0.0166	AT1G75580
0.00	6.76	5.91	6.34	0.0426	AT1G06360
0.00	6.42	6.25	6.34	0.0085	AT3G01323
0.00	5.87	6.76	6.32	0.0448	AT5G50480
0.00	6.70	5.92	6.31	0.0393	AT1G47990
0.00	6.52	6.09	6.31	0.0217	AT4G10603
0.00	6.53	6.07	6.30	0.0232	AT2G42720
0.00	5.93	6.67	6.30	0.0373	AT3G03850
0.00	6.21	6.37	6.29	0.0081	AT3G61950
0.00	6.41	6.14	6.28	0.0137	AT5G53390
0.00	6.21	6.34	6.28	0.0066	AT1G28590
0.00	5.84	6.69	6.26	0.0431	AT5G61850
0.00	6.57	5.95	6.26	0.0315	AT5G46690
0.00	5.91	6.60	6.25	0.0351	AT2G02515
0.00	6.22	6.25	6.24	0.0015	AT2G02990
0.00	6.29	6.16	6.23	0.0066	AT5G20740
0.00	5.78	6.66	6.22	0.0450	AT5G19730
0.00	6.12	6.30	6.21	0.0092	AT1G53520
0.00	6.55	5.86	6.21	0.0354	AT5G15530
0.00	6.42	5.99	6.21	0.0220	AT4G10850
0.00	6.37	6.03	6.20	0.0175	AT5G54067
0.00	6.58	5.81	6.20	0.0395	AT3G01327
0.00	5.83	6.55	6.19	0.0370	AT3G17155
0.00	6.08	6.29	6.19	0.0108	AT2G47930
0.00	6.29	6.07	6.18	0.0113	AT3G16895
0.00	6.39	5.96	6.18	0.0222	AT4G33355
0.00	5.91	6.44	6.18	0.0273	AT3G05960
0.00	5.91	6.44	6.17	0.0273	AT1G49640
0.00	6.11	6.24	6.17	0.0067	AT3G20520
0.00	6.19	6.15	6.17	0.0021	AT4G39510
0.00	5.75	6.60	6.17	0.0437	AT1G16705
0.00	6.20	6.14	6.17	0.0031	AT5G07990
0.00	6.08	6.22	6.15	0.0072	AT1G78760
0.00	5.96	6.33	6.15	0.0192	AT4G39917
0.00	5.88	6.40	6.14	0.0269	AT5G59845
0.00	5.93	6.35	6.14	0.0218	AT2G04570
0.00	6.16	6.11	6.13	0.0026	AT5G12940
0.00	6.00	6.27	6.13	0.0140	AT4G18970
0.00	5.76	6.51	6.13	0.0389	AT4G22115
0.00	6.05	6.21	6.13	0.0083	AT1G09380
0.00	6.53	5.72	6.12	0.0420	AT3G29152
0.00	5.86	6.38	6.12	0.0270	AT1G17190
0.00	6.51	5.73	6.12	0.0405	AT3G20030
0.00	5.67	6.56	6.11	0.0462	AT3G17130
0.00	6.08	6.11	6.10	0.0016	AT4G18960
0.00	5.64	6.55	6.10	0.0474	AT2G37640
0.00	6.49	5.69	6.09	0.0418	AT1G61097
0.00	6.46	5.72	6.09	0.0386	AT3G12970
0.00	5.91	6.26	6.09	0.0183	AT3G18470
0.00	6.48	5.69	6.09	0.0413	AT3G05470
0.00	6.40	5.76	6.08	0.0335	AT3G12880
0.00	6.50	5.66	6.08	0.0439	AT1G67775
0.00	5.70	6.43	6.07	0.0383	AT5G45950
0.00	6.52	5.61	6.06	0.0477	AT4G20670
0.00	5.68	6.42	6.05	0.0389	AT5G01420

0.00	6.41	5.67	6.04	0.0389	AT3G61970
0.00	6.06	5.96	6.01	0.0053	AT2G29790
0.00	6.34	5.68	6.01	0.0349	AT2G30810
0.00	6.34	5.64	5.99	0.0372	AT1G80100
0.00	5.50	6.44	5.97	0.0500	AT1G12064
0.00	6.24	5.70	5.97	0.0288	AT4G21040
0.00	5.93	6.00	5.97	0.0037	AT3G22142
0.00	6.27	5.64	5.96	0.0336	AT1G22090
0.00	5.97	5.94	5.95	0.0016	AT2G37380
0.00	6.15	5.68	5.92	0.0253	AT1G60987
0.00	6.04	5.76	5.90	0.0151	AT1G31040
0.00	6.03	5.77	5.90	0.0140	AT3G54550
0.00	6.09	5.70	5.90	0.0211	AT2G38995
0.00	6.15	5.64	5.89	0.0275	AT2G40475
0.00	5.80	5.94	5.87	0.0076	AT5G08640
0.00	5.77	5.97	5.87	0.0108	AT1G75900
0.00	6.07	5.65	5.86	0.0228	AT1G78170
0.00	6.16	5.55	5.86	0.0331	AT5G38197
0.00	6.25	5.44	5.85	0.0440	AT4G22430
0.00	5.56	6.12	5.84	0.0305	AT2G20825
0.00	5.62	6.01	5.81	0.0213	AT1G02810
0.00	6.13	5.50	5.81	0.0345	AT3G24690
0.00	5.62	6.01	5.81	0.0213	AT5G13170
0.00	6.23	5.38	5.81	0.0465	AT1G59670
0.00	5.68	5.92	5.80	0.0132	AT2G28040
0.00	5.96	5.62	5.79	0.0187	AT1G75930
0.00	5.41	6.16	5.78	0.0412	AT5G01180
0.00	6.10	5.45	5.78	0.0358	AT1G52030
0.00	5.62	5.92	5.77	0.0165	AT2G38530
0.00	5.35	6.18	5.76	0.0457	AT4G10290
0.00	5.70	5.82	5.76	0.0066	AT3G05800
0.00	6.15	5.35	5.75	0.0442	AT1G45191
0.00	5.37	6.11	5.74	0.0410	AT4G02830
0.00	6.13	5.34	5.74	0.0438	AT1G08985
0.00	5.86	5.61	5.74	0.0139	AT3G23450
0.00	5.48	5.96	5.72	0.0267	AT3G22000
0.00	5.74	5.69	5.72	0.0028	AT2G28790
0.00	5.49	5.95	5.72	0.0256	AT5G26250
0.00	5.64	5.78	5.71	0.0078	AT1G67750
0.00	6.05	5.36	5.71	0.0385	AT2G16220
0.00	5.78	5.60	5.69	0.0101	AT4G03945
0.00	5.55	5.83	5.69	0.0157	AT4G11140
0.00	6.13	5.26	5.69	0.0485	AT4G13380
0.00	5.73	5.63	5.68	0.0056	AT4G20940
0.00	5.51	5.85	5.68	0.0190	AT5G12270
0.00	5.46	5.89	5.68	0.0241	AT2G41340
0.00	5.24	6.11	5.68	0.0487	AT3G22120
0.00	6.10	5.23	5.67	0.0488	AT3G05460
0.00	5.40	5.91	5.65	0.0287	AT3G48300
0.00	6.06	5.22	5.64	0.0473	AT1G68500
0.00	5.43	5.84	5.63	0.0231	AT1G65060
0.00	5.90	5.36	5.63	0.0305	AT1G28360
0.00	5.80	5.45	5.62	0.0198	AT1G44110
0.00	5.38	5.84	5.61	0.0261	AT3G29370
0.00	5.91	5.31	5.61	0.0340	AT5G61120
0.00	5.62	5.59	5.61	0.0017	AT3G48510
0.00	5.28	5.93	5.60	0.0369	AT4G30610
0.00	5.69	5.51	5.60	0.0102	AT5G53840
0.00	5.23	5.96	5.60	0.0415	AT5G14000

0.00	5.70	5.50	5.60	0.0114	AT5G62110
0.00	5.83	5.36	5.59	0.0267	AT2G34870
0.00	5.43	5.76	5.59	0.0188	AT3G53530
0.00	5.45	5.74	5.59	0.0165	AT5G47590
0.00	5.21	5.97	5.59	0.0432	AT4G29580
0.00	5.59	5.58	5.58	0.0006	AT1G79840
0.00	5.15	6.01	5.58	0.0490	AT2G42590
0.00	5.65	5.51	5.58	0.0080	AT3G10720
0.00	5.19	5.97	5.58	0.0444	AT5G66700
0.00	5.37	5.79	5.58	0.0239	AT1G20240
0.00	5.46	5.69	5.57	0.0131	AT2G02540
0.00	5.87	5.26	5.57	0.0349	AT2G17310
0.00	5.73	5.40	5.57	0.0189	AT5G12460
0.00	5.39	5.72	5.55	0.0189	AT1G67040
0.00	5.68	5.42	5.55	0.0149	AT2G45470
0.00	5.54	5.55	5.55	0.0006	AT3G54940
0.00	5.34	5.75	5.55	0.0235	AT5G39560
0.00	5.64	5.43	5.54	0.0121	AT3G58100
0.00	5.37	5.65	5.51	0.0162	AT1G44760
0.00	5.42	5.60	5.51	0.0104	AT1G58055
0.00	5.76	5.25	5.50	0.0295	AT5G07280
0.00	5.72	5.28	5.50	0.0255	AT1G65710
0.00	5.28	5.71	5.49	0.0249	AT3G60000
0.00	5.76	5.22	5.49	0.0313	AT2G36724
0.00	5.54	5.43	5.48	0.0064	AT5G57920
0.00	5.16	5.80	5.48	0.0371	AT5G07260
0.00	5.69	5.24	5.46	0.0262	AT1G27220
0.00	5.80	5.10	5.45	0.0408	AT3G29040
0.00	5.51	5.39	5.45	0.0070	AT3G01140
0.00	5.45	5.45	5.45	0.0000	AT1G51030
0.00	5.72	5.16	5.44	0.0327	AT1G04380
0.00	5.34	5.53	5.44	0.0111	AT2G23800
0.00	5.19	5.63	5.41	0.0259	AT1G77525
0.00	5.71	5.09	5.40	0.0365	AT5G17430
0.00	5.30	5.49	5.40	0.0112	AT1G74670
0.00	5.51	5.29	5.40	0.0130	AT1G71250
0.00	5.31	5.46	5.39	0.0089	AT5G18290
0.00	5.34	5.41	5.37	0.0041	AT5G23530
0.00	5.40	5.34	5.37	0.0036	AT5G22580
0.00	5.23	5.50	5.36	0.0160	AT1G23200
0.00	5.41	5.30	5.36	0.0065	AT5G09970
0.00	5.59	5.12	5.35	0.0279	AT1G04520
0.00	5.23	5.47	5.35	0.0143	AT5G07200
0.00	5.70	4.97	5.34	0.0435	AT5G47920
0.00	5.14	5.53	5.33	0.0233	AT1G12030
0.00	5.11	5.55	5.33	0.0263	AT4G09090
0.00	5.20	5.46	5.33	0.0155	AT1G44830
0.00	5.55	5.11	5.33	0.0263	AT2G30432
0.00	5.50	5.15	5.32	0.0209	AT1G05490
0.00	5.21	5.43	5.32	0.0132	AT4G01500
0.00	5.71	4.92	5.32	0.0472	AT2G22620
0.00	5.21	5.39	5.30	0.0108	AT3G02480
0.00	5.33	5.26	5.30	0.0042	AT5G66590
0.00	5.61	4.98	5.30	0.0378	AT2G20463
0.00	5.34	5.25	5.30	0.0054	AT5G43020
0.00	5.58	5.01	5.30	0.0342	AT5G66330
0.00	5.63	4.95	5.29	0.0409	AT2G42860
0.00	5.24	5.33	5.29	0.0054	AT1G70720
0.00	5.17	5.40	5.28	0.0139	AT5G37810

0.00	5.00	5.57	5.28	0.0343	AT5G63240
0.00	5.33	5.23	5.28	0.0060	AT5G51210
0.00	5.37	5.19	5.28	0.0109	AT3G20395
0.00	5.32	5.24	5.28	0.0048	AT3G27835
0.00	5.44	5.11	5.28	0.0199	AT5G45960
0.00	5.08	5.46	5.27	0.0229	AT5G03120
0.00	5.57	4.97	5.27	0.0362	AT4G01080
0.00	5.02	5.52	5.27	0.0302	AT5G40040
0.00	4.88	5.66	5.27	0.0470	AT3G48231
0.00	5.38	5.16	5.27	0.0133	AT4G02290
0.00	5.24	5.29	5.26	0.0030	AT1G01600
0.00	4.93	5.59	5.26	0.0399	AT1G24010
0.00	5.02	5.49	5.26	0.0285	AT4G27550
0.00	5.41	5.10	5.25	0.0188	AT1G78220
0.00	5.49	5.01	5.25	0.0291	AT4G00400
0.00	5.05	5.42	5.24	0.0225	AT4G05630
0.00	5.25	5.22	5.24	0.0018	AT1G64670
0.00	5.07	5.40	5.24	0.0201	AT5G02580
0.00	4.85	5.61	5.23	0.0462	AT3G17010
0.00	5.40	5.07	5.23	0.0201	AT1G68905
0.00	4.92	5.54	5.23	0.0377	AT3G42725
0.00	5.51	4.95	5.23	0.0341	AT3G23140
0.00	4.90	5.56	5.23	0.0401	AT2G01520
0.00	5.17	5.27	5.22	0.0061	AT3G21420
0.00	4.93	5.50	5.22	0.0348	AT3G25670
0.00	5.20	5.22	5.21	0.0012	AT5G56430
0.00	5.23	5.16	5.19	0.0043	AT1G02070
0.00	5.33	5.03	5.18	0.0184	AT4G17810
0.00	5.01	5.35	5.18	0.0209	AT4G24275
0.00	5.15	5.20	5.18	0.0031	AT3G14760
0.00	5.07	5.27	5.17	0.0123	AT5G55132
0.00	5.24	5.10	5.17	0.0086	AT5G09730
0.00	5.22	5.11	5.17	0.0068	AT1G12090
0.00	5.35	4.96	5.15	0.0241	AT1G13755
0.00	4.99	5.32	5.15	0.0204	AT4G13790
0.00	5.31	4.98	5.15	0.0204	AT2G40170
0.00	5.39	4.91	5.15	0.0296	AT4G28310
0.00	5.23	5.05	5.14	0.0111	AT5G42800
0.00	5.17	5.10	5.13	0.0043	AT5G50360
0.00	5.41	4.86	5.13	0.0341	AT3G06840
0.00	5.23	5.04	5.13	0.0118	AT3G21410
0.00	4.80	5.43	5.12	0.0392	AT5G19175
0.00	5.09	5.13	5.11	0.0025	AT1G30920
0.00	5.49	4.73	5.11	0.0473	AT5G32619
0.00	4.97	5.25	5.11	0.0174	AT2G13610
0.00	4.80	5.40	5.10	0.0374	AT4G12900
0.00	4.98	5.23	5.10	0.0156	AT4G29285
0.00	4.96	5.24	5.10	0.0175	AT4G02330
0.00	4.89	5.30	5.09	0.0256	AT1G01110
0.00	5.10	5.08	5.09	0.0013	AT5G42223
0.00	4.91	5.25	5.08	0.0213	AT3G14190
0.00	4.89	5.27	5.08	0.0238	AT5G44680
0.00	5.34	4.81	5.08	0.0332	AT4G14120
0.00	5.28	4.88	5.08	0.0251	AT4G34510
0.00	5.21	4.94	5.08	0.0169	AT1G68460
0.00	4.99	5.14	5.06	0.0094	AT5G67110
0.00	5.32	4.81	5.06	0.0320	AT1G30795
0.00	5.26	4.85	5.06	0.0258	AT2G03770
0.00	4.97	5.14	5.05	0.0107	AT1G72970

0.00	4.69	5.41	5.05	0.0453	AT4G14330
0.00	4.68	5.41	5.05	0.0460	AT3G56520
0.00	5.21	4.88	5.04	0.0208	AT5G60970
0.00	5.06	5.03	5.04	0.0019	AT5G39860
0.00	5.18	4.89	5.04	0.0183	AT1G79060
0.00	5.08	4.97	5.03	0.0070	AT1G50110
0.00	4.91	5.14	5.02	0.0146	AT4G15480
0.00	5.02	5.01	5.01	0.0006	AT2G42540
0.00	5.11	4.91	5.01	0.0127	AT5G05220
0.00	5.16	4.84	5.00	0.0204	AT4G33220
0.00	4.76	5.23	4.99	0.0299	AT3G21620
0.00	4.95	5.00	4.98	0.0032	AT3G17760
0.00	4.64	5.31	4.97	0.0428	AT1G61255
0.00	4.71	5.23	4.97	0.0333	AT5G62170
0.00	4.97	4.97	4.97	0.0000	AT4G29150
0.00	4.86	5.06	4.96	0.0128	AT3G63430
0.00	4.96	4.96	4.96	0.0000	AT5G66580
0.00	5.00	4.92	4.96	0.0051	AT4G00180
0.00	5.29	4.62	4.96	0.0430	AT3G21840
0.00	5.01	4.90	4.95	0.0071	AT5G13140
0.00	4.96	4.94	4.95	0.0013	AT3G25980
0.00	4.93	4.97	4.95	0.0026	AT1G18250
0.00	5.24	4.66	4.95	0.0373	AT1G23410
0.00	4.84	5.05	4.95	0.0135	AT4G29360
0.00	4.84	5.04	4.94	0.0129	AT5G17340
0.00	4.81	5.06	4.94	0.0161	AT2G14620
0.00	4.63	5.24	4.93	0.0393	AT1G53282
0.00	5.13	4.71	4.92	0.0272	AT1G52690
0.00	5.25	4.58	4.91	0.0433	AT3G21500
0.00	4.67	5.16	4.91	0.0317	AT4G27330
0.00	4.69	5.14	4.91	0.0291	AT3G01325
0.00	5.06	4.77	4.91	0.0188	AT4G10490
0.00	4.62	5.20	4.91	0.0376	AT3G18360
0.00	5.25	4.56	4.91	0.0447	AT5G42230
0.00	4.71	5.09	4.90	0.0247	AT2G31980
0.00	4.63	5.17	4.90	0.0350	AT2G18500
0.00	5.12	4.67	4.89	0.0292	AT4G37810
0.00	5.11	4.66	4.89	0.0293	AT4G37950
0.00	5.16	4.61	4.88	0.0358	AT2G37720
0.00	4.81	4.96	4.88	0.0098	AT5G59040
0.00	4.97	4.80	4.88	0.0111	AT5G56120
0.00	4.93	4.83	4.88	0.0065	AT5G41720
0.00	5.17	4.59	4.88	0.0378	AT5G46795
0.00	4.92	4.84	4.88	0.0052	AT1G24590
0.00	4.51	5.24	4.87	0.0476	AT1G28030
0.00	4.93	4.82	4.87	0.0072	AT3G48540
0.00	5.23	4.51	4.87	0.0470	AT1G20500
0.00	5.02	4.71	4.87	0.0203	AT5G02890
0.00	4.83	4.90	4.87	0.0046	AT5G06020
0.00	5.11	4.62	4.87	0.0320	AT1G05260
0.00	4.53	5.19	4.86	0.0432	AT4G19940
0.00	4.67	5.05	4.86	0.0249	AT5G18030
0.00	5.12	4.59	4.86	0.0347	AT1G70270
0.00	4.89	4.81	4.85	0.0053	AT5G16250
0.00	4.49	5.19	4.84	0.0460	AT1G23590
0.00	4.94	4.73	4.84	0.0138	AT3G03820
0.00	5.21	4.46	4.84	0.0493	AT3G16670
0.00	5.15	4.52	4.84	0.0414	AT4G13968
0.00	4.62	5.04	4.83	0.0277	AT3G02640

0.00	4.88	4.78	4.83	0.0066	AT5G20240
0.00	5.17	4.47	4.82	0.0461	AT5G54400
0.00	4.93	4.71	4.82	0.0145	AT1G08560
0.00	5.16	4.47	4.81	0.0455	AT5G60630
0.00	4.61	5.01	4.81	0.0265	AT5G62850
0.00	4.90	4.72	4.81	0.0119	AT4G32717
0.00	5.01	4.60	4.81	0.0271	AT3G17520
0.00	4.69	4.91	4.80	0.0146	AT5G14690
0.00	4.88	4.71	4.80	0.0113	AT4G25830
0.00	4.44	5.14	4.79	0.0464	AT2G45050
0.00	4.70	4.88	4.79	0.0120	AT5G56320
0.00	4.53	5.04	4.79	0.0339	AT3G06220
0.00	4.99	4.57	4.78	0.0280	AT1G05540
0.00	4.67	4.88	4.78	0.0140	AT5G23940
0.00	4.87	4.68	4.77	0.0127	AT3G56220
0.00	4.47	5.07	4.77	0.0400	AT4G32540
0.00	4.59	4.95	4.77	0.0240	AT1G67265
0.00	4.87	4.65	4.76	0.0147	AT1G13607
0.00	4.56	4.95	4.76	0.0261	AT4G09500
0.00	5.12	4.39	4.76	0.0488	AT4G35670
0.00	4.93	4.58	4.75	0.0234	AT1G49190
0.00	5.01	4.49	4.75	0.0348	AT5G06150
0.00	4.48	5.01	4.75	0.0355	AT3G46320
0.00	4.64	4.85	4.74	0.0141	AT3G27810
0.00	4.44	5.04	4.74	0.0402	AT5G37940
0.00	4.68	4.80	4.74	0.0081	AT3G56370
0.00	4.56	4.91	4.73	0.0235	AT5G48360
0.00	4.89	4.55	4.72	0.0229	AT5G17220
0.00	4.94	4.49	4.72	0.0304	AT3G61172
0.00	4.48	4.95	4.71	0.0317	AT1G62340
0.00	4.74	4.68	4.71	0.0041	AT1G73550
0.00	5.00	4.42	4.71	0.0391	AT1G73325
0.00	4.46	4.94	4.70	0.0325	AT3G51290
0.00	4.70	4.69	4.70	0.0007	AT2G16630
0.00	4.80	4.59	4.70	0.0142	AT2G05790
0.00	4.38	5.01	4.69	0.0426	AT3G48350
0.00	4.43	4.94	4.69	0.0346	AT3G48550
0.00	4.67	4.71	4.69	0.0027	AT1G60590
0.00	4.72	4.63	4.68	0.0061	AT4G39070
0.00	4.60	4.74	4.67	0.0095	AT5G18010
0.00	4.42	4.92	4.67	0.0340	AT1G54840
0.00	4.86	4.48	4.67	0.0259	AT3G11160
0.00	4.50	4.84	4.67	0.0232	AT5G18050
0.00	4.38	4.95	4.67	0.0388	AT2G22345
0.00	4.60	4.74	4.67	0.0095	AT4G16165
0.00	4.73	4.60	4.66	0.0089	AT3G25050
0.00	4.39	4.93	4.66	0.0368	AT4G39650
0.00	4.97	4.34	4.65	0.0430	AT1G13230
0.00	4.92	4.39	4.65	0.0362	AT1G03710
0.00	4.29	5.01	4.65	0.0492	AT5G15580
0.00	4.74	4.56	4.65	0.0123	AT4G36350
0.00	4.75	4.55	4.65	0.0137	AT1G24650
0.00	4.38	4.91	4.65	0.0363	AT1G53070
0.00	4.69	4.59	4.64	0.0069	AT1G02730
0.00	4.57	4.71	4.64	0.0096	AT2G40670
0.00	4.86	4.42	4.64	0.0302	AT1G75890
0.00	4.68	4.59	4.63	0.0062	AT1G32780
0.00	4.70	4.57	4.63	0.0089	AT4G38000
0.00	4.91	4.36	4.63	0.0377	AT4G27890

0.00	4.59	4.66	4.63	0.0048	AT3G28290
0.00	4.72	4.54	4.63	0.0124	AT3G51280
0.00	4.59	4.65	4.62	0.0041	AT1G14900
0.00	4.66	4.57	4.62	0.0062	AT1G65090
0.00	4.60	4.64	4.62	0.0028	AT5G66230
0.00	4.44	4.79	4.62	0.0241	AT3G01490
0.00	4.95	4.29	4.62	0.0454	AT2G34210
0.00	4.59	4.63	4.61	0.0028	AT1G77520
0.00	4.87	4.33	4.60	0.0373	AT2G26695
0.00	4.32	4.88	4.60	0.0387	AT2G37700
0.00	4.50	4.70	4.60	0.0138	AT2G16580
0.00	4.36	4.82	4.59	0.0319	AT3G09950
0.00	4.75	4.43	4.59	0.0222	AT5G59870
0.00	4.46	4.71	4.59	0.0174	AT4G37980
0.00	4.26	4.91	4.59	0.0451	AT1G68450
0.00	4.28	4.89	4.58	0.0423	AT5G36710
0.00	4.50	4.66	4.58	0.0111	AT1G29195
0.00	4.67	4.49	4.58	0.0125	AT5G17500
0.00	4.90	4.26	4.58	0.0444	AT2G21420
0.00	4.68	4.46	4.57	0.0153	AT4G18570
0.00	4.35	4.79	4.57	0.0306	AT1G29490
0.00	4.86	4.25	4.56	0.0426	AT4G13840
0.00	4.42	4.68	4.55	0.0182	AT1G47395
0.00	4.32	4.78	4.55	0.0322	AT5G51560
0.00	4.60	4.50	4.55	0.0070	AT2G14900
0.00	4.33	4.74	4.54	0.0288	AT5G38170
0.00	4.45	4.61	4.53	0.0112	AT5G19060
0.00	5.17	3.89	4.53	0.0347	AT1G09510
0.00	4.27	4.79	4.53	0.0365	AT3G27710
0.00	4.43	4.63	4.53	0.0141	AT4G25480
0.00	4.22	4.83	4.52	0.0428	AT2G03190
0.00	4.50	4.54	4.52	0.0028	AT5G18080
0.00	4.46	4.58	4.52	0.0085	AT5G13400
0.00	4.39	4.64	4.51	0.0176	AT5G02540
0.00	4.55	4.46	4.51	0.0064	AT1G70710
0.00	4.81	4.20	4.51	0.0430	AT1G63100
0.00	4.25	4.75	4.50	0.0353	AT2G22000
0.00	4.72	4.28	4.50	0.0311	AT3G17480
0.00	4.45	4.55	4.50	0.0071	AT1G80370
0.00	4.68	4.30	4.49	0.0269	AT5G55730
0.00	4.76	4.22	4.49	0.0382	AT3G01324
0.00	4.23	4.73	4.48	0.0355	AT5G11130
0.00	4.48	4.47	4.47	0.0007	AT5G18060
0.00	4.59	4.35	4.47	0.0171	AT5G15000
0.00	4.21	4.73	4.47	0.0370	AT3G03130
0.00	4.20	4.74	4.47	0.0384	AT4G02800
0.00	4.75	4.18	4.47	0.0406	AT4G23290
0.00	4.17	4.76	4.46	0.0420	AT3G42473
0.00	4.51	4.41	4.46	0.0071	AT1G76500
0.00	4.54	4.37	4.45	0.0121	AT5G42910
0.00	4.11	4.79	4.45	0.0485	AT4G12617
0.00	4.78	4.12	4.45	0.0471	AT2G03290
0.00	4.35	4.54	4.45	0.0136	AT4G17280
0.00	4.21	4.65	4.43	0.0316	AT5G46940
0.00	4.25	4.59	4.42	0.0245	AT3G11520
0.00	4.75	4.08	4.42	0.0482	AT4G37490
0.00	4.68	4.15	4.42	0.0382	AT1G27250
0.00	4.10	4.72	4.41	0.0447	AT1G23570
0.00	4.17	4.64	4.41	0.0339	AT3G61177

0.00	4.70	4.10	4.40	0.0433	AT2G16270
0.00	4.67	4.13	4.40	0.0390	AT2G03933
0.00	4.08	4.72	4.40	0.0462	AT3G26010
0.00	4.17	4.61	4.39	0.0319	AT2G25270
0.00	4.33	4.43	4.38	0.0073	AT3G10510
0.00	4.06	4.70	4.38	0.0464	AT5G55340
0.00	4.23	4.53	4.38	0.0218	AT3G05180
0.00	4.38	4.38	4.38	0.0000	AT3G04300
0.00	4.38	4.36	4.37	0.0015	AT3G63200
0.00	4.41	4.33	4.37	0.0058	AT5G35550
0.00	4.20	4.53	4.36	0.0241	AT2G17620
0.00	4.32	4.39	4.36	0.0051	AT5G19172
0.00	4.10	4.61	4.36	0.0372	AT5G41900
0.00	4.07	4.63	4.35	0.0409	AT3G48240
0.00	4.63	4.07	4.35	0.0409	AT4G12910
0.00	4.16	4.53	4.34	0.0271	AT3G11825
0.00	4.23	4.46	4.34	0.0168	AT5G56369
0.00	4.50	4.18	4.34	0.0235	AT3G61550
0.00	4.65	4.03	4.34	0.0454	AT4G12140
0.00	4.35	4.32	4.34	0.0022	AT1G34245
0.00	4.56	4.10	4.33	0.0338	AT2G45130
0.00	4.49	4.18	4.33	0.0228	AT5G06030
0.00	4.17	4.47	4.32	0.0221	AT3G20820
0.00	4.00	4.64	4.32	0.0471	AT2G33690
0.00	4.39	4.25	4.32	0.0103	AT3G21310
0.00	4.15	4.48	4.32	0.0243	AT5G23400
0.00	4.43	4.19	4.31	0.0177	AT3G54060
0.00	4.19	4.43	4.31	0.0177	AT3G03450
0.00	3.97	4.62	4.30	0.0481	AT5G65390
0.00	4.05	4.54	4.29	0.0363	AT2G33850
0.00	4.45	4.14	4.29	0.0230	AT1G47610
0.00	4.11	4.46	4.28	0.0260	AT4G19980
0.00	4.14	4.42	4.28	0.0208	AT4G29650
0.00	4.07	4.49	4.28	0.0312	AT1G07610
0.00	4.11	4.45	4.28	0.0253	AT4G33270
0.00	3.97	4.58	4.28	0.0453	AT3G11180
0.00	4.52	4.03	4.28	0.0364	AT5G22940
0.00	4.30	4.24	4.27	0.0045	AT2G36050
0.00	3.95	4.58	4.27	0.0469	AT3G12470
0.00	4.01	4.52	4.27	0.0380	AT2G14365
0.00	4.11	4.41	4.26	0.0224	AT3G20260
0.00	4.21	4.30	4.26	0.0067	AT5G64080
0.00	4.08	4.44	4.26	0.0269	AT2G41230
0.00	4.39	4.08	4.23	0.0233	AT4G24973
0.00	4.51	3.93	4.22	0.0437	AT4G39630
0.00	4.35	4.08	4.21	0.0204	AT3G02110
0.00	4.35	4.07	4.21	0.0212	AT4G00210
0.00	3.92	4.50	4.21	0.0438	AT5G51480
0.00	4.01	4.40	4.20	0.0295	AT2G20515
0.00	4.42	3.99	4.20	0.0325	AT2G23260
0.00	4.20	4.20	4.20	0.0000	AT3G61175
0.00	4.13	4.26	4.20	0.0099	AT3G25290
0.00	4.03	4.36	4.19	0.0250	AT5G40630
0.00	4.01	4.37	4.19	0.0273	AT5G13060
0.00	4.10	4.27	4.19	0.0129	AT2G32765
0.00	4.60	3.77	4.19	0.0267	AT2G36190
0.00	4.18	4.18	4.18	0.0000	AT5G63085
0.00	4.13	4.20	4.16	0.0053	AT2G17723
0.00	4.43	3.90	4.16	0.0405	AT3G46490

0.00	4.15	4.17	4.16	0.0015	AT5G57123
0.00	4.24	4.07	4.16	0.0130	AT1G18370
0.00	3.97	4.34	4.15	0.0283	AT4G33550
0.00	4.17	4.14	4.15	0.0023	AT1G78500
0.00	4.36	3.94	4.15	0.0322	AT2G36270
0.00	4.39	3.91	4.15	0.0368	AT1G34355
0.00	4.03	4.26	4.15	0.0177	AT3G50620
0.00	4.31	3.99	4.15	0.0245	AT1G79720
0.00	3.97	4.32	4.14	0.0269	AT3G55646
0.00	4.07	4.22	4.14	0.0115	AT4G29280
0.00	4.17	4.10	4.13	0.0054	AT3G09930
0.00	4.01	4.25	4.13	0.0185	AT4G31890
0.00	4.04	4.22	4.13	0.0139	AT2G36560
0.00	4.13	4.12	4.13	0.0008	AT3G10590
0.00	3.86	4.39	4.13	0.0408	AT2G45400
0.00	4.17	4.07	4.12	0.0077	AT5G50750
0.00	4.20	4.04	4.12	0.0124	AT3G28860
0.00	4.06	4.17	4.11	0.0085	AT5G65730
0.00	3.85	4.38	4.11	0.0409	AT4G37800
0.00	4.23	3.99	4.11	0.0186	AT3G50740
0.00	4.35	3.87	4.11	0.0371	AT5G23650
0.00	4.21	4.00	4.11	0.0163	AT4G27260
0.00	4.16	4.05	4.10	0.0085	AT1G49475
0.00	4.22	3.99	4.10	0.0178	AT3G55660
0.00	4.05	4.14	4.10	0.0070	AT1G49430
0.00	3.86	4.33	4.09	0.0365	AT4G16230
0.00	4.11	4.08	4.09	0.0023	AT3G54180
0.00	4.07	4.11	4.09	0.0031	AT1G05440
0.00	4.41	3.77	4.09	0.0497	AT4G18310
0.00	4.00	4.17	4.09	0.0132	AT3G21945
0.00	3.99	4.18	4.09	0.0148	AT2G37390
0.00	3.79	4.38	4.09	0.0459	AT5G18430
0.00	4.22	3.94	4.08	0.0218	AT5G50490
0.00	3.86	4.29	4.07	0.0336	AT3G02120
0.00	4.36	3.79	4.07	0.0445	AT2G30730
0.00	3.79	4.35	4.07	0.0437	AT1G34640
0.00	3.90	4.24	4.07	0.0266	AT1G19970
0.00	3.96	4.17	4.07	0.0164	AT4G16447
0.00	3.77	4.34	4.06	0.0447	AT4G11810
0.00	4.18	3.92	4.05	0.0204	AT3G50020
0.00	3.84	4.26	4.05	0.0330	AT2G32990
0.00	4.27	3.82	4.05	0.0354	AT4G32980
0.00	4.12	3.96	4.04	0.0126	AT1G68907
0.00	4.05	4.03	4.04	0.0016	AT4G18920
0.00	3.93	4.14	4.04	0.0166	AT1G10190
0.00	4.24	3.83	4.03	0.0323	AT4G13080
0.00	4.16	3.91	4.03	0.0197	AT1G33811
0.00	4.22	3.83	4.03	0.0308	AT2G22200
0.00	3.82	4.23	4.03	0.0324	AT3G59890
0.00	4.04	4.01	4.02	0.0024	AT1G17560
0.00	3.80	4.23	4.01	0.0341	AT4G03965
0.00	3.92	4.11	4.01	0.0151	AT5G57780
0.00	3.90	4.13	4.01	0.0182	AT5G48480
0.00	3.96	4.07	4.01	0.0087	AT5G18020
0.00	4.19	3.83	4.01	0.0286	AT1G23500
0.00	3.71	4.32	4.01	0.0483	AT4G23496
0.00	4.02	4.00	4.01	0.0016	AT5G08730
0.00	3.99	4.03	4.01	0.0032	AT3G55710
0.00	4.05	3.96	4.01	0.0072	AT5G55520

0.00	4.05	3.95	4.00	0.0080	AT5G44640
0.00	4.13	3.87	4.00	0.0207	AT2G43870
0.00	4.08	3.91	4.00	0.0135	AT2G37300
0.00	3.74	4.25	4.00	0.0406	AT1G22330
0.00	4.15	3.83	3.99	0.0255	AT4G32890
0.00	3.68	4.30	3.99	0.0494	AT3G45140
0.00	3.89	4.08	3.99	0.0152	AT5G01015
0.00	4.03	3.94	3.98	0.0072	AT1G48990
0.00	3.94	4.02	3.98	0.0064	AT1G65900
0.00	4.27	3.68	3.98	0.0472	AT3G17680
0.00	4.07	3.89	3.98	0.0144	AT2G47880
0.00	4.11	3.84	3.98	0.0216	AT5G03860
0.00	3.67	4.28	3.98	0.0488	AT5G62340
0.00	3.73	4.21	3.97	0.0384	AT4G29570
0.00	4.17	3.76	3.97	0.0329	AT2G18420
0.00	4.12	3.82	3.97	0.0240	AT4G02700
0.00	4.00	3.94	3.97	0.0048	AT1G58270
0.00	4.06	3.87	3.97	0.0153	AT4G10630
0.00	3.74	4.17	3.96	0.0346	AT1G29440
0.00	3.75	4.14	3.95	0.0314	AT3G43083
0.00	4.05	3.83	3.94	0.0178	AT4G22390
0.00	3.91	3.98	3.94	0.0056	AT4G14770
0.00	3.75	4.13	3.94	0.0307	AT3G15357
0.00	3.86	4.02	3.94	0.0129	AT5G62280
0.00	3.81	4.07	3.94	0.0210	AT5G26230
0.00	3.69	4.17	3.93	0.0388	AT5G66780
0.00	3.93	3.93	3.93	0.0000	AT2G29290
0.00	3.78	4.07	3.93	0.0235	AT1G72670
0.00	4.03	3.82	3.92	0.0170	AT5G03870
0.00	3.66	4.18	3.92	0.0422	AT3G61898
0.00	3.69	4.14	3.92	0.0365	AT3G46940
0.00	3.62	4.21	3.92	0.0479	AT5G38160
0.00	3.77	4.07	3.92	0.0243	AT4G03010
0.00	3.67	4.16	3.92	0.0398	AT5G04150
0.00	4.11	3.72	3.91	0.0317	AT5G45850
0.00	3.70	4.13	3.91	0.0349	AT3G09870
0.00	4.11	3.71	3.91	0.0325	AT2G44000
0.00	3.75	4.07	3.91	0.0260	AT3G13175
0.00	3.97	3.85	3.91	0.0098	AT5G58500
0.00	4.19	3.62	3.90	0.0464	AT5G26850
0.00	3.83	3.94	3.88	0.0090	AT1G72210
0.00	3.99	3.77	3.88	0.0180	AT5G44950
0.00	3.68	4.06	3.87	0.0312	AT4G11290
0.00	3.67	4.07	3.87	0.0329	AT4G20240
0.00	3.68	4.05	3.87	0.0304	AT3G25100
0.00	3.79	3.94	3.87	0.0124	AT1G27030
0.00	3.84	3.89	3.86	0.0041	AT4G29610
0.00	3.98	3.75	3.86	0.0189	AT1G23600
0.00	3.94	3.78	3.86	0.0132	AT4G24130
0.00	3.91	3.80	3.86	0.0091	AT1G56580
0.00	3.79	3.93	3.86	0.0115	AT2G26180
0.00	3.70	4.01	3.85	0.0256	AT5G39240
0.00	3.92	3.79	3.85	0.0107	AT1G48940
0.00	3.88	3.82	3.85	0.0050	AT2G38760
0.00	3.80	3.90	3.85	0.0083	AT4G28430
0.00	3.84	3.86	3.85	0.0017	AT4G23870
0.00	3.94	3.76	3.85	0.0149	AT3G61490
0.00	4.01	3.69	3.85	0.0264	AT2G10535
0.00	3.78	3.91	3.84	0.0108	AT2G04170

0.00	3.85	3.82	3.84	0.0025	AT3G01960
0.00	3.62	4.05	3.83	0.0357	AT2G18890
0.00	3.78	3.88	3.83	0.0083	AT5G45650
0.00	3.94	3.72	3.83	0.0183	AT4G35690
0.00	4.01	3.65	3.83	0.0299	AT2G45420
0.00	3.61	4.04	3.83	0.0357	AT4G31290
0.00	4.10	3.55	3.83	0.0457	AT5G08505
0.00	3.89	3.75	3.82	0.0117	AT2G36570
0.00	3.96	3.68	3.82	0.0233	AT2G37740
0.00	3.98	3.65	3.82	0.0275	AT5G50760
0.00	3.90	3.72	3.81	0.0150	AT1G12010
0.00	3.63	3.99	3.81	0.0301	AT5G46540
0.00	3.94	3.66	3.80	0.0234	AT1G07795
0.00	3.52	4.08	3.80	0.0468	AT5G51600
0.00	4.09	3.51	3.80	0.0485	AT5G59105
0.00	3.99	3.60	3.80	0.0327	AT5G39160
0.00	3.80	3.79	3.80	0.0008	AT5G63140
0.00	4.01	3.58	3.79	0.0360	AT3G52810
0.00	3.71	3.86	3.79	0.0126	AT3G09190
0.00	3.63	3.94	3.79	0.0261	AT3G54710
0.00	3.53	4.04	3.79	0.0428	AT3G52630
0.00	3.60	3.97	3.79	0.0311	AT5G24790
0.00	3.59	3.98	3.78	0.0328	AT3G58070
0.00	3.72	3.84	3.78	0.0101	AT2G42460
0.00	3.57	4.00	3.78	0.0361	AT5G02550
0.00	3.78	3.78	3.78	0.0000	AT2G29980
0.00	3.62	3.94	3.78	0.0269	AT4G34220
0.00	3.75	3.81	3.78	0.0051	AT2G02100
0.00	3.93	3.63	3.78	0.0252	AT4G10895
0.00	3.72	3.83	3.78	0.0093	AT2G19910
0.00	3.65	3.90	3.77	0.0211	AT4G24260
0.00	3.48	4.06	3.77	0.0489	AT2G27535
0.00	3.73	3.81	3.77	0.0068	AT5G59970
0.00	3.77	3.77	3.77	0.0000	AT1G52190
0.00	3.73	3.81	3.77	0.0068	AT3G28007
0.00	3.47	4.06	3.77	0.0498	AT1G26770
0.00	3.88	3.65	3.77	0.0194	AT4G37840
0.00	3.95	3.58	3.76	0.0313	AT1G23520
0.00	3.66	3.86	3.76	0.0169	AT5G64330
0.00	3.55	3.97	3.76	0.0355	AT4G21270
0.00	3.62	3.90	3.76	0.0237	AT1G53140
0.00	3.79	3.73	3.76	0.0051	AT4G23750
0.00	3.67	3.84	3.76	0.0144	AT2G36026
0.00	4.00	3.51	3.76	0.0415	AT1G66290
0.00	3.75	3.76	3.75	0.0008	AT3G55120
0.00	3.83	3.66	3.75	0.0144	AT1G75710
0.00	3.55	3.95	3.75	0.0339	AT1G66430
0.00	3.46	4.02	3.74	0.0476	AT4G17160
0.00	3.73	3.75	3.74	0.0017	AT1G72810
0.00	3.56	3.91	3.73	0.0298	AT3G18050
0.00	3.81	3.66	3.73	0.0128	AT4G33260
0.00	3.88	3.58	3.73	0.0256	AT2G38170
0.00	3.51	3.95	3.73	0.0375	AT5G24580
0.00	3.76	3.70	3.73	0.0051	AT4G12880
0.00	3.65	3.80	3.73	0.0128	AT1G80280
0.00	3.48	3.97	3.73	0.0418	AT3G05190
0.00	3.76	3.68	3.72	0.0068	AT3G43720
0.00	3.82	3.62	3.72	0.0171	AT5G67270
0.00	3.80	3.64	3.72	0.0137	AT1G27461

0.00	3.58	3.84	3.71	0.0223	AT5G03960
0.00	3.79	3.62	3.71	0.0146	AT3G23890
0.00	3.96	3.45	3.70	0.0437	AT5G59320
0.00	3.74	3.67	3.70	0.0060	AT3G58780
0.00	3.75	3.64	3.70	0.0095	AT1G31760
0.00	3.73	3.66	3.69	0.0060	AT1G01250
0.00	3.71	3.67	3.69	0.0035	AT1G17140
0.00	3.51	3.87	3.69	0.0310	AT4G15820
0.00	3.74	3.63	3.69	0.0095	AT1G48480
0.00	3.70	3.68	3.69	0.0017	AT2G21650
0.00	3.59	3.78	3.68	0.0164	AT1G19460
0.00	3.51	3.85	3.68	0.0294	AT4G11310
0.00	3.94	3.41	3.68	0.0458	AT3G15270
0.00	3.45	3.90	3.68	0.0389	AT1G15757
0.00	3.65	3.70	3.67	0.0043	AT3G10520
0.00	3.55	3.79	3.67	0.0208	AT3G02210
0.00	3.71	3.61	3.66	0.0087	AT4G39000
0.00	3.53	3.79	3.66	0.0226	AT4G35620
0.00	3.70	3.62	3.66	0.0070	AT1G71695
0.00	3.73	3.59	3.66	0.0122	AT1G12080
0.00	3.72	3.60	3.66	0.0104	AT2G07170
0.00	3.89	3.42	3.66	0.0409	AT4G38660
0.00	3.72	3.59	3.66	0.0113	AT4G39590
0.00	3.57	3.74	3.66	0.0148	AT1G01610
0.00	3.53	3.78	3.65	0.0218	AT5G63225
0.00	3.67	3.64	3.65	0.0026	AT3G59220
0.00	3.65	3.65	3.65	0.0000	AT1G45010
0.00	3.58	3.73	3.65	0.0131	AT2G46680
0.00	3.58	3.72	3.65	0.0122	AT1G61070
0.00	3.78	3.52	3.65	0.0227	AT2G15440
0.00	3.54	3.76	3.65	0.0192	AT5G04160
0.00	3.36	3.93	3.64	0.0497	AT5G42232
0.00	3.50	3.79	3.64	0.0253	AT3G03341
0.00	3.51	3.77	3.64	0.0227	AT5G10100
0.00	3.91	3.36	3.64	0.0481	AT3G54410
0.00	3.62	3.63	3.63	0.0009	AT2G28950
0.00	3.83	3.43	3.63	0.0350	AT4G19905
0.00	3.50	3.75	3.62	0.0219	AT3G57510
0.00	3.41	3.84	3.62	0.0377	AT2G44450
0.00	3.58	3.67	3.62	0.0079	AT1G07473
0.00	3.44	3.81	3.62	0.0325	AT4G34790
0.00	3.40	3.85	3.62	0.0395	AT5G58830
0.00	3.64	3.60	3.62	0.0035	AT3G24340
0.00	3.64	3.60	3.62	0.0035	AT4G02810
0.00	3.38	3.85	3.62	0.0413	AT3G26742
0.00	3.77	3.47	3.62	0.0264	AT1G04670
0.00	3.80	3.43	3.62	0.0326	AT1G76310
0.00	3.68	3.55	3.61	0.0114	AT4G05475
0.00	3.39	3.82	3.60	0.0379	AT4G26770
0.00	3.64	3.57	3.60	0.0062	AT4G14310
0.00	3.54	3.66	3.60	0.0106	AT3G22820
0.00	3.60	3.58	3.59	0.0018	AT4G23490
0.00	3.75	3.42	3.59	0.0293	AT1G65180
0.00	3.46	3.70	3.58	0.0213	AT4G00220
0.00	3.59	3.58	3.58	0.0009	AT4G12690
0.00	3.71	3.46	3.58	0.0222	AT2G45570
0.00	3.64	3.53	3.58	0.0098	AT3G05480
0.00	3.67	3.50	3.58	0.0151	AT4G36700
0.00	3.67	3.50	3.58	0.0151	AT2G26250

0.00	3.79	3.37	3.58	0.0373	AT2G46720
0.00	3.55	3.59	3.57	0.0036	AT4G36380
0.00	3.78	3.36	3.57	0.0374	AT4G26660
0.00	3.34	3.79	3.57	0.0401	AT4G16980
0.00	3.68	3.45	3.57	0.0205	AT1G01370
0.00	3.58	3.54	3.56	0.0036	AT2G16750
0.00	3.54	3.57	3.56	0.0027	AT1G26540
0.00	3.42	3.69	3.56	0.0242	AT1G14430
0.00	3.43	3.67	3.55	0.0215	AT5G14410
0.00	3.39	3.71	3.55	0.0287	AT5G43990
0.00	3.55	3.55	3.55	0.0000	AT5G47530
0.00	3.61	3.49	3.55	0.0108	AT3G49670
0.00	3.41	3.67	3.54	0.0234	AT3G51230
0.00	3.47	3.61	3.54	0.0126	AT5G03260
0.00	3.61	3.47	3.54	0.0126	AT4G16515
0.00	3.68	3.38	3.53	0.0270	AT1G74420
0.00	3.55	3.50	3.53	0.0045	AT2G34190
0.00	3.25	3.80	3.52	0.0496	AT1G54820
0.00	3.66	3.38	3.52	0.0253	AT5G39130
0.00	3.29	3.75	3.52	0.0415	AT5G66005
0.00	3.66	3.36	3.51	0.0272	AT5G39520
0.00	3.60	3.42	3.51	0.0163	AT1G20590
0.00	3.43	3.59	3.51	0.0145	AT5G10400
0.00	3.51	3.49	3.50	0.0018	AT1G08465
0.00	3.57	3.43	3.50	0.0127	AT2G28470
0.00	3.65	3.35	3.50	0.0273	AT4G33280
0.00	3.37	3.63	3.50	0.0236	AT1G58360
0.00	3.50	3.49	3.50	0.0009	AT4G28680
0.00	3.23	3.77	3.50	0.0490	AT2G16210
0.00	3.70	3.30	3.50	0.0363	AT5G60220
0.00	3.44	3.55	3.50	0.0100	AT5G26120
0.00	3.23	3.76	3.49	0.0482	AT5G55480
0.00	3.57	3.42	3.49	0.0137	AT5G11550
0.00	3.46	3.53	3.49	0.0064	AT3G07010
0.00	3.35	3.64	3.49	0.0264	AT1G27770
0.00	3.42	3.56	3.49	0.0128	AT5G65010
0.00	3.67	3.31	3.49	0.0328	AT3G53380
0.00	3.64	3.33	3.48	0.0283	AT4G05190
0.00	3.52	3.44	3.48	0.0073	AT1G02205
0.00	3.47	3.49	3.48	0.0018	AT4G34250
0.00	3.60	3.36	3.48	0.0219	AT3G18850
0.00	3.34	3.61	3.47	0.0247	AT1G31290
0.00	3.42	3.53	3.47	0.0101	AT3G29030
0.00	3.24	3.70	3.47	0.0421	AT1G19830
0.00	3.40	3.54	3.47	0.0128	AT2G35950
0.00	3.66	3.27	3.47	0.0358	AT3G62070
0.00	3.50	3.44	3.47	0.0055	AT1G24130
0.00	3.22	3.71	3.47	0.0449	AT2G23200
0.00	3.37	3.55	3.46	0.0166	AT3G50890
0.00	3.55	3.36	3.46	0.0175	AT5G24420
0.00	3.51	3.40	3.45	0.0101	AT5G56510
0.00	3.47	3.44	3.45	0.0028	AT4G28720
0.00	3.31	3.60	3.45	0.0267	AT3G04290
0.00	3.58	3.33	3.45	0.0230	AT2G45970
0.00	3.58	3.32	3.45	0.0240	AT5G09210
0.00	3.41	3.49	3.45	0.0074	AT5G05810
0.00	3.26	3.64	3.45	0.0350	AT1G29500
0.00	3.48	3.41	3.45	0.0065	AT5G20790
0.00	3.55	3.34	3.44	0.0194	AT4G35190

0.00	3.51	3.37	3.44	0.0130	AT1G22250
0.00	3.27	3.60	3.43	0.0306	AT1G19640
0.00	3.38	3.49	3.43	0.0102	AT4G28556
0.00	3.30	3.56	3.43	0.0241	AT3G10190
0.00	3.34	3.52	3.43	0.0167	AT3G19270
0.00	3.67	3.18	3.43	0.0455	AT1G75920
0.00	3.53	3.32	3.43	0.0195	AT2G39060
0.00	3.33	3.52	3.43	0.0177	AT4G15680
0.00	3.28	3.56	3.42	0.0260	AT4G16140
0.00	3.42	3.41	3.41	0.0009	AT3G28220
0.00	3.32	3.50	3.41	0.0168	AT3G45930
0.00	3.18	3.63	3.40	0.0420	AT2G23050
0.00	3.37	3.43	3.40	0.0056	AT3G27360
0.00	3.51	3.29	3.40	0.0206	AT5G27330
0.00	3.52	3.27	3.39	0.0234	AT3G01328
0.00	3.28	3.50	3.39	0.0207	AT3G18485
0.00	3.05	3.72	3.38	0.0467	AT3G56350
0.00	3.48	3.29	3.38	0.0179	AT2G27040
0.00	3.36	3.40	3.38	0.0038	AT5G40310
0.00	3.39	3.37	3.38	0.0019	AT1G67035
0.00	3.26	3.49	3.38	0.0217	AT1G31140
0.00	3.19	3.56	3.38	0.0349	AT1G80540
0.00	3.32	3.42	3.37	0.0094	AT3G59420
0.00	3.47	3.27	3.37	0.0189	AT5G43590
0.00	3.29	3.44	3.37	0.0142	AT2G18328
0.00	3.26	3.46	3.36	0.0189	AT2G34490
0.00	3.48	3.23	3.35	0.0237	AT4G23380
0.00	3.18	3.52	3.35	0.0323	AT5G27220
0.00	3.23	3.47	3.35	0.0228	AT1G60060
0.00	3.41	3.29	3.35	0.0114	AT3G52820
0.00	3.37	3.33	3.35	0.0038	AT2G35760
0.00	3.38	3.32	3.35	0.0057	AT5G14610
0.00	3.46	3.23	3.35	0.0219	AT2G24610
0.00	3.46	3.22	3.34	0.0229	AT4G11400
0.00	3.22	3.46	3.34	0.0229	AT4G28250
0.00	3.40	3.28	3.34	0.0114	AT5G61570
0.00	3.29	3.39	3.34	0.0095	AT5G37790
0.00	3.16	3.52	3.34	0.0343	AT5G47610
0.00	3.61	3.07	3.34	0.0312	AT1G77210
0.00	3.30	3.37	3.34	0.0067	AT4G23820
0.00	3.28	3.39	3.33	0.0105	AT1G75750
0.00	3.13	3.53	3.33	0.0382	AT3G16240
0.00	3.43	3.23	3.33	0.0191	AT1G10680
0.00	3.21	3.45	3.33	0.0229	AT5G28000
0.00	3.23	3.42	3.33	0.0182	AT4G17215
0.00	3.33	3.32	3.32	0.0010	AT5G65360
0.00	3.36	3.28	3.32	0.0077	AT2G46290
0.00	3.38	3.25	3.31	0.0125	AT2G38180
0.00	3.53	3.09	3.31	0.0423	AT4G10190
0.00	3.27	3.35	3.31	0.0077	AT2G04160
0.00	3.21	3.40	3.31	0.0183	AT4G33160
0.00	3.32	3.29	3.31	0.0029	AT1G01900
0.00	3.18	3.43	3.31	0.0241	AT1G10780
0.00	3.07	3.54	3.31	0.0452	AT1G07370
0.00	3.42	3.19	3.30	0.0221	AT3G29050
0.00	3.40	3.21	3.30	0.0183	AT2G30424
0.00	3.22	3.39	3.30	0.0164	AT1G06660
0.00	3.55	3.06	3.30	0.0471	AT3G53075
0.00	3.29	3.31	3.30	0.0019	AT5G52330

0.00	3.19	3.41	3.30	0.0212	AT4G28650
0.00	3.28	3.31	3.30	0.0029	AT2G47010
0.00	3.30	3.30	3.30	0.0000	AT2G43360
0.00	3.13	3.46	3.29	0.0319	AT1G70985
0.00	3.09	3.49	3.29	0.0387	AT5G12050
0.00	3.51	3.07	3.29	0.0425	AT5G67360
0.00	3.18	3.40	3.29	0.0213	AT1G16750
0.00	3.31	3.27	3.29	0.0039	AT1G22370
0.00	3.20	3.37	3.29	0.0165	AT5G39493
0.00	3.11	3.46	3.29	0.0339	AT5G67070
0.00	3.51	3.05	3.28	0.0446	AT3G48160
0.00	3.28	3.28	3.28	0.0000	AT1G02460
0.00	3.11	3.45	3.28	0.0330	AT1G29460
0.00	3.41	3.14	3.28	0.0262	AT1G22110
0.00	3.17	3.38	3.28	0.0204	AT1G26100
0.00	3.17	3.38	3.28	0.0204	AT4G01410
0.00	3.48	3.06	3.27	0.0408	AT4G29700
0.00	3.13	3.41	3.27	0.0272	AT3G50660
0.00	3.26	3.27	3.26	0.0010	AT4G24510
0.00	3.32	3.20	3.26	0.0117	AT5G48950
0.00	3.19	3.32	3.26	0.0127	AT2G04780
0.00	3.03	3.48	3.26	0.0439	AT4G13710
0.00	3.16	3.34	3.25	0.0176	AT3G15550
0.00	3.23	3.27	3.25	0.0039	AT3G45230
0.00	3.04	3.44	3.24	0.0392	AT2G33330
0.00	3.43	3.04	3.24	0.0383	AT5G37010
0.00	3.47	3.00	3.24	0.0462	AT4G31760
0.00	3.17	3.30	3.23	0.0128	AT1G06830
0.00	3.08	3.38	3.23	0.0295	AT2G02130
0.00	3.20	3.26	3.23	0.0059	AT4G08630
0.00	3.07	3.39	3.23	0.0315	AT1G33170
0.00	3.29	3.17	3.23	0.0118	AT2G24615
0.00	3.41	3.04	3.22	0.0365	AT5G42280
0.00	3.02	3.42	3.22	0.0395	AT4G09984
0.00	3.23	3.22	3.22	0.0010	AT3G24220
0.00	3.33	3.11	3.22	0.0217	AT1G17770
0.00	3.35	3.08	3.22	0.0267	AT1G15260
0.00	3.20	3.24	3.22	0.0040	AT3G54560
0.00	3.06	3.37	3.21	0.0307	AT3G49220
0.00	3.17	3.26	3.21	0.0089	AT2G47780
0.00	3.31	3.11	3.21	0.0198	AT2G17630
0.00	3.07	3.35	3.21	0.0277	AT3G51470
0.00	3.12	3.28	3.20	0.0159	AT4G18760
0.00	3.06	3.34	3.20	0.0278	AT2G42900
0.00	3.03	3.37	3.20	0.0338	AT4G25490
0.00	3.36	3.04	3.20	0.0318	AT2G41540
0.00	3.44	2.94	3.19	0.0498	AT3G30180
0.00	3.19	3.19	3.19	0.0000	AT1G77270
0.00	2.99	3.38	3.19	0.0389	AT5G50375
0.00	3.24	3.12	3.18	0.0120	AT4G33130
0.00	3.32	3.05	3.18	0.0270	AT1G21880
0.00	2.98	3.38	3.18	0.0400	AT4G39700
0.00	3.14	3.23	3.18	0.0090	AT4G26760
0.00	3.21	3.14	3.18	0.0070	AT4G24040
0.00	3.39	2.96	3.17	0.0430	AT4G31370
0.00	3.26	3.07	3.16	0.0191	AT2G44190
0.00	2.98	3.35	3.16	0.0372	AT3G12170
0.00	2.99	3.34	3.16	0.0352	AT3G14530
0.00	3.04	3.29	3.16	0.0251	AT5G66800

0.00	3.32	3.00	3.16	0.0322	AT4G00160
0.00	3.07	3.24	3.16	0.0171	AT1G52400
0.00	3.10	3.21	3.16	0.0111	AT2G16850
0.00	3.15	3.16	3.15	0.0010	AT1G14490
0.00	2.95	3.36	3.15	0.0413	AT5G03795
0.00	3.36	2.94	3.15	0.0424	AT1G77580
0.00	3.23	3.08	3.15	0.0151	AT1G13590
0.00	3.09	3.19	3.14	0.0101	AT1G62370
0.00	2.98	3.31	3.14	0.0334	AT3G53080
0.00	3.18	3.10	3.14	0.0081	AT5G56580
0.00	3.14	3.13	3.14	0.0010	AT1G09540
0.00	3.32	2.95	3.14	0.0375	AT2G15090
0.00	3.14	3.13	3.13	0.0010	AT4G36180
0.00	3.09	3.17	3.13	0.0081	AT2G36080
0.00	3.35	2.91	3.13	0.0447	AT5G55370
0.00	2.92	3.34	3.13	0.0426	AT1G71770
0.00	2.95	3.30	3.12	0.0356	AT3G60220
0.00	3.22	3.02	3.12	0.0204	AT4G10950
0.00	2.92	3.32	3.12	0.0408	AT1G54040
0.00	3.17	3.06	3.12	0.0112	AT2G45490
0.00	3.16	3.07	3.11	0.0092	AT1G74160
0.00	3.04	3.18	3.11	0.0143	AT3G21460
0.00	2.95	3.26	3.11	0.0318	AT4G26320
0.00	2.89	3.32	3.10	0.0440	AT1G76830
0.00	3.22	2.99	3.10	0.0236	AT1G02040
0.00	3.08	3.13	3.10	0.0051	AT1G68120
0.00	2.87	3.33	3.10	0.0471	AT1G71320
0.00	2.90	3.29	3.10	0.0401	AT2G06850
0.00	3.00	3.19	3.09	0.0195	AT1G72250
0.00	3.25	2.93	3.09	0.0329	AT5G03840
0.00	3.20	2.98	3.09	0.0227	AT4G23800
0.00	3.23	2.95	3.09	0.0288	AT1G03170
0.00	3.06	3.11	3.09	0.0052	AT2G34170
0.00	3.22	2.95	3.09	0.0278	AT3G48410
0.00	3.03	3.14	3.08	0.0113	AT1G25450
0.00	3.13	3.03	3.08	0.0103	AT3G52970
0.00	3.15	3.00	3.08	0.0155	AT1G05210
0.00	3.01	3.14	3.08	0.0135	AT1G11220
0.00	3.28	2.87	3.08	0.0424	AT3G10910
0.00	3.10	3.05	3.07	0.0052	AT2G17300
0.00	2.97	3.17	3.07	0.0207	AT2G25880
0.00	3.05	3.09	3.07	0.0041	AT4G14713
0.00	3.07	3.07	3.07	0.0000	AT2G34650
0.00	3.01	3.12	3.07	0.0114	AT5G13790
0.00	2.87	3.26	3.07	0.0404	AT1G04680
0.00	3.05	3.07	3.06	0.0021	AT4G25960
0.00	2.99	3.14	3.06	0.0156	AT3G12145
0.00	3.06	3.06	3.06	0.0000	AT5G10390
0.00	3.14	2.98	3.06	0.0166	AT5G49800
0.00	3.03	3.08	3.06	0.0052	AT1G68570
0.00	3.03	3.08	3.05	0.0052	AT1G09470
0.00	3.12	2.99	3.05	0.0135	AT5G61730
0.00	3.13	2.96	3.04	0.0178	AT5G01910
0.00	3.05	3.03	3.04	0.0021	AT2G43820
0.00	3.21	2.86	3.04	0.0367	AT4G01730
0.00	3.00	3.07	3.04	0.0073	AT4G13990
0.00	3.16	2.92	3.04	0.0251	AT3G18630
0.00	3.13	2.94	3.04	0.0199	AT5G44110
0.00	2.89	3.18	3.03	0.0304	AT2G18260

0.00	3.14	2.92	3.03	0.0231	AT5G25470
0.00	2.98	3.07	3.02	0.0095	AT1G68250
0.00	3.18	2.86	3.02	0.0337	AT3G57860
0.00	3.08	2.95	3.01	0.0137	AT4G10860
0.00	3.00	3.01	3.01	0.0011	AT5G35390
0.00	2.82	3.19	3.00	0.0391	AT1G51190
0.00	3.20	2.79	3.00	0.0435	AT1G63850
0.00	3.04	2.95	3.00	0.0096	AT2G27145
0.00	2.84	3.16	3.00	0.0339	AT5G20850
0.00	2.79	3.20	3.00	0.0435	AT2G36400
0.00	3.07	2.91	2.99	0.0170	AT1G70380
0.00	3.23	2.76	2.99	0.0498	AT4G16620
0.00	2.76	3.23	2.99	0.0498	AT5G10430
0.00	2.94	3.04	2.99	0.0106	AT5G65410
0.00	2.85	3.12	2.99	0.0288	AT5G14920
0.00	3.06	2.91	2.98	0.0160	AT4G33467
0.00	2.88	3.08	2.98	0.0214	AT1G65920
0.00	2.88	3.08	2.98	0.0214	AT1G09350
0.00	2.80	3.16	2.98	0.0384	AT4G11211
0.00	2.87	3.08	2.98	0.0225	AT3G27330
0.00	2.83	3.13	2.98	0.0320	AT5G07500
0.00	2.85	3.11	2.98	0.0278	AT3G47295
0.00	3.20	2.76	2.98	0.0469	AT2G31985
0.00	2.92	3.03	2.98	0.0118	AT1G68170
0.00	3.13	2.82	2.97	0.0331	AT5G15660
0.00	2.83	3.12	2.97	0.0349	AT1G78090
0.00	2.98	2.97	2.97	0.0011	AT2G22230
0.00	2.78	3.16	2.97	0.0407	AT3G22020
0.00	2.97	2.97	2.97	0.0000	AT3G14240
0.00	3.03	2.91	2.97	0.0129	AT3G54250
0.00	2.83	3.11	2.97	0.0300	AT3G49580
0.00	3.06	2.87	2.96	0.0204	AT5G12910
0.00	3.08	2.85	2.96	0.0247	AT3G03280
0.00	2.95	2.98	2.96	0.0032	AT5G54190
0.00	3.14	2.79	2.96	0.0375	AT1G74055
0.00	2.81	3.11	2.96	0.0322	AT1G71830
0.00	2.85	3.05	2.95	0.0216	AT3G52340
0.00	3.14	2.74	2.94	0.0432	AT2G03090
0.00	2.81	3.07	2.94	0.0281	AT5G62700
0.00	2.81	3.06	2.94	0.0271	AT1G09450
0.00	2.72	3.15	2.94	0.0466	AT1G10380
0.00	2.74	3.12	2.93	0.0412	AT3G19850
0.00	2.89	2.97	2.93	0.0087	AT1G23080
0.00	2.83	3.02	2.93	0.0207	AT3G57160
0.00	2.79	3.05	2.92	0.0283	AT1G04250
0.00	2.76	3.08	2.92	0.0348	AT5G49215
0.00	2.93	2.90	2.91	0.0033	AT1G62640
0.00	3.02	2.80	2.91	0.0241	AT2G05990
0.00	2.81	3.02	2.91	0.0229	AT3G23670
0.00	2.99	2.83	2.91	0.0175	AT3G60840
0.00	2.80	3.02	2.91	0.0241	AT3G01410
0.00	3.06	2.74	2.90	0.0351	AT2G35640
0.00	2.92	2.88	2.90	0.0044	AT3G03826
0.00	2.94	2.85	2.90	0.0099	AT1G16850
0.00	2.92	2.87	2.90	0.0055	AT3G05020
0.00	2.91	2.88	2.89	0.0033	AT4G02060
0.00	2.75	3.03	2.89	0.0308	AT1G21090
0.00	2.88	2.89	2.89	0.0011	AT4G12730
0.00	2.88	2.89	2.89	0.0011	AT1G64640

0.00	2.75	3.02	2.89	0.0298	AT4G15700
0.00	2.73	3.04	2.89	0.0342	AT2G43261
0.00	2.96	2.81	2.88	0.0165	AT3G13520
0.00	2.83	2.93	2.88	0.0111	AT4G15970
0.00	2.83	2.93	2.88	0.0111	AT4G25850
0.00	2.80	2.95	2.88	0.0166	AT3G51680
0.00	2.89	2.86	2.88	0.0033	AT1G57750
0.00	2.79	2.95	2.87	0.0177	AT3G57500
0.00	3.08	2.67	2.87	0.0453	AT1G17060
0.00	2.82	2.93	2.87	0.0122	AT3G27060
0.00	2.66	3.08	2.87	0.0465	AT2G24630
0.00	3.09	2.65	2.87	0.0487	AT1G72130
0.00	3.08	2.65	2.87	0.0477	AT3G30842
0.00	2.74	3.00	2.87	0.0288	AT1G21460
0.00	2.93	2.80	2.87	0.0144	AT2G29890
0.00	2.68	3.04	2.86	0.0400	AT1G20650
0.00	2.76	2.96	2.86	0.0223	AT3G10080
0.00	3.01	2.70	2.86	0.0345	AT5G38080
0.00	2.82	2.89	2.86	0.0078	AT4G21480
0.00	2.66	3.04	2.85	0.0424	AT1G55740
0.00	2.98	2.72	2.85	0.0290	AT4G21820
0.00	2.87	2.83	2.85	0.0045	AT1G09750
0.00	3.01	2.67	2.84	0.0381	AT2G38810
0.00	2.72	2.96	2.84	0.0269	AT5G42180
0.00	2.66	3.02	2.84	0.0403	AT3G58650
0.00	2.80	2.88	2.84	0.0090	AT5G59510
0.00	2.86	2.82	2.84	0.0045	AT5G22791
0.00	2.77	2.90	2.84	0.0146	AT5G59020
0.00	2.76	2.89	2.83	0.0146	AT2G24280
0.00	2.61	3.04	2.83	0.0484	AT3G53900
0.00	2.68	2.97	2.83	0.0326	AT1G76240
0.00	2.78	2.86	2.82	0.0090	AT5G15950
0.00	2.78	2.85	2.81	0.0079	AT1G12460
0.00	2.93	2.69	2.81	0.0272	AT3G22880
0.00	3.03	2.60	2.81	0.0485	AT1G43800
0.00	2.79	2.83	2.81	0.0045	AT3G16150
0.00	2.60	3.01	2.81	0.0464	AT5G10720
0.00	2.65	2.96	2.80	0.0351	AT1G49470
0.00	2.83	2.78	2.80	0.0057	AT4G17680
0.00	2.66	2.94	2.80	0.0318	AT1G06260
0.00	2.87	2.72	2.79	0.0171	AT1G03440
0.00	2.73	2.84	2.79	0.0126	AT1G13608
0.00	2.94	2.63	2.79	0.0354	AT1G48180
0.00	2.96	2.61	2.79	0.0400	AT2G26330
0.00	2.63	2.94	2.79	0.0354	AT1G02180
0.00	2.72	2.85	2.79	0.0149	AT3G55360
0.00	2.87	2.69	2.78	0.0206	AT1G70680
0.00	2.84	2.73	2.78	0.0126	AT5G65710
0.00	2.76	2.79	2.78	0.0034	AT4G33820
0.00	2.56	2.99	2.77	0.0492	AT5G40155
0.00	2.82	2.71	2.77	0.0127	AT2G35490
0.00	2.65	2.87	2.76	0.0254	AT3G12700
0.00	2.73	2.80	2.76	0.0081	AT1G13250
0.00	2.67	2.84	2.75	0.0196	AT5G57260
0.00	2.84	2.67	2.75	0.0196	AT1G23130
0.00	2.63	2.88	2.75	0.0289	AT3G23060
0.00	2.83	2.67	2.75	0.0185	AT1G45130
0.00	2.85	2.66	2.75	0.0219	AT1G55360
0.00	2.78	2.71	2.75	0.0081	AT5G60490

0.00	2.77	2.72	2.74	0.0058	AT2G34060
0.00	2.57	2.91	2.74	0.0394	AT1G70990
0.00	2.87	2.61	2.74	0.0302	AT3G01350
0.00	2.73	2.74	2.73	0.0012	AT1G80640
0.00	2.64	2.83	2.73	0.0221	AT3G24450
0.00	2.76	2.70	2.73	0.0070	AT1G30600
0.00	2.61	2.85	2.73	0.0280	AT3G28960
0.00	2.59	2.87	2.73	0.0326	AT3G22770
0.00	2.64	2.81	2.73	0.0199	AT5G63090
0.00	2.81	2.64	2.73	0.0199	AT3G23727
0.00	2.82	2.63	2.73	0.0222	AT1G12805
0.00	2.74	2.71	2.73	0.0035	AT4G14690
0.00	2.67	2.78	2.72	0.0128	AT1G75090
0.00	2.68	2.76	2.72	0.0094	AT3G56260
0.00	2.64	2.79	2.72	0.0176	AT4G28190
0.00	2.85	2.58	2.71	0.0316	AT4G36600
0.00	2.52	2.90	2.71	0.0446	AT1G09240
0.00	2.66	2.76	2.71	0.0117	AT3G05890
0.00	2.55	2.87	2.71	0.0375	AT2G38060
0.00	2.67	2.75	2.71	0.0094	AT3G04960
0.00	2.80	2.61	2.71	0.0223	AT2G16890
0.00	2.72	2.68	2.70	0.0047	AT2G42570
0.00	2.66	2.73	2.70	0.0083	AT1G10100
0.00	2.76	2.64	2.70	0.0141	AT4G19430
0.00	2.79	2.61	2.70	0.0212	AT2G28620
0.00	2.53	2.87	2.70	0.0400	AT5G62960
0.00	2.85	2.54	2.70	0.0321	AT4G15530
0.00	2.68	2.71	2.69	0.0035	AT1G27670
0.00	2.57	2.82	2.69	0.0295	AT1G29980
0.00	2.58	2.81	2.69	0.0271	AT1G19440
0.00	2.80	2.58	2.69	0.0260	AT1G05370
0.00	2.74	2.65	2.69	0.0106	AT1G15760
0.00	2.67	2.71	2.69	0.0047	AT5G14130
0.00	2.55	2.82	2.69	0.0320	AT3G48460
0.00	2.56	2.81	2.69	0.0296	AT4G16460
0.00	2.69	2.68	2.69	0.0012	AT4G26960
0.00	2.53	2.84	2.68	0.0367	AT4G03210
0.00	2.59	2.77	2.68	0.0214	AT3G05400
0.00	2.80	2.56	2.68	0.0285	AT1G22170
0.00	2.62	2.74	2.68	0.0143	AT5G65420
0.00	2.74	2.61	2.68	0.0155	AT2G27770
0.00	2.69	2.66	2.68	0.0036	AT5G40150
0.00	2.80	2.56	2.68	0.0285	AT1G72600
0.00	2.60	2.75	2.67	0.0178	AT5G22740
0.00	2.53	2.82	2.67	0.0345	AT4G13700
0.00	2.66	2.69	2.67	0.0036	AT2G22900
0.00	2.74	2.59	2.67	0.0179	AT4G27980
0.00	2.54	2.79	2.67	0.0298	AT1G35310
0.00	2.63	2.70	2.67	0.0084	AT2G27060
0.00	2.55	2.79	2.67	0.0286	AT1G78390
0.00	2.55	2.78	2.66	0.0275	AT4G13230
0.00	2.63	2.69	2.66	0.0072	AT1G80160
0.00	2.64	2.68	2.66	0.0048	AT3G59550
0.00	2.48	2.85	2.66	0.0441	AT4G37580
0.00	2.50	2.81	2.66	0.0371	AT5G12900
0.00	2.49	2.82	2.66	0.0395	AT2G13820
0.00	2.53	2.77	2.65	0.0288	AT3G26445
0.00	2.67	2.63	2.65	0.0048	AT1G09200
0.00	2.64	2.66	2.65	0.0024	AT1G29640

0.00	2.45	2.85	2.65	0.0480	AT3G56480
0.00	2.83	2.47	2.65	0.0432	AT1G27120
0.00	2.50	2.78	2.64	0.0337	AT5G20270
0.00	2.65	2.63	2.64	0.0024	AT1G14580
0.00	2.59	2.69	2.64	0.0121	AT4G15690
0.00	2.53	2.75	2.64	0.0265	AT2G02780
0.00	2.47	2.80	2.63	0.0398	AT2G38110
0.00	2.74	2.53	2.63	0.0254	AT1G75390
0.00	2.57	2.70	2.63	0.0157	AT5G51890
0.00	2.66	2.60	2.63	0.0073	AT3G11600
0.00	2.60	2.67	2.63	0.0085	AT3G43160
0.00	2.47	2.79	2.63	0.0387	AT5G10310
0.00	2.55	2.71	2.63	0.0194	AT4G20430
0.00	2.56	2.70	2.63	0.0169	AT2G01660
0.00	2.60	2.64	2.62	0.0049	AT4G33330
0.00	2.55	2.69	2.62	0.0170	AT1G03010
0.00	2.48	2.76	2.62	0.0340	AT5G22140
0.00	2.48	2.76	2.62	0.0340	AT4G36770
0.00	2.54	2.69	2.61	0.0183	AT5G24570
0.00	2.52	2.70	2.61	0.0219	AT5G67230
0.00	2.53	2.69	2.61	0.0195	AT2G30766
0.00	2.53	2.69	2.61	0.0195	AT3G26770
0.00	2.56	2.65	2.61	0.0110	AT4G35783
0.00	2.59	2.62	2.61	0.0037	AT2G28330
0.00	2.72	2.49	2.60	0.0281	AT1G53230
0.00	2.59	2.61	2.60	0.0024	AT5G39320
0.00	2.58	2.62	2.60	0.0049	AT1G77590
0.00	2.65	2.55	2.60	0.0122	AT3G46770
0.00	2.52	2.67	2.60	0.0184	AT2G16440
0.00	2.48	2.71	2.59	0.0282	AT1G19940
0.00	2.51	2.68	2.59	0.0208	AT2G27130
0.00	2.65	2.54	2.59	0.0135	AT1G49580
0.00	2.52	2.67	2.59	0.0184	AT1G15870
0.00	2.64	2.55	2.59	0.0110	AT1G20870
0.00	2.63	2.56	2.59	0.0086	AT1G66730
0.00	2.73	2.45	2.59	0.0344	AT1G10370
0.00	2.51	2.66	2.59	0.0185	AT4G28703
0.00	2.56	2.61	2.58	0.0062	AT5G05965
0.00	2.58	2.58	2.58	0.0000	AT2G33560
0.00	2.66	2.50	2.58	0.0197	AT5G59690
0.00	2.57	2.58	2.58	0.0012	AT4G37295
0.00	2.48	2.68	2.58	0.0247	AT3G28917
0.00	2.48	2.67	2.58	0.0235	AT1G29660
0.00	2.42	2.72	2.57	0.0371	AT4G38050
0.00	2.72	2.42	2.57	0.0371	AT5G07720
0.00	2.41	2.73	2.57	0.0396	AT4G11180
0.00	2.64	2.50	2.57	0.0173	AT1G72270
0.00	2.39	2.75	2.57	0.0445	AT1G62870
0.00	2.62	2.51	2.57	0.0136	AT3G13000
0.00	2.42	2.70	2.56	0.0348	AT5G15310
0.00	2.68	2.44	2.56	0.0298	AT1G12330
0.00	2.66	2.45	2.56	0.0261	AT4G30800
0.00	2.67	2.45	2.56	0.0273	AT1G75550
0.00	2.44	2.67	2.56	0.0286	AT2G24230
0.00	2.51	2.60	2.55	0.0112	AT5G11460
0.00	1.24	3.86	2.55	0.0076	AT2G47520
0.00	2.45	2.65	2.55	0.0250	AT5G07590
0.00	2.45	2.66	2.55	0.0261	AT2G29130
0.00	2.58	2.52	2.55	0.0075	AT4G33400

0.00	2.45	2.66	2.55	0.0261	AT3G22830
0.00	2.60	2.51	2.55	0.0112	AT3G14890
0.00	2.61	2.49	2.55	0.0150	AT1G60390
0.00	2.35	2.75	2.55	0.0498	AT1G19060
0.00	2.70	2.40	2.55	0.0374	AT4G09510
0.00	2.38	2.72	2.55	0.0424	AT3G08030
0.00	2.49	2.61	2.55	0.0150	AT2G37560
0.00	2.35	2.74	2.55	0.0487	AT5G23780
0.00	2.44	2.64	2.54	0.0251	AT1G68800
0.00	2.50	2.58	2.54	0.0100	AT1G77760
0.00	2.64	2.44	2.54	0.0251	AT5G18600
0.00	2.59	2.48	2.54	0.0138	AT2G22190
0.00	2.56	2.51	2.54	0.0063	AT4G17180
0.00	2.45	2.61	2.53	0.0201	AT1G29395
0.00	2.61	2.45	2.53	0.0201	AT2G43800
0.00	2.51	2.55	2.53	0.0050	AT3G06140
0.00	2.64	2.41	2.53	0.0290	AT4G25433
0.00	2.37	2.69	2.53	0.0402	AT5G09980
0.00	2.56	2.49	2.53	0.0088	AT2G33400
0.00	2.46	2.59	2.53	0.0164	AT5G08260
0.00	2.55	2.50	2.52	0.0063	AT1G69480
0.00	2.62	2.43	2.52	0.0239	AT5G27450
0.00	2.52	2.52	2.52	0.0000	AT2G25530
0.00	2.63	2.41	2.52	0.0278	AT1G66760
0.00	2.44	2.60	2.52	0.0202	AT4G22470
0.00	2.59	2.45	2.52	0.0177	AT2G16060
0.00	2.45	2.58	2.52	0.0164	AT4G32290
0.00	2.62	2.41	2.51	0.0266	AT5G63650
0.00	2.39	2.63	2.51	0.0304	AT3G52940
0.00	2.64	2.38	2.51	0.0329	AT3G60530
0.00	2.34	2.67	2.51	0.0419	AT1G56670
0.00	2.63	2.38	2.51	0.0317	AT5G27550
0.00	2.66	2.35	2.50	0.0393	AT5G10020
0.00	2.58	2.42	2.50	0.0204	AT5G58960
0.00	2.40	2.60	2.50	0.0255	AT1G78370
0.00	2.39	2.60	2.49	0.0268	AT1G62050
0.00	2.53	2.46	2.49	0.0089	AT3G49250
0.00	2.35	2.64	2.49	0.0370	AT1G11740
0.00	2.45	2.53	2.49	0.0102	AT4G35100
0.00	2.36	2.62	2.49	0.0332	AT3G05330
0.00	2.63	2.35	2.49	0.0358	AT3G66656
0.00	2.68	2.29	2.48	0.0499	AT1G03120
0.00	2.57	2.39	2.48	0.0231	AT3G48980
0.00	2.62	2.34	2.48	0.0359	AT3G22790
0.00	2.52	2.44	2.48	0.0103	AT4G04460
0.00	2.37	2.59	2.48	0.0282	AT2G40085
0.00	2.59	2.36	2.48	0.0296	AT3G25860
0.00	2.45	2.50	2.48	0.0064	AT1G58340
0.00	2.64	2.31	2.48	0.0424	AT2G35730
0.00	2.55	2.40	2.48	0.0193	AT5G05240
0.00	2.28	2.66	2.47	0.0489	AT1G48100
0.00	2.43	2.52	2.47	0.0116	AT1G21150
0.00	2.30	2.64	2.47	0.0437	AT2G28710
0.00	2.55	2.40	2.47	0.0193	AT5G23910
0.00	2.32	2.62	2.47	0.0386	AT1G19450
0.00	2.58	2.35	2.47	0.0297	AT5G22200
0.00	2.32	2.61	2.46	0.0374	AT5G52900
0.00	2.54	2.38	2.46	0.0207	AT1G76740
0.00	2.60	2.32	2.46	0.0362	AT1G67630

0.00	2.51	2.41	2.46	0.0129	AT3G47600
0.00	2.47	2.45	2.46	0.0026	AT1G08810
0.00	2.32	2.60	2.46	0.0362	AT5G10620
0.00	2.52	2.39	2.46	0.0169	AT1G80850
0.00	2.56	2.35	2.45	0.0272	AT5G15210
0.00	2.33	2.57	2.45	0.0312	AT3G24630
0.00	2.33	2.57	2.45	0.0312	AT4G27730
0.00	2.44	2.46	2.45	0.0026	AT1G35780
0.00	2.28	2.61	2.45	0.0429	AT4G14200
0.00	2.58	2.32	2.45	0.0337	AT1G10550
0.00	2.53	2.36	2.45	0.0221	AT3G63110
0.00	2.36	2.53	2.45	0.0221	AT4G22250
0.00	2.50	2.38	2.44	0.0157	AT5G45630
0.00	2.54	2.34	2.44	0.0261	AT3G61930
0.00	2.25	2.63	2.44	0.0495	AT3G46660
0.00	2.51	2.36	2.44	0.0196	AT4G12440
0.00	2.36	2.51	2.44	0.0196	AT2G47485
0.00	2.29	2.58	2.44	0.0379	AT5G23420
0.00	2.42	2.46	2.44	0.0052	AT5G11510
0.00	2.26	2.61	2.44	0.0457	AT5G08150
0.00	2.33	2.53	2.43	0.0262	AT4G28180
0.00	2.55	2.30	2.43	0.0328	AT3G14740
0.00	2.56	2.29	2.42	0.0354	AT4G10910
0.00	2.38	2.46	2.42	0.0105	AT3G03170
0.00	2.42	2.42	2.42	0.0000	AT1G10030
0.00	2.53	2.29	2.41	0.0317	AT5G24280
0.00	2.37	2.46	2.41	0.0119	AT3G54750
0.00	2.30	2.52	2.41	0.0290	AT4G36360
0.00	2.29	2.53	2.41	0.0317	AT2G41940
0.00	2.26	2.55	2.41	0.0383	AT4G25845
0.00	2.43	2.38	2.41	0.0066	AT3G19820
0.00	2.31	2.50	2.40	0.0251	AT1G11130
0.00	2.42	2.39	2.40	0.0040	AT1G65295
0.00	2.25	2.55	2.40	0.0397	AT1G56430
0.00	2.46	2.34	2.40	0.0159	AT2G26580
0.00	2.42	2.38	2.40	0.0053	AT2G43050
0.00	2.49	2.31	2.40	0.0239	AT2G43040
0.00	2.43	2.37	2.40	0.0080	AT4G22756
0.00	2.48	2.32	2.40	0.0212	AT1G51440
0.00	2.33	2.46	2.40	0.0173	AT5G02220
0.00	2.50	2.29	2.39	0.0279	AT2G35585
0.00	2.41	2.38	2.39	0.0040	AT1G16920
0.00	2.41	2.37	2.39	0.0053	AT5G66310
0.00	2.22	2.56	2.39	0.0452	AT3G44610
0.00	2.50	2.28	2.39	0.0293	AT5G11230
0.00	2.39	2.38	2.39	0.0013	AT5G61130
0.00	2.54	2.22	2.38	0.0427	AT1G51130
0.00	2.30	2.46	2.38	0.0214	AT4G10960
0.00	2.33	2.43	2.38	0.0134	AT2G44690
0.00	2.31	2.44	2.38	0.0174	AT5G44040
0.00	2.26	2.49	2.38	0.0308	AT3G01550
0.00	2.44	2.32	2.38	0.0160	AT1G16780
0.00	2.33	2.42	2.37	0.0121	AT5G66660
0.00	2.29	2.45	2.37	0.0215	AT5G54510
0.00	2.28	2.46	2.37	0.0242	AT1G19510
0.00	2.20	2.55	2.37	0.0468	AT1G67600
0.00	2.24	2.50	2.37	0.0349	AT4G20140
0.00	2.35	2.38	2.37	0.0040	AT2G24170
0.00	2.35	2.39	2.37	0.0054	AT5G50420

0.00	2.23	2.50	2.37	0.0363	AT4G22235
0.00	2.52	2.21	2.36	0.0417	AT2G47360
0.00	2.38	2.34	2.36	0.0054	AT3G50700
0.00	2.35	2.37	2.36	0.0027	AT1G33480
0.00	2.45	2.27	2.36	0.0243	AT3G51240
0.00	2.25	2.47	2.36	0.0297	AT1G60960
0.00	2.49	2.22	2.36	0.0365	AT4G09170
0.00	2.39	2.31	2.35	0.0108	AT2G45360
0.00	2.38	2.32	2.35	0.0081	AT1G71870
0.00	2.30	2.40	2.35	0.0135	AT1G02190
0.00	2.37	2.33	2.35	0.0054	AT1G27045
0.00	2.21	2.48	2.35	0.0366	AT5G44010
0.00	2.25	2.44	2.34	0.0258	AT1G12380
0.00	2.44	2.25	2.34	0.0258	AT2G44130
0.00	2.24	2.44	2.34	0.0272	AT5G19090
0.00	2.42	2.26	2.34	0.0218	AT5G47380
0.00	2.30	2.37	2.34	0.0095	AT2G16760
0.00	2.36	2.32	2.34	0.0054	AT3G28857
0.00	2.47	2.20	2.34	0.0368	AT1G62520
0.00	2.22	2.45	2.33	0.0313	AT1G19950
0.00	2.40	2.27	2.33	0.0177	AT2G04235
0.00	2.25	2.41	2.33	0.0218	AT5G10160
0.00	2.01	2.64	2.33	0.0378	AT3G09640
0.00	2.33	2.32	2.32	0.0014	AT1G28700
0.00	2.41	2.24	2.32	0.0233	AT1G53160
0.00	2.30	2.34	2.32	0.0055	AT4G36105
0.00	2.47	2.16	2.32	0.0426	AT2G21800
0.00	2.22	2.40	2.31	0.0248	AT1G51060
0.00	2.34	2.28	2.31	0.0083	AT2G24440
0.00	2.18	2.44	2.31	0.0358	AT1G34430
0.00	2.34	2.27	2.31	0.0097	AT2G42090
0.00	2.38	2.24	2.31	0.0193	AT4G31380
0.00	2.17	2.44	2.31	0.0372	AT1G12860
0.00	2.24	2.37	2.31	0.0179	AT1G18350
0.00	2.28	2.33	2.31	0.0069	AT1G79480
0.00	2.12	2.48	2.30	0.0497	AT4G08040
0.00	2.32	2.28	2.30	0.0055	AT3G51600
0.00	2.29	2.31	2.30	0.0028	AT3G25700
0.00	2.36	2.24	2.30	0.0166	AT5G39890
0.00	2.41	2.19	2.30	0.0304	AT1G28390
0.00	2.15	2.45	2.30	0.0415	AT2G03505
0.00	2.37	2.23	2.30	0.0194	AT3G54920
0.00	2.27	2.33	2.30	0.0083	AT1G70895
0.00	2.48	2.12	2.30	0.0497	AT1G19330
0.00	2.41	2.19	2.30	0.0304	AT5G62550
0.00	2.24	2.35	2.29	0.0153	AT4G23640
0.00	2.34	2.25	2.29	0.0125	AT5G15960
0.00	2.25	2.33	2.29	0.0111	AT4G04080
0.00	2.17	2.40	2.29	0.0320	AT1G55330
0.00	2.33	2.24	2.28	0.0125	AT5G42120
0.00	2.22	2.34	2.28	0.0167	AT3G43270
0.00	2.34	2.22	2.28	0.0167	AT5G18660
0.00	2.20	2.36	2.28	0.0223	AT4G24140
0.00	2.33	2.22	2.28	0.0154	AT3G62050
0.00	2.12	2.43	2.28	0.0433	AT4G33600
0.00	2.33	2.22	2.27	0.0154	AT1G50240
0.00	2.45	2.10	2.27	0.0489	AT5G17980
0.00	2.31	2.24	2.27	0.0098	AT1G30040
0.00	2.34	2.19	2.27	0.0211	AT4G04890

0.00	2.38	2.15	2.27	0.0323	AT4G17190
0.00	2.44	2.09	2.26	0.0491	AT2G39900
0.00	2.18	2.35	2.26	0.0239	AT1G07090
0.00	2.18	2.35	2.26	0.0239	AT4G16360
0.00	2.41	2.12	2.26	0.0407	AT4G32710
0.00	2.33	2.19	2.26	0.0197	AT1G15190
0.00	2.11	2.41	2.26	0.0422	AT2G42200
0.00	2.25	2.26	2.26	0.0014	AT2G43550
0.00	2.14	2.37	2.26	0.0324	AT3G59140
0.00	2.26	2.25	2.25	0.0014	AT3G63060
0.00	2.28	2.22	2.25	0.0085	AT1G80270
0.00	2.39	2.11	2.25	0.0396	AT2G45340
0.00	2.09	2.41	2.25	0.0452	AT2G43660
0.00	2.10	2.40	2.25	0.0424	AT1G01300
0.00	2.24	2.26	2.25	0.0028	AT1G21810
0.00	2.28	2.21	2.25	0.0099	AT2G29050
0.00	2.28	2.21	2.25	0.0099	AT5G21100
0.00	2.20	2.29	2.25	0.0128	AT1G30570
0.00	2.26	2.23	2.24	0.0043	AT5G62360
0.00	2.37	2.12	2.24	0.0354	AT2G26640
0.00	2.07	2.42	2.24	0.0495	AT4G12490
0.00	2.19	2.29	2.24	0.0142	AT4G32830
0.00	2.08	2.40	2.24	0.0454	AT5G42720
0.00	2.28	2.20	2.24	0.0114	AT1G02340
0.00	2.40	2.07	2.24	0.0469	AT3G18490
0.00	2.25	2.22	2.24	0.0043	AT5G07140
0.00	2.10	2.36	2.23	0.0371	AT4G02100
0.00	2.31	2.15	2.23	0.0228	AT4G22010
0.00	2.27	2.18	2.23	0.0129	AT1G70370
0.00	2.30	2.16	2.23	0.0200	AT4G00893
0.00	2.39	2.06	2.22	0.0471	AT3G22640
0.00	2.06	2.39	2.22	0.0471	AT1G71020
0.00	2.32	2.13	2.22	0.0272	AT1G17030
0.00	2.27	2.17	2.22	0.0143	AT1G14180
0.00	2.14	2.30	2.22	0.0229	AT5G64850
0.00	2.29	2.15	2.22	0.0201	AT2G24970
0.00	2.29	2.15	2.22	0.0201	AT3G18550
0.00	2.10	2.34	2.22	0.0344	AT4G35650
0.00	2.32	2.11	2.22	0.0302	AT1G53870
0.00	2.06	2.37	2.22	0.0445	AT5G37980
0.00	2.20	2.23	2.21	0.0043	AT3G04470
0.00	2.37	2.06	2.21	0.0445	AT5G01890
0.00	2.10	2.32	2.21	0.0317	AT1G29670
0.00	2.16	2.26	2.21	0.0144	AT3G23805
0.00	2.27	2.14	2.21	0.0188	AT1G10270
0.00	2.06	2.35	2.20	0.0418	AT4G37770
0.00	2.07	2.33	2.20	0.0376	AT1G02450
0.00	2.17	2.22	2.20	0.0073	AT2G01120
0.00	2.15	2.24	2.20	0.0130	AT2G30200
0.00	2.13	2.26	2.19	0.0188	AT5G25590
0.00	2.05	2.34	2.19	0.0420	AT4G36540
0.00	2.17	2.21	2.19	0.0058	AT1G75960
0.00	2.13	2.26	2.19	0.0188	AT5G57700
0.00	2.33	2.05	2.19	0.0406	AT5G65350
0.00	2.22	2.15	2.19	0.0102	AT1G10560
0.00	2.15	2.22	2.18	0.0102	AT4G23690
0.00	2.15	2.22	2.18	0.0102	AT5G47230
0.00	2.12	2.25	2.18	0.0189	AT1G78770
0.00	2.07	2.29	2.18	0.0321	AT5G13710

0.00	2.24	2.12	2.18	0.0175	AT1G21060
0.00	2.04	2.33	2.18	0.0422	AT5G26000
0.00	2.05	2.31	2.18	0.0379	AT4G19230
0.00	2.13	2.22	2.18	0.0132	AT5G11690
0.00	2.07	2.28	2.18	0.0307	AT4G30410
0.00	2.35	2.01	2.18	0.0495	AT2G44930
0.00	2.32	2.02	2.17	0.0439	AT1G06450
0.00	2.19	2.15	2.17	0.0059	AT1G03070
0.00	2.12	2.22	2.17	0.0147	AT3G07960
0.00	2.24	2.09	2.16	0.0220	AT2G29370
0.00	2.18	2.14	2.16	0.0059	AT4G40010
0.00	2.06	2.26	2.16	0.0295	AT2G17370
0.00	2.01	2.32	2.16	0.0455	AT1G73360
0.00	2.31	2.01	2.16	0.0441	AT5G57240
0.00	2.15	2.17	2.16	0.0029	AT3G29810
0.00	2.19	2.13	2.16	0.0088	AT2G23700
0.00	2.04	2.28	2.16	0.0353	AT5G49665
0.00	2.13	2.18	2.16	0.0074	AT3G20670
0.00	2.16	2.15	2.15	0.0015	AT5G41460
0.00	1.99	2.32	2.15	0.0486	AT1G17830
0.00	2.01	2.30	2.15	0.0428	AT3G06035
0.00	2.19	2.11	2.15	0.0118	AT5G59800
0.00	2.26	2.04	2.15	0.0325	AT5G05510
0.00	2.06	2.22	2.14	0.0238	AT1G22570
0.00	2.16	2.12	2.14	0.0059	AT1G75060
0.00	2.01	2.28	2.14	0.0400	AT3G02170
0.00	2.31	1.98	2.14	0.0489	AT5G15740
0.00	2.07	2.21	2.14	0.0208	AT3G48970
0.00	2.23	2.05	2.14	0.0268	AT5G47670
0.00	2.10	2.17	2.14	0.0104	AT1G28010
0.00	2.17	2.10	2.14	0.0104	AT5G50820
0.00	2.02	2.26	2.14	0.0357	AT5G53830
0.00	2.07	2.20	2.13	0.0194	AT3G13690
0.00	2.19	2.07	2.13	0.0179	AT1G07420
0.00	2.08	2.18	2.13	0.0149	AT2G34850
0.00	1.99	2.27	2.13	0.0418	AT2G03200
0.00	2.04	2.22	2.13	0.0269	AT1G55205
0.00	2.10	2.16	2.13	0.0090	AT5G16080
0.00	2.16	2.10	2.13	0.0090	AT1G78340
0.00	2.16	2.10	2.13	0.0090	AT3G28040
0.00	2.28	1.97	2.13	0.0464	AT3G26922
0.00	2.05	2.21	2.13	0.0239	AT2G22600
0.00	2.11	2.14	2.13	0.0045	AT5G25830
0.00	2.24	2.01	2.12	0.0344	AT1G68400
0.00	2.14	2.10	2.12	0.0060	AT2G26910
0.00	2.11	2.13	2.12	0.0030	AT3G13672
0.00	2.09	2.15	2.12	0.0090	AT2G25685
0.00	2.16	2.08	2.12	0.0120	AT1G70830
0.00	2.15	2.08	2.12	0.0105	AT1G53060
0.00	2.19	2.05	2.12	0.0210	AT2G28740
0.00	2.21	2.02	2.12	0.0286	AT1G65010
0.00	2.24	1.99	2.11	0.0376	AT5G06590
0.00	1.99	2.23	2.11	0.0362	AT3G15020
0.00	1.98	2.23	2.11	0.0378	AT5G50940
0.00	2.08	2.13	2.11	0.0076	AT4G19460
0.00	2.07	2.14	2.11	0.0106	AT3G20150
0.00	2.19	2.02	2.10	0.0257	AT5G43870
0.00	2.15	2.05	2.10	0.0152	AT4G22290
0.00	2.07	2.12	2.10	0.0076	AT4G35840

0.00	2.20	1.99	2.10	0.0319	AT1G08280
0.00	2.05	2.14	2.10	0.0137	AT3G62630
0.00	2.00	2.19	2.09	0.0288	AT2G34920
0.00	1.97	2.21	2.09	0.0365	AT5G44020
0.00	2.02	2.16	2.09	0.0213	AT2G48030
0.00	2.03	2.14	2.09	0.0168	AT5G05390
0.00	2.00	2.17	2.09	0.0259	AT5G41060
0.00	1.98	2.18	2.08	0.0306	AT2G41650
0.00	2.68	1.48	2.08	0.0256	AT1G20620
0.00	2.04	2.11	2.08	0.0107	AT3G46550
0.00	2.15	2.00	2.07	0.0230	AT3G56570
0.00	2.03	2.12	2.07	0.0138	AT3G03680
0.00	2.14	2.01	2.07	0.0199	AT5G35120
0.00	2.16	1.99	2.07	0.0261	AT1G72140
0.00	2.03	2.11	2.07	0.0123	AT2G18340
0.00	2.11	2.03	2.07	0.0123	AT2G29570
0.00	2.15	1.98	2.07	0.0262	AT1G59930
0.00	2.03	2.10	2.07	0.0108	AT3G04850
0.00	1.95	2.18	2.07	0.0354	AT5G10480
0.00	2.02	2.11	2.07	0.0139	AT3G59900
0.00	2.07	2.06	2.06	0.0015	AT1G04820
0.00	2.14	1.99	2.06	0.0231	AT4G02170
0.00	1.92	2.21	2.06	0.0446	AT5G22870
0.00	1.97	2.15	2.06	0.0278	AT3G52370
0.00	1.91	2.21	2.06	0.0463	AT2G46970
0.00	2.06	2.06	2.06	0.0000	AT5G46570
0.00	1.99	2.12	2.06	0.0201	AT2G33640
0.00	1.93	2.18	2.05	0.0387	AT5G46710
0.00	2.19	1.92	2.05	0.0418	AT5G46290
0.00	2.01	2.10	2.05	0.0139	AT2G37585
0.00	1.95	2.16	2.05	0.0325	AT3G22070
0.00	1.91	2.19	2.05	0.0434	AT4G00390
0.00	2.07	2.02	2.05	0.0078	AT3G23167
0.00	1.97	2.12	2.04	0.0233	AT1G15950
0.00	2.18	1.91	2.04	0.0420	AT2G01760
0.00	1.98	2.10	2.04	0.0187	AT3G16570
0.00	1.88	2.20	2.04	0.0498	AT4G30650
0.00	2.18	1.90	2.04	0.0436	AT4G24710
0.00	1.93	2.15	2.04	0.0343	AT3G07990
0.00	2.03	2.05	2.04	0.0031	AT1G22980
0.00	2.07	2.00	2.04	0.0109	AT4G11450
0.00	2.11	1.96	2.03	0.0235	AT5G15900
0.00	2.08	1.99	2.03	0.0141	AT4G14150
0.00	2.03	2.03	2.03	0.0000	AT1G65370
0.00	2.14	1.93	2.03	0.0328	AT3G19380
0.00	2.15	1.91	2.03	0.0376	AT1G50010
0.00	2.04	2.02	2.03	0.0031	AT1G63230
0.00	1.90	2.16	2.03	0.0407	AT3G28500
0.00	2.15	1.90	2.03	0.0392	AT2G35620
0.00	1.92	2.12	2.02	0.0315	AT4G14020
0.00	1.98	2.06	2.02	0.0126	AT2G26730
0.00	1.87	2.17	2.02	0.0472	AT2G42300
0.00	1.89	2.14	2.02	0.0394	AT2G45900
0.00	1.97	2.06	2.02	0.0142	AT3G19050
0.00	2.11	1.92	2.01	0.0300	AT5G35670
0.00	1.88	2.14	2.01	0.0411	AT5G67200
0.00	2.12	1.91	2.01	0.0331	AT1G51770
0.00	2.00	2.02	2.01	0.0032	AT4G21870
0.00	1.96	2.06	2.01	0.0158	AT4G12970

0.00	2.05	1.96	2.01	0.0143	AT4G01580
0.00	1.98	2.03	2.00	0.0079	AT1G49730
0.00	2.02	1.98	2.00	0.0064	AT1G07640
0.00	1.99	2.01	2.00	0.0032	AT1G62150
0.00	2.13	1.86	2.00	0.0430	AT2G36660
0.00	1.90	2.10	2.00	0.0318	AT4G27230
0.00	-1.91	-2.08	-2.00	0.0271	AT2G46360
0.00	-2.05	-1.96	-2.00	0.0143	AT5G61370
0.00	-1.87	-2.14	-2.00	0.0428	AT4G17660
0.00	-1.92	-2.10	-2.01	0.0285	AT2G23900
0.00	-2.15	-1.87	-2.01	0.0443	AT4G36850
0.00	-2.00	-2.02	-2.01	0.0032	AT4G39030
0.00	-1.89	-2.15	-2.02	0.0409	AT4G34730
0.00	-2.02	-2.02	-2.02	0.0000	AT4G04490
0.00	-2.16	-1.88	-2.02	0.0441	AT2G36120
0.00	-1.90	-2.14	-2.02	0.0378	AT3G14205
0.00	-2.04	-2.01	-2.02	0.0047	AT5G58390
0.00	-1.88	-2.17	-2.02	0.0455	AT1G12780
0.00	-1.87	-2.18	-2.02	0.0486	AT1G76370
0.00	-2.17	-1.89	-2.03	0.0438	AT3G62150
0.00	-1.96	-2.10	-2.03	0.0219	AT4G34600
0.00	-2.15	-1.91	-2.03	0.0376	AT4G37920
0.00	-1.93	-2.13	-2.03	0.0313	AT1G78830
0.00	-2.14	-1.93	-2.04	0.0328	AT1G11765
0.00	-2.14	-1.93	-2.04	0.0328	AT2G43590
0.00	-2.20	-1.88	-2.04	0.0498	AT2G46690
0.00	-1.96	-2.12	-2.04	0.0250	AT3G63500
0.00	-2.07	-2.01	-2.04	0.0094	AT3G01860
0.00	-1.94	-2.15	-2.04	0.0327	AT1G72030
0.00	-1.89	-2.20	-2.04	0.0482	AT1G77230
0.00	-1.96	-2.13	-2.04	0.0264	AT1G52240
0.00	-2.06	-2.03	-2.04	0.0047	AT5G23240
0.00	-2.04	-2.04	-2.04	0.0000	AT5G54090
0.00	-1.91	-2.18	-2.04	0.0420	AT3G26220
0.00	-2.03	-2.06	-2.04	0.0047	AT5G41040
0.00	-2.10	-1.99	-2.05	0.0171	AT5G36220
0.00	-1.90	-2.20	-2.05	0.0465	AT1G53590
0.00	-1.92	-2.19	-2.05	0.0418	AT5G56640
0.00	-2.01	-2.10	-2.06	0.0139	AT4G03400
0.00	-1.96	-2.16	-2.06	0.0309	AT4G34140
0.00	-1.91	-2.21	-2.06	0.0463	AT4G28400
0.00	-2.07	-2.06	-2.06	0.0015	AT3G15620
0.00	-2.01	-2.12	-2.07	0.0170	AT1G80440
0.00	-1.92	-2.22	-2.07	0.0461	AT3G06260
0.00	-2.05	-2.09	-2.07	0.0062	AT5G53110
0.00	-1.92	-2.21	-2.07	0.0446	AT4G13810
0.00	-2.12	-2.02	-2.07	0.0154	AT1G65660
0.00	-2.20	-1.95	-2.07	0.0383	AT5G64530
0.00	-1.93	-2.23	-2.08	0.0458	AT5G26170
0.00	-2.07	-2.10	-2.08	0.0046	AT3G42640
0.00	-2.07	-2.10	-2.08	0.0046	AT5G57630
0.00	-1.96	-2.21	-2.08	0.0381	AT2G18570
0.00	-2.15	-2.02	-2.09	0.0198	AT4G18740
0.00	-2.02	-2.16	-2.09	0.0213	AT4G02660
0.00	-2.22	-1.96	-2.09	0.0395	AT3G01085
0.00	-2.04	-2.15	-2.09	0.0167	AT2G16365
0.00	-2.19	-2.01	-2.10	0.0273	AT1G23850
0.00	-2.19	-2.00	-2.10	0.0288	AT5G58660
0.00	-2.02	-2.18	-2.10	0.0242	AT4G13580

0.00	-2.16	-2.05	-2.10	0.0166	AT1G10990
0.00	-2.03	-2.18	-2.11	0.0227	AT5G65610
0.00	-2.16	-2.05	-2.11	0.0166	AT4G31875
0.00	-1.95	-2.27	-2.11	0.0482	AT1G72150
0.00	-2.12	-2.11	-2.11	0.0015	AT3G20190
0.00	-1.99	-2.24	-2.12	0.0376	AT5G55570
0.00	-2.27	-1.97	-2.12	0.0450	AT1G71140
0.00	-2.16	-2.08	-2.12	0.0120	AT4G33420
0.00	-2.19	-2.06	-2.12	0.0195	AT2G31500
0.00	-2.15	-2.11	-2.13	0.0060	AT2G29070
0.00	-2.13	-2.13	-2.13	0.0000	AT2G01320
0.00	-2.02	-2.24	-2.13	0.0328	AT3G26170
0.00	-2.09	-2.17	-2.13	0.0120	AT3G14420
0.00	-2.01	-2.25	-2.13	0.0358	AT5G67340
0.00	-2.05	-2.21	-2.13	0.0239	AT3G48180
0.00	-2.10	-2.17	-2.14	0.0104	AT5G40450
0.00	-2.15	-2.12	-2.14	0.0045	AT1G04350
0.00	-2.13	-2.15	-2.14	0.0030	AT1G55930
0.00	-2.16	-2.13	-2.14	0.0045	AT1G10140
0.00	-2.06	-2.24	-2.15	0.0266	AT2G41260
0.00	-2.05	-2.26	-2.15	0.0310	AT5G64890
0.00	-2.31	-2.00	-2.15	0.0457	AT4G14560
0.00	-2.17	-2.14	-2.15	0.0044	AT1G58030
0.00	-2.00	-2.31	-2.15	0.0457	AT1G08140
0.00	-2.16	-2.16	-2.16	0.0000	AT5G64100
0.00	-2.12	-2.21	-2.16	0.0132	AT3G50970
0.00	-2.14	-2.19	-2.16	0.0074	AT3G09405
0.00	-2.15	-2.19	-2.17	0.0059	AT1G04470
0.00	-2.32	-2.02	-2.17	0.0439	AT5G56870
0.00	-2.02	-2.33	-2.18	0.0453	AT1G23560
0.00	-2.17	-2.19	-2.18	0.0029	AT5G53050
0.00	-2.04	-2.32	-2.18	0.0408	AT5G28830
0.00	-2.02	-2.34	-2.18	0.0466	AT1G04540
0.00	-2.29	-2.10	-2.19	0.0275	AT2G36650
0.00	-2.14	-2.25	-2.19	0.0159	AT3G13090
0.00	-2.30	-2.09	-2.19	0.0304	AT3G24620
0.00	-2.10	-2.29	-2.20	0.0275	AT5G45490
0.00	-2.25	-2.14	-2.20	0.0159	AT1G01460
0.00	-2.32	-2.08	-2.20	0.0347	AT5G13730
0.00	-2.03	-2.37	-2.20	0.0491	AT1G78820
0.00	-2.21	-2.20	-2.20	0.0014	AT3G12500
0.00	-2.26	-2.15	-2.21	0.0159	AT2G14800
0.00	-2.13	-2.28	-2.21	0.0216	AT3G53150
0.00	-2.07	-2.35	-2.21	0.0403	AT5G17050
0.00	-2.35	-2.07	-2.21	0.0403	AT1G66870
0.00	-2.15	-2.27	-2.21	0.0173	AT4G01630
0.00	-2.38	-2.05	-2.21	0.0473	AT4G31230
0.00	-2.27	-2.16	-2.22	0.0158	AT5G64800
0.00	-2.26	-2.19	-2.22	0.0100	AT1G75700
0.00	-2.07	-2.39	-2.23	0.0456	AT1G33960
0.00	-2.22	-2.24	-2.23	0.0029	AT5G23760
0.00	-2.24	-2.22	-2.23	0.0029	AT4G21840
0.00	-2.07	-2.39	-2.23	0.0456	AT1G02820
0.00	-2.34	-2.13	-2.24	0.0299	AT3G14690
0.00	-2.36	-2.12	-2.24	0.0341	AT4G35610
0.00	-2.11	-2.37	-2.24	0.0369	AT3G15500
0.00	-2.40	-2.09	-2.24	0.0439	AT2G15318
0.00	-2.09	-2.40	-2.25	0.0439	AT4G27500
0.00	-2.11	-2.39	-2.25	0.0396	AT2G32540

0.00	-2.26	-2.24	-2.25	0.0028	AT3G46690
0.00	-2.40	-2.09	-2.25	0.0439	AT1G18020
0.00	-2.10	-2.41	-2.25	0.0437	AT2G28800
0.00	-2.24	-2.27	-2.25	0.0042	AT1G78290
0.00	-2.10	-2.41	-2.26	0.0437	AT3G15356
0.00	-2.12	-2.39	-2.26	0.0381	AT5G56840
0.00	-2.26	-2.25	-2.26	0.0014	AT4G04510
0.00	-2.12	-2.39	-2.26	0.0381	AT5G07475
0.00	-2.20	-2.32	-2.26	0.0169	AT4G23540
0.00	-2.11	-2.41	-2.26	0.0422	AT5G03310
0.00	-2.28	-2.25	-2.26	0.0042	AT1G18265
0.00	-2.30	-2.23	-2.27	0.0098	AT1G10920
0.00	-2.29	-2.25	-2.27	0.0056	AT5G28690
0.00	-2.23	-2.32	-2.27	0.0126	AT5G24540
0.00	-2.17	-2.39	-2.28	0.0307	AT1G05580
0.00	-2.39	-2.17	-2.28	0.0307	AT2G20670
0.00	-2.29	-2.28	-2.28	0.0014	AT2G24850
0.00	-2.13	-2.45	-2.29	0.0444	AT1G66980
0.00	-2.28	-2.31	-2.30	0.0042	AT4G11070
0.00	-2.17	-2.42	-2.30	0.0346	AT1G03090
0.00	-2.23	-2.37	-2.30	0.0194	AT3G54800
0.00	-2.20	-2.40	-2.30	0.0277	AT1G74250
0.00	-2.12	-2.48	-2.30	0.0497	AT1G54520
0.00	-2.16	-2.46	-2.31	0.0413	AT3G07600
0.00	-2.18	-2.45	-2.32	0.0371	AT1G01490
0.00	-2.40	-2.24	-2.32	0.0219	AT2G15480
0.00	-2.31	-2.34	-2.32	0.0041	AT3G21870
0.00	-2.38	-2.27	-2.32	0.0151	AT4G01700
0.00	-2.20	-2.45	-2.33	0.0342	AT3G44735
0.00	-2.51	-2.15	-2.33	0.0491	AT3G62990
0.00	-2.30	-2.37	-2.33	0.0095	AT2G02720
0.00	-2.18	-2.48	-2.33	0.0409	AT1G06570
0.00	-2.37	-2.30	-2.33	0.0095	AT3G55970
0.00	-2.39	-2.28	-2.34	0.0150	AT3G43250
0.00	-2.25	-2.43	-2.34	0.0245	AT5G52730
0.00	-2.25	-2.42	-2.34	0.0232	AT4G30140
0.00	-2.31	-2.37	-2.34	0.0082	AT1G77330
0.00	-2.41	-2.28	-2.34	0.0176	AT4G36670
0.00	-2.44	-2.25	-2.34	0.0258	AT4G32940
0.00	-2.31	-2.37	-2.34	0.0082	AT3G48450
0.00	-2.21	-2.48	-2.34	0.0366	AT5G23270
0.00	-2.26	-2.43	-2.35	0.0231	AT4G15030
0.00	-2.24	-2.46	-2.35	0.0298	AT3G04700
0.00	-2.21	-2.49	-2.35	0.0379	AT4G32040
0.00	-2.29	-2.41	-2.35	0.0163	AT1G30320
0.00	-2.34	-2.38	-2.36	0.0054	AT1G77145
0.00	-2.29	-2.43	-2.36	0.0189	AT1G44160
0.00	-2.36	-2.37	-2.37	0.0013	AT1G79280
0.00	-2.27	-2.46	-2.37	0.0256	AT3G14630
0.00	-2.37	-2.37	-2.37	0.0000	AT5G63530
0.00	-2.39	-2.36	-2.38	0.0040	AT4G09780
0.00	-2.24	-2.52	-2.38	0.0374	AT1G35490
0.00	-2.23	-2.53	-2.38	0.0401	AT1G68010
0.00	-2.48	-2.29	-2.38	0.0253	AT3G05725
0.00	-2.43	-2.33	-2.38	0.0134	AT5G15110
0.00	-2.31	-2.47	-2.39	0.0213	AT1G49970
0.00	-2.43	-2.35	-2.39	0.0107	AT1G67980
0.00	-2.52	-2.27	-2.39	0.0332	AT5G67080
0.00	-2.42	-2.39	-2.40	0.0040	AT3G61880

0.00	-2.53	-2.28	-2.41	0.0331	AT1G78450
0.00	-2.41	-2.42	-2.41	0.0013	AT5G67030
0.00	-2.35	-2.48	-2.41	0.0171	AT1G53980
0.00	-2.32	-2.51	-2.42	0.0250	AT1G17540
0.00	-2.53	-2.33	-2.43	0.0262	AT1G76410
0.00	-2.36	-2.51	-2.43	0.0196	AT1G20470
0.00	-2.32	-2.55	-2.44	0.0300	AT5G43180
0.00	-2.27	-2.62	-2.45	0.0455	AT2G41870
0.00	-2.32	-2.58	-2.45	0.0337	AT1G65500
0.00	-2.26	-2.64	-2.45	0.0493	AT4G00940
0.00	-2.42	-2.47	-2.45	0.0065	AT1G26380
0.00	-2.40	-2.50	-2.45	0.0130	AT4G15130
0.00	-2.38	-2.52	-2.45	0.0182	AT3G28540
0.00	-2.32	-2.58	-2.45	0.0337	AT3G45300
0.00	-2.39	-2.52	-2.45	0.0169	AT1G10480
0.00	-2.51	-2.40	-2.46	0.0143	AT1G54410
0.00	-2.55	-2.37	-2.46	0.0233	AT1G07340
0.00	-2.42	-2.50	-2.46	0.0104	AT5G54170
0.00	-2.43	-2.49	-2.46	0.0078	AT1G09415
0.00	-2.39	-2.54	-2.46	0.0194	AT1G11070
0.00	-2.38	-2.56	-2.47	0.0232	AT3G54150
0.00	-2.49	-2.45	-2.47	0.0052	AT5G56550
0.00	-2.36	-2.60	-2.48	0.0308	AT2G28180
0.00	-2.62	-2.34	-2.48	0.0359	AT1G09080
0.00	-2.39	-2.59	-2.49	0.0256	AT1G77930
0.00	-2.49	-2.49	-2.49	0.0000	AT5G52740
0.00	-2.55	-2.43	-2.49	0.0153	AT4G37820
0.00	-2.45	-2.54	-2.50	0.0115	AT3G26440
0.00	-2.34	-2.66	-2.50	0.0407	AT1G33612
0.00	-2.61	-2.39	-2.50	0.0280	AT2G15029
0.00	-2.49	-2.51	-2.50	0.0025	AT2G24990
0.00	-2.55	-2.46	-2.50	0.0114	AT1G61820
0.00	-2.37	-2.64	-2.50	0.0343	AT2G01100
0.00	-2.40	-2.62	-2.51	0.0279	AT2G15080
0.00	-2.70	-2.33	-2.51	0.0467	AT4G16630
0.00	-2.44	-2.58	-2.51	0.0177	AT5G48570
0.00	-2.57	-2.46	-2.52	0.0139	AT2G02710
0.00	-2.37	-2.66	-2.52	0.0367	AT3G17980
0.00	-2.33	-2.71	-2.52	0.0479	AT1G77810
0.00	-2.42	-2.62	-2.52	0.0252	AT1G66852
0.00	-2.33	-2.71	-2.52	0.0479	AT4G37560
0.00	-2.37	-2.68	-2.52	0.0390	AT4G30250
0.00	-2.46	-2.59	-2.52	0.0164	AT1G79860
0.00	-2.69	-2.38	-2.54	0.0389	AT4G10500
0.00	-2.74	-2.35	-2.54	0.0487	AT1G18990
0.00	-2.54	-2.55	-2.55	0.0013	AT3G10120
0.00	-2.40	-2.69	-2.55	0.0362	AT3G45710
0.00	-2.50	-2.62	-2.56	0.0149	AT5G63970
0.00	-2.37	-2.77	-2.57	0.0494	AT1G17820
0.00	-2.57	-2.59	-2.58	0.0025	AT1G20440
0.00	-2.61	-2.55	-2.58	0.0074	AT3G10790
0.00	-2.48	-2.69	-2.58	0.0258	AT2G05850
0.00	-2.54	-2.63	-2.59	0.0111	AT1G16730
0.00	-2.68	-2.50	-2.59	0.0221	AT3G21710
0.00	-2.73	-2.46	-2.59	0.0331	AT4G18340
0.00	-2.42	-2.76	-2.59	0.0417	AT3G28930
0.00	-2.64	-2.56	-2.60	0.0098	AT3G26830
0.00	-2.45	-2.75	-2.60	0.0367	AT1G79680
0.00	-2.52	-2.68	-2.60	0.0196	AT4G01895

0.00	-2.64	-2.56	-2.60	0.0098	AT1G47890
0.00	-2.53	-2.68	-2.60	0.0183	AT4G09300
0.00	-2.74	-2.48	-2.61	0.0317	AT5G57520
0.00	-2.62	-2.61	-2.62	0.0012	AT4G31870
0.00	-2.60	-2.64	-2.62	0.0049	AT3G52010
0.00	-2.53	-2.71	-2.62	0.0219	AT3G45160
0.00	-2.63	-2.63	-2.63	0.0000	AT3G15840
0.00	-2.68	-2.57	-2.63	0.0133	AT5G45090
0.00	-2.65	-2.63	-2.64	0.0024	AT2G31380
0.00	-2.52	-2.77	-2.64	0.0301	AT1G80810
0.00	-2.49	-2.83	-2.66	0.0406	AT5G48140
0.00	-2.80	-2.52	-2.66	0.0335	AT1G52580
0.00	-2.56	-2.78	-2.67	0.0262	AT2G43850
0.00	-2.66	-2.70	-2.68	0.0048	AT2G04038
0.00	-2.59	-2.78	-2.68	0.0225	AT4G27300
0.00	-2.62	-2.75	-2.69	0.0154	AT4G13040
0.00	-2.58	-2.80	-2.69	0.0260	AT5G53320
0.00	-2.66	-2.74	-2.70	0.0094	AT4G06746
0.00	-2.84	-2.56	-2.70	0.0330	AT1G32510
0.00	-2.68	-2.73	-2.70	0.0059	AT2G19050
0.00	-2.51	-2.91	-2.71	0.0469	AT1G72540
0.00	-2.63	-2.81	-2.72	0.0211	AT3G05900
0.00	-2.78	-2.66	-2.72	0.0140	AT2G26975
0.00	-2.55	-2.91	-2.73	0.0419	AT3G20530
0.00	-2.86	-2.63	-2.75	0.0267	AT2G27285
0.00	-2.69	-2.80	-2.75	0.0128	AT1G66600
0.00	-2.68	-2.84	-2.76	0.0184	AT3G17510
0.00	-2.59	-2.95	-2.77	0.0413	AT2G05540
0.00	-2.68	-2.90	-2.79	0.0251	AT5G13080
0.00	-2.66	-2.96	-2.81	0.0340	AT1G60730
0.00	-2.65	-2.98	-2.82	0.0373	AT1G75040
0.00	-2.81	-2.83	-2.82	0.0023	AT4G04500
0.00	-2.64	-3.01	-2.83	0.0416	AT4G37925
0.00	-2.73	-2.93	-2.83	0.0225	AT3G60300
0.00	-2.76	-2.90	-2.83	0.0157	AT5G15140
0.00	-2.77	-2.92	-2.84	0.0168	AT2G30770
0.00	-2.71	-2.98	-2.85	0.0302	AT1G62630
0.00	-2.79	-2.92	-2.85	0.0145	AT1G20120
0.00	-2.94	-2.79	-2.86	0.0167	AT5G64110
0.00	-2.98	-2.75	-2.86	0.0255	AT5G58784
0.00	-2.95	-2.78	-2.86	0.0189	AT2G34610
0.00	-2.79	-2.95	-2.87	0.0177	AT3G49340
0.00	-2.69	-3.06	-2.87	0.0409	AT3G51880
0.00	-2.75	-3.02	-2.89	0.0298	AT5G07080
0.00	-2.76	-3.01	-2.89	0.0276	AT1G08450
0.00	-2.72	-3.06	-2.89	0.0374	AT3G49601
0.00	-2.88	-2.91	-2.90	0.0033	AT1G43160
0.00	-3.08	-2.73	-2.91	0.0383	AT2G07140
0.00	-2.89	-2.93	-2.91	0.0044	AT1G28190
0.00	-2.91	-2.91	-2.91	0.0000	AT5G55490
0.00	-2.87	-2.96	-2.91	0.0098	AT2G07040
0.00	-2.70	-3.15	-2.92	0.0489	AT1G02610
0.00	-2.91	-2.93	-2.92	0.0022	AT2G30840
0.00	-2.71	-3.14	-2.93	0.0467	AT3G02810
0.00	-2.96	-2.91	-2.94	0.0054	AT2G26410
0.00	-2.84	-3.04	-2.94	0.0216	AT5G12000
0.00	-2.93	-2.97	-2.95	0.0043	AT5G59780
0.00	-3.10	-2.81	-2.96	0.0312	AT4G13450
0.00	-2.90	-3.01	-2.96	0.0118	AT1G66700

0.00	-2.88	-3.05	-2.96	0.0182	AT3G57950
0.00	-2.79	-3.15	-2.97	0.0385	AT3G16530
0.00	-2.89	-3.04	-2.97	0.0161	AT5G40000
0.00	-2.82	-3.14	-2.98	0.0341	AT5G03270
0.00	-3.10	-2.88	-2.99	0.0234	AT2G29460
0.00	-3.22	-2.77	-2.99	0.0477	AT2G39410
0.00	-3.13	-2.89	-3.01	0.0254	AT1G67230
0.00	-3.01	-3.02	-3.02	0.0011	AT5G07100
0.00	-2.91	-3.12	-3.02	0.0222	AT3G50360
0.00	-2.91	-3.13	-3.02	0.0232	AT3G07820
0.00	-2.94	-3.11	-3.03	0.0179	AT1G67000
0.00	-3.20	-2.86	-3.03	0.0357	AT4G13890
0.00	-2.87	-3.20	-3.04	0.0346	AT1G60240
0.00	-2.85	-3.23	-3.04	0.0397	AT5G61590
0.00	-3.06	-3.07	-3.06	0.0010	AT5G52390
0.00	-3.16	-3.00	-3.08	0.0165	AT3G14650
0.00	-3.03	-3.13	-3.08	0.0103	AT3G09940
0.00	-3.24	-2.92	-3.08	0.0330	AT3G44300
0.00	-3.16	-3.01	-3.08	0.0155	AT4G08780
0.00	-3.04	-3.14	-3.09	0.0103	AT3G49620
0.00	-3.20	-2.99	-3.10	0.0216	AT2G39030
0.00	-3.06	-3.14	-3.10	0.0082	AT4G02650
0.00	-2.88	-3.32	-3.10	0.0451	AT4G12830
0.00	-3.12	-3.09	-3.10	0.0031	AT5G64840
0.00	-3.17	-3.10	-3.14	0.0071	AT3G62230
0.00	-3.35	-2.94	-3.15	0.0414	AT4G05030
0.00	-3.37	-2.94	-3.16	0.0433	AT3G53980
0.00	-3.25	-3.07	-3.16	0.0181	AT1G03590
0.00	-3.01	-3.33	-3.17	0.0321	AT2G47810
0.00	-3.08	-3.26	-3.17	0.0181	AT3G45130
0.00	-3.28	-3.06	-3.17	0.0221	AT3G04360
0.00	-3.14	-3.23	-3.18	0.0090	AT1G01680
0.00	-3.29	-3.09	-3.19	0.0200	AT3G13610
0.00	-3.02	-3.39	-3.20	0.0367	AT2G01530
0.00	-3.05	-3.36	-3.21	0.0308	AT3G14640
0.00	-3.08	-3.35	-3.22	0.0267	AT2G30790
0.00	-3.09	-3.37	-3.23	0.0276	AT5G51680
0.00	-3.25	-3.25	-3.25	0.0000	AT1G06160
0.00	-3.38	-3.12	-3.25	0.0255	AT2G17890
0.00	-3.22	-3.33	-3.27	0.0107	AT3G07780
0.00	-3.20	-3.38	-3.29	0.0174	AT1G49270
0.00	-3.30	-3.30	-3.30	0.0000	AT4G37150
0.00	-3.31	-3.31	-3.31	0.0000	AT4G25000
0.00	-3.32	-3.32	-3.32	0.0000	AT1G20920
0.00	-3.18	-3.56	-3.37	0.0359	AT5G53020
0.00	-3.13	-3.65	-3.39	0.0487	AT1G49520
0.00	-3.29	-3.54	-3.42	0.0233	AT5G44400
0.00	-3.17	-3.67	-3.42	0.0465	AT3G28580
0.00	-3.70	-3.18	-3.44	0.0480	AT2G23620
0.00	-3.47	-3.48	-3.48	0.0009	AT1G13650
0.00	-3.58	-3.37	-3.48	0.0192	AT2G36450
0.00	-3.26	-3.71	-3.48	0.0410	AT4G21920
0.00	-3.61	-3.37	-3.49	0.0219	AT4G39675
0.00	-3.44	-3.55	-3.49	0.0100	AT4G20860
0.00	-3.35	-3.65	-3.50	0.0273	AT2G22510
0.00	-3.60	-3.46	-3.53	0.0126	AT5G16500
0.00	-3.60	-3.47	-3.53	0.0117	AT5G41440
0.00	-3.72	-3.38	-3.55	0.0305	AT4G37710
0.00	-3.61	-3.51	-3.56	0.0089	AT5G44380

0.00	-3.71	-3.49	-3.60	0.0194	AT4G21200
0.00	-3.67	-3.80	-3.73	0.0111	AT1G74590
0.00	-3.71	-3.77	-3.74	0.0051	AT2G44990
0.00	-3.99	-3.53	-3.76	0.0389	AT1G31050
0.00	-3.56	-3.99	-3.78	0.0362	AT4G19370
0.00	-3.59	-4.00	-3.79	0.0344	AT1G52810
0.00	-3.71	-3.88	-3.79	0.0143	AT4G25434
0.00	-3.94	-3.88	-3.91	0.0049	AT1G53990
0.00	-3.92	-3.91	-3.92	0.0008	AT1G19530
0.00	-3.97	-3.88	-3.93	0.0073	AT5G06420
0.00	-4.17	-3.70	-3.94	0.0380	AT1G20160
0.00	-3.78	-4.10	-3.94	0.0258	AT2G21730
0.00	-4.12	-3.84	-3.98	0.0224	AT2G38390
0.00	-4.16	-3.84	-4.00	0.0255	AT5G22555
0.00	-4.20	-3.88	-4.04	0.0252	AT2G38380
0.00	-4.10	-3.99	-4.05	0.0087	AT4G32810
0.00	-3.91	-4.34	-4.12	0.0332	AT4G36430
0.00	-4.11	-4.31	-4.21	0.0151	AT1G61810
0.00	-4.00	-4.43	-4.21	0.0324	AT1G64160
0.00	-3.92	-4.54	-4.23	0.0466	AT1G49210
0.00	-4.32	-4.30	-4.31	0.0015	AT5G39100
0.00	-4.38	-4.33	-4.36	0.0037	AT4G38080
0.00	-4.07	-4.71	-4.39	0.0463	AT5G42490
0.00	-4.34	-4.62	-4.48	0.0199	AT2G18370
0.00	-4.65	-4.55	-4.60	0.0069	AT3G05950
0.00	-5.11	-4.41	-4.76	0.0467	AT2G44370
0.00	-4.74	-4.88	-4.81	0.0093	AT4G01985
0.00	-4.72	-4.93	-4.82	0.0139	AT5G37690
0.00	-4.58	-5.11	-4.85	0.0348	AT5G65600
0.00	-4.73	-5.12	-4.93	0.0252	AT4G17220
0.00	-4.94	-4.97	-4.96	0.0019	AT5G09480
0.00	-5.03	-5.24	-5.13	0.0130	AT5G40990
0.00	-5.27	-5.58	-5.42	0.0182	AT2G38600
0.00	-5.56	-5.45	-5.51	0.0064	AT5G58860
0.00	-5.74	-6.18	-5.96	0.0235	AT1G60470

Supplementary material 4: List of up- and down-regulated genes (HE-6D-WL vs HE) with their normalized fold induction and adjusted p value

Normalized Fold OE	Fold OE-6D-WL 1	Fold OE-6D-WL 2	Normalized Fold OE-6D-WL	Adjusted P value	Gene ID
0.00	7.16	7.19	7.17	0.0013	AT5G53680
0.00	7.14	6.75	6.95	0.0179	AT4G21830
0.00	6.46	6.52	6.49	0.0029	AT2G45580
0.00	5.86	6.08	5.97	0.0117	AT3G63380
0.00	5.80	5.99	5.90	0.0103	AT3G11430
0.00	5.90	5.78	5.84	0.0065	AT1G10585
0.00	5.92	5.76	5.84	0.0087	AT1G35310
0.00	5.76	5.50	5.63	0.0147	AT2G39030
0.00	5.59	5.37	5.48	0.0128	AT1G48720
0.00	5.62	5.33	5.48	0.0169	AT5G38910
0.00	5.41	5.17	5.29	0.0144	AT5G23020
0.00	5.36	5.19	5.28	0.0103	AT2G31230
0.00	5.00	5.00	5.00	0.0000	AT2G38240
0.00	4.68	5.24	4.96	0.0359	AT3G28810
0.00	4.93	4.94	4.93	0.0006	AT5G10040
0.00	4.87	4.92	4.89	0.0033	AT5G59720
0.00	4.84	4.93	4.88	0.0059	AT4G10250
0.00	4.90	4.73	4.82	0.0112	AT1G05700
0.00	4.72	4.81	4.77	0.0060	AT5G10625
0.00	4.61	4.89	4.75	0.0188	AT5G65600
0.00	4.68	4.69	4.69	0.0007	AT3G13229
0.00	4.47	4.77	4.62	0.0207	AT1G60470
0.00	4.56	4.59	4.57	0.0021	AT4G37710
0.00	4.21	4.81	4.51	0.0423	AT4G18450
0.00	4.54	4.46	4.50	0.0057	AT1G73260
0.00	4.22	4.76	4.49	0.0382	AT4G13450
0.00	4.64	4.33	4.49	0.0220	AT1G66120
0.00	4.24	4.72	4.48	0.0341	AT1G28305
0.00	4.41	4.30	4.35	0.0080	AT1G43160
0.00	4.57	4.12	4.34	0.0329	AT2G10970
0.00	4.01	4.66	4.34	0.0476	AT4G23200
0.00	4.56	4.08	4.32	0.0353	AT3G03520
0.00	3.96	4.60	4.28	0.0475	AT1G26250
0.00	4.24	4.31	4.27	0.0052	AT2G39518
0.00	4.39	4.12	4.26	0.0202	AT1G18860
0.00	4.19	4.32	4.25	0.0097	AT2G38940
0.00	4.29	4.17	4.23	0.0090	AT5G43580
0.00	4.12	4.30	4.21	0.0136	AT1G01453
0.00	4.08	4.32	4.20	0.0182	AT4G11650
0.00	4.44	3.95	4.20	0.0371	AT1G12940

0.00	3.85	4.39	4.12	0.0417	AT2G47950
0.00	4.23	3.98	4.10	0.0194	AT5G39310
0.00	3.91	4.27	4.09	0.0280	AT2G15780
0.00	4.02	4.09	4.05	0.0055	AT5G07420
0.00	4.15	3.93	4.04	0.0173	AT5G13080
0.00	3.91	4.16	4.03	0.0197	AT1G64200
0.00	4.11	3.92	4.01	0.0151	AT2G34610
0.00	3.83	4.20	4.01	0.0293	AT2G19000
0.00	4.16	3.83	4.00	0.0263	AT3G25240
0.00	3.79	4.18	3.99	0.0311	AT4G28350
0.00	3.73	4.22	3.98	0.0392	AT3G22650
0.00	3.82	4.13	3.98	0.0248	AT4G20860
0.00	4.17	3.75	3.96	0.0337	AT3G12230
0.00	3.88	3.96	3.92	0.0065	AT1G68620
0.00	4.15	3.68	3.92	0.0382	AT1G66550
0.00	3.92	3.91	3.91	0.0008	AT2G15760
0.00	3.73	4.09	3.91	0.0293	AT2G43386
0.00	4.05	3.76	3.91	0.0236	AT1G62760
0.00	3.70	4.05	3.88	0.0287	AT2G16730
0.00	3.82	3.93	3.88	0.0090	AT4G04540
0.00	3.74	4.00	3.87	0.0214	AT5G41590
0.00	3.89	3.84	3.86	0.0041	AT1G32350
0.00	4.03	3.66	3.85	0.0306	AT3G59900
0.00	3.83	3.85	3.84	0.0017	AT5G06740
0.00	3.75	3.92	3.84	0.0141	AT5G56640
0.00	3.55	4.10	3.83	0.0457	AT1G60240
0.00	3.61	4.05	3.83	0.0365	AT1G76640
0.00	3.73	3.89	3.81	0.0134	AT3G53600
0.00	3.57	4.04	3.81	0.0393	AT1G70540
0.00	3.62	3.98	3.80	0.0301	AT4G06746
0.00	3.63	3.96	3.79	0.0277	AT3G52600
0.00	3.71	3.87	3.79	0.0134	AT2G29470
0.00	3.65	3.91	3.78	0.0219	AT5G62750
0.00	3.89	3.66	3.78	0.0194	AT2G47340
0.00	3.77	3.76	3.77	0.0008	AT1G28190
0.00	3.52	4.01	3.76	0.0414	AT2G47030
0.00	3.70	3.78	3.74	0.0068	AT2G28210
0.00	4.03	3.45	3.74	0.0493	AT4G18395
0.00	3.49	3.97	3.73	0.0409	AT5G50830
0.00	3.61	3.76	3.68	0.0130	AT5G15140
0.00	3.80	3.56	3.68	0.0208	AT1G11770
0.00	3.86	3.49	3.67	0.0320	AT1G52580
0.00	3.79	3.55	3.67	0.0208	AT1G71520
0.00	3.74	3.57	3.66	0.0148	AT1G31885
0.00	3.42	3.88	3.65	0.0401	AT5G58170
0.00	3.84	3.44	3.64	0.0349	AT3G10460

0.00	3.82	3.45	3.63	0.0324	AT2G19010
0.00	3.77	3.49	3.63	0.0245	AT1G68150
0.00	3.52	3.74	3.63	0.0193	AT5G27870
0.00	3.60	3.66	3.63	0.0053	AT5G48140
0.00	3.45	3.81	3.63	0.0315	AT5G66985
0.00	3.84	3.39	3.61	0.0396	AT5G54300
0.00	3.79	3.43	3.61	0.0317	AT2G44560
0.00	3.48	3.73	3.60	0.0221	AT3G17980
0.00	3.85	3.36	3.60	0.0432	AT5G51890
0.00	3.52	3.68	3.60	0.0141	AT1G16030
0.00	3.51	3.68	3.59	0.0150	AT5G42490
0.00	3.55	3.64	3.59	0.0080	AT3G46520
0.00	3.67	3.50	3.59	0.0151	AT3G28750
0.00	3.50	3.66	3.58	0.0142	AT1G44414
0.00	3.44	3.71	3.57	0.0240	AT4G33860
0.00	3.46	3.67	3.57	0.0187	AT3G22510
0.00	3.59	3.54	3.57	0.0045	AT1G11765
0.00	3.46	3.67	3.57	0.0187	AT1G50610
0.00	3.35	3.75	3.55	0.0358	AT2G17900
0.00	3.44	3.66	3.55	0.0197	AT4G24640
0.00	3.47	3.63	3.55	0.0143	AT3G05930
0.00	3.47	3.63	3.55	0.0143	AT1G52560
0.00	3.72	3.35	3.54	0.0333	AT3G04360
0.00	3.47	3.59	3.53	0.0108	AT2G19050
0.00	3.43	3.63	3.53	0.0180	AT2G28180
0.00	3.58	3.46	3.52	0.0109	AT3G21570
0.00	3.61	3.39	3.50	0.0200	AT2G17740
0.00	3.31	3.68	3.49	0.0337	AT5G23270
0.00	3.43	3.55	3.49	0.0109	AT1G27020
0.00	3.38	3.60	3.49	0.0201	AT2G46360
0.00	3.43	3.55	3.49	0.0109	AT5G04180
0.00	3.40	3.58	3.49	0.0164	AT3G25165
0.00	3.36	3.60	3.48	0.0219	AT4G37990
0.00	3.42	3.53	3.48	0.0101	AT1G19500
0.00	3.30	3.65	3.48	0.0320	AT1G51250
0.00	3.37	3.57	3.47	0.0183	AT1G11250
0.00	3.39	3.52	3.45	0.0120	AT5G15110
0.00	3.23	3.68	3.45	0.0414	AT5G60740
0.00	3.39	3.51	3.45	0.0111	AT4G27670
0.00	3.44	3.45	3.45	0.0009	AT1G10370
0.00	3.34	3.55	3.44	0.0194	AT3G01230
0.00	3.49	3.40	3.44	0.0083	AT4G09780
0.00	3.37	3.51	3.44	0.0130	AT3G56180
0.00	3.23	3.64	3.44	0.0379	AT2G28640
0.00	3.24	3.63	3.44	0.0361	AT4G15610
0.00	3.70	3.17	3.43	0.0490	AT3G06830

0.00	3.44	3.43	3.43	0.0009	AT3G09940
0.00	3.53	3.33	3.43	0.0186	AT5G26692
0.00	3.54	3.32	3.43	0.0204	AT4G18980
0.00	3.25	3.61	3.43	0.0334	AT4G30140
0.00	3.24	3.62	3.43	0.0352	AT2G26850
0.00	3.20	3.63	3.42	0.0400	AT4G29930
0.00	3.57	3.24	3.40	0.0308	AT5G10500
0.00	3.38	3.42	3.40	0.0037	AT3G18220
0.00	3.32	3.47	3.40	0.0141	AT3G11180
0.00	3.15	3.63	3.39	0.0450	AT1G54070
0.00	3.19	3.58	3.39	0.0366	AT4G21920
0.00	3.17	3.60	3.39	0.0404	AT2G43230
0.00	3.26	3.50	3.38	0.0226	AT1G75930
0.00	3.13	3.62	3.37	0.0461	AT4G12400
0.00	3.16	3.58	3.37	0.0396	AT2G31430
0.00	3.22	3.52	3.37	0.0283	AT1G55560
0.00	3.42	3.32	3.37	0.0094	AT2G21990
0.00	3.26	3.48	3.37	0.0208	AT3G54240
0.00	3.50	3.23	3.37	0.0255	AT5G52720
0.00	3.36	3.36	3.36	0.0000	AT2G26450
0.00	3.30	3.42	3.36	0.0114	AT3G53150
0.00	3.43	3.28	3.36	0.0142	AT2G04220
0.00	3.34	3.37	3.35	0.0028	AT3G36659
0.00	3.35	3.34	3.35	0.0010	AT2G44540
0.00	3.19	3.50	3.35	0.0295	AT3G54800
0.00	3.52	3.16	3.34	0.0343	AT2G02140
0.00	3.32	3.35	3.33	0.0029	AT5G25430
0.00	3.31	3.34	3.32	0.0029	AT2G24850
0.00	3.46	3.19	3.32	0.0258	AT2G16586
0.00	3.07	3.56	3.32	0.0470	AT5G02110
0.00	3.52	3.11	3.31	0.0393	AT4G29740
0.00	3.33	3.29	3.31	0.0038	AT1G16420
0.00	3.05	3.57	3.31	0.0499	AT3G19615
0.00	3.26	3.35	3.31	0.0087	AT3G26860
0.00	3.28	3.33	3.31	0.0048	AT2G47180
0.00	3.27	3.34	3.30	0.0067	AT5G39050
0.00	3.34	3.25	3.30	0.0087	AT4G02250
0.00	3.35	3.24	3.30	0.0106	AT2G33710
0.00	3.16	3.42	3.29	0.0251	AT3G21230
0.00	3.41	3.18	3.29	0.0222	AT2G46300
0.00	3.27	3.30	3.28	0.0029	AT1G30710
0.00	3.51	3.04	3.27	0.0456	AT5G50040
0.00	3.16	3.39	3.27	0.0223	AT3G28830
0.00	3.48	3.06	3.27	0.0408	AT5G37670
0.00	3.08	3.44	3.26	0.0351	AT2G40340
0.00	3.23	3.29	3.26	0.0059	AT1G76370

0.00	3.19	3.32	3.26	0.0127	AT5G26717
0.00	3.22	3.28	3.25	0.0059	AT4G25780
0.00	3.04	3.46	3.25	0.0411	AT4G29658
0.00	3.48	3.02	3.25	0.0450	AT3G25760
0.00	3.39	3.11	3.25	0.0274	AT4G35700
0.00	3.11	3.38	3.25	0.0265	AT2G40850
0.00	3.23	3.25	3.24	0.0020	AT3G46700
0.00	3.20	3.27	3.23	0.0069	AT5G64750
0.00	3.23	3.22	3.23	0.0010	AT3G61230
0.00	3.05	3.40	3.22	0.0345	AT3G25655
0.00	3.46	2.97	3.22	0.0484	AT5G35110
0.00	3.28	3.14	3.21	0.0139	AT1G69940
0.00	3.37	3.06	3.21	0.0307	AT1G02575
0.00	3.02	3.40	3.21	0.0376	AT1G13520
0.00	3.19	3.23	3.21	0.0040	AT5G58050
0.00	2.99	3.42	3.21	0.0426	AT3G25573
0.00	3.19	3.22	3.21	0.0030	AT3G21720
0.00	3.07	3.34	3.20	0.0268	AT3G20580
0.00	3.06	3.35	3.20	0.0288	AT3G02810
0.00	3.34	3.05	3.20	0.0289	AT3G26110
0.00	3.21	3.18	3.19	0.0030	AT4G02650
0.00	3.24	3.14	3.19	0.0100	AT1G64170
0.00	3.18	3.20	3.19	0.0020	AT1G01980
0.00	2.98	3.39	3.18	0.0409	AT1G66210
0.00	3.19	3.15	3.17	0.0040	AT5G48540
0.00	3.05	3.29	3.17	0.0241	AT3G05150
0.00	3.36	2.98	3.17	0.0381	AT5G25340
0.00	3.27	3.06	3.17	0.0211	AT1G01460
0.00	3.16	3.17	3.16	0.0010	AT1G24620
0.00	2.92	3.40	3.16	0.0483	AT4G00940
0.00	3.10	3.22	3.16	0.0121	AT1G74590
0.00	3.31	3.02	3.16	0.0291	AT2G46860
0.00	3.21	3.12	3.16	0.0091	AT5G11920
0.00	3.21	3.11	3.16	0.0101	AT1G26380
0.00	3.32	2.99	3.15	0.0333	AT3G44870
0.00	2.99	3.32	3.15	0.0333	AT5G53050
0.00	2.99	3.32	3.15	0.0333	AT1G53870
0.00	3.04	3.27	3.15	0.0232	AT1G17540
0.00	3.01	3.29	3.15	0.0283	AT3G47780
0.00	3.12	3.18	3.15	0.0061	AT3G20210
0.00	2.97	3.32	3.14	0.0354	AT5G49920
0.00	3.19	3.09	3.14	0.0101	AT1G19610
0.00	3.13	3.15	3.14	0.0020	AT1G54890
0.00	3.13	3.15	3.14	0.0020	AT1G67290
0.00	3.38	2.89	3.14	0.0497	AT4G31970
0.00	3.32	2.96	3.14	0.0365	AT2G40180

0.00	3.02	3.24	3.13	0.0224	AT1G66600
0.00	3.13	3.13	3.13	0.0000	AT4G36490
0.00	2.97	3.29	3.13	0.0325	AT5G02780
0.00	3.14	3.11	3.12	0.0031	AT5G12000
0.00	3.17	3.07	3.12	0.0102	AT5G20390
0.00	3.06	3.17	3.12	0.0112	AT1G49270
0.00	3.15	3.07	3.11	0.0082	AT1G07160
0.00	3.11	3.11	3.11	0.0000	AT2G34500
0.00	3.23	2.98	3.11	0.0256	AT4G14710
0.00	3.28	2.93	3.10	0.0358	AT2G26490
0.00	3.15	3.06	3.10	0.0092	AT1G30473
0.00	2.91	3.29	3.10	0.0390	AT3G27170
0.00	2.97	3.24	3.10	0.0277	AT2G05850
0.00	3.12	3.08	3.10	0.0041	AT4G19810
0.00	3.08	3.12	3.10	0.0041	AT5G48430
0.00	2.90	3.29	3.09	0.0401	AT1G32940
0.00	2.97	3.21	3.09	0.0247	AT3G20530
0.00	2.97	3.21	3.09	0.0247	AT4G26260
0.00	3.03	3.14	3.09	0.0113	AT4G03290
0.00	3.21	2.95	3.08	0.0269	AT5G57510
0.00	3.05	3.11	3.08	0.0062	AT4G00350
0.00	2.98	3.17	3.08	0.0197	AT1G69930
0.00	3.06	3.09	3.08	0.0031	AT1G02090
0.00	3.05	3.09	3.07	0.0041	AT3G17060
0.00	3.03	3.11	3.07	0.0083	AT5G05070
0.00	2.94	3.20	3.07	0.0269	AT5G15450
0.00	3.08	3.05	3.06	0.0031	AT1G01310
0.00	3.14	2.98	3.06	0.0166	AT3G43250
0.00	3.06	3.06	3.06	0.0000	AT4G25200
0.00	3.10	3.01	3.06	0.0094	AT5G55490
0.00	3.10	3.02	3.06	0.0083	AT5G57150
0.00	3.17	2.94	3.05	0.0240	AT4G38190
0.00	3.18	2.92	3.05	0.0271	AT4G33905
0.00	3.03	3.07	3.05	0.0042	AT1G05580
0.00	3.02	3.06	3.04	0.0042	AT1G77530
0.00	2.86	3.20	3.03	0.0357	AT3G53230
0.00	3.00	3.07	3.03	0.0073	AT2G23900
0.00	3.14	2.93	3.03	0.0220	AT3G21080
0.00	2.99	3.06	3.03	0.0074	AT1G10770
0.00	2.92	3.13	3.03	0.0221	AT1G48020
0.00	3.04	3.01	3.03	0.0032	AT3G47760
0.00	3.08	2.97	3.03	0.0116	AT1G61120
0.00	2.81	3.23	3.02	0.0442	AT5G61710
0.00	3.19	2.85	3.02	0.0358	AT3G25170
0.00	3.20	2.84	3.02	0.0379	AT1G61800
0.00	3.21	2.81	3.01	0.0422	AT2G02720

0.00	2.85	3.17	3.01	0.0338	AT1G60760
0.00	3.02	2.99	3.01	0.0032	AT2G45130
0.00	2.84	3.16	3.00	0.0339	AT1G02360
0.00	2.96	3.03	3.00	0.0074	AT3G04700
0.00	2.99	3.00	3.00	0.0011	AT5G45090
0.00	2.79	3.19	2.99	0.0425	AT5G49690
0.00	2.78	3.20	2.99	0.0446	AT4G29460
0.00	3.12	2.86	2.99	0.0277	AT5G59540
0.00	3.11	2.85	2.98	0.0278	AT5G20820
0.00	3.19	2.77	2.98	0.0448	AT1G19250
0.00	2.88	3.08	2.98	0.0214	AT1G08140
0.00	3.05	2.89	2.97	0.0171	AT3G21520
0.00	2.98	2.96	2.97	0.0021	AT3G19310
0.00	3.05	2.89	2.97	0.0171	AT4G19038
0.00	2.91	3.03	2.97	0.0129	AT5G08250
0.00	2.85	3.09	2.97	0.0257	AT4G15980
0.00	3.18	2.75	2.97	0.0461	AT2G29460
0.00	2.92	3.01	2.96	0.0097	AT4G27580
0.00	3.07	2.86	2.96	0.0225	AT1G63245
0.00	3.05	2.87	2.96	0.0194	AT1G14880
0.00	3.01	2.91	2.96	0.0108	AT3G13227
0.00	2.73	3.18	2.95	0.0484	AT4G18170
0.00	2.74	3.16	2.95	0.0452	AT2G02310
0.00	3.03	2.86	2.95	0.0184	AT1G22400
0.00	3.03	2.86	2.94	0.0184	AT3G05725
0.00	2.89	2.99	2.94	0.0108	AT3G05610
0.00	2.80	3.08	2.94	0.0303	AT1G30850
0.00	3.12	2.75	2.94	0.0401	AT1G76070
0.00	2.78	3.09	2.94	0.0336	AT2G33460
0.00	3.03	2.84	2.93	0.0206	AT1G60740
0.00	3.04	2.82	2.93	0.0239	AT5G54095
0.00	2.77	3.09	2.93	0.0347	AT1G07500
0.00	2.78	3.08	2.93	0.0326	AT3G04070
0.00	2.92	2.91	2.92	0.0011	AT5G40990
0.00	2.74	3.09	2.91	0.0382	AT1G55920
0.00	2.78	3.05	2.91	0.0295	AT4G05100
0.00	2.94	2.88	2.91	0.0066	AT2G35980
0.00	2.83	2.99	2.91	0.0175	AT1G71000
0.00	2.96	2.86	2.91	0.0109	AT5G14890
0.00	2.96	2.86	2.91	0.0109	AT3G52010
0.00	3.10	2.71	2.91	0.0427	AT5G57520
0.00	2.95	2.86	2.91	0.0099	AT3G28510
0.00	3.03	2.79	2.91	0.0262	AT5G67080
0.00	2.79	3.01	2.90	0.0241	AT3G62180
0.00	2.69	3.09	2.89	0.0440	AT1G51410
0.00	2.96	2.82	2.89	0.0154	AT5G58770

0.00	2.89	2.89	2.89	0.0000	AT5G09590
0.00	2.89	2.87	2.88	0.0022	AT4G37150
0.00	2.98	2.78	2.88	0.0221	AT1G22810
0.00	2.87	2.90	2.88	0.0033	AT3G20190
0.00	2.91	2.85	2.88	0.0066	AT2G07040
0.00	2.93	2.82	2.88	0.0122	AT5G25450
0.00	2.96	2.79	2.88	0.0188	AT3G55970
0.00	2.98	2.77	2.87	0.0232	AT5G48400
0.00	2.89	2.85	2.87	0.0044	AT5G39880
0.00	2.89	2.84	2.87	0.0056	AT1G35490
0.00	2.83	2.89	2.86	0.0067	AT4G09300
0.00	2.70	3.01	2.86	0.0345	AT2G38340
0.00	2.91	2.81	2.86	0.0111	AT2G29420
0.00	2.78	2.93	2.85	0.0167	AT1G54575
0.00	2.66	3.04	2.85	0.0424	AT1G03050
0.00	2.98	2.72	2.85	0.0290	AT2G39530
0.00	2.65	3.04	2.85	0.0436	AT1G66060
0.00	2.65	3.02	2.84	0.0415	AT3G62640
0.00	2.87	2.79	2.83	0.0090	AT3G44860
0.00	2.67	2.99	2.83	0.0360	AT5G26220
0.00	3.00	2.66	2.83	0.0382	AT1G44160
0.00	2.83	2.82	2.83	0.0011	AT5G54170
0.00	2.69	2.96	2.82	0.0304	AT2G47040
0.00	2.86	2.78	2.82	0.0090	AT5G50030
0.00	2.80	2.85	2.82	0.0056	AT2G33100
0.00	2.64	2.99	2.81	0.0395	AT3G26934
0.00	3.01	2.61	2.81	0.0452	AT1G48820
0.00	2.67	2.94	2.81	0.0306	AT5G42150
0.00	2.71	2.89	2.80	0.0205	AT5G28690
0.00	2.74	2.86	2.80	0.0136	AT3G27331
0.00	2.69	2.91	2.80	0.0250	AT1G17170
0.00	2.89	2.71	2.80	0.0205	AT4G25590
0.00	3.00	2.60	2.80	0.0454	AT1G19025
0.00	2.88	2.71	2.79	0.0194	AT1G44120
0.00	2.58	2.99	2.79	0.0468	AT1G61680
0.00	2.78	2.80	2.79	0.0023	AT1G76690
0.00	2.62	2.95	2.79	0.0377	AT1G68110
0.00	2.83	2.73	2.78	0.0114	AT5G52640
0.00	2.90	2.65	2.78	0.0287	AT4G23493
0.00	2.86	2.69	2.78	0.0195	AT1G23350
0.00	2.60	2.93	2.77	0.0379	AT1G09935
0.00	2.70	2.84	2.77	0.0161	AT3G29250
0.00	2.75	2.78	2.76	0.0035	AT4G35170
0.00	2.96	2.56	2.76	0.0461	AT3G19020
0.00	2.54	2.97	2.75	0.0496	AT4G34530
0.00	2.92	2.58	2.75	0.0393	AT1G68610

0.00	2.75	2.75	2.75	0.0000	AT4G01895
0.00	2.81	2.69	2.75	0.0139	AT3G15500
0.00	2.89	2.61	2.75	0.0324	AT2G31500
0.00	2.78	2.71	2.75	0.0081	AT4G23700
0.00	2.60	2.90	2.75	0.0347	AT3G03800
0.00	2.65	2.85	2.75	0.0231	AT5G51680
0.00	2.81	2.68	2.75	0.0151	AT1G13890
0.00	2.70	2.78	2.74	0.0093	AT4G38060
0.00	2.87	2.61	2.74	0.0302	AT5G51440
0.00	2.66	2.81	2.74	0.0175	AT1G11100
0.00	2.72	2.75	2.73	0.0035	AT1G76680
0.00	2.74	2.73	2.73	0.0012	AT5G15250
0.00	2.72	2.73	2.72	0.0012	AT2G44240
0.00	2.73	2.71	2.72	0.0023	AT1G49520
0.00	2.75	2.68	2.72	0.0082	AT2G25600
0.00	2.82	2.60	2.71	0.0258	AT1G02390
0.00	2.70	2.72	2.71	0.0023	AT2G25140
0.00	2.62	2.80	2.71	0.0211	AT3G47440
0.00	2.70	2.71	2.71	0.0012	AT3G09520
0.00	2.59	2.82	2.70	0.0270	AT5G45880
0.00	2.52	2.88	2.70	0.0424	AT1G30475
0.00	2.65	2.75	2.70	0.0118	AT2G29410
0.00	2.75	2.64	2.70	0.0130	AT1G74010
0.00	2.86	2.53	2.70	0.0389	AT3G01700
0.00	2.51	2.89	2.70	0.0447	AT5G62660
0.00	2.79	2.60	2.69	0.0224	AT2G19190
0.00	2.77	2.61	2.69	0.0189	AT5G47000
0.00	2.75	2.63	2.69	0.0142	AT4G28290
0.00	2.71	2.65	2.68	0.0071	AT1G17870
0.00	2.55	2.80	2.68	0.0297	AT1G01750
0.00	2.75	2.59	2.67	0.0191	AT1G79400
0.00	2.59	2.75	2.67	0.0191	AT5G15600
0.00	2.71	2.63	2.67	0.0095	AT5G61680
0.00	2.79	2.54	2.67	0.0298	AT1G65610
0.00	2.87	2.46	2.67	0.0489	AT1G72510
0.00	2.66	2.67	2.66	0.0012	AT1G70140
0.00	2.72	2.61	2.66	0.0131	AT5G07430
0.00	2.53	2.78	2.66	0.0300	AT1G67000
0.00	2.68	2.62	2.65	0.0072	AT1G62730
0.00	2.65	2.65	2.65	0.0000	AT1G12060
0.00	2.67	2.62	2.65	0.0060	AT5G19473
0.00	2.45	2.84	2.64	0.0468	AT3G48520
0.00	2.74	2.54	2.64	0.0241	AT3G57690
0.00	2.50	2.79	2.64	0.0349	AT2G18210
0.00	2.56	2.72	2.64	0.0193	AT2G28490
0.00	2.51	2.76	2.63	0.0302	AT3G06260

0.00	2.76	2.50	2.63	0.0314	AT4G39610
0.00	2.68	2.57	2.63	0.0133	AT1G08310
0.00	2.50	2.75	2.63	0.0303	AT2G47050
0.00	2.73	2.52	2.63	0.0255	AT3G26830
0.00	2.51	2.74	2.62	0.0279	AT2G17050
0.00	2.71	2.53	2.62	0.0219	AT5G36220
0.00	2.76	2.48	2.62	0.0340	AT5G48570
0.00	2.72	2.52	2.62	0.0243	AT5G55970
0.00	2.55	2.66	2.61	0.0134	AT2G35765
0.00	2.71	2.49	2.60	0.0269	AT3G28210
0.00	2.63	2.56	2.60	0.0086	AT2G27180
0.00	2.77	2.42	2.59	0.0429	AT5G25350
0.00	2.42	2.76	2.59	0.0417	AT2G22180
0.00	2.51	2.67	2.59	0.0197	AT1G48000
0.00	2.64	2.54	2.59	0.0123	AT4G21120
0.00	2.54	2.64	2.59	0.0123	AT5G02750
0.00	2.58	2.59	2.59	0.0012	AT4G21850
0.00	2.47	2.70	2.58	0.0283	AT1G78340
0.00	2.54	2.62	2.58	0.0099	AT3G09960
0.00	2.71	2.43	2.57	0.0346	AT2G33270
0.00	2.58	2.55	2.57	0.0037	AT1G04470
0.00	2.62	2.50	2.56	0.0149	AT2G42005
0.00	2.63	2.49	2.56	0.0174	AT1G14080
0.00	2.60	2.51	2.56	0.0112	AT3G18250
0.00	2.41	2.69	2.55	0.0349	AT4G32810
0.00	2.45	2.65	2.55	0.0250	AT1G01940
0.00	2.45	2.65	2.55	0.0250	AT5G10410
0.00	2.50	2.59	2.55	0.0113	AT4G12545
0.00	2.51	2.58	2.55	0.0088	AT5G56840
0.00	2.57	2.52	2.55	0.0063	AT2G22340
0.00	2.34	2.74	2.54	0.0500	AT5G51060
0.00	2.71	2.37	2.54	0.0425	AT2G29490
0.00	2.56	2.50	2.53	0.0075	AT1G02920
0.00	2.51	2.56	2.53	0.0063	AT4G04510
0.00	2.70	2.35	2.53	0.0441	AT5G16500
0.00	2.51	2.54	2.53	0.0038	AT3G08970
0.00	2.60	2.45	2.52	0.0189	AT3G43120
0.00	2.53	2.51	2.52	0.0025	AT3G52450
0.00	2.48	2.56	2.52	0.0101	AT1G03070
0.00	2.54	2.50	2.52	0.0051	AT4G15420
0.00	2.44	2.59	2.51	0.0190	AT1G13970
0.00	2.41	2.61	2.51	0.0253	AT4G36990
0.00	2.35	2.66	2.50	0.0393	AT2G32530
0.00	2.33	2.67	2.50	0.0432	AT1G73165
0.00	2.48	2.52	2.50	0.0051	AT1G30070
0.00	2.52	2.47	2.50	0.0064	AT5G13150

0.00	2.50	2.49	2.50	0.0013	AT4G35180
0.00	2.62	2.37	2.50	0.0319	AT5G60260
0.00	2.34	2.65	2.49	0.0395	AT3G10320
0.00	2.61	2.37	2.49	0.0307	AT2G26410
0.00	2.41	2.56	2.48	0.0192	AT1G63930
0.00	2.58	2.37	2.47	0.0270	AT1G11720
0.00	2.58	2.35	2.47	0.0297	AT1G51890
0.00	2.44	2.48	2.46	0.0052	AT5G26700
0.00	2.40	2.52	2.46	0.0155	AT1G61610
0.00	2.59	2.32	2.46	0.0350	AT2G02320
0.00	2.38	2.53	2.46	0.0194	AT1G77450
0.00	2.46	2.45	2.45	0.0013	AT4G17250
0.00	2.31	2.60	2.45	0.0376	AT2G29950
0.00	2.58	2.33	2.45	0.0324	AT2G04070
0.00	2.53	2.37	2.45	0.0208	AT5G16980
0.00	2.42	2.48	2.45	0.0078	AT1G66700
0.00	2.45	2.45	2.45	0.0000	AT4G08170
0.00	2.51	2.38	2.45	0.0169	AT4G12500
0.00	2.33	2.56	2.44	0.0299	AT5G45810
0.00	2.48	2.40	2.44	0.0104	AT2G17500
0.00	2.25	2.62	2.44	0.0483	AT5G60300
0.00	2.55	2.32	2.43	0.0300	AT5G12340
0.00	2.49	2.38	2.43	0.0144	AT1G22985
0.00	2.53	2.33	2.43	0.0262	AT4G18430
0.00	2.51	2.33	2.42	0.0237	AT5G39520
0.00	2.39	2.46	2.42	0.0092	AT3G48450
0.00	2.26	2.58	2.42	0.0420	AT2G32830
0.00	2.42	2.42	2.42	0.0000	AT3G62230
0.00	2.49	2.35	2.42	0.0184	AT5G16010
0.00	2.43	2.41	2.42	0.0026	AT1G62420
0.00	2.27	2.56	2.42	0.0382	AT2G30770
0.00	2.49	2.35	2.42	0.0184	AT3G48850
0.00	2.53	2.28	2.41	0.0331	AT1G13360
0.00	2.30	2.50	2.40	0.0265	AT5G48410
0.00	2.51	2.29	2.40	0.0292	AT2G45210
0.00	2.45	2.34	2.40	0.0146	AT2G39410
0.00	2.45	2.33	2.39	0.0160	AT2G44940
0.00	2.35	2.41	2.38	0.0080	AT5G44920
0.00	2.36	2.39	2.38	0.0040	AT2G01920
0.00	2.19	2.55	2.37	0.0483	AT3G04710
0.00	2.37	2.36	2.37	0.0013	AT3G42640
0.00	2.33	2.40	2.36	0.0094	AT4G19760
0.00	2.23	2.48	2.36	0.0338	AT2G30840
0.00	2.50	2.22	2.36	0.0377	AT2G33420
0.00	2.42	2.27	2.34	0.0204	AT4G09100
0.00	2.42	2.27	2.34	0.0204	AT1G53990

0.00	2.41	2.28	2.34	0.0176	AT1G51920
0.00	2.26	2.42	2.34	0.0218	AT1G17020
0.00	2.38	2.30	2.34	0.0109	AT5G13220
0.00	2.16	2.52	2.34	0.0489	AT5G37340
0.00	2.18	2.49	2.34	0.0422	AT2G43000
0.00	2.32	2.35	2.34	0.0041	AT3G47420
0.00	2.44	2.23	2.33	0.0286	AT5G03630
0.00	2.30	2.36	2.33	0.0082	AT4G03320
0.00	2.28	2.38	2.33	0.0137	AT5G38900
0.00	2.23	2.43	2.33	0.0273	AT4G27410
0.00	2.30	2.36	2.33	0.0082	AT2G32120
0.00	2.45	2.20	2.33	0.0342	AT1G66510
0.00	2.41	2.23	2.32	0.0247	AT3G17790
0.00	2.46	2.19	2.32	0.0369	AT1G03780
0.00	2.34	2.30	2.32	0.0055	AT2G43018
0.00	2.29	2.34	2.32	0.0069	AT5G04610
0.00	2.28	2.35	2.31	0.0096	AT2G03850
0.00	2.39	2.24	2.31	0.0206	AT3G23220
0.00	2.29	2.34	2.31	0.0069	AT3G59940
0.00	2.48	2.15	2.31	0.0453	AT4G12410
0.00	2.31	2.30	2.30	0.0014	AT5G01550
0.00	2.34	2.27	2.30	0.0097	AT1G55570
0.00	2.26	2.35	2.30	0.0124	AT5G49700
0.00	2.34	2.26	2.30	0.0111	AT3G01630
0.00	2.30	2.30	2.30	0.0000	AT5G44990
0.00	2.47	2.13	2.30	0.0470	AT1G32970
0.00	2.35	2.25	2.30	0.0138	AT2G35730
0.00	2.15	2.44	2.30	0.0402	AT3G52080
0.00	2.32	2.27	2.30	0.0069	AT1G09480
0.00	2.22	2.37	2.30	0.0208	AT4G33467
0.00	2.31	2.28	2.29	0.0042	AT2G46240
0.00	2.28	2.30	2.29	0.0028	AT3G62170
0.00	2.12	2.45	2.28	0.0459	AT3G18950
0.00	2.43	2.13	2.28	0.0418	AT4G13560
0.00	2.26	2.30	2.28	0.0056	AT1G47890
0.00	2.32	2.25	2.28	0.0098	AT2G21640
0.00	2.18	2.37	2.28	0.0266	AT4G26120
0.00	2.24	2.31	2.27	0.0098	AT4G21840
0.00	2.31	2.24	2.27	0.0098	AT3G21180
0.00	2.36	2.18	2.27	0.0252	AT5G14380
0.00	2.26	2.27	2.27	0.0014	AT4G27790
0.00	2.19	2.34	2.26	0.0211	AT3G22600
0.00	2.38	2.14	2.26	0.0338	AT1G43800
0.00	2.31	2.21	2.26	0.0141	AT1G68630
0.00	2.21	2.31	2.26	0.0141	AT2G29440
0.00	2.37	2.14	2.26	0.0324	AT3G55890

0.00	2.10	2.41	2.25	0.0437	AT1G15380
0.00	2.25	2.25	2.25	0.0000	AT1G76930
0.00	2.17	2.31	2.24	0.0199	AT3G52460
0.00	2.20	2.28	2.24	0.0114	AT3G24620
0.00	2.19	2.28	2.24	0.0128	AT1G61860
0.00	2.35	2.13	2.24	0.0312	AT1G63910
0.00	2.33	2.14	2.23	0.0270	AT2G35210
0.00	2.25	2.22	2.23	0.0043	AT2G22055
0.00	2.23	2.24	2.23	0.0014	AT5G04890
0.00	2.07	2.40	2.23	0.0469	AT5G13330
0.00	2.29	2.17	2.23	0.0171	AT3G21700
0.00	2.28	2.18	2.23	0.0143	AT4G14630
0.00	2.37	2.08	2.23	0.0414	AT5G03610
0.00	2.28	2.17	2.23	0.0157	AT1G76080
0.00	2.33	2.11	2.22	0.0315	AT3G51910
0.00	2.18	2.26	2.22	0.0115	AT5G50760
0.00	2.27	2.16	2.21	0.0158	AT2G25470
0.00	2.25	2.18	2.21	0.0101	AT3G13390
0.00	2.19	2.23	2.21	0.0058	AT2G36690
0.00	2.28	2.14	2.21	0.0202	AT3G09440
0.00	2.13	2.28	2.20	0.0216	AT1G68390
0.00	2.13	2.27	2.20	0.0202	AT3G49580
0.00	2.07	2.33	2.20	0.0376	AT1G15520
0.00	2.29	2.11	2.20	0.0260	AT2G45610
0.00	2.37	2.03	2.20	0.0491	AT2G42360
0.00	2.16	2.23	2.20	0.0102	AT3G22620
0.00	2.29	2.08	2.19	0.0306	AT3G05140
0.00	2.17	2.20	2.18	0.0044	AT3G46690
0.00	2.28	2.08	2.18	0.0292	AT5G19875
0.00	2.13	2.22	2.18	0.0132	AT3G02410
0.00	2.33	2.02	2.18	0.0453	AT2G23770
0.00	2.29	2.06	2.18	0.0336	AT3G51660
0.00	2.12	2.23	2.17	0.0161	AT1G61290
0.00	2.24	2.11	2.17	0.0190	AT5G45820
0.00	2.25	2.09	2.17	0.0235	AT3G01240
0.00	2.26	2.08	2.17	0.0264	AT5G64510
0.00	2.00	2.33	2.17	0.0484	AT3G12910
0.00	2.03	2.29	2.16	0.0383	AT4G36050
0.00	2.03	2.28	2.16	0.0369	AT4G11170
0.00	2.32	1.99	2.16	0.0486	AT2G02010
0.00	2.15	2.16	2.15	0.0015	AT3G09405
0.00	2.19	2.11	2.15	0.0118	AT1G17615
0.00	2.29	2.00	2.15	0.0430	AT1G72416
0.00	2.07	2.21	2.14	0.0208	AT4G15130
0.00	2.28	2.00	2.14	0.0416	AT4G04078
0.00	2.21	2.07	2.14	0.0208	AT1G69880

0.00	2.10	2.18	2.14	0.0119	AT5G36970
0.00	2.19	2.07	2.13	0.0179	AT1G30700
0.00	2.26	1.99	2.13	0.0404	AT3G01270
0.00	2.21	2.04	2.13	0.0255	AT3G13310
0.00	2.29	1.96	2.13	0.0493	AT1G67920
0.00	2.04	2.20	2.12	0.0240	AT3G16530
0.00	2.19	2.04	2.12	0.0226	AT5G64790
0.00	2.18	2.04	2.11	0.0211	AT1G07350
0.00	2.15	2.08	2.11	0.0105	AT3G56200
0.00	2.03	2.19	2.11	0.0241	AT3G02150
0.00	2.00	2.22	2.11	0.0332	AT1G78380
0.00	2.15	2.06	2.11	0.0136	AT3G07090
0.00	2.16	2.06	2.11	0.0151	AT5G51740
0.00	2.11	2.10	2.11	0.0015	AT5G55090
0.00	2.27	1.94	2.10	0.0498	AT3G18773
0.00	2.18	2.03	2.10	0.0227	AT2G30480
0.00	2.01	2.20	2.10	0.0287	AT5G55930
0.00	1.99	2.21	2.10	0.0333	AT1G26770
0.00	2.01	2.18	2.09	0.0258	AT3G50360
0.00	2.00	2.17	2.09	0.0259	AT3G62590
0.00	1.95	2.22	2.09	0.0412	AT4G13180
0.00	1.96	2.21	2.08	0.0381	AT5G19580
0.00	2.12	2.03	2.08	0.0138	AT5G60270
0.00	2.20	1.95	2.08	0.0383	AT3G01085
0.00	2.16	1.99	2.07	0.0261	AT2G26560
0.00	2.12	2.03	2.07	0.0138	AT5G27520
0.00	2.18	1.96	2.07	0.0338	AT3G11170
0.00	2.15	1.98	2.06	0.0262	AT3G16330
0.00	2.04	2.08	2.06	0.0062	AT1G18350
0.00	2.10	2.01	2.06	0.0139	AT2G47000
0.00	2.15	1.96	2.05	0.0294	AT1G33030
0.00	2.04	2.07	2.05	0.0046	AT4G25380
0.00	2.04	2.06	2.05	0.0031	AT3G16040
0.00	2.06	2.04	2.05	0.0031	AT1G10140
0.00	2.00	2.10	2.05	0.0155	AT5G13490
0.00	1.89	2.21	2.05	0.0496	AT2G16060
0.00	1.91	2.18	2.05	0.0420	AT1G29280
0.00	1.97	2.12	2.04	0.0233	AT5G03350
0.00	2.20	1.89	2.04	0.0482	AT2G19310
0.00	2.19	1.89	2.04	0.0467	AT1G55790
0.00	2.02	2.06	2.04	0.0062	AT3G01180
0.00	2.01	2.07	2.04	0.0094	AT5G22570
0.00	2.05	2.02	2.03	0.0047	AT2G15480
0.00	1.95	2.11	2.03	0.0251	AT3G55870
0.00	1.89	2.17	2.03	0.0438	AT3G01620
0.00	2.13	1.93	2.03	0.0313	AT5G02500

0.00	2.13	1.93	2.03	0.0313	AT5G42020
0.00	1.98	2.07	2.02	0.0141	AT1G30050
0.00	1.89	2.16	2.02	0.0424	AT1G76190
0.00	1.91	2.14	2.02	0.0361	AT2G35070
0.00	2.00	2.04	2.02	0.0063	AT4G04020
0.00	2.00	2.03	2.02	0.0047	AT3G63010
0.00	1.98	2.05	2.01	0.0111	AT2G27690
0.00	2.02	2.01	2.01	0.0016	AT3G22370
0.00	1.95	2.07	2.01	0.0190	AT3G22235
0.00	1.97	2.05	2.01	0.0127	AT4G28550
0.00	2.15	1.87	2.01	0.0443	AT5G47830
0.00	1.93	2.08	2.01	0.0238	AT5G56760
0.00	2.02	1.98	2.00	0.0064	AT4G32510
0.00	1.86	2.14	2.00	0.0445	AT5G24080
0.00	2.04	1.96	2.00	0.0127	AT4G26780
0.00	2.13	1.86	2.00	0.0430	AT1G44350
0.00	1.93	2.07	2.00	0.0223	AT1G60730
0.00	-1.92	-2.07	-2.00	0.0239	AT1G77200
0.00	-2.12	-1.88	-2.00	0.0382	AT1G33610
0.00	-2.04	-1.97	-2.00	0.0111	AT5G23280
0.00	-2.11	-1.90	-2.01	0.0333	AT1G58270
0.00	-1.96	-2.05	-2.01	0.0143	AT1G64760
0.00	-2.05	-1.97	-2.01	0.0127	AT5G56490
0.00	-1.92	-2.10	-2.01	0.0285	AT3G28220
0.00	-1.96	-2.06	-2.01	0.0158	AT2G19620
0.00	-1.89	-2.14	-2.01	0.0394	AT2G45050
0.00	-2.13	-1.90	-2.01	0.0363	AT4G28720
0.00	-2.08	-1.95	-2.02	0.0205	AT2G30150
0.00	-2.07	-1.97	-2.02	0.0158	AT4G08874
0.00	-1.97	-2.07	-2.02	0.0158	AT5G58400
0.00	-1.95	-2.09	-2.02	0.0221	AT3G13175
0.00	-1.91	-2.14	-2.02	0.0361	AT2G40530
0.00	-2.03	-2.03	-2.03	0.0000	AT2G42200
0.00	-2.14	-1.92	-2.03	0.0345	AT2G42540
0.00	-2.12	-1.94	-2.03	0.0282	AT1G30740
0.00	-2.13	-1.94	-2.03	0.0297	AT5G43020
0.00	-2.11	-1.96	-2.03	0.0235	AT4G03330
0.00	-1.90	-2.17	-2.04	0.0422	AT1G77060
0.00	-2.09	-2.01	-2.05	0.0124	AT1G03170
0.00	-2.20	-1.90	-2.05	0.0465	AT1G08080
0.00	-1.90	-2.20	-2.05	0.0465	AT3G48360
0.00	-2.00	-2.10	-2.05	0.0155	AT3G44350
0.00	-1.94	-2.17	-2.05	0.0356	AT4G16980
0.00	-1.96	-2.16	-2.06	0.0309	AT3G53232
0.00	-2.01	-2.13	-2.07	0.0184	AT1G70390
0.00	-1.99	-2.16	-2.07	0.0261	AT4G02200

0.00	-1.99	-2.15	-2.07	0.0246	AT3G10080
0.00	-2.09	-2.06	-2.07	0.0046	AT2G26190
0.00	-2.15	-2.00	-2.08	0.0230	AT2G39855
0.00	-2.00	-2.15	-2.08	0.0230	AT3G51060
0.00	-2.08	-2.08	-2.08	0.0000	AT1G74450
0.00	-2.04	-2.13	-2.08	0.0137	AT5G19530
0.00	-2.08	-2.09	-2.08	0.0015	AT4G22010
0.00	-2.16	-2.01	-2.08	0.0229	AT4G02290
0.00	-2.09	-2.09	-2.09	0.0000	AT3G19380
0.00	-1.97	-2.21	-2.09	0.0365	AT1G27890
0.00	-1.96	-2.22	-2.09	0.0395	AT3G10570
0.00	-2.13	-2.05	-2.09	0.0122	AT5G07030
0.00	-2.09	-2.11	-2.10	0.0030	AT1G72920
0.00	-2.22	-1.99	-2.11	0.0347	AT2G34060
0.00	-1.97	-2.25	-2.11	0.0422	AT1G01380
0.00	-2.02	-2.21	-2.11	0.0286	AT5G61480
0.00	-2.15	-2.11	-2.13	0.0060	AT5G03960
0.00	-2.06	-2.19	-2.13	0.0195	AT1G47655
0.00	-2.05	-2.21	-2.13	0.0239	AT1G01200
0.00	-2.03	-2.25	-2.14	0.0327	AT1G15760
0.00	-2.14	-2.14	-2.14	0.0000	AT3G57920
0.00	-2.00	-2.27	-2.14	0.0402	AT2G15300
0.00	-2.21	-2.08	-2.15	0.0193	AT2G22190
0.00	-2.23	-2.07	-2.15	0.0237	AT3G27200
0.00	-2.19	-2.12	-2.15	0.0103	AT1G69120
0.00	-2.02	-2.29	-2.16	0.0398	AT5G21960
0.00	-2.10	-2.21	-2.16	0.0162	AT4G12690
0.00	-2.11	-2.22	-2.16	0.0162	AT3G24450
0.00	-2.09	-2.24	-2.16	0.0220	AT1G52000
0.00	-2.13	-2.21	-2.17	0.0117	AT1G22550
0.00	-2.12	-2.23	-2.17	0.0161	AT3G07540
0.00	-2.05	-2.34	-2.20	0.0420	AT5G12900
0.00	-2.06	-2.36	-2.21	0.0431	AT5G15580
0.00	-2.13	-2.29	-2.21	0.0230	AT1G65900
0.00	-2.20	-2.22	-2.21	0.0029	AT3G24520
0.00	-2.21	-2.23	-2.22	0.0029	AT1G70740
0.00	-2.17	-2.27	-2.22	0.0143	AT1G10380
0.00	-2.29	-2.16	-2.23	0.0186	AT5G16190
0.00	-2.26	-2.20	-2.23	0.0086	AT4G28703
0.00	-2.23	-2.24	-2.24	0.0014	AT2G36570
0.00	-2.28	-2.21	-2.24	0.0099	AT1G06830
0.00	-2.14	-2.36	-2.25	0.0311	AT1G27770
0.00	-2.36	-2.13	-2.25	0.0326	AT5G19190
0.00	-2.08	-2.43	-2.25	0.0493	AT5G45530
0.00	-2.11	-2.41	-2.26	0.0422	AT2G01420
0.00	-2.17	-2.35	-2.26	0.0253	AT3G48550

0.00	-2.22	-2.30	-2.26	0.0113	AT1G27820
0.00	-2.22	-2.30	-2.26	0.0113	AT2G46780
0.00	-2.26	-2.27	-2.26	0.0014	AT2G21050
0.00	-2.25	-2.27	-2.26	0.0028	AT1G45207
0.00	-2.33	-2.20	-2.26	0.0183	AT5G34830
0.00	-2.27	-2.27	-2.27	0.0000	AT2G28950
0.00	-2.28	-2.26	-2.27	0.0028	AT4G13600
0.00	-2.27	-2.29	-2.28	0.0028	AT3G57270
0.00	-2.26	-2.31	-2.29	0.0070	AT5G40630
0.00	-2.21	-2.37	-2.29	0.0222	AT3G01490
0.00	-2.25	-2.34	-2.29	0.0125	AT4G02130
0.00	-2.21	-2.39	-2.30	0.0249	AT1G41830
0.00	-2.22	-2.42	-2.32	0.0274	AT4G02850
0.00	-2.30	-2.34	-2.32	0.0055	AT5G20740
0.00	-2.21	-2.43	-2.32	0.0302	AT4G02170
0.00	-2.22	-2.42	-2.32	0.0274	AT5G03840
0.00	-2.36	-2.29	-2.32	0.0096	AT3G56880
0.00	-2.30	-2.38	-2.34	0.0109	AT1G56430
0.00	-2.28	-2.40	-2.34	0.0163	AT3G03030
0.00	-2.44	-2.25	-2.35	0.0258	AT1G11850
0.00	-2.21	-2.49	-2.35	0.0379	AT5G16030
0.00	-2.31	-2.41	-2.36	0.0135	AT3G48100
0.00	-2.32	-2.40	-2.36	0.0108	AT5G44630
0.00	-2.29	-2.45	-2.37	0.0215	AT5G47380
0.00	-2.46	-2.28	-2.37	0.0242	AT5G51870
0.00	-2.36	-2.38	-2.37	0.0027	AT1G63860
0.00	-2.34	-2.41	-2.38	0.0094	AT4G16140
0.00	-2.36	-2.40	-2.38	0.0053	AT2G42800
0.00	-2.47	-2.29	-2.38	0.0241	AT1G33440
0.00	-2.21	-2.55	-2.38	0.0454	AT1G75500
0.00	-2.42	-2.35	-2.38	0.0093	AT1G05835
0.00	-2.42	-2.35	-2.38	0.0093	AT1G56240
0.00	-2.36	-2.41	-2.39	0.0067	AT4G26890
0.00	-2.32	-2.46	-2.39	0.0186	AT1G70370
0.00	-2.48	-2.31	-2.40	0.0226	AT1G74440
0.00	-2.23	-2.57	-2.40	0.0450	AT5G55750
0.00	-2.42	-2.38	-2.40	0.0053	AT1G72230
0.00	-2.33	-2.47	-2.40	0.0186	AT1G49830
0.00	-2.46	-2.34	-2.40	0.0159	AT4G38400
0.00	-2.58	-2.23	-2.40	0.0462	AT5G17260
0.00	-2.22	-2.59	-2.41	0.0489	AT1G11500
0.00	-2.56	-2.26	-2.41	0.0396	AT4G15670
0.00	-2.42	-2.41	-2.41	0.0013	AT2G47780
0.00	-2.31	-2.52	-2.41	0.0277	AT1G78400
0.00	-2.34	-2.52	-2.43	0.0236	AT3G60670
0.00	-2.39	-2.48	-2.43	0.0118	AT3G12610

0.00	-2.39	-2.48	-2.44	0.0118	AT1G77760
0.00	-2.34	-2.55	-2.44	0.0273	AT3G05860
0.00	-2.41	-2.47	-2.44	0.0078	AT1G04330
0.00	-2.27	-2.62	-2.45	0.0455	AT1G52770
0.00	-2.29	-2.62	-2.46	0.0427	AT1G73550
0.00	-2.46	-2.45	-2.46	0.0013	AT1G66180
0.00	-2.30	-2.63	-2.46	0.0426	AT1G79760
0.00	-2.36	-2.57	-2.46	0.0271	AT2G41010
0.00	-2.63	-2.30	-2.47	0.0426	AT5G67450
0.00	-2.34	-2.60	-2.47	0.0335	AT4G01130
0.00	-2.57	-2.38	-2.47	0.0244	AT5G08330
0.00	-2.47	-2.49	-2.48	0.0026	AT5G48900
0.00	-2.49	-2.47	-2.48	0.0026	AT5G25460
0.00	-2.42	-2.56	-2.49	0.0179	AT1G75920
0.00	-2.42	-2.55	-2.49	0.0166	AT1G72910
0.00	-2.41	-2.57	-2.49	0.0204	AT1G23340
0.00	-2.60	-2.38	-2.49	0.0281	AT4G00770
0.00	-2.40	-2.58	-2.49	0.0230	AT2G40610
0.00	-2.54	-2.44	-2.49	0.0128	AT1G27670
0.00	-2.40	-2.59	-2.50	0.0242	AT4G17280
0.00	-2.46	-2.53	-2.50	0.0089	AT1G49500
0.00	-2.39	-2.62	-2.50	0.0292	AT1G33170
0.00	-2.46	-2.56	-2.51	0.0127	AT3G45710
0.00	-2.68	-2.34	-2.51	0.0431	AT4G20670
0.00	-2.43	-2.59	-2.51	0.0203	AT5G40910
0.00	-2.34	-2.70	-2.52	0.0454	AT4G14650
0.00	-2.48	-2.56	-2.52	0.0101	AT5G61850
0.00	-2.70	-2.35	-2.52	0.0441	AT3G45960
0.00	-2.37	-2.69	-2.53	0.0402	AT5G40330
0.00	-2.47	-2.59	-2.53	0.0151	AT2G29290
0.00	-2.61	-2.46	-2.53	0.0188	AT5G55470
0.00	-2.51	-2.61	-2.56	0.0124	AT1G67750
0.00	-2.57	-2.54	-2.56	0.0037	AT4G11000
0.00	-2.71	-2.41	-2.56	0.0373	AT3G12890
0.00	-2.38	-2.76	-2.57	0.0470	AT1G54820
0.00	-2.60	-2.63	-2.61	0.0037	AT1G01600
0.00	-2.64	-2.65	-2.64	0.0012	AT2G43610
0.00	-2.57	-2.72	-2.65	0.0180	AT2G01950
0.00	-2.50	-2.84	-2.67	0.0405	AT5G48490
0.00	-2.51	-2.84	-2.68	0.0392	AT5G62340
0.00	-2.60	-2.77	-2.69	0.0201	AT2G30432
0.00	-2.66	-2.71	-2.69	0.0059	AT4G34220
0.00	-2.73	-2.66	-2.70	0.0083	AT5G62040
0.00	-2.68	-2.74	-2.71	0.0070	AT1G09750
0.00	-2.84	-2.60	-2.72	0.0281	AT3G46130
0.00	-2.90	-2.55	-2.73	0.0408	AT1G01130

0.00	-2.83	-2.62	-2.73	0.0245	AT3G29410
0.00	-2.52	-2.95	-2.73	0.0499	AT3G58850
0.00	-2.94	-2.54	-2.74	0.0464	AT2G33000
0.00	-2.79	-2.71	-2.75	0.0093	AT1G04800
0.00	-2.72	-2.78	-2.75	0.0069	AT3G45650
0.00	-2.80	-2.71	-2.76	0.0104	AT4G17490
0.00	-2.79	-2.76	-2.78	0.0034	AT5G15265
0.00	-2.61	-2.97	-2.79	0.0410	AT4G19230
0.00	-2.86	-2.81	-2.83	0.0056	AT3G10720
0.00	-2.80	-2.90	-2.85	0.0112	AT3G01550
0.00	-2.67	-3.03	-2.85	0.0402	AT4G32717
0.00	-2.89	-2.88	-2.88	0.0011	AT5G15780
0.00	-3.06	-2.79	-2.92	0.0294	AT5G12235
0.00	-2.85	-3.00	-2.92	0.0163	AT2G28410
0.00	-2.87	-3.02	-2.94	0.0162	AT5G04150
0.00	-2.72	-3.18	-2.95	0.0495	AT3G18050
0.00	-2.97	-2.93	-2.95	0.0043	AT4G15690
0.00	-3.06	-2.98	-3.02	0.0084	AT2G35640
0.00	-3.06	-3.00	-3.03	0.0063	AT2G47930
0.00	-3.25	-2.82	-3.04	0.0450	AT3G52810
0.00	-2.85	-3.24	-3.04	0.0407	AT2G44930
0.00	-2.96	-3.13	-3.04	0.0178	AT2G01300
0.00	-2.95	-3.15	-3.05	0.0209	AT2G20870
0.00	-2.86	-3.29	-3.08	0.0444	AT3G21945
0.00	-2.88	-3.35	-3.12	0.0479	AT2G23800
0.00	-3.08	-3.16	-3.12	0.0082	AT5G33370
0.00	-3.28	-2.97	-3.12	0.0316	AT3G12710
0.00	-3.29	-2.96	-3.12	0.0336	AT3G01840
0.00	-3.01	-3.24	-3.13	0.0234	AT4G02330
0.00	-3.15	-3.12	-3.13	0.0030	AT1G71450
0.00	-2.93	-3.36	-3.14	0.0435	AT1G12570
0.00	-3.23	-3.09	-3.16	0.0141	AT5G02200
0.00	-3.13	-3.27	-3.20	0.0139	AT1G12845
0.00	-3.01	-3.39	-3.20	0.0378	AT4G15660
0.00	-3.27	-3.15	-3.21	0.0119	AT2G31980
0.00	-3.26	-3.15	-3.21	0.0109	AT2G32885
0.00	-3.13	-3.32	-3.22	0.0187	AT5G04370
0.00	-3.21	-3.25	-3.23	0.0039	AT3G59310
0.00	-3.21	-3.26	-3.24	0.0049	AT3G43083
0.00	-3.25	-3.36	-3.31	0.0106	AT5G63090
0.00	-3.13	-3.49	-3.31	0.0346	AT1G66100
0.00	-3.26	-3.36	-3.31	0.0096	AT4G15680
0.00	-3.06	-3.56	-3.31	0.0480	AT2G42990
0.00	-3.12	-3.51	-3.31	0.0374	AT5G45340
0.00	-3.06	-3.58	-3.32	0.0498	AT1G66760
0.00	-3.39	-3.25	-3.32	0.0134	AT1G01190

0.00	-3.27	-3.37	-3.32	0.0096	AT3G10600
0.00	-3.36	-3.29	-3.33	0.0067	AT5G22380
0.00	-3.30	-3.37	-3.34	0.0067	AT5G42290
0.00	-3.20	-3.56	-3.38	0.0339	AT4G08040
0.00	-3.55	-3.21	-3.38	0.0320	AT3G17520
0.00	-3.42	-3.35	-3.38	0.0066	AT1G15360
0.00	-3.31	-3.46	-3.39	0.0141	AT3G46880
0.00	-3.48	-3.32	-3.40	0.0150	AT5G38000
0.00	-3.17	-3.63	-3.40	0.0430	AT1G17700
0.00	-3.35	-3.53	-3.44	0.0167	AT3G54000
0.00	-3.43	-3.47	-3.45	0.0037	AT1G63030
0.00	-3.56	-3.35	-3.45	0.0193	AT1G06350
0.00	-3.60	-3.36	-3.48	0.0219	AT1G23600
0.00	-3.54	-3.49	-3.52	0.0045	AT2G10940
0.00	-3.57	-3.50	-3.53	0.0063	AT5G55450
0.00	-3.39	-3.70	-3.54	0.0278	AT5G15230
0.00	-3.51	-3.70	-3.60	0.0168	AT5G20630
0.00	-3.60	-3.63	-3.62	0.0026	AT5G12940
0.00	-3.63	-3.75	-3.69	0.0104	AT2G29130
0.00	-3.45	-3.95	-3.70	0.0429	AT1G33760
0.00	-3.75	-3.85	-3.80	0.0084	AT4G25470
0.00	-3.79	-3.87	-3.83	0.0066	AT3G11825
0.00	-4.13	-3.64	-3.88	0.0401	AT5G18430
0.00	-3.88	-3.98	-3.93	0.0081	AT1G80660
0.00	-3.81	-4.27	-4.04	0.0362	AT1G02810
0.00	-3.86	-4.22	-4.04	0.0283	AT5G22430
0.00	-4.15	-3.95	-4.05	0.0157	AT5G61605
0.00	-4.14	-4.06	-4.10	0.0062	AT2G28405
0.00	-4.07	-4.18	-4.12	0.0085	AT5G55720
0.00	-4.12	-4.16	-4.14	0.0031	AT3G19200
0.00	-3.92	-4.36	-4.14	0.0338	AT5G07560
0.00	-4.17	-4.12	-4.14	0.0038	AT1G14480
0.00	-4.07	-4.28	-4.18	0.0160	AT2G28190
0.00	-4.50	-4.26	-4.38	0.0174	AT5G51190
0.00	-4.30	-4.60	-4.45	0.0215	AT2G28355
0.00	-4.34	-4.57	-4.45	0.0164	AT3G09870
0.00	-4.53	-4.56	-4.54	0.0021	AT5G10100
0.00	-4.66	-4.62	-4.64	0.0027	AT2G24610
0.00	-4.43	-4.93	-4.68	0.0340	AT3G54070
0.00	-4.62	-4.86	-4.74	0.0161	AT5G37940
0.00	-5.17	-5.03	-5.10	0.0087	AT5G07530
0.00	-4.91	-5.67	-5.29	0.0457	AT1G66850
0.00	-5.09	-5.54	-5.32	0.0269	AT4G25490
0.00	-6.05	-5.97	-6.01	0.0042	AT5G07550

Supplementary material 5: List of up- and down-regulated genes (HE-6D-WL vs WT-6D-WL) with their normalized fold induction and adjusted p value

Normalized Fold WT-6D-WL	Fold OE-6D-WL 1	Fold OE-6D-WL 2	Normalized Fold OE-6D-WL	Adjusted P value	Gene ID
0.00	6.93	7.00	6.97	0.0032	AT3G12000
0.00	6.83	6.41	6.62	0.0202	AT4G11180
0.00	6.99	6.08	6.54	0.0443	AT1G53130
0.00	6.41	6.63	6.52	0.0107	AT3G04290
0.00	6.39	6.33	6.36	0.0030	AT5G38910
0.00	6.45	5.67	6.06	0.0409	AT1G11600
0.00	6.07	6.02	6.04	0.0026	AT4G11320
0.00	6.17	5.65	5.91	0.0280	AT1G72290
0.00	5.94	5.47	5.71	0.0262	AT3G11430
0.00	5.15	5.58	5.37	0.0255	AT4G23680
0.00	5.61	4.98	5.30	0.0378	AT1G26945
0.00	5.59	4.81	5.20	0.0477	AT4G30180
0.00	5.12	5.22	5.17	0.0062	AT2G14690
0.00	4.90	5.43	5.16	0.0326	AT5G47600
0.00	4.90	5.21	5.06	0.0195	AT5G45950
0.00	5.37	4.68	5.03	0.0436	AT4G19430
0.00	5.07	4.70	4.89	0.0241	AT1G17180
0.00	5.21	4.49	4.85	0.0472	AT5G22430
0.00	5.08	4.39	4.74	0.0463	AT2G18620
0.00	4.62	4.82	4.72	0.0135	AT1G11850
0.00	4.64	4.59	4.62	0.0034	AT2G24210
0.00	4.47	4.28	4.37	0.0138	AT1G24130
0.00	4.29	4.32	4.30	0.0022	AT5G08000
0.00	3.91	4.50	4.21	0.0446	AT1G22760
0.00	4.27	4.01	4.14	0.0200	AT5G20630
0.00	3.89	4.21	4.05	0.0251	AT5G44300
0.00	4.16	3.81	3.98	0.0279	AT1G66120
0.00	3.82	4.13	3.98	0.0248	AT4G10260
0.00	3.70	4.21	3.96	0.0410	AT1G31310
0.00	3.90	3.96	3.93	0.0049	AT1G28170
0.00	3.61	4.17	3.89	0.0457	AT3G28220
0.00	3.97	3.75	3.86	0.0181	AT5G59040
0.00	4.13	3.55	3.84	0.0480	AT3G59610
0.00	3.75	3.91	3.83	0.0133	AT5G24780
0.00	4.00	3.59	3.80	0.0344	AT4G32714
0.00	3.80	3.64	3.72	0.0137	AT1G23200
0.00	3.49	3.83	3.66	0.0295	AT1G52690
0.00	3.69	3.61	3.65	0.0070	AT5G53680
0.00	3.64	3.65	3.65	0.0009	AT4G36610
0.00	3.39	3.66	3.53	0.0244	AT2G10940

0.00	3.46	3.56	3.51	0.0091	AT3G27810
0.00	3.67	3.27	3.47	0.0367	AT5G25330
0.00	3.22	3.65	3.43	0.0398	AT5G12460
0.00	3.16	3.69	3.43	0.0492	AT3G08900
0.00	3.52	3.33	3.42	0.0177	AT3G03240
0.00	3.67	3.14	3.41	0.0494	AT5G53390
0.00	3.63	3.16	3.40	0.0440	AT4G29305
0.00	3.40	3.35	3.38	0.0047	AT1G19320
0.00	3.09	3.53	3.31	0.0423	AT2G38530
0.00	3.03	3.54	3.29	0.0493	AT2G23400
0.00	3.10	3.47	3.28	0.0358	AT1G25340
0.00	3.20	3.34	3.27	0.0136	AT5G65730
0.00	3.22	3.27	3.25	0.0049	AT4G13790
0.00	3.42	3.01	3.22	0.0405	AT5G40420
0.00	3.17	3.25	3.21	0.0079	AT3G47350
0.00	3.03	3.38	3.21	0.0347	AT3G17760
0.00	3.26	3.09	3.17	0.0170	AT5G25460
0.00	3.18	3.16	3.17	0.0020	AT1G70970
0.00	3.35	2.99	3.17	0.0361	AT5G50335
0.00	3.30	3.03	3.17	0.0271	AT5G37810
0.00	2.93	3.38	3.16	0.0453	AT2G16580
0.00	3.35	2.92	3.14	0.0436	AT4G18320
0.00	3.21	3.00	3.10	0.0215	AT2G23060
0.00	2.96	3.20	3.08	0.0248	AT1G28700
0.00	3.04	3.11	3.07	0.0072	AT5G03620
0.00	2.91	3.21	3.06	0.0312	AT1G31530
0.00	2.99	3.12	3.05	0.0135	AT4G38860
0.00	2.97	3.14	3.05	0.0177	AT5G61850
0.00	2.88	3.19	3.04	0.0325	AT1G19640
0.00	3.11	2.95	3.03	0.0168	AT1G80080
0.00	2.90	3.13	3.01	0.0243	AT4G15620
0.00	3.08	2.93	3.01	0.0159	AT2G34315
0.00	2.96	2.99	2.98	0.0032	AT4G20670
0.00	3.01	2.93	2.97	0.0086	AT3G47340
0.00	2.83	3.08	2.96	0.0269	AT2G46870
0.00	2.87	2.99	2.93	0.0130	AT5G63225
0.00	3.06	2.74	2.90	0.0351	AT1G07340
0.00	3.06	2.70	2.88	0.0397	AT4G39760
0.00	3.05	2.71	2.88	0.0375	AT5G17340
0.00	2.89	2.84	2.86	0.0056	AT1G26796
0.00	2.94	2.78	2.86	0.0178	AT3G61840
0.00	2.90	2.80	2.85	0.0112	AT4G27330
0.00	2.64	3.06	2.85	0.0468	AT2G32530
0.00	2.78	2.90	2.84	0.0134	AT5G20240
0.00	2.95	2.66	2.81	0.0329	AT2G40610
0.00	2.83	2.78	2.80	0.0057	AT3G02640

0.00	2.98	2.61	2.79	0.0421	AT2G14620
0.00	2.83	2.75	2.79	0.0091	AT1G02810
0.00	2.89	2.65	2.77	0.0276	AT1G15160
0.00	2.57	2.97	2.77	0.0459	AT3G49580
0.00	2.81	2.69	2.75	0.0139	AT5G38080
0.00	2.66	2.84	2.75	0.0208	AT1G61090
0.00	2.86	2.64	2.75	0.0255	AT3G23730
0.00	2.71	2.78	2.74	0.0081	AT4G28365
0.00	2.78	2.71	2.74	0.0081	AT1G49810
0.00	2.66	2.82	2.74	0.0186	AT3G44250
0.00	2.91	2.56	2.73	0.0407	AT5G11130
0.00	2.67	2.79	2.73	0.0140	AT1G27461
0.00	2.93	2.52	2.73	0.0478	AT1G77830
0.00	2.71	2.72	2.71	0.0012	AT1G27220
0.00	2.61	2.80	2.70	0.0223	AT1G66850
0.00	2.56	2.80	2.68	0.0285	AT1G30950
0.00	2.68	2.67	2.68	0.0012	AT1G02065
0.00	2.55	2.76	2.66	0.0252	AT1G75880
0.00	2.48	2.82	2.65	0.0408	AT1G26390
0.00	2.72	2.55	2.63	0.0205	AT3G18360
0.00	2.66	2.60	2.63	0.0073	AT5G20820
0.00	2.47	2.78	2.63	0.0375	AT3G06220
0.00	2.55	2.68	2.61	0.0158	AT4G10490
0.00	2.40	2.81	2.61	0.0500	AT1G13420
0.00	2.58	2.61	2.60	0.0037	AT4G23380
0.00	2.46	2.74	2.60	0.0342	AT5G51210
0.00	2.48	2.70	2.59	0.0270	AT5G27200
0.00	2.42	2.76	2.59	0.0417	AT4G22810
0.00	2.58	2.58	2.58	0.0000	AT3G21830
0.00	2.74	2.41	2.57	0.0407	AT1G60470
0.00	2.71	2.41	2.56	0.0373	AT5G07500
0.00	2.61	2.49	2.55	0.0150	AT1G69530
0.00	2.61	2.49	2.55	0.0150	AT1G12805
0.00	2.71	2.39	2.55	0.0399	AT1G29490
0.00	2.42	2.67	2.55	0.0312	AT4G30230
0.00	2.46	2.63	2.55	0.0213	AT3G21840
0.00	2.40	2.66	2.53	0.0327	AT4G39590
0.00	2.58	2.47	2.52	0.0139	AT5G48100
0.00	2.56	2.46	2.51	0.0127	AT2G02540
0.00	2.60	2.42	2.51	0.0228	AT2G16060
0.00	2.60	2.41	2.50	0.0241	AT5G18020
0.00	2.59	2.42	2.50	0.0216	AT4G02330
0.00	2.52	2.48	2.50	0.0051	AT3G45248
0.00	2.46	2.53	2.49	0.0089	AT1G04250
0.00	2.38	2.60	2.49	0.0281	AT1G52695
0.00	2.47	2.50	2.49	0.0038	AT1G53285

0.00	2.33	2.64	2.48	0.0397	AT4G12825
0.00	2.52	2.44	2.48	0.0103	AT2G41280
0.00	2.60	2.34	2.47	0.0335	AT1G70560
0.00	2.43	2.50	2.46	0.0090	AT3G50010
0.00	2.46	2.46	2.46	0.0000	AT1G68570
0.00	2.28	2.62	2.45	0.0441	AT1G05835
0.00	2.53	2.37	2.45	0.0208	AT1G26795
0.00	2.35	2.52	2.43	0.0222	AT2G10535
0.00	2.47	2.39	2.43	0.0105	AT3G23130
0.00	2.49	2.33	2.41	0.0211	AT5G36770
0.00	2.29	2.52	2.40	0.0304	AT5G51490
0.00	2.47	2.34	2.40	0.0172	AT5G06839
0.00	2.27	2.54	2.40	0.0357	AT4G21690
0.00	2.32	2.49	2.40	0.0225	AT5G08250
0.00	2.27	2.52	2.39	0.0332	AT4G15090
0.00	2.48	2.31	2.39	0.0226	AT2G45830
0.00	2.35	2.43	2.39	0.0107	AT5G59720
0.00	2.41	2.37	2.39	0.0053	AT4G31910
0.00	2.53	2.24	2.38	0.0387	AT4G33560
0.00	2.38	2.38	2.38	0.0000	AT1G02575
0.00	2.23	2.54	2.38	0.0413	AT3G24690
0.00	2.38	2.37	2.38	0.0013	AT2G43050
0.00	2.45	2.29	2.37	0.0215	AT3G12145
0.00	2.52	2.22	2.37	0.0402	AT3G12470
0.00	2.49	2.24	2.37	0.0336	AT1G21910
0.00	2.42	2.31	2.36	0.0148	AT3G60780
0.00	2.22	2.50	2.36	0.0377	AT4G10250
0.00	2.38	2.33	2.36	0.0068	AT5G49100
0.00	2.41	2.30	2.36	0.0149	AT4G12500
0.00	2.48	2.23	2.36	0.0338	AT1G23570
0.00	2.47	2.20	2.34	0.0368	AT5G17700
0.00	2.35	2.32	2.34	0.0041	AT1G78160
0.00	2.48	2.17	2.33	0.0424	AT2G45610
0.00	2.32	2.34	2.33	0.0027	AT4G12490
0.00	2.34	2.29	2.32	0.0069	AT5G18050
0.00	2.13	2.48	2.31	0.0482	AT4G35160
0.00	2.31	2.26	2.28	0.0070	AT5G23020
0.00	2.34	2.22	2.28	0.0167	AT4G32490
0.00	2.29	2.25	2.27	0.0056	AT5G18080
0.00	2.10	2.43	2.26	0.0463	AT3G12870
0.00	2.23	2.29	2.26	0.0085	AT1G61095
0.00	2.18	2.32	2.25	0.0198	AT5G10140
0.00	2.26	2.24	2.25	0.0028	AT4G13700
0.00	2.19	2.31	2.25	0.0170	AT1G61093
0.00	2.26	2.24	2.25	0.0028	AT4G30880
0.00	2.10	2.39	2.24	0.0411	AT1G66430

0.00	2.21	2.28	2.24	0.0099	AT5G01075
0.00	2.32	2.16	2.24	0.0227	AT3G17070
0.00	2.34	2.14	2.24	0.0284	AT3G42725
0.00	2.14	2.33	2.24	0.0270	AT5G07160
0.00	2.29	2.18	2.23	0.0157	AT5G16250
0.00	2.17	2.30	2.23	0.0185	AT2G17470
0.00	2.25	2.21	2.23	0.0057	AT4G08950
0.00	2.15	2.28	2.22	0.0187	AT4G24275
0.00	2.21	2.21	2.21	0.0000	AT4G01140
0.00	2.14	2.28	2.21	0.0202	AT2G22805
0.00	2.26	2.14	2.20	0.0174	AT4G32100
0.00	2.31	2.09	2.20	0.0318	AT5G07200
0.00	2.05	2.34	2.19	0.0420	AT5G38960
0.00	2.20	2.19	2.19	0.0015	AT2G17723
0.00	2.08	2.31	2.19	0.0333	AT3G05980
0.00	2.13	2.23	2.18	0.0146	AT5G50770
0.00	2.09	2.27	2.18	0.0263	AT1G63060
0.00	2.18	2.17	2.17	0.0015	AT1G70440
0.00	2.25	2.10	2.17	0.0219	AT4G38660
0.00	2.14	2.20	2.17	0.0088	AT4G01500
0.00	2.31	2.04	2.17	0.0395	AT1G54560
0.00	2.10	2.24	2.17	0.0205	AT2G43386
0.00	2.29	2.03	2.16	0.0383	AT1G15150
0.00	2.06	2.25	2.16	0.0280	AT1G75640
0.00	2.28	2.02	2.15	0.0384	AT1G26250
0.00	2.27	2.03	2.15	0.0355	AT1G26799
0.00	2.08	2.22	2.15	0.0207	AT4G34760
0.00	2.29	2.00	2.14	0.0430	AT3G54400
0.00	2.23	2.05	2.14	0.0268	AT5G09970
0.00	2.02	2.26	2.14	0.0357	AT2G16300
0.00	2.13	2.15	2.14	0.0030	AT5G13170
0.00	2.16	2.11	2.13	0.0075	AT3G03820
0.00	2.04	2.22	2.13	0.0269	AT3G55660
0.00	2.14	2.12	2.13	0.0030	AT5G12940
0.00	2.00	2.24	2.12	0.0360	AT1G22110
0.00	2.11	2.13	2.12	0.0030	AT1G29450
0.00	2.19	2.02	2.11	0.0257	AT3G17130
0.00	2.10	2.11	2.11	0.0015	AT3G14210
0.00	2.24	1.95	2.10	0.0440	AT4G38850
0.00	2.05	2.14	2.09	0.0137	AT1G43666
0.00	1.98	2.20	2.09	0.0335	AT3G06840
0.00	2.11	2.06	2.09	0.0076	AT4G35170
0.00	2.07	2.09	2.08	0.0031	AT5G39560
0.00	1.97	2.19	2.08	0.0336	AT3G21910
0.00	2.11	2.04	2.07	0.0107	AT5G57920
0.00	1.99	2.16	2.07	0.0261	AT2G42900

0.00	1.96	2.16	2.06	0.0309	AT1G65450
0.00	2.19	1.93	2.06	0.0401	AT3G25750
0.00	1.95	2.16	2.06	0.0325	AT1G48930
0.00	2.18	1.93	2.06	0.0387	AT5G07030
0.00	2.11	2.00	2.06	0.0170	AT1G65610
0.00	1.97	2.14	2.05	0.0263	AT4G38000
0.00	2.02	2.06	2.04	0.0062	AT1G78950
0.00	1.99	2.09	2.04	0.0156	AT1G07476
0.00	2.03	2.05	2.04	0.0031	AT1G44414
0.00	2.01	2.06	2.04	0.0078	AT2G43610
0.00	1.96	2.11	2.04	0.0235	AT2G36790
0.00	1.89	2.18	2.03	0.0453	AT2G32610
0.00	1.97	2.09	2.03	0.0188	AT4G27810
0.00	1.89	2.17	2.03	0.0438	AT3G23167
0.00	1.89	2.16	2.02	0.0424	AT5G05810
0.00	2.13	1.92	2.02	0.0330	AT1G62340
0.00	2.12	1.92	2.02	0.0315	AT4G33660
0.00	2.01	2.03	2.02	0.0032	AT4G28950
0.00	1.96	2.08	2.02	0.0189	AT1G17170
0.00	2.03	2.00	2.02	0.0047	AT5G65370
0.00	1.95	2.08	2.01	0.0205	AT1G53520
0.00	1.86	2.17	2.01	0.0489	AT5G37473
0.00	1.94	2.08	2.01	0.0222	AT2G03770
0.00	2.10	1.91	2.00	0.0301	AT3G26140
0.00	1.99	2.01	2.00	0.0032	AT2G44240
0.00	2.12	1.88	2.00	0.0382	AT2G34700
0.00	1.91	2.08	2.00	0.0271	AT2G27385
0.00	-2.01	-2.00	-2.00	0.0357	AT5G37478
0.00	-2.12	-1.92	-2.02	0.0251	AT1G29720
0.00	-1.89	-2.16	-2.02	0.0029	AT1G30860
0.00	-2.05	-2.00	-2.02	0.0031	AT4G34600
0.00	-2.22	-1.85	-2.03	0.0177	AT1G69970
0.00	-2.05	-2.03	-2.04	0.0392	AT5G61040
0.00	-1.96	-2.12	-2.04	0.0160	AT2G15320
0.00	-2.05	-2.05	-2.05	0.0270	AT1G48970
0.00	-1.94	-2.19	-2.06	0.0236	AT1G17455
0.00	-2.34	-1.79	-2.07	0.0162	AT4G21930
0.00	-2.03	-2.11	-2.07	0.0371	AT2G07728
0.00	-2.26	-1.92	-2.09	0.0062	AT1G13609
0.00	-1.92	-2.30	-2.11	0.0118	AT5G54225
0.00	-1.13	-3.11	-2.12	0.0441	AT4G04740
0.00	-1.93	-2.33	-2.13	0.0106	AT3G01680
0.00	-1.77	-2.55	-2.16	0.0185	AT1G17940
0.00	-2.13	-2.23	-2.18	0.0317	AT5G44660
0.00	-2.37	-2.00	-2.18	0.0023	AT5G62940
0.00	-2.90	-1.53	-2.21	0.0311	AT4G08874

0.00	-2.00	-2.49	-2.24	0.0211	AT3G04450
0.00	-2.14	-2.35	-2.25	0.0099	AT1G18730
0.00	-2.38	-2.12	-2.25	0.0148	AT4G32785
0.00	-2.03	-2.48	-2.25	0.0358	AT1G73390
0.00	-2.26	-2.38	-2.32	0.0433	AT5G06610
0.00	-2.88	-1.81	-2.35	0.0185	AT2G32290
0.00	-2.39	-2.55	-2.47	0.0029	AT2G45900
0.00	-2.14	-2.81	-2.48	0.0081	AT5G48110
0.00	-2.29	-2.70	-2.49	0.0185	AT5G64905
0.00	-2.75	-2.25	-2.50	0.0090	AT1G27620
0.00	-2.63	-2.47	-2.55	0.0205	AT5G64910
0.00	-3.02	-2.15	-2.58	0.0163	AT5G24990
0.00	-2.40	-2.78	-2.59	0.0442	AT2G22980
0.00	-2.63	-2.57	-2.60	0.0000	AT4G39650
0.00	-2.84	-2.44	-2.64	0.0374	AT4G37150
0.00	-2.72	-2.58	-2.65	0.0147	AT3G27027
0.00	-2.46	-2.95	-2.70	0.0409	AT3G01860
0.00	-2.52	-2.90	-2.71	0.0381	AT1G54445
0.00	-1.53	-3.92	-2.73	0.0448	AT5G10970
0.00	-2.93	-2.56	-2.75	0.0000	AT1G69080
0.00	-2.92	-2.57	-2.75	0.0159	AT1G10920
0.00	-2.81	-2.69	-2.75	0.0261	AT1G49820
0.00	-3.39	-2.20	-2.79	0.0258	AT2G33480
0.00	-2.52	-3.10	-2.81	0.0271	AT1G56240
0.00	-1.84	-3.87	-2.86	0.0062	AT2G23148
0.00	-2.69	-3.06	-2.87	0.0434	AT3G03650
0.00	-3.00	-3.05	-3.03	0.0172	AT5G67370
0.00	-2.67	-3.43	-3.05	0.0366	AT1G18485
0.00	-2.01	-4.15	-3.08	0.0177	AT1G48260
0.00	-3.39	-2.90	-3.14	0.0189	AT4G30830
0.00	-3.58	-2.78	-3.18	0.0428	AT1G69550
0.00	-2.42	-4.02	-3.22	0.0406	AT2G36145
0.00	-3.12	-3.33	-3.22	0.0123	AT2G41850
0.00	-3.66	-2.85	-3.25	0.0146	AT1G73260
0.00	-3.02	-3.50	-3.26	0.0441	AT2G07787
0.00	-3.30	-3.24	-3.27	0.0416	AT2G24540
0.00	-2.62	-3.92	-3.27	0.0297	AT1G30700
0.00	-3.23	-3.31	-3.27	0.0152	AT3G01670
0.00	-3.07	-3.56	-3.31	0.0454	AT5G46115
0.00	-3.64	-3.03	-3.34	0.0030	AT3G24520
0.00	-3.37	-3.31	-3.34	0.0415	AT2G47950
0.00	-3.86	-2.84	-3.35	0.0205	AT5G14360
0.00	-3.28	-3.56	-3.42	0.0111	AT1G69490
0.00	-3.44	-3.45	-3.45	0.0460	AT3G27425
0.00	-3.42	-3.51	-3.46	0.0452	AT1G49720
0.00	-3.23	-3.75	-3.49	0.0404	AT3G19370

0.00	-3.69	-3.38	-3.53	0.0207	AT5G04150
0.00	-3.24	-4.02	-3.63	0.0470	AT5G04200
0.00	-3.03	-4.37	-3.70	0.0122	AT4G15765
0.00	-4.78	-2.68	-3.73	0.0473	AT3G14570
0.00	-3.76	-3.72	-3.74	0.0450	AT1G19220
0.00	-3.55	-4.23	-3.89	0.0306	AT2G02515
0.00	-4.28	-3.71	-3.99	0.0340	AT2G39470
0.00	-4.18	-3.91	-4.05	0.0039	AT4G34560
0.00	-3.65	-4.49	-4.07	0.0395	AT4G32110
0.00	-4.67	-3.53	-4.10	0.0019	AT5G39260
0.00	-4.15	-4.19	-4.17	0.0256	AT5G45890
0.00	-4.45	-3.90	-4.18	0.0454	AT2G32860
0.00	-4.14	-4.56	-4.35	0.0303	AT3G48420
0.00	-4.30	-4.47	-4.38	0.0319	AT1G70760
0.00	-4.65	-4.14	-4.39	0.0248	AT1G15980
0.00	-3.80	-5.19	-4.49	0.0219	AT3G06500
0.00	-4.25	-4.81	-4.53	0.0155	AT1G49620
0.00	-5.23	-4.09	-4.66	0.0131	AT4G37390
0.00	-4.41	-5.57	-4.99	0.0389	AT3G12890
0.00	-5.02	-5.00	-5.01	0.0242	AT3G26130
0.00	-5.03	-5.00	-5.02	0.0141	AT2G17940
0.00	-4.97	-5.20	-5.08	0.0484	AT3G18290
0.00	-5.57	-4.91	-5.24	0.0484	AT3G56810
0.00	-6.36	-5.61	-5.99	0.0335	AT2G34000
0.00	-5.89	-6.76	-6.32	0.0469	AT5G23810
0.00	-7.03	-7.27	-7.15	0.0310	AT2G39850

Supplementary material 6

Up-regulated Functional category

Term	Number of genes	P- Value
GO:0050896~response to stimulus	532	0.0000
GO:0006950~response to stress	326	0.0000
GO:0051707~response to other organism	145	0.0000
GO:0005576~extracellular region	188	0.0000
GO:0009628~response to abiotic stimulus	191	0.0000
GO:0009607~response to biotic stimulus	151	0.0000
GO:0032502~developmental process	256	0.0000
GO:0042221~response to chemical stimulus	275	0.0000
GO:0007275~multicellular organismal development	238	0.0000
GO:0032501~multicellular organismal process	247	0.0000
GO:0048513~organ development	120	0.0000
GO:0048731~system development	120	0.0000
GO:0051704~multi-organism process	160	0.0000
GO:0010033~response to organic substance	183	0.0000
GO:0009719~response to endogenous stimulus	158	0.0000
GO:0048856~anatomical structure development	198	0.0000
GO:0001906~cell killing	68	0.0000
GO:0031640~killing of cells of another organism	68	0.0000
GO:0009620~response to fungus	86	0.0000
GO:0032787~monocarboxylic acid metabolic process	74	0.0000
GO:0050832~defense response to fungus	78	0.0000
GO:0009698~phenylpropanoid metabolic process	44	0.0000
GO:0009725~response to hormone stimulus	139	0.0000
GO:0006631~fatty acid metabolic process	50	0.0000
GO:0004497~monooxygenase activity	61	0.0000
GO:0009813~flavonoid biosynthetic process	22	0.0000
GO:0042398~cellular amino acid derivative biosynthetic process	46	0.0000
GO:0005506~iron ion binding	107	0.0000
GO:0019825~oxygen binding	48	0.0000
GO:0009699~phenylpropanoid biosynthetic process	35	0.0000
GO:0009812~flavonoid metabolic process	22	0.0000
GO:0003677~DNA binding	276	0.0000
GO:0006575~cellular amino acid derivative metabolic process	57	0.0000
GO:0009416~response to light stimulus	81	0.0000
GO:0035251~UDP-glucosyltransferase activity	31	0.0000
GO:0009266~response to temperature stimulus	66	0.0000
GO:0009314~response to radiation	82	0.0000
GO:0009414~response to water deprivation	41	0.0000
GO:0046527~glucosyltransferase activity	33	0.0000
GO:0008194~UDP-glycosyltransferase activity	46	0.0000
GO:0003700~transcription factor activity	200	0.0000
GO:0009415~response to water	42	0.0000
GO:0006633~fatty acid biosynthetic process	38	0.0000
GO:0006952~defense response	145	0.0000
GO:0016758~transferase activity, transferring hexosyl groups	65	0.0000
GO:0020037~heme binding	62	0.0000
GO:0006725~cellular aromatic compound metabolic process	63	0.0000
GO:0046906~tetrapyrrole binding	65	0.0000
GO:0009408~response to heat	32	0.0000
GO:0042546~cell wall biogenesis	23	0.0000
GO:0019748~secondary metabolic process	72	0.0000
GO:0046658~anchored to plasma membrane	21	0.0000
GO:0009739~response to gibberellin stimulus	30	0.0000

GO:0016053~organic acid biosynthetic process	69	**0.0000**
GO:0046394~carboxylic acid biosynthetic process	69	**0.0000**
GO:0022621~shoot system development	53	**0.0000**
GO:0016757~transferase activity, transferring glycosyl groups	74	**0.0000**
GO:0046914~transition metal ion binding	294	**0.0000**
GO:0009753~response to jasmonic acid stimulus	35	**0.0000**
GO:0010383~cell wall polysaccharide metabolic process	10	**0.0000**
GO:0016491~oxidoreductase activity	176	**0.0000**
GO:0048367~shoot development	52	**0.0000**
GO:0005634~nucleus	363	**0.0000**
GO:0003006~reproductive developmental process	114	**0.0000**
GO:0009908~flower development	45	**0.0000**
GO:0030528~transcription regulator activity	214	**0.0000**
GO:0080044~quercetin 7-O-glucosyltransferase activity	10	**0.0000**
GO:0008610~lipid biosynthetic process	68	**0.0000**
GO:0048608~reproductive structure development	105	**0.0000**
GO:0006629~lipid metabolic process	110	**0.0000**
GO:0042180~cellular ketone metabolic process	110	**0.0000**
GO:0019438~aromatic compound biosynthetic process	42	**0.0000**
GO:0043436~oxoacid metabolic process	108	**0.0000**
GO:0019752~carboxylic acid metabolic process	108	**0.0000**
GO:0006082~organic acid metabolic process	108	**0.0000**
GO:0009791~post-embryonic development	117	**0.0000**
GO:0080043~quercetin 3-O-glucosyltransferase activity	11	**0.0000**
GO:0043167~ion binding	362	**0.0000**
GO:0043169~cation binding	361	**0.0000**
GO:0048869~cellular developmental process	69	**0.0000**
GO:0045491~xylan metabolic process	8	**0.0000**
GO:0010410~hemicellulose metabolic process	8	**0.0000**
GO:0010417~glucuronoxylan biosynthetic process	7	**0.0000**
GO:0045492~xylan biosynthetic process	7	**0.0000**
GO:0070592~cell wall polysaccharide biosynthetic process	7	**0.0000**
GO:0010413~glucuronoxylan metabolic process	7	**0.0000**
GO:0065007~biological regulation	444	**0.0000**
GO:0044255~cellular lipid metabolic process	73	**0.0000**
GO:0022414~reproductive process	119	**0.0000**
GO:0042445~hormone metabolic process	20	**0.0000**
GO:0031226~intrinsic to plasma membrane	24	**0.0000**
GO:0010817~regulation of hormone levels	27	**0.0000**
GO:0006350~transcription	163	**0.0000**
GO:0000003~reproduction	120	**0.0000**
GO:0009888~tissue development	43	**0.0000**
GO:0009653~anatomical structure morphogenesis	70	**0.0000**
GO:0065008~regulation of biological quality	90	**0.0000**
GO:0016051~carbohydrate biosynthetic process	44	**0.0001**
GO:0009832~plant-type cell wall biogenesis	16	**0.0001**
GO:0010382~cellular cell wall macromolecule metabolic process	8	**0.0001**
GO:0048046~apoplast	57	**0.0001**
GO:0030054~cell junction	10	**0.0001**
GO:0008202~steroid metabolic process	18	**0.0001**
GO:0046872~metal ion binding	336	**0.0001**
GO:0055044~symplast	8	**0.0001**
GO:0009506~plasmodesma	8	**0.0001**
GO:0009723~response to ethylene stimulus	43	**0.0001**
GO:0006970~response to osmotic stress	60	**0.0001**
GO:0031323~regulation of cellular metabolic process	247	**0.0002**
GO:0070589~cellular component macromolecule biosynthetic process	7	**0.0002**
GO:0044038~cell wall macromolecule biosynthetic process	7	**0.0002**

GO:0048827~phyllome development	36	**0.0002**
GO:0030154~cell differentiation	49	**0.0002**
GO:0003824~catalytic activity	770	**0.0002**
GO:0009889~regulation of biosynthetic process	237	**0.0002**
GO:0031326~regulation of cellular biosynthetic process	237	**0.0002**
GO:0008544~epidermis development	24	**0.0003**
GO:0007398~ectoderm development	24	**0.0003**
GO:0048523~negative regulation of cellular process	34	**0.0003**
GO:0000786~nucleosome	16	**0.0003**
GO:0032993~protein-DNA complex	17	**0.0003**
GO:0010286~heat acclimation	8	**0.0003**
GO:0010025~wax biosynthetic process	8	**0.0003**
GO:0019219~regulation of nucleobase, nucleoside, nucleotide and nucleic acid metabolic process	231	**0.0003**
GO:0009873~ethylene mediated signaling pathway	31	**0.0003**
GO:0051171~regulation of nitrogen compound metabolic process	232	**0.0003**
GO:0009605~response to external stimulus	53	**0.0003**
GO:0006519~cellular amino acid and derivative metabolic process	82	**0.0004**
GO:0051716~cellular response to stimulus	121	**0.0004**
GO:0009055~electron carrier activity	80	**0.0004**
GO:0048825~cotyledon development	10	**0.0004**
GO:0010556~regulation of macromolecule biosynthetic process	232	**0.0004**
GO:0010166~wax metabolic process	8	**0.0004**
GO:0045449~regulation of transcription	227	**0.0005**
GO:0031225~anchored to membrane	47	**0.0005**
GO:0009913~epidermal cell differentiation	23	**0.0005**
GO:0005911~cell-cell junction	8	**0.0005**
GO:0051219~phosphoprotein binding	7	**0.0005**
GO:0045309~protein phosphorylated amino acid binding	7	**0.0005**
GO:0019439~aromatic compound catabolic process	10	**0.0006**
GO:0009808~lignin metabolic process	16	**0.0006**
GO:0006333~chromatin assembly or disassembly	20	**0.0006**
GO:0047262~polygalacturonate 4-alpha-galacturonosyltransferase activity	9	**0.0007**
GO:0016628~oxidoreductase activity, acting on the CH-CH group of donors, NAD or NADP as acceptor	9	**0.0007**
GO:0031407~oxylipin metabolic process	11	**0.0007**
GO:0009834~secondary cell wall biogenesis	8	**0.0007**
GO:0051213~dioxygenase activity	15	**0.0007**
GO:0000785~chromatin	19	**0.0007**
GO:0006355~regulation of transcription, DNA-dependent	131	**0.0008**
GO:0055114~oxidation reduction	141	**0.0008**
GO:0009611~response to wounding	27	**0.0008**
GO:0033692~cellular polysaccharide biosynthetic process	21	**0.0009**
GO:0051252~regulation of RNA metabolic process	131	**0.0009**
GO:0034637~cellular carbohydrate biosynthetic process	32	**0.0010**
GO:0080090~regulation of primary metabolic process	240	**0.0010**
GO:0009651~response to salt stress	53	**0.0010**
GO:0009718~anthocyanin biosynthetic process	6	**0.0011**
GO:0032870~cellular response to hormone stimulus	60	**0.0011**
GO:0009755~hormone-mediated signaling	60	**0.0011**
GO:0006334~nucleosome assembly	16	**0.0012**
GO:0034728~nucleosome organization	16	**0.0012**
GO:0009409~response to cold	37	**0.0012**
GO:0050794~regulation of cellular process	360	**0.0012**
GO:0016679~oxidoreductase activity, acting on diphenols and related substances as donors	11	**0.0012**
GO:0016128~phytosteroid metabolic process	7	**0.0014**
GO:0016131~brassinosteroid metabolic process	7	**0.0014**

GO:0044036~cell wall macromolecule metabolic process	13	**0.0015**
GO:0000271~polysaccharide biosynthetic process	21	**0.0015**
GO:0048518~positive regulation of biological process	34	**0.0015**
GO:0009639~response to red or far red light	29	**0.0015**
GO:0006694~steroid biosynthetic process	12	**0.0016**
GO:0043565~sequence-specific DNA binding	60	**0.0016**
GO:0031497~chromatin assembly	16	**0.0016**
GO:0009694~jasmonic acid metabolic process	9	**0.0016**
GO:0016563~transcription activator activity	24	**0.0017**
GO:0016702~oxidoreductase activity, acting on single donors with incorporation of molecular oxygen, incorporation of two atoms of oxygen	14	**0.0017**
GO:0008471~laccase activity	7	**0.0018**
GO:0048437~floral organ development	23	**0.0018**
GO:0065004~protein-DNA complex assembly	16	**0.0019**
GO:0044427~chromosomal part	26	**0.0021**
GO:0016701~oxidoreductase activity, acting on single donors with incorporation of molecular oxygen	15	**0.0021**
GO:0009751~response to salicylic acid stimulus	26	**0.0023**
GO:0048878~chemical homeostasis	22	**0.0024**
GO:0044459~plasma membrane part	39	**0.0024**
GO:0019904~protein domain specific binding	7	**0.0025**
GO:0019222~regulation of metabolic process	255	**0.0026**
GO:0046271~phenylpropanoid catabolic process	7	**0.0029**
GO:0046274~lignin catabolic process	7	**0.0029**
GO:0007017~microtubule-based process	22	**0.0030**
GO:0048569~post-embryonic organ development	29	**0.0031**
GO:0044264~cellular polysaccharide metabolic process	26	**0.0032**
GO:0016043~cellular component organization	144	**0.0033**
GO:0006323~DNA packaging	16	**0.0037**
GO:0048236~plant-type spore development	7	**0.0041**
GO:0050789~regulation of biological process	381	**0.0041**
GO:0004126~cytidine deaminase activity	5	**0.0042**
GO:0009937~regulation of gibberellic acid mediated signaling	6	**0.0043**
GO:0048316~seed development	57	**0.0046**
GO:0010468~regulation of gene expression	235	**0.0046**
GO:0010154~fruit development	59	**0.0049**
GO:0004838~L-tyrosine:2-oxoglutarate aminotransferase activity	4	**0.0050**
GO:0080087~callose binding	4	**0.0050**
GO:0070547~L-tyrosine aminotransferase activity	4	**0.0050**
GO:0005694~chromosome	31	**0.0051**
GO:0006325~chromatin organization	33	**0.0052**
GO:0048522~positive regulation of cellular process	23	**0.0053**
GO:0009644~response to high light intensity	11	**0.0054**
GO:0000041~transition metal ion transport	15	**0.0057**
GO:0009642~response to light intensity	15	**0.0057**
GO:0004312~fatty-acid synthase activity	6	**0.0057**
GO:0006826~iron ion transport	8	**0.0057**
GO:0046135~pyrimidine nucleoside catabolic process	5	**0.0060**
GO:0046133~pyrimidine ribonucleoside catabolic process	5	**0.0060**
GO:0010043~response to zinc ion	5	**0.0060**
GO:0048583~regulation of response to stimulus	25	**0.0060**
GO:0030244~cellulose biosynthetic process	10	**0.0061**
GO:0046283~anthocyanin metabolic process	6	**0.0062**
GO:0060255~regulation of macromolecule metabolic process	242	**0.0065**
GO:0010023~proanthocyanidin biosynthetic process	4	**0.0066**
GO:0016682~oxidoreductase activity, acting on diphenols and related substances as donors, oxygen as acceptor	8	**0.0066**
GO:0048229~gametophyte development	29	**0.0066**

GO:0009740~gibberellic acid mediated signaling	9	**0.0068**
GO:0010476~gibberellin-mediated signaling	9	**0.0068**
GO:0006869~lipid transport	24	**0.0073**
GO:0006996~organelle organization	74	**0.0077**
GO:0010876~lipid localization	26	**0.0080**
GO:0005783~endoplasmic reticulum	55	**0.0080**
GO:0044430~cytoskeletal part	28	**0.0082**
GO:0009553~embryo sac development	15	**0.0082**
GO:0045735~nutrient reservoir activity	13	**0.0083**
GO:0016746~transferase activity, transferring acyl groups	38	**0.0089**
GO:0022402~cell cycle process	22	**0.0091**
GO:0048366~leaf development	28	**0.0092**
GO:0009164~nucleoside catabolic process	5	**0.0093**
GO:0042454~ribonucleoside catabolic process	5	**0.0093**
GO:0033205~cytokinesis during cell cycle	7	**0.0093**
GO:0009922~fatty acid elongase activity	4	**0.0094**
GO:0010279~indole-3-acetic acid amido synthetase activity	4	**0.0094**
GO:0048467~gynoecium development	12	**0.0099**
GO:0030312~external encapsulating structure	72	**0.0102**
GO:0003993~acid phosphatase activity	10	**0.0104**
GO:0008289~lipid binding	32	**0.0105**
GO:0008092~cytoskeletal protein binding	18	**0.0106**
GO:0019842~vitamin binding	24	**0.0108**
GO:0032989~cellular component morphogenesis	33	**0.0113**
GO:0009737~response to abscisic acid stimulus	39	**0.0113**
GO:0015674~di-, tri-valent inorganic cation transport	12	**0.0113**
GO:0022622~root system development	29	**0.0115**
GO:0048364~root development	29	**0.0115**
GO:0010252~auxin homeostasis	6	**0.0117**
GO:0009556~microsporogenesis	6	**0.0117**
GO:0009606~tropism	11	**0.0118**
GO:0009867~jasmonic acid mediated signaling pathway	9	**0.0119**
GO:0005856~cytoskeleton	33	**0.0120**
GO:0009629~response to gravity	10	**0.0120**
GO:0016747~transferase activity, transferring acyl groups other than amino-acyl groups	35	**0.0121**
GO:0007049~cell cycle	33	**0.0135**
GO:0034655~nucleobase, nucleoside, nucleotide and nucleic acid catabolic process	5	**0.0135**
GO:0034656~nucleobase, nucleoside and nucleotide catabolic process	5	**0.0135**
GO:0042219~cellular amino acid derivative catabolic process	8	**0.0135**
GO:0007568~aging	14	**0.0140**
GO:0009617~response to bacterium	34	**0.0143**
GO:0005618~cell wall	70	**0.0143**
GO:0001708~cell fate specification	7	**0.0149**
GO:0044270~nitrogen compound catabolic process	7	**0.0149**
GO:0005507~copper ion binding	24	**0.0150**
GO:0000160~two-component signal transduction system (phosphorelay)	31	**0.0150**
GO:0048438~floral whorl development	18	**0.0153**
GO:0010436~carotenoid dioxygenase activity	4	**0.0154**
GO:0045549~9-cis-epoxycarotenoid dioxygenase activity	4	**0.0154**
GO:0040007~growth	34	**0.0159**
GO:0009574~preprophase band	5	**0.0160**
GO:0016627~oxidoreductase activity, acting on the CH-CH group of donors	13	**0.0162**
GO:0016705~oxidoreductase activity, acting on paired donors, with incorporation or reduction of molecular oxygen	27	**0.0162**

GO:0046700~heterocycle catabolic process	8	0.0163
GO:0051258~protein polymerization	8	0.0163
GO:0016036~cellular response to phosphate starvation	9	0.0166
GO:0032535~regulation of cellular component size	33	0.0169
GO:0016740~transferase activity	272	0.0172
GO:0005102~receptor binding	9	0.0176
GO:0015020~glucuronosyltransferase activity	5	0.0182
GO:0009887~organ morphogenesis	25	0.0183
GO:0051276~chromosome organization	35	0.0187
GO:0000325~plant-type vacuole	13	0.0188
GO:0007018~microtubule-based movement	14	0.0193
GO:0000226~microtubule cytoskeleton organization	9	0.0194
GO:0009269~response to desiccation	6	0.0196
GO:0007010~cytoskeleton organization	18	0.0198
GO:0009705~plant-type vacuole membrane	9	0.0201
GO:0005975~carbohydrate metabolic process	107	0.0205
GO:0016760~cellulose synthase (UDP-forming) activity	7	0.0208
GO:0022607~cellular component assembly	41	0.0212
GO:0015630~microtubule cytoskeleton	23	0.0213
GO:0005835~fatty acid synthase complex	3	0.0214
GO:0009630~gravitropism	9	0.0225
GO:0009850~auxin metabolic process	9	0.0225
GO:0009615~response to virus	9	0.0225
GO:0016126~sterol biosynthetic process	8	0.0229
GO:0080046~quercetin 4'-O-glucosyltransferase activity	4	0.0231
GO:0016165~lipoxygenase activity	4	0.0231
GO:0008361~regulation of cell size	31	0.0237
GO:0000902~cell morphogenesis	29	0.0239
GO:0032271~regulation of protein polymerization	6	0.0247
GO:0010035~response to inorganic substance	64	0.0250
GO:0031324~negative regulation of cellular metabolic process	15	0.0255
GO:0048584~positive regulation of response to stimulus	11	0.0256
GO:0015197~peptide transporter activity	6	0.0257
GO:0015198~oligopeptide transporter activity	6	0.0257
GO:0042446~hormone biosynthetic process	9	0.0259
GO:0042401~biogenic amine biosynthetic process	9	0.0259
GO:0009790~embryonic development	51	0.0266
GO:0051493~regulation of cytoskeleton organization	7	0.0268
GO:0048481~ovule development	8	0.0269
GO:0005874~microtubule	18	0.0286
GO:0016709~oxidoreductase activity, acting on paired donors, with incorporation or reduction of molecular oxygen, NADH or NADPH as one donor, and incorporation of one atom of oxygen	10	0.0287
GO:0044262~cellular carbohydrate metabolic process	61	0.0289
GO:0000910~cytokinesis	9	0.0297
GO:0009972~cytidine deamination	4	0.0299
GO:0046087~cytidine metabolic process	4	0.0299
GO:0006216~cytidine catabolic process	4	0.0299
GO:0006260~DNA replication	19	0.0302
GO:0044087~regulation of cellular component biogenesis	6	0.0305
GO:0010647~positive regulation of cell communication	6	0.0305
GO:0043254~regulation of protein complex assembly	6	0.0305
GO:0048440~carpel development	10	0.0313
GO:0004366~glycerol-3-phosphate O-acyltransferase activity	4	0.0326
GO:0016139~glycoside catabolic process	5	0.0329
GO:0016049~cell growth	29	0.0331
GO:0009119~ribonucleoside metabolic process	9	0.0339
GO:0008415~acyltransferase activity	28	0.0342
GO:0016759~cellulose synthase activity	8	0.0343

GO:0015631~tubulin binding	8	0.0343
GO:0009886~post-embryonic morphogenesis	13	0.0349
GO:0043086~negative regulation of catalytic activity	12	0.0355
GO:0016125~sterol metabolic process	8	0.0361
GO:0030170~pyridoxal phosphate binding	17	0.0363
GO:0070279~vitamin B6 binding	17	0.0363
GO:0070704~sterol desaturase activity	3	0.0367
GO:0047215~indole-3-acetate beta-glucosyltransferase activity	3	0.0367
GO:0019239~deaminase activity	5	0.0381
GO:0009250~glucan biosynthetic process	13	0.0383
GO:0042594~response to starvation	12	0.0391
GO:0035315~hair cell differentiation	11	0.0396
GO:0010026~trichome differentiation	11	0.0396
GO:0042440~pigment metabolic process	16	0.0403
GO:0048037~cofactor binding	56	0.0407
GO:0012505~endomembrane system	386	0.0410
GO:0010227~floral organ abscission	5	0.0417
GO:0016129~phytosteroid biosynthetic process	4	0.0419
GO:0016132~brassinosteroid biosynthetic process	4	0.0419
GO:0031224~intrinsic to membrane	256	0.0423
GO:0005976~polysaccharide metabolic process	29	0.0427
GO:0005774~vacuolar membrane	13	0.0433
GO:0016143~S-glycoside metabolic process	9	0.0433
GO:0019757~glycosinolate metabolic process	9	0.0433
GO:0009741~response to brassinosteroid stimulus	9	0.0433
GO:0019760~glucosinolate metabolic process	9	0.0433
GO:0045165~cell fate commitment	7	0.0438
GO:0015692~lead ion transport	3	0.0441
GO:0032101~regulation of response to external stimulus	3	0.0441
GO:0032104~regulation of response to extracellular stimulus	3	0.0441
GO:0032107~regulation of response to nutrient levels	3	0.0441
GO:0009833~primary cell wall biogenesis	3	0.0441
GO:0080027~response to herbivore	3	0.0441
GO:0007242~intracellular signaling cascade	87	0.0471
GO:0031669~cellular response to nutrient levels	12	0.0471
GO:0009267~cellular response to starvation	11	0.0482
GO:0010038~response to metal ion	46	0.0486
GO:0009809~lignin biosynthetic process	9	0.0487

Down-regulated Functional category

Term	Number of genes	P- Value
GO:0045087~innate immune response	52	0.0000
GO:0006955~immune response	54	0.0000
GO:0002376~immune system process	55	0.0000
GO:0006457~protein folding	48	0.0000
GO:0004888~transmembrane receptor activity	44	0.0000
GO:0012501~programmed cell death	44	0.0000
GO:0006915~apoptosis	37	0.0000
GO:0031072~heat shock protein binding	32	0.0000
GO:0016265~death	45	0.0000
GO:0008219~cell death	45	0.0000
GO:0009507~chloroplast	343	0.0000
GO:0009536~plastid	347	0.0000
GO:0005488~binding	1109	0.0000
GO:0051082~unfolded protein binding	24	0.0000
GO:0006800~oxygen and reactive oxygen species metabolic process	22	0.0001
GO:0004872~receptor activity	78	0.0001

GO:0034614~cellular response to reactive oxygen species	20	**0.0001**
GO:0034599~cellular response to oxidative stress	20	**0.0001**
GO:0070301~cellular response to hydrogen peroxide	18	**0.0002**
GO:0042744~hydrogen peroxide catabolic process	18	**0.0002**
GO:0020037~heme binding	53	**0.0002**
GO:0042743~hydrogen peroxide metabolic process	18	**0.0003**
GO:0055114~oxidation reduction	123	**0.0003**
GO:0046983~protein dimerization activity	39	**0.0003**
GO:0004713~protein tyrosine kinase activity	45	**0.0006**
GO:0046914~transition metal ion binding	278	**0.0007**
GO:0009570~chloroplast stroma	56	**0.0008**
GO:0046906~tetrapyrrole binding	54	**0.0008**
GO:0000302~response to reactive oxygen species	23	**0.0009**
GO:0044434~chloroplast part	109	**0.0010**
GO:0009055~electron carrier activity	79	**0.0013**
GO:0009532~plastid stroma	56	**0.0024**
GO:0006468~protein amino acid phosphorylation	107	**0.0025**
GO:0044435~plastid part	109	**0.0028**
GO:0004601~peroxidase activity	21	**0.0031**
GO:0016684~oxidoreductase activity, acting on peroxide as acceptor	21	**0.0031**
GO:0060089~molecular transducer activity	88	**0.0034**
GO:0004871~signal transducer activity	88	**0.0034**
GO:0043167~ion binding	344	**0.0037**
GO:0016491~oxidoreductase activity	157	**0.0038**
GO:0043169~cation binding	343	**0.0038**
GO:0044436~thylakoid part	46	**0.0038**
GO:0016209~antioxidant activity	23	**0.0040**
GO:0046872~metal ion binding	327	**0.0044**
GO:0031976~plastid thylakoid	45	**0.0045**
GO:0009534~chloroplast thylakoid	45	**0.0045**
GO:0031984~organelle subcompartment	45	**0.0050**
GO:0043687~post-translational protein modification	134	**0.0052**
GO:0000166~nucleotide binding	320	**0.0059**
GO:0003677~DNA binding	240	**0.0061**
GO:0005524~ATP binding	231	**0.0067**
GO:0042542~response to hydrogen peroxide	18	**0.0074**
GO:0045038~protein import into chloroplast thylakoid membrane	4	**0.0074**
GO:0007165~signal transduction	115	**0.0081**
GO:0005506~iron ion binding	81	**0.0081**
GO:0051716~cellular response to stimulus	96	**0.0083**
GO:0016998~cell wall macromolecule catabolic process	8	**0.0092**
GO:0032559~adenyl ribonucleotide binding	232	**0.0093**
GO:0006979~response to oxidative stress	34	**0.0118**
GO:0006950~response to stress	209	**0.0123**
GO:0009579~thylakoid	57	**0.0132**
GO:0008270~zinc ion binding	166	**0.0132**
GO:0001883~purine nucleoside binding	244	**0.0147**
GO:0030554~adenyl nucleotide binding	244	**0.0147**
GO:0004518~nuclease activity	26	**0.0160**
GO:0009734~auxin mediated signaling pathway	17	**0.0160**
GO:0008937~ferredoxin reductase activity	4	**0.0163**
GO:0016731~oxidoreductase activity, acting on iron-sulfur proteins as donors, NAD or NADP as acceptor	4	**0.0163**
GO:0001882~nucleoside binding	244	**0.0166**
GO:0032553~ribonucleotide binding	251	**0.0181**
GO:0032555~purine ribonucleotide binding	251	**0.0181**
GO:0033903~endo-1,3(4)-beta-glucanase activity	3	**0.0203**
GO:0009535~chloroplast thylakoid membrane	36	**0.0207**

GO:0055035~plastid thylakoid membrane	36	**0.0207**
GO:0016790~thiolester hydrolase activity	16	**0.0208**
GO:0006464~protein modification process	145	**0.0216**
GO:0005515~protein binding	343	**0.0221**
GO:0045036~protein targeting to chloroplast	6	**0.0226**
GO:0051276~chromosome organization	30	**0.0229**
GO:0017076~purine nucleotide binding	264	**0.0257**
GO:0042651~thylakoid membrane	37	**0.0260**
GO:0006325~chromatin organization	26	**0.0278**
GO:0008080~N-acetyltransferase activity	10	**0.0291**
GO:0010051~xylem and phloem pattern formation	8	**0.0299**
GO:0032870~cellular response to hormone stimulus	45	**0.0303**
GO:0009755~hormone-mediated signaling	45	**0.0303**
GO:0048511~rhythmic process	9	**0.0307**
GO:0016310~phosphorylation	108	**0.0314**
GO:0005529~sugar binding	18	**0.0322**
GO:0004672~protein kinase activity	112	**0.0339**
GO:0044036~cell wall macromolecule metabolic process	9	**0.0342**
GO:0016730~oxidoreductase activity, acting on iron-sulfur proteins as donors	4	**0.0345**
GO:0010305~leaf vascular tissue pattern formation	5	**0.0360**
GO:0004392~heme oxygenase (decyclizing) activity	3	**0.0383**
GO:0009733~response to auxin stimulus	36	**0.0384**
GO:0016706~oxidoreductase activity, acting on paired donors, with incorporation or reduction of molecular oxygen, 2-oxoglutarate as one donor, and incorporation of one atom each of oxygen into both donors	13	**0.0393**
GO:0004386~helicase activity	23	**0.0396**
GO:0017111~nucleoside-triphosphatase activity	83	**0.0400**
GO:0030246~carbohydrate binding	27	**0.0410**
GO:0043412~biopolymer modification	154	**0.0458**
GO:0010588~cotyledon vascular tissue pattern formation	4	**0.0460**
GO:0043226~organelle	785	**0.0465**
GO:0008037~cell recognition	7	**0.0469**
GO:0048544~recognition of pollen	7	**0.0469**
GO:0034357~photosynthetic membrane	38	**0.0473**
GO:0043229~intracellular organelle	785	**0.0485**
GO:0016410~N-acyltransferase activity	10	**0.0489**
GO:0010345~suberin biosynthetic process	3	**0.0498**

Up-regulated Pathway categories

Term	Number of genes	P- Value
ath00941:Flavonoid biosynthesis	8	**0.0008**
P00049:Parkinson disease	18	**0.0018**
ath01070:Biosynthesis of plant hormones	42	**0.0035**
ath00592:alpha-Linolenic acid metabolism	8	**0.0099**
ath01062:Biosynthesis of terpenoids and steroids	28	**0.0112**
ath00905:Brassinosteroid biosynthesis	4	**0.0117**
P00059:p53 pathway	11	**0.0154**
P00006:Apoptosis signaling pathway	11	**0.0171**
ath01061:Biosynthesis of phenylpropanoids	33	**0.0179**
P00048:PI3 kinase pathway	7	**0.0219**
ath00908:Zeatin biosynthesis	6	**0.0346**
ath00940:Phenylpropanoid biosynthesis	16	**0.0421**

Down-regulated Pathway categories

Term	Number of genes	P- Value
ath00680:Methane metabolism	17	**0.0001**
ath00940:Phenylpropanoid biosynthesis	18	**0.0005**
ath00360:Phenylalanine metabolism	14	**0.0023**
ath00945:Stilbenoid, diarylheptanoid and gingerol biosynthesis	11	**0.0121**
ath00903:Limonene and pinene degradation	11	**0.0164**
ath03060:Protein export	6	**0.0201**
ath00910:Nitrogen metabolism	8	**0.0210**

Supplementary material 7: List of Bins with their names and normalized p-value used in Ma

Bins	Name	P-value
10	cell wall	0
20.1.7.12	stress.biotic.PR-proteins.plant defensins	0
29.2.1.2	protein.synthesis.ribosomal protein.eukaryotic	0
29.2.1	protein.synthesis.ribosomal protein	8.88178E-16
29.2.1.2.1	protein.synthesis.ribosomal protein.eukaryotic.40S subunit	1.60525E-11
29.4	protein.postranslational modification	2.09438E-11
20.2.1	stress.abiotic.heat	2.9415E-10
26.21	misc.protease inhibitor/seed storage/lipid transfer protein (LTP) family protein	7.80184E-10
35.1.5	not assigned.no ontology.pentatricopeptide (PPR) repeat-containing protein	2.10511E-09
26.28	misc.GDSL-motif lipase	5.76489E-09
29.2.1.2.2	protein.synthesis.ribosomal protein.eukaryotic.60S subunit	6.77337E-09
29.2	protein.synthesis	4.00594E-08
30	signalling	2.67655E-07
16.7	secondary metabolism.wax	3.54081E-07
30.2.3	signalling.receptor kinases.leucine rich repeat III	4.75969E-07
30.2.17	signalling.receptor kinases.DUF 26	5.49564E-07
10.5.1	cell wall.cell wall proteins.AGPs	5.77453E-07
26.19	misc.plastocyanin-like	8.09148E-07
27.3.32	RNA.regulation of transcription.WRKY domain transcription factor family	9.57122E-07
17	hormone metabolism	1.03302E-06
28.1.3	DNA.synthesis/chromatin structure.histone	4.57722E-06
29.4.1	protein.postranslational modification.kinase	4.76782E-06
10.7	cell wall.modification	5.576E-06
3.5	minor CHO metabolism.others	1.14161E-05
29.5.11.4.3	protein.degradation.ubiquitin.E3.SCF	1.97895E-05
27.3.41	RNA.regulation of transcription.B3 transcription factor family	2.46821E-05
10.6.3	cell wall.degradation.pectate lyases and polygalacturonases	3.10815E-05
17.2.3	hormone metabolism.auxin.induced-regulated-responsive-activated	3.32029E-05
20.1.7	stress.biotic.PR-proteins	3.95973E-05
11.4	lipid metabolism.TAG synthesis	5.20752E-05
27.1.1	RNA.processing.splicing	5.64054E-05
29.4.1.57	protein.postranslational modification.kinase.receptor like cytoplasmatic kinase VII	6.87772E-05
17.6	hormone metabolism.gibberelin	8.79341E-05
10.8.99	cell wall.pectin*esterases.misc	8.96677E-05
29.5.7	protein.degradation.metalloprotease	0.000131563
11.1	lipid metabolism.FA synthesis and FA elongation	0.000151908
30.2.25	signalling.receptor kinases.wall associated kinase	0.000171498
17.2	hormone metabolism.auxin	0.000227617
10.6	cell wall.degradation	0.000233265
26.4	misc.beta 1,3 glucan hydrolases	0.000236293
29.5.11.4.3.2	protein.degradation.ubiquitin.E3.SCF.FBOX	0.000264372
11.1.10	lipid metabolism.FA synthesis and FA elongation.beta ketoacyl CoA synthase	0.000265685
35.1.21	not assigned.no ontology.epsin N-terminal homology (ENTH) domain-containing pro	0.000284465
27.3.1	RNA.regulation of transcription.ABI3/VP1-related B3-domain-containing transcripti	0.000357717
10.5	cell wall.cell wall proteins	0.000382628
20.2.3	stress.abiotic.drought/salt	0.000389247
34.8	transport.metabolite transporters at the envelope membrane	0.000406728
27.1	RNA.processing	0.000449219
26.3.4	misc.gluco-, galacto- and mannosidases.endoglucanase	0.000466589
10.8	cell wall.pectin*esterases	0.000494611
17.6.3	hormone metabolism.gibberelin.induced-regulated-responsive-activated	0.000563582
1	PS	0.000569326
17.3	hormone metabolism.brassinosteroid	0.000634884

30.3	signalling.calcium	0.000686324
17.3.1	hormone metabolism.brassinosteroid.synthesis-degradation	0.000817501
26.1	misc.misc2	0.000928231
35.1.8	not assigned.no ontology.BSD domain-containing protein	0.000955535
33	development	0.001006017
29.8	protein.assembly and cofactor ligation	0.001093822
27.3.80	RNA.regulation of transcription.zf-HD	0.001202283
30.2.13	signalling.receptor kinases.leucine rich repeat XIII	0.001335132
27.3.52	RNA.regulation of transcription.Global transcription factor group	0.001335748
11	lipid metabolism	0.001468945
27.3.10	RNA.regulation of transcription.C2C2(Zn) YABBY family	0.001532093
30.2.7	signalling.receptor kinases.leucine rich repeat VII	0.00155352
29.5.1	protein.degradation.subtilases	0.001654204
26.8	misc.nitrilases, *nitrile lyases, berberine bridge enzymes, reticuline oxidases, troponi	0.001691843
10.5.4	cell wall.cell wall proteins.HRGP	0.001799806
35.1.22	not assigned.no ontology.late embryogenesis abundant domain-containing protein	0.001949375
13.1.6	amino acid metabolism.synthesis.aromatic aa	0.002486261
27.3.9	RNA.regulation of transcription.C2C2(Zn) GATA transcription factor family	0.002653566
27.3.14	RNA.regulation of transcription.CCAAT box binding factor family, HAP2	0.002700364
30.2.20	signalling.receptor kinases.wheat LRK10 like	0.002762455
20.2	stress.abiotic	0.003097627
33.99	development.unspecified	0.003253772
27.3.29	RNA.regulation of transcription.TCP transcription factor family	0.003359612
20.1.2	stress.biotic.receptors	0.00338976
14	S-assimilation	0.003407025
11.1.12	lipid metabolism.FA synthesis and FA elongation.ACP protein	0.003680423
30.4	signalling.phosphinositides	0.004363324
17.6.1.13	hormone metabolism.gibberelin.synthesis-degradation.GA2 oxidase	0.0048055
30.2.16	signalling.receptor kinases.Catharanthus roseus-like RLK1	0.004976964
10.2	cell wall.cellulose synthesis	0.005155882
30.2.24	signalling.receptor kinases.S-locus glycoprotein like	0.005436124
29.5.11.4.3.1	protein.degradation.ubiquitin.E3.SCF.SKP	0.005447694
34.13	transport.peptides and oligopeptides	0.005847368
21.1	redox.thioredoxin	0.005998359
21.4	redox.glutaredoxins	0.006142178
29.5.4	protein.degradation.aspartate protease	0.006319734
18	Co-factor and vitamine metabolism	0.006671858
13.1.6.5	amino acid metabolism.synthesis.aromatic aa.tryptophan	0.006758265
1.1.6	PS.lightreaction.NADH DH	0.00684309
29	protein	0.006885524
8.3	TCA / org. transformation.carbonic anhydrases	0.007334831
11.6	lipid metabolism.lipid transfer proteins etc	0.008291534
13.1.1.3	amino acid metabolism.synthesis.central amino acid metabolism.alanine	0.008500813
17.3.1.1	hormone metabolism.brassinosteroid.synthesis-degradation.BRs	0.008631142
13.1.1.3.1	amino acid metabolism.synthesis.central amino acid metabolism.alanine.alanine amir	0.008915676
17.6.1.11	hormone metabolism.gibberelin.synthesis-degradation.GA20 oxidase	0.009022883
29.2.1.2.2.82	protein.synthesis.ribosomal protein.eukaryotic.60S subunit.P2	0.009872045
3.2	minor CHO metabolism.trehalose	0.010227501
29.4.1.59	protein.postranslational modification.kinase.receptor like cytoplasmatic kinase IX	0.010475802
30.2.15	signalling.receptor kinases.thaumatin like	0.011048342
26	misc	0.011217075
1.1.5.3	PS.lightreaction.other electron carrier (ox/red).ferredoxin reductase	0.011487263
3.2.1	minor CHO metabolism.trehalose.TPS	0.011651487
16.1	secondary metabolism.simple phenols	0.011727781
26.24	misc.GCN5-related N-acetyltransferase	0.01206135
11.5.2	lipid metabolism.glyceral metabolism.Glycerol-3-phosphate dehydrogenase (NAD+)	0.012278261
3.2.2	minor CHO metabolism.trehalose.TPP	0.012615494
21.2	redox.ascorbate and glutathione	0.013272539

14.1	S-assimilation.APS	0.014125754
27.3.73	RNA.regulation of transcription.Zn-finger(CCHC)	0.01477027
29.3.4	protein.targeting.secretory pathway	0.015025832
16.8.1.1	secondary metabolism.flavonoids.anthocyanins.leucocyanidin oxygenase	0.015070825
29.2.99	protein.synthesis.misc	0.015129737
16.5.1.3.3	secondary metabolism.sulfur-containing.glucosinolates.degradation.nitrilase	0.016167547
29.3.3	protein.targeting.chloroplast	0.016171814
16.8.3.1	secondary metabolism.flavonoids.dihydroflavonols.dihydrokaempferol 4-reductase	0.017158364
17.7.1.5	hormone metabolism.jasmonate.synthesis-degradation.12-Oxo-PDA-reductase	0.017218443
31.3	cell.cycle	0.017861337
33.3	development.squamosa promoter binding like (SPL)	0.019078503
26.1	misc.cytochrome P450	0.01951085
17.5.3	hormone metabolism.ethylene.induced-regulated-responsive-activated	0.020111612
30.2.9	signalling.receptor kinases.leucine rich repeat IX	0.020597085
1.1	PS.lightreaction	0.02109758
1.2	PS.photorespiration	0.021114347
29.3.5	protein.targeting.peroxisomes	0.021690896
30.2.4	signalling.receptor kinases.leucine rich repeat IV	0.022312313
27.3.40	RNA.regulation of transcription.Aux/IAA family	0.022967211
11.3.3	lipid metabolism.Phospholipid synthesis.phosphatidate cytidylyltransferase	0.023343342
29.2.1.2.1.17	protein.synthesis.ribosomal protein.eukaryotic.40S subunit.S17	0.023534604
30.9	signalling.lipids	0.023699289
26.4.1	misc.beta 1,3 glucan hydrolases.glucan endo-1,3-beta-glucosidase	0.023889211
11.1.11	lipid metabolism.FA synthesis and FA elongation.fatty acid elongase	0.024048573
21.6	redox.dismutases and catalases	0.024563978
29.3.4.3	protein.targeting.secretory pathway.vacuole	0.02460487
21	redox	0.025198092
27.3.8	RNA.regulation of transcription.C2C2(Zn) DOF zinc finger family	0.02554474
11.3.6	lipid metabolism.Phospholipid synthesis.choline-phosphate cytidylyltransferase	0.025647728
13.2.4.4	amino acid metabolism.degradation.branched-chain group.leucine	0.025852935
29.2.1.1.3.2	protein.synthesis.ribosomal protein.prokaryotic.unknown organellar.50S subunit	0.027620755
13.1.6.1	amino acid metabolism.synthesis.aromatic aa.chorismate	0.02770433
16.8.4	secondary metabolism.flavonoids.flavonols	0.027715863
20.1.3	stress.biotic.signalling	0.027797018
30.2.8	signalling.receptor kinases	0.028910755
35.1.42	not assigned.no ontology.proline rich family	0.029747601
10.5.2	cell wall.cell wall proteins.proline rich proteins	0.03040922
17.3.1.2	hormone metabolism.brassinosteroid.synthesis-degradation.sterols	0.030879451
16.8.4.1	secondary metabolism.flavonoids.flavonols.dihydrokaempferol 4-reductase	0.031579472
29.3	protein.targeting	0.031617945
29.2.1.1	protein.synthesis.ribosomal protein.prokaryotic	0.033043305
27.3.35	RNA.regulation of transcription.bZIP transcription factor family	0.033165034
13.1.6.5.5	amino acid metabolism.synthesis.aromatic aa.tryptophan.tryptophan synthase	0.034502533
27.3.36	RNA.regulation of transcription.Argonaute	0.034688039
1.1.5	PS.lightreaction.other electron carrier (ox/red)	0.034841083
10.1.12	cell wall.precursor synthesis.KDO pathway	0.035119656
30.7	signalling.14-3-3 proteins	0.036196273
27.3.99	RNA.regulation of transcription.unclassified	0.036358735
16.8.1.21	secondary metabolism.flavonoids.anthocyanins.anthocyanin 5-aromatic acyltransfera	0.036494554
27.4	RNA.RNA binding	0.036579604
16	secondary metabolism	0.03714996
29.2.1.2.2.81	protein.synthesis.ribosomal protein.eukaryotic.60S subunit.P1	0.037497251
15.2	metal handling.binding, chelation and storage	0.038294312
29.2.1.2.1.11	protein.synthesis.ribosomal protein.eukaryotic.40S subunit.S11	0.038829164
29.2.1.2.1.15	protein.synthesis.ribosomal protein.eukaryotic.40S subunit.S15	0.03914259
10.6.1	cell wall.degradation.cellulases and beta -1,4-glucanases	0.0396184
34	transport	0.039848321
13	amino acid metabolism	0.040245246

10.3	cell wall.hemicellulose synthesis	0.040493885
17.4.1	hormone metabolism.cytokinin.synthesis-degradation	0.040697319
29.1.22	protein.aa activation.asparagine-tRNA ligase	0.04092272
3	minor CHO metabolism	0.041282792
27.3.66	RNA.regulation of transcription.Psudo ARR transcription factor family	0.041354873
27.3.71	RNA.regulation of transcription.SNF7	0.041396223
10.1.4	cell wall.precursor synthesis.UGD	0.041501876
29.5.9	protein.degradation.AAA type	0.042426528
34.19	transport.Major Intrinsic Proteins	0.04250865
35.1	not assigned.no ontology	0.043164097
29.2.1.2.1.25	protein.synthesis.ribosomal protein.eukaryotic.40S subunit.S25	0.043757296
29.5.11.4.5.1	protein.degradation.ubiquitin.E3.BTB/POZ Cullin3.Cullin3	0.043967882
30.1	signalling.in sugar and nutrient physiology	0.044114771
17.1.3	hormone metabolism.abscisic acid.induced-regulated-responsive-activated	0.044327721
30.8	signalling.misc	0.045462772
27.3.3	RNA.regulation of transcription.AP2/EREBP, APETALA2/Ethylene-responsive elem	0.045507464
10.2.2	cell wall.cellulose synthesis.COBRA	0.045897048
1.1.40	PS.lightreaction.cyclic electron flow-chlororespiration	0.046865323
23.1.99	nucleotide metabolism.synthesis.unspecified	0.047421445
8.1.4	TCA / org. transformation.TCA.IDH	0.047961762
11.1.5	lipid metabolism.FA synthesis and FA elongation.beta hydroxyacyl ACP dehydratase	0.048645432
27.3.37	RNA.regulation of transcription.AS2,Lateral Organ Boundaries Gene Family	0.049217439
30.2.8.2	signalling.receptor kinases.leucine rich repeat VIII-2	0.049719082
26.13	misc.acid and other phosphatases	0.049871408

apman

Supplementary material 8: List of up-regulated genes with MaRAP2-4 interacting *cis*-elements

DRE	JARE	GCC
AT4G15750	AT5G20630	AT5G50790
AT2G42840	AT4G12870	AT3G26140
AT5G45670	AT1G16390	AT1G10640
AT4G29030	AT2G10940	AT4G35725
AT4G12870	AT3G16370	AT4G14750
AT5G39220	AT3G17600	AT3G10570
AT5G50790	AT5G63230	AT1G15150
AT3G54340	AT5G51490	AT5G07540
AT1G53130	AT2G32370	AT4G33790
AT5G01075	AT4G26590	AT5G48100
AT5G08000	AT4G14750	AT4G31840
AT3G04280	AT1G70560	AT1G78760
AT5G51950	AT5G24780	AT5G12940
AT5G09370	AT5G47150	AT2G29790
AT4G12890	AT5G38180	AT1G80100
AT1G26945	AT1G31310	AT2G28040
AT3G17600	AT1G43640	AT2G38530
AT1G55260	AT1G49770	AT4G02830
AT3G12610	AT3G50870	AT1G28360
AT5G25460	AT1G28590	AT2G34870
AT1G10640	AT1G09380	AT5G66590
AT4G39330	AT3G18470	AT1G01110
AT2G35310	AT3G61970	AT3G14190
AT2G27880	AT2G37380	AT1G68460
AT5G01790	AT2G38995	AT1G50110
AT1G52695	AT5G08640	AT4G29150
AT1G34580	AT4G22430	AT2G14620
AT2G42110	AT5G12270	AT4G27330
AT4G15210	AT1G68500	AT5G59040
AT4G28780	AT4G30610	AT1G24590
AT1G31310	AT1G44760	AT5G02890
AT3G43960	AT5G57920	AT5G18030
AT4G17480	AT1G23200	AT1G08560
AT3G05980	AT5G09970	AT3G46320
AT5G48100	AT5G47920	AT5G17220
AT2G20870	AT1G70720	AT4G25480
AT3G13540	AT5G45960	AT2G33850
AT4G23600	AT4G01080	AT1G47610
AT5G48900	AT5G40040	AT5G51480
AT1G06360	AT4G02290	AT1G49430
AT5G53390	AT3G23140	AT1G34640
AT2G02990	AT4G24275	AT4G18920
AT1G78760	AT5G55132	AT1G17560
AT5G12940	AT3G17760	AT1G65900
AT2G37640	AT5G62170	AT2G47880
AT3G61970	AT5G13140	AT3G15357
AT3G54550	AT1G52690	AT3G13175
AT1G78170	AT5G02890	AT2G38760
AT5G01180	AT3G16670	AT4G28430
AT2G38530	AT1G08560	AT3G61490
AT4G10290	AT5G23940	AT3G52810
AT1G67750	AT3G46320	AT1G53140

DRE+JARE	DRE+GCC	JARE+GCC
AT4G12870	AT5G50790	AT4G14750
AT3G17600	AT1G10640	AT5G02890
AT1G31310	AT5G48100	AT1G08560
AT3G61970	AT1G78760	AT3G46320
AT1G44760	AT5G12940	AT5G17220
AT5G09970	AT2G38530	AT2G33850
AT1G70720	AT2G34870	AT4G12690
AT4G02290	AT5G66590	AT4G23380
AT3G17760	AT3G14190	AT1G02460
AT5G62170	AT1G68460	AT3G24220
AT5G02890	AT1G50110	AT4G23800
AT3G16670	AT5G02890	AT1G25450
AT1G08560	AT1G08560	AT5G44110
AT5G23940	AT5G17220	AT3G52340
AT5G17220	AT4G25480	AT5G10720
AT4G18570	AT2G33850	AT2G13820
AT4G13840	AT5G51480	AT3G11600
AT2G14900	AT1G49430	AT3G08030
AT5G02540	AT2G47880	AT3G14740
AT1G70710	AT3G15357	AT2G35585
AT4G02800	AT3G13175	AT2G45360
AT1G76500	AT3G61490	AT1G71870
AT3G20820	AT3G52810	AT3G59140
AT2G33850	AT1G53140	AT3G04850
AT4G33270	AT4G11310	AT5G35670
AT1G34355	AT2G26250	
AT3G59890	AT3G19270	
AT1G12010	AT1G67035	
AT4G34220	AT1G60060	
AT2G02100	AT4G28250	
AT1G52190	AT5G61570	
AT1G26770	AT4G10190	
AT1G75710	AT1G02460	
AT4G33260	AT4G01410	
AT4G38660	AT2G17630	
AT2G15440	AT1G09540	
AT3G57510	AT1G54040	
AT4G36380	AT4G23800	
AT3G07010	AT2G34650	
AT4G35190	AT5G44110	
AT5G67360	AT4G12730	
AT1G02460	AT1G55740	
AT1G29460	AT3G12700	
AT4G13710	AT2G38110	
AT5G42280	AT5G39320	
AT3G49220	AT1G19940	
AT4G00160	AT1G29660	
AT4G23800	AT3G08030	
AT1G68570	AT2G37560	
AT2G43820	AT3G03170	
AT5G44110	AT2G35585	
AT5G10430	AT5G11230	

DRE+JARE+GCC
AT5G02890
AT1G08560
AT5G17220
AT2G33850
AT1G02460
AT4G23800
AT5G44110
AT3G08030
AT2G35585
AT1G71870
AT3G59140
AT3G04850

AT3G29370	AT5G17220	AT4G11310
AT2G34870	AT3G48350	AT1G71695
AT3G53530	AT4G39650	AT2G07170
AT5G47590	AT3G09950	AT1G01610
AT1G79840	AT1G68450	AT4G12690
AT2G42590	AT4G18570	AT2G26250
AT2G45470	AT4G13840	AT5G14410
AT1G44760	AT2G14900	AT5G55480
AT5G07280	AT5G02540	AT5G65010
AT2G23800	AT1G70710	AT1G19830
AT5G17430	AT5G11130	AT3G04290
AT5G18290	AT4G02800	AT3G19270
AT5G09970	AT1G76500	AT5G27330
AT5G07200	AT3G20820	AT1G67035
AT1G05490	AT5G23400	AT4G23380
AT3G02480	AT2G33850	AT1G60060
AT5G66590	AT4G33270	AT4G28250
AT5G43020	AT5G40630	AT5G61570
AT1G70720	AT1G34355	AT1G77210
AT5G37810	AT3G28860	AT5G28000
AT5G03120	AT3G50740	AT4G10190
AT4G02290	AT3G55660	AT2G43360
AT1G78220	AT3G54180	AT1G02460
AT4G00400	AT3G59890	AT4G01410
AT5G02580	AT2G26180	AT3G24220
AT2G01520	AT4G23870	AT2G17630
AT3G21420	AT2G45420	AT1G09540
AT1G02070	AT1G12010	AT1G54040
AT2G40170	AT5G24790	AT4G23800
AT1G30920	AT4G34220	AT1G25450
AT2G13610	AT2G02100	AT2G34650
AT4G29285	AT4G10895	AT5G01910
AT4G02330	AT4G24260	AT5G44110
AT3G14190	AT1G52190	AT5G07500
AT1G68460	AT1G26770	AT3G52340
AT5G67110	AT1G75710	AT1G62640
AT1G50110	AT4G33260	AT4G12730
AT2G42540	AT1G80280	AT3G27060
AT5G05220	AT3G23890	AT1G72130
AT3G21620	AT1G48480	AT1G55740
AT3G17760	AT2G21650	AT5G10720
AT5G62170	AT3G10520	AT1G02180
AT3G63430	AT4G38660	AT3G12700
AT5G66580	AT4G39590	AT4G37580
AT2G31980	AT1G61070	AT2G13820
AT2G18500	AT2G15440	AT2G38110
AT5G46795	AT3G57510	AT1G75390
AT5G02890	AT3G24340	AT3G11600
AT1G05260	AT4G12690	AT5G39320
AT1G70270	AT4G36380	AT1G19940
AT5G16250	AT4G28680	AT5G05965
AT3G03820	AT3G07010	AT1G29660
AT3G16670	AT5G09210	AT3G08030
AT3G02640	AT4G35190	AT2G37560
AT5G20240	AT2G27040	AT5G08260
AT1G08560	AT4G23380	AT1G48100
AT3G17520	AT1G10680	AT3G14740
AT2G45050	AT5G67360	AT3G03170

AT5G65410	AT1G71870
AT3G23670	AT1G34430
AT4G25850	AT2G43550
AT3G22880	AT3G59140
AT1G70680	AT2G26640
AT3G22770	AT3G13690
AT3G48460	AT3G04850
AT5G65420	AT3G52370
AT2G22900	
AT2G01660	
AT1G66730	
AT2G24230	
AT3G08030	
AT3G60530	
AT1G11740	
AT2G35730	
AT1G56430	
AT2G35585	
AT1G67600	
AT2G24170	
AT1G71870	
AT4G36105	
AT5G18660	
AT3G59140	
AT5G62360	
AT5G64850	
AT1G02450	
AT3G02170	
AT3G62630	
AT3G04850	
AT2G46970	

AT5G23940	AT1G02460	AT3G54750
AT4G32540	AT1G29460	AT5G02220
AT4G35670	AT5G48950	AT2G35585
AT5G17220	AT4G13710	AT5G11230
AT1G62340	AT5G42280	AT1G19510
AT2G05790	AT3G24220	AT2G45360
AT4G39070	AT3G49220	AT1G71870
AT4G36350	AT4G18760	AT1G02190
AT3G28290	AT4G00160	AT1G34430
AT3G51280	AT2G16850	AT2G43550
AT3G01490	AT2G36080	AT3G59140
AT4G18570	AT1G71770	AT2G26640
AT4G13840	AT4G23800	AT1G71020
AT2G14900	AT1G25450	AT1G75060
AT5G38170	AT3G52970	AT3G13690
AT4G25480	AT4G25960	AT2G26910
AT5G02540	AT1G68570	AT1G08280
AT1G70710	AT2G43820	AT1G72140
AT5G55730	AT5G44110	AT3G04850
AT4G02800	AT3G57860	AT5G10480
AT1G76500	AT5G20850	AT3G52370
AT5G42910	AT5G10430	AT5G35670
AT3G11520	AT5G65410	
AT5G55340	AT5G14920	
AT5G35550	AT4G11211	
AT4G12910	AT3G54250	
AT3G20820	AT3G52340	
AT5G65390	AT3G23670	
AT2G33850	AT3G01410	
AT1G07610	AT2G35640	
AT4G33270	AT4G25850	
AT3G11180	AT3G22880	
AT5G64080	AT5G10720	
AT5G51480	AT3G55360	
AT2G32765	AT1G70680	
AT2G36190	AT5G65710	
AT3G46490	AT1G23130	
AT1G34355	AT5G60490	
AT3G50620	AT3G22770	
AT1G79720	AT4G15530	
AT2G36560	AT5G14130	
AT5G50750	AT3G48460	
AT5G65730	AT5G65420	
AT4G37800	AT2G22900	
AT5G23650	AT2G13820	
AT4G27260	AT1G09200	
AT1G49430	AT5G51890	
AT5G18430	AT3G11600	
AT2G32990	AT3G43160	
AT4G32980	AT2G01660	
AT3G59890	AT4G36770	
AT4G23496	AT3G26770	
AT5G01015	AT1G49580	
AT1G48990	AT1G66730	
AT2G47880	AT4G38050	
AT4G14770	AT4G30800	
AT3G15357	AT2G24230	
AT5G66780	AT3G08030	

AT5G38160	AT3G60530
AT4G03010	AT1G11740
AT3G09870	AT2G35730
AT3G13175	AT3G47600
AT5G26850	AT1G10550
AT4G11290	AT3G46660
AT1G27030	AT3G14740
AT4G29610	AT4G10910
AT1G56580	AT1G56430
AT3G61490	AT1G51440
AT4G31290	AT2G35585
AT1G12010	AT1G67600
AT5G39160	AT4G20140
AT3G52810	AT2G24170
AT3G58070	AT2G45360
AT4G34220	AT1G71870
AT2G02100	AT5G44010
AT2G27535	AT2G16760
AT5G59970	AT1G19950
AT1G52190	AT4G36105
AT1G26770	AT1G51060
AT5G64330	AT2G24440
AT1G53140	AT4G04080
AT4G23750	AT5G18660
AT1G75710	AT1G07090
AT1G72810	AT3G59140
AT4G33260	AT1G21810
AT2G38170	AT5G62360
AT5G24580	AT4G32830
AT4G12880	AT1G70370
AT3G43720	AT5G64850
AT5G67270	AT1G02450
AT5G59320	AT2G30200
AT1G01250	AT3G29810
AT1G17140	AT2G23700
AT4G11310	AT3G02170
AT1G12080	AT1G70830
AT4G38660	AT3G62630
AT3G59220	AT5G44020
AT1G45010	AT3G04850
AT2G15440	AT2G46970
AT5G04160	AT4G00390
AT3G57510	AT4G14150
AT4G02810	AT5G35670
AT1G76310	
AT4G23490	
AT2G45570	
AT4G36700	
AT2G26250	
AT4G36380	
AT4G16980	
AT4G16515	
AT5G39130	
AT4G33280	
AT1G58360	
AT5G60220	
AT3G07010	
AT4G28720	

AT2G45970
AT5G20790
AT4G35190
AT1G19640
AT3G19270
AT2G39060
AT4G15680
AT4G16140
AT3G28220
AT2G23050
AT5G40310
AT1G67035
AT4G28250
AT5G61570
AT3G59420
AT1G60060
AT3G52820
AT2G35760
AT1G75750
AT3G16240
AT5G67360
AT4G10190
AT1G01900
AT1G07370
AT1G26100
AT4G01410
AT1G02460
AT1G29460
AT4G13710
AT5G42280
AT3G45230
AT4G31760
AT1G33170
AT2G02130
AT3G54560
AT3G49220
AT2G47780
AT2G17630
AT3G51470
AT1G77270
AT5G66800
AT4G00160
AT1G52400
AT1G54040
AT2G45490
AT1G74160
AT3G53080
AT5G56580
AT1G09540
AT2G15090
AT4G26320
AT3G60220
AT5G03840
AT4G23800
AT1G03170
AT2G34170
AT5G49800
AT1G68570

AT1G11220
AT2G34650
AT5G13790
AT2G43820
AT4G01730
AT4G13990
AT3G18630
AT5G44110
AT5G25470
AT1G68250
AT5G65410
AT1G65920
AT1G09350
AT1G71830
AT5G10430
AT3G14240
AT5G15660
AT1G78090
AT1G10380
AT3G19850
AT1G23080
AT1G16850
AT3G05020
AT5G49215
AT2G05990
AT3G23670
AT4G25850
AT4G12730
AT1G57750
AT3G13520
AT1G17060
AT1G09750
AT1G21460
AT1G55740
AT5G59020
AT5G59510
AT3G22880
AT5G15950
AT3G12700
AT1G13250
AT5G57260
AT1G70680
AT2G26330
AT1G03440
AT1G45130
AT1G70990
AT3G22770
AT4G14690
AT3G56260
AT3G05890
AT2G38060
AT2G28620
AT5G62960
AT3G05400
AT1G27670
AT5G65420
AT5G22740
AT4G13700

AT2G22900
AT3G48460
AT4G16460
AT1G35310
AT2G27060
AT1G78390
AT4G13230
AT1G29640
AT3G56480
AT1G27120
AT5G20270
AT2G28330
AT5G24570
AT2G02780
AT2G38110
AT5G22140
AT2G01660
AT5G39320
AT4G11180
AT2G33560
AT2G16440
AT1G19940
AT2G27130
AT1G66730
AT1G10370
AT4G28703
AT1G29660
AT4G37295
AT3G13000
AT5G15310
AT1G77760
AT5G18600
AT2G29130
AT2G24230
AT3G08030
AT2G37560
AT3G22830
AT3G14890
AT1G60390
AT1G29395
AT2G43800
AT3G06140
AT2G25530
AT1G66760
AT3G60530
AT1G69480
AT1G62050
AT5G63650
AT1G11740
AT1G58340
AT2G35730
AT3G22790
AT4G04460
AT5G52900
AT5G23910
AT1G67630
AT5G10620
AT4G27730

AT1G35780
AT4G22250
AT3G61930
AT5G11510
AT4G22756
AT1G56430
AT2G26580
AT3G19820
AT3G03170
AT1G10030
AT4G36360
AT2G35585
AT1G67600
AT5G66310
AT3G44610
AT5G11230
AT2G24170
AT5G50420
AT3G01550
AT1G12380
AT2G44130
AT4G09170
AT5G47380
AT1G71870
AT1G53160
AT4G36105
AT1G34430
AT5G39890
AT5G15960
AT3G43270
AT5G18660
AT2G42200
AT2G43550
AT3G59140
AT3G63060
AT5G62360
AT2G26640
AT4G12490
AT5G42720
AT1G02340
AT4G02100
AT4G22010
AT5G64850
AT5G37980
AT3G04470
AT1G29670
AT4G37770
AT1G02450
AT1G10560
AT5G47230
AT1G21060
AT4G30410
AT2G29370
AT4G40010
AT5G41460
AT3G06035
AT3G02170
AT5G15740

AT3G13690
AT2G34850
AT5G16080
AT2G22600
AT5G25830
AT1G68400
AT2G28740
AT5G43870
AT4G22290
AT3G62630
AT2G41650
AT3G03680
AT3G04850
AT3G52370
AT2G46970
AT5G46570
AT5G46710
AT4G30650
AT1G22980
AT4G11450
AT1G65370
AT2G35620
AT4G14020
AT2G42300
AT2G45900

Supplementary material 9

Supplementary material & methods

Microarray preparation and analysis

Total RNA was isolated from control and 6 days waterlogged *Arabidopsis* whole plants (both OE lines and WT) by QIAGEN RNeasy(R) Plant Mini Kit. The yield and RNA purity were measured by NanodropND 2000 and Bioanalyser. The mRNA from control and treated roots was used as probe against Arabidopsis_GXP_4x44K AMADID slide. RNA labeling and single microarray hybridization involved 2 technical replicates. Each oligo-nucleotide was randomly replicated and printed on the array. Briefly, 0.5 µg of total RNA was amplified by the Agilent's Quick-Amp labeling Kit (p/n5190-0442) and labeled with Cy3-CTP. T7 promoter based-linear amplification method was used to generate labeled complementary RNA for One-Color Microarray-Based Gene Expression Analysis. In total, 0.825 µg Cy-labeled cRNA was fragmented to an average size of about 50–100 nucleotides by incubation with fragmentation buffer (Agilent Technologies) at 60 °C for 30 min. The fragmented labeled cRNA was then pooled and hybridized to Arabidopsis_GXP_4x44K AMADID slide with Agilent's In situ Hybridzation kit following manufacture's protocol. After washing and blow-drying with a nitrogen gun, microarrays were scanned with Agilent microarray scanner (Agilent Technologies) at 535 nm for Cy3. Scanned images were analyzed by Agilent Feature Extraction Software (v10.7) to quantify signal and background intensity for each feature. The Microarray images were overall clean showing uniform intensity with very low background noise. GeneSpring GX 12.6 Software determined the normalization and statistical significance. In intra array normalization, gProcessed signal (dye normalized background subtracted signal intensity) is log transformed and then for each of the array the 75th percentile value is calculated separately. In each sample the log transformed intensity values for each probe is subtracted by the calculated 75th percentile value of the respective array and expression values are obtained. Fold induction values from both the microarray analysis were normalized to a single fold value. For filtering up-regulation we consider flag should be Detected in the treated sample and can be compromised or Detected in the control samples and Fold >=2 in treated. For filtering down-regulation we consider flag can be Detected or compromised in the treated sample and should be Detected in the control sample and Fold <=2 in treated. *P* values were adjusted for 2 experimental replicates using Student's t-test. Fold induction values from both the microarrays along with their normalized fold induction and adjusted *P* values for individual genes are given in Supplementary material 2, 3 and 4. Responsive genes were classified into functional and pathway categories by DAVID database (Supplementary material 5). The microarray data described in this study have been processed for deposition in Gene Expression Omnibus

with GEO accession number: GSE64070. Microarray expression data were visualized using the Mapman 3.6 software (Thimm *et al.* 2004), and functional bins were identified as being significantly responsive to waterlogging stress. Bins with their name and p-value corrected with Wilcoxon rank sum test are given in Supplementary material 6.

Quantification of total Carbohydrate content

Carbohydrate estimation was done by measuring absorbance at 490 nm by Chow and Landhäusser 2004, Qi et al. 2012. For this 200 mg of whole plant tissue was taken; carbohydrates were hydrolyzed and extracted by boiling in 2.5N HCl for 3 hours, cooled to (room temperature) RT and neutralized by Sodium carbonate till the effervescence disappear. Volume was made to 50 ml and to 0.5 ml of the sample added 250 µl of 4% phenol and 1.25 ml 96% Sulphuric acid, mixed properly and OD taken. Standard curve was prepared by diluting 50µg/ml of standard glucose solution.

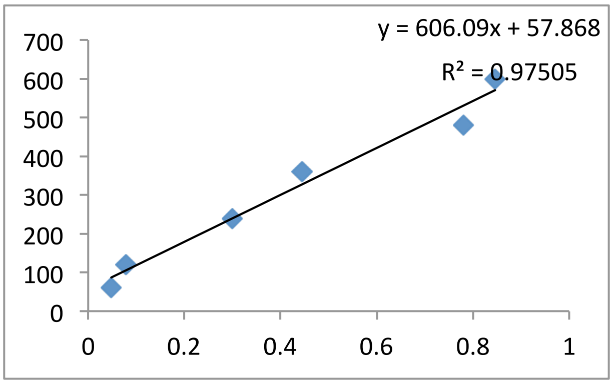

$$\text{Concentration}\left(\frac{\text{mol}}{\text{g}}\right) = \frac{x(\text{gm})}{\text{Mol. wt}\left(\frac{\text{gm}}{\text{mol}}\right) * \text{weight (gm)}}$$

X (gm) = mass of sugar sample deduced from graph

Mol.wt = mol.wt of monosaccharide/polysaccharide

Weight = fresh weight of the sample

Quantification of total soluble protein

Protein estimation was done by Bradford method using Bradford reagent (CBB G250). Approximately 200 mg tissue was taken and ground finely in 1 ml of extraction buffer (100 mM KPO4 -pH 7.8, 1 mM EDTA, 1% Triton X-100, and 10% glycerol), vortexed for 3 seconds and incubated at room temperature for 30 minutes to extract protein, again vortexed and centrifuged at 5000g for 10 minutes, pellet discarded and supernatant was taken. To 1 ml of sample 5 ml of 1:4 diluted Bradford dye was added and mixed properly and absorbance was taken at 595 nm against blank (dye reagent + NaOH). Protein contents of all the samples were deduced from the standard curve prepared with BSA concentrations 20, 40, 60, 80, 100 and 120 µg/µl.

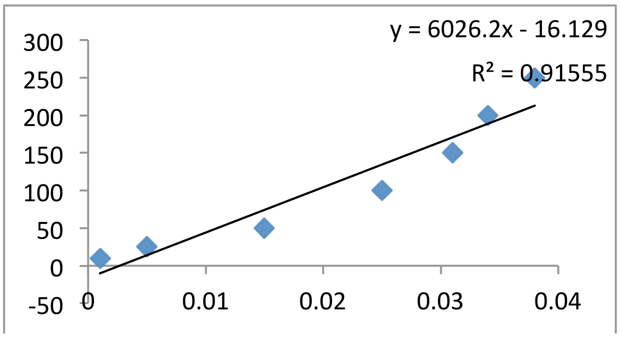

Quantification of total chlorophyll content

Chlorophyll estimation was performed as described by Vernon (1960). Approximately 100 mg of deveined leaf tissue was taken and ground to a fine paste in 80% chilled acetone and the suspension was subjected to centrifugation at maximum speed for 15 minutes. Pellet discarded and supernatant was taken, final volume was made to 10 ml by 80% chilled acetone. Absorbance was taken at 663 nm and 645 nm wavelengths. Amount of chlorophyll was calculated using following formula

$$\text{Total chlorophyll content} = \frac{(\text{A663}\times 8.02) + (\text{A645}\times 20.2)\times \text{Total volume}}{\text{Volume of solution used for abs.}\times \text{Gram fresh wt. of tissue taken}}$$

Measurement of Relative Water Content

The relative water content (RWC) was determined as described by Yamasaki and Dillenburg (1999). Leaves were cut from treated plants and their fresh weight (FW) was measured. To determine turgid mass (TM), the leaves were floated in deionized water for 7 hours. Finally, the leaf samples were dried in an oven at 80°C for 48 hours to obtain the dry weight (DW). The RWC was then calculated using the following formula

$$\text{RWC\%} = \frac{(\text{FW} - \text{DW})}{(\text{TM} - \text{DW})}\times 100$$

Measurement of Lipid peroxidation

Lipid peroxidation was analyzed by the thiobarbituric acid test, which determines MDA as an end product of lipid peroxidation (Hodges et al., 1999). For this 50 mg of whole plant tissue was crushed in 1 ml of phosphate buffer pH 7.4, centrifuged at 48ºC for 20 minutes, mixed with 500 µl 20% TCA and incubated on ice for 30 minutess. Added 500 µl of 0.65% of TBA and incubated at 95ºC for 30 minutes. Centrifuged and absorbance of the supernatant was measured at 532 nm. The MDA concentration was calculated from the extinction coefficient 155 mM^{-1} cm^{-1} using the following formula

$$MDA\ level = [\frac{OD532\times 100}{1.56}]\times \frac{TV}{[wt\times 1000]}$$

OD = optical density; TV = total volume of the extract (ml); wt = weight of the leaf tissue (g)

Extraction for antioxidant activity measurement

For assays of the enzymatic cellular antioxidants whole plant samples (0.4–0.8 g) were homogenized in ice-cold extraction buffer (pH 7.5) containing 50 mM 4-(2- hydroxyethyl)-1-piperazineethanesulfonic acid (HEPES),

0.4mM ethylenediaminetetraacetic acid (EDTA), 5 mM MgCl2, 10 % glycerol, 1% polyvinylpyrrolidone (PVP), 2 mM dithiothreitol (DTT), and 1 mM phenyl methyl sulfonyl fluoride (Gegenheimer, 1990). The homogenate was centrifuged (14,000 g) at 4 °C for 20 min. The supernatant was assayed for enzyme activity.

Quantification of Catalase activity

Catalase assay was done as described by Sinha (1972). The extract (0.5 ml) was added to the reaction mixture containing 1 ml of 0.01 M phosphate buffer (pH 7.0), 0.5 ml of 0.2 M H_2O_2, 0.4 ml H_2O and incubated for different time period. Reaction was terminated by adding the 2 ml of acid reagent (5% $K_2Cr_2O_7$ with glacial acetic acid in 1:3 ratios). To the control, the enzyme was added after the addition of acid reagent. Tubes were heated for 10 minutes and absorbance was recorded at 610 nm. Catalase activity was expressed in terms of – Enzyme unit (EU) of H_2O_2 consumed/min/mg FW using following formula

$$\frac{\Delta C}{min} = \frac{\Delta A}{min} \times \frac{1}{pathlength} \times \frac{1}{extinction\ coefficient}$$

Quantification of SOD activity

Total **SOD activity** was assayed according to Das et al. (2000). The 3 ml reaction mixture contained 50 mM potassium phosphate buffer (pH 7.8), 13 mM methionine, 75 mM NBT, 2 mM riboflavin, 0.1 mM EDTA, and 100 µl enzyme extract. Reaction mixture was illuminated for 15 minutes at a light intensity of 5000 lx. One unit of SOD activity was defined as the amount of enzyme required to cause 50 % inhibition of the reduction of NBT as monitored at 560 nm.

$$\%\ \text{Scavenging} = \frac{\text{Absorbance control} - \text{Absorbance sample}}{\text{Absorbance control}} \times 100$$

Quantification of GPx activity

GPx activity was measured according to the procedure of Rotruck et al. (1973) with some modifications. The reaction mixture consisting of 0.4 ml of 0.4 M sodium phosphate buffer (pH 7.0), 0.1 ml of 10 mM NaN_3, 0.2 ml of 4 mM reduced glutathione, 0.1 ml of 2.5 mM H_2O_2, 0.2 ml of water and 0.5 ml of enzyme was incubated at 0, 30, 60, 90 seconds respectively. Reaction was terminated with 0.5 ml of 10% TCA and after centrifugation; 2 ml of the supernatant was added to 3 ml of phosphate buffer and 1ml of DTNB reagent (0.04% DTNB in 1% sodium citrate). The color developed was read at 412 nm and the enzyme activity is expressed in terms of EU of glutathione utilized/min/mg FW using the following formula

$$\frac{\Delta C}{min} = \frac{\Delta A}{min} \times \frac{1}{pathlength} \times \frac{1}{extinction\ coefficient}$$

DPPH radical scavenging assay

For DPPH radical scavenging activity aliquots of extracts were made to total volume of 3 ml using methanol. 0.15 ml of freshly prepared DPPH solution (98µg/ml) was added, stirred and left to stand at RT for 30 minutes in dark. The control contains only DPPH solution in methanol while methanol served as the blank (negative control). The reduction capability of DPPH radical was determined by the decrease in its absorbance at 517 nm (Patel et al., 2011).

$$\%\ \text{Scavenging} = \frac{\text{Absorbance control} - \text{Absorbance sample}}{\text{Absorbance control}} \times 100$$

FRAP assay

Total antioxidant activity by FRAP assay was done according to Benzie and Strain (1996). 1.5 ml of freshly prepared FRAP solution, containing 25 ml of 300 mM acetate buffer pH 3.6, 2.5 ml of 10 mM TPTZ in 40 mM HCl, and 2.5 ml of 20 mM FeCl3•6H2O solution, was mixed with 1ml of extracts, and absorbance was read at 593 nm. The standard curve was linear between 100 and 500 µM FeSO4•7H2O. Results are expressed in µg Fe(II)/mg FW plant material and compared with that of ascorbic acid.

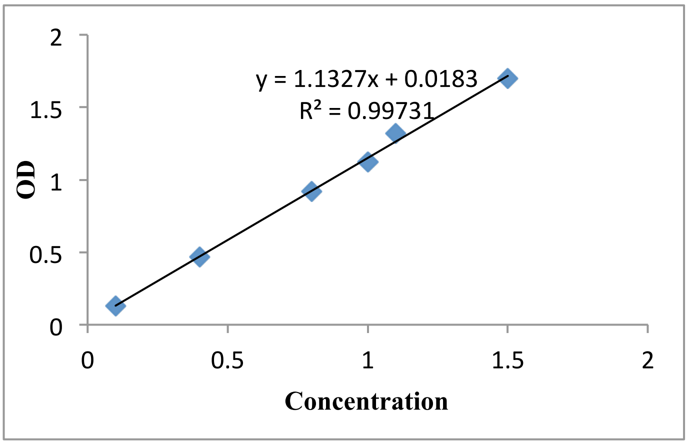

Quantification of GSH

For Estimation of GSH Boyne and Ellman (1972) method was applied. 1ml of the sample extract was treated with 4 ml of metaphosphoric acid precipitating solution (1.67 g of glacial metaphosphoric acid, 0.2 g EDTA and 30 g NaCl in 100 ml water). After centrifugation, 2.0 ml of the protein-free supernatant was mixed with 0.2 ml of 0.4 M Na_2HPO_4 and1 ml of DTNB reagent (40 mg DTNB in 100 ml of aqueous 1% $Na_3C_6H_5O_7$).

Absorbance was read at 412 nm within 2 minutes. GSH concentration was expressed as µg/mg FW using following formula using standard graph

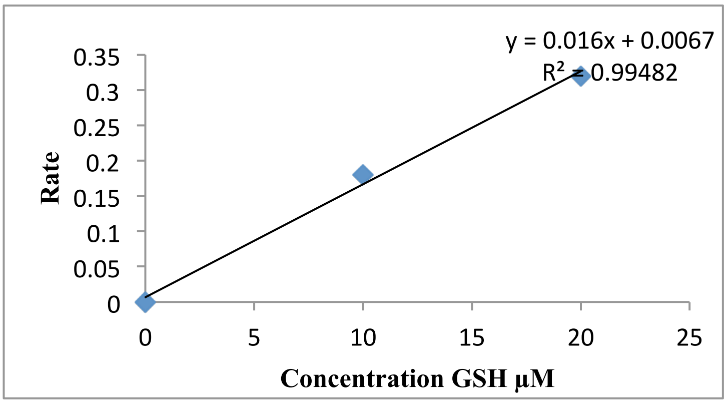

Quantification of NO scavenging activity

50 µl of test sample previously dissolved in DMSO was added to 200 µl of 10 mM Sodium Nitroprusside and incubated at room temperature for 2 hours. From it 50 µl of the sample was taken and to it added 100 µl of Griess reagent (1 part of the 0.1 % Naphthalene Diamine Dihydrochloridein water and 1 part 1% sulphanilamide in 5% concentrated phosphoric acid). The absorbance of chromophore formed was measured at 546 nm on UV-visible spectrometer (Patel et al. 2011). NO scavenging was calculated using following formula:

$$\%\ \text{Scavenging} = \frac{\text{Absorbance control} - \text{Absorbance sample}}{\text{Absorbance control}} \times 100$$
